# Preassembly‐Controlled Radical Recombination at Bismuth: Decarboxylative C─N Coupling with Sulfonamides

**DOI:** 10.1002/chem.202500396

**Published:** 2025-04-21

**Authors:** Elina K. Taskinen, Dominik Birnthaler, Vid Kermelj, Burkhard König

**Affiliations:** ^1^ Faculty of Chemistry and Pharmacy University of Regensburg Universitätsstr. 31 93053 Regensburg Germany; ^2^ Faculty of Chemistry and Chemical Technology University of Ljubljana Večna pot 113 Ljubljana 1000 Slovenia

**Keywords:** bismuth phochemistry, decarboxylative C─N coupling, preassembly

## Abstract

Persistent transition metal radicals form the foundation for many metallaphotoredox protocols. Their ability to efficiently trap organic radicals and convert them into various coupling products has inspired the exploration of selective radical reactions even beyond the d‐block. Radical processes involving bismuth hold great potential, but innovative strategies are required to control the reactivity of bismuth intermediates. Herein, we report preassembly as a powerful strategy to enforce a selective recombination of a bismuth(II) radical and an organic radical. As a result, an inner‐sphere pathway is accessed, enabling the formation of C─N coupling products.

## Graphical abstract

Radical processes involving bismuth hold great potential, but innovative strategies are required to control the reactivity of bismuth intermediates. Herein, we report a direct photochemical decarboxylation with molecular bismuth for C─N coupling of benzylic carboxylic acids and sulfonamides. While highly reactive and hard‐to‐control bismuth radicals are formed, preassembly exerts control over the reaction outcome.

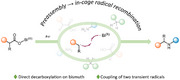

## 1 Introduction

**Scheme 1:**
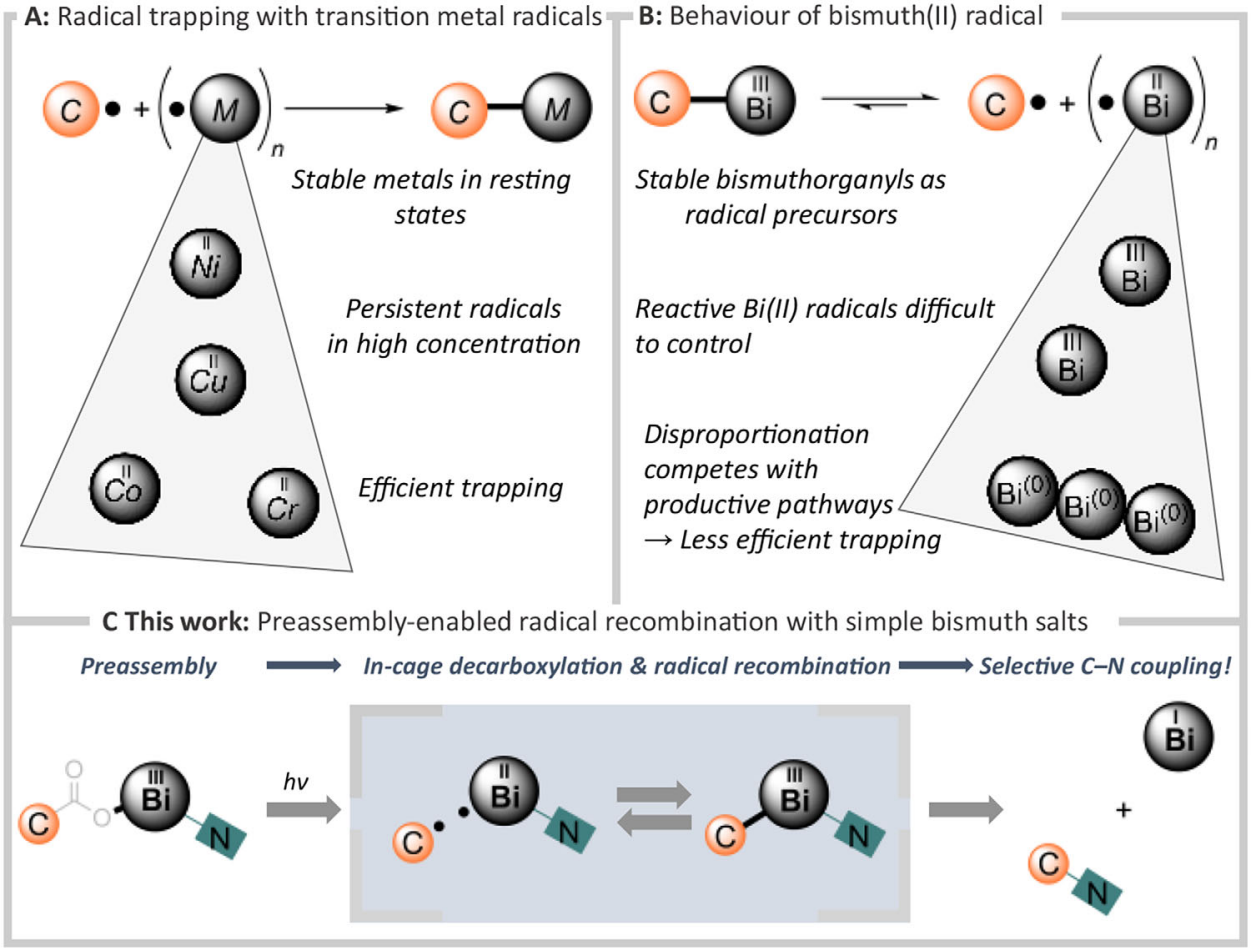
Radical trapping with persistent transition metal radicals (A), behaviour of bismuth(II) radical (B) and the concept of this work (C).

Photochemistry has provided mild and facile means to convert easily accessible, bench‐stable feedstock chemicals into corresponding radical intermediates.^[^ ^1^, ^2^, ^3^, ^4^ ^]^ However, despite the ease of the radical generation, directing their downstream reactivity has turned out to be fundamentally more challenging due to their high reactivity and short lifetimes.^[^ ^5^ ^]^ As a result, swift radical trapping with organic radical acceptors such as activated olefins or electron‐rich heteroarenes has been established as a successful strategy toward selective C─C bond formations.^[^ ^6^, ^7^ ^]^ Moreover, the merger of transition metals with light‐driven processes has enabled the trapping of the initially generated radical into organometallic intermediates, where the follow‐up reactions of the organic fragment are highly dependent on the metal centre (Scheme 1a).^[^ ^8^ ^]^ For instance, with nickel, the trapped radical typically undergoes reductive elimination or is cleaved off from the metal centre by radical substitution (S_H_2 mechanism).^[^ ^9^, ^10^, ^11^ ^]^ Cobalt, in contrast, provides access to unsaturated organic products via a *β*‐hydride elimination.^[^ ^12^, ^13^ ^]^ Organic substrates bound to chromium and titanium, in turn, display carbanion‐like reactivity and are often used to attack carbonyl electrophiles.^[^ ^14^, ^15^ ^]^ Copper is well known for its ability to oxidize radicals to carbocations and even simple copper salts are widely used as powerful terminal oxidants in many radical processes.^[^ ^16^, ^17^ ^]^ Despite the diversity of these transition metal‐based methods, their reactivity is largely based on the same concept: the persistent radical effect.^[^ ^18^ ^]^ While the stability of these metal radicals favours the radical trapping process, the resting nature of the radical (oxidation)states simultaneously makes them rather unreactive toward the closed‐shell starting materials. Thus, a second catalyst is needed to activate the organic substrates, requiring fine adjustments between multiple catalytic systems.^[^ ^8^, ^19^ ^]^

In recent years bismuth redox chemistry has emerged as a transition metal–like platform for synthesis and catalysis, broadening elementary steps like oxidative addition and reductive elimination beyond the d‐block.^[^ ^20^, ^21^, ^22^ ^]^ The enabling factor in the bismuth redox chemistry is the easy thermal or photochemical homolysis of bismuth‐carbon and bismuth‐heteroatom bonds combined with the metal's effortless shuttling between various oxidation states.^[^ ^23^, ^24^, ^25^, ^26^, ^27^ ^]^ In the realm of recent reports on bismuth redox transformations, we were particularly inspired by the work of Cornella detailing the radical recombination/dissociation equilibrium between their *N*,*C*,*N*‐pincer ligand‐stabilized Bi(II) complex and an α‐amino alkyl radical.^[^ ^28^ ^]^

Given our previous report with ligand‐free bismuth redox chemistry, we wondered whether we could utilize simple bismuth salts for a similar radical trapping process (Scheme 1b).^[^ ^29^ ^]^ However, in strong contrast to transition metals, main group metal radicals without specially designed ligands are usually transient in nature, making them prone to undesired dimerization and disproportionation.^[^ ^30^, ^31^ ^]^ Therefore, simple bismuth radicals cannot be added directly to the reaction mixture but need to be generated in situ. Although this presents a challenge, it also provides the opportunity to utilize a single metal center in two consecutive key steps, first activating the organic substrate by single electron transfer (SET) and then trapping the formed radical intermediate. Herein, we realized that our success relied on making the rate of the desired cross‐coupling faster than the competing homocoupling and disproportionation processes.^[^ ^18^ ^]^ Rather than relying on diffusion‐controlled combination of reagents, we envisioned that bringing the reagents together prior to the onset of the reaction could increase the probability of the following cross‐coupling event.

Over the past years, preassembly has been established as an innovative strategy to make the unfavored processes possible.^[^ ^32^ ^]^ As an example, earlier this year the Wolf group addressed the challenges stemming from the short excited state lifetimes of their cobalt photocatalyst by pre‐coordinating the excited state quencher before irradiation.^[^ ^33^ ^]^ To facilitate the preorganization in our system, we considered carboxylic acids as potent radical precursors, as they typically form metal carboxylates, setting the stage for ligand‐to‐metal charge transfer (LMCT) reactivity. Upon excitation, the bismuth(II) radical and substrate‐derived radical are generated simultaneously in a codependent manner. Since LMCT decarboxylations typically follow an inner‐sphere mechanism,^[^ ^34^, ^35^ ^]^ the substrate radical and bismuth radical are likely staying in close proximity, favoring the subsequent radical recombination. While many molecular bismuth carboxylates have been structurally characterized,^[^ ^36^, ^37^, ^38^, ^39^ ^]^ their potential for photochemical transformations has remained underutilized. However, indications on the photoactivity of bismuth carboxylates from our earlier study gave us confidence that this reaction mode could be obtainable with the right set of conditions.^[^ ^29^ ^]^

## 2 Results and Discussion

### 2.1 Optimization

We started our optimization studies by using equimolar amounts of the carboxylic acid **1a**, sulfonamide **2a,** and a base to identify the best‐performing bismuth precursor under 365 nm irradiation. Following our previous study on bismuth LMCT photoactivity, we decided to focus our screening on inexpensive and commercially available salts (Tables 1 and S2). We found that the reaction outcome was highly dependent on the bismuth precursor utilized. While many salts (such as Bi(NO_3_)_3_, Bi(OAc)_3_, and BiOCl) did not give any conversion of the starting material, both BiCl_3_ and Bi(OTf)_3_ were able to decarboxylate the benzylic acid **1a** (Entries 1 and 2). However, only Bi(OTf)_3_ afforded the desired cross‐coupling product **3a,** whereas with BiCl_3_ the homocoupling of the benzylic radical was mainly observed. Similarly to previous observations on LMCT decarboxylations, we also found the combination of a hydrogencarbonate base and DCM as a solvent to give the best yield.^[^ ^17^ ^]^ After a broad screening of possible variables (see Supporting Information), a significant yield improvement was obtained when either the sulfonamide (Entry 3) or the acid (Entry 4) was used in excess. This observation led to two optimal conditions: in **conditions A** the acid partner serves as the limiting reagent combined with 3.5 equivalent of the nucleophile, whereas in **conditions B** the sulfonamide counterpart is the limiting reagent, and 3.0 equivalents of the carboxylic acid are used. Whereas conditions A worked reliably over a broad variety of carboxylic acids, conditions B proved to be particularly useful in the cases where the sulfonamides were only sparingly soluble in DCM. Apart from the solubility considerations, the dual nature of our optimized conditions leaves room for a flexible choice of reagent in excess depending on other factors such as starting material availability and costs. Using Bi(OTf)_3_ in catalytic amounts in combination with a chemical oxidant, such as NFSI, peroxides, or persulfates, led to only trace amounts of the product (Entries 5 and 6, Table S8). Although the reoxidation of bismuth would be mechanistically interesting, the simplicity and economical feasibility of using stoichiometric bismuth triflate is well comparable to commonly used oxidants.

**Table 1:** Optimization of the reaction conditions.

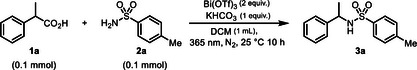			
Entry		Deviation	Yield [%]^[^ ^a^ ^]^
1	As above		32
2	BiCl_3_ instead of Bi(OTf)_3_		*traces*
3^[^ ^b^ ^]^	**A**	Sulfonamide (3.5 equiv.)	60
4^[^ ^b^ ^]^	**B**	Acid (3.0 equiv.)	60
5	Bi(OTf)_3_ (20 mol%) + NFSI (2 equiv.)		11
6	Bi(OTf)_3_ (20 mol%) + (NH_4_)_2_S_2_O_8_ (2 equiv.)		*traces*
	Control Experiments		
7	No Bismuth		n.d.
8	No Light		n.d.
9	60 °C dark		n.d.

^[a]^ Calibrated GC‐FID yield.

^[b]^ KHCO_3_ (3 equiv.). NFSI = *N*‐Fluorobenzenesulfonimide

Finally, control experiments confirmed the necessity of bismuth salt and photochemical nature of our transformation. In the absence of a bismuth source, no product could be detected (Entry 7). No product was also observed in the absence of light either at room temperature or upon heating to 60 °C (Entries 8 and 9).

### 2.2 Scope of the Carboxylic Acids

**Scheme 2:**
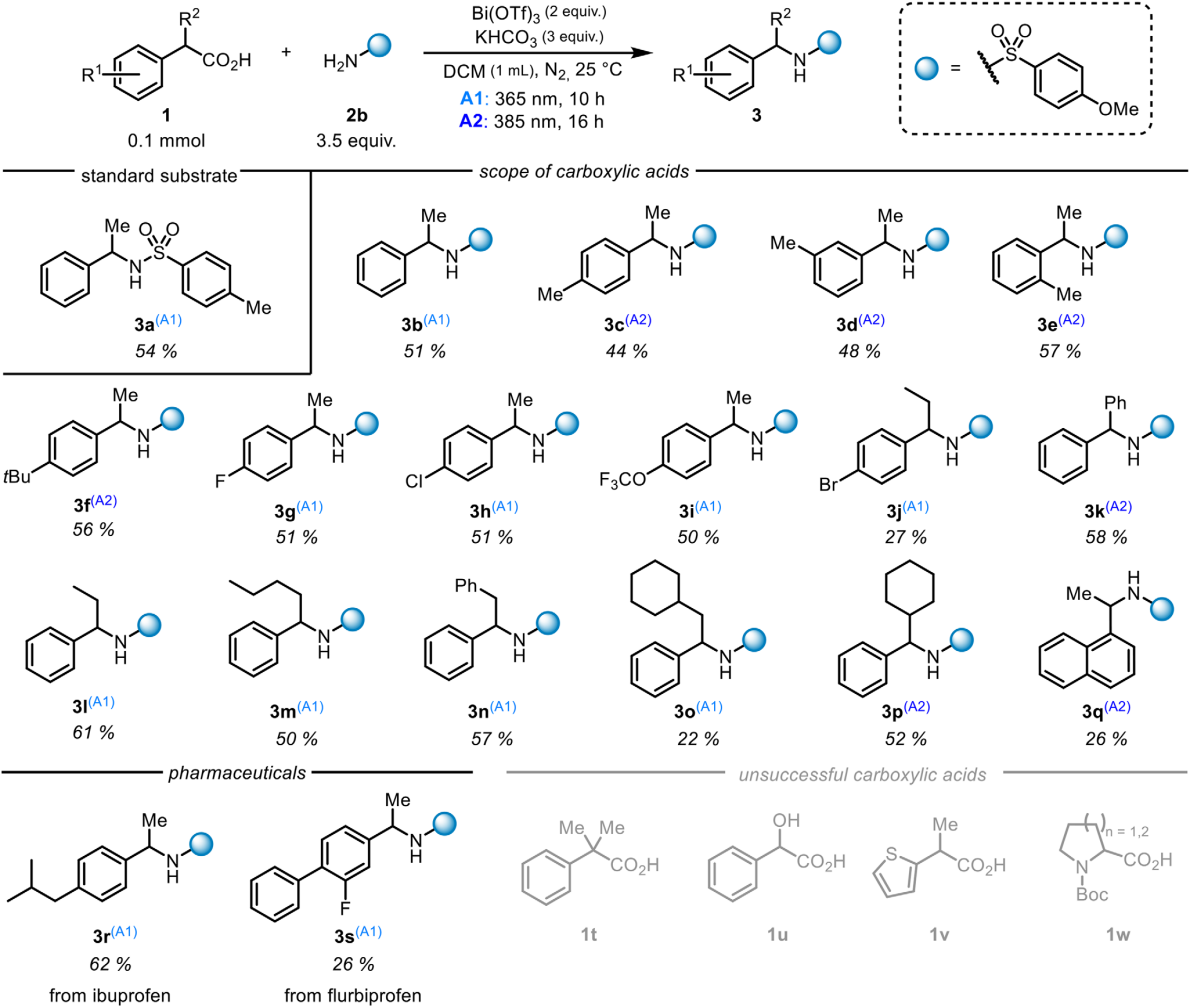
Scope of carboxylic acids.

Next, we studied the scope of the transformation (Scheme 2). (±)‐2‐Phenylpropanoic acid (**1a**) was smoothly converted into the C─N coupling products with *p*‐methylphenyl and *p*‐methoxyphenyl sulfonamides (products **3a** and **3b**). Different methylation patterns were well tolerated, and particularly, the *ortho*‐methylated acid gave a high yield (**3c**, **3d,** and **3e**). A *tert*‐butyl group was also compatible as an electron‐donating substituent in the *para*position (**3f**). We also noticed that the reactions of the electron‐rich carboxylic acids were best carried out with 385 nm irradiation to avoid undesired degradation. For electron‐withdrawing substituents, halogens such as fluorine (**3g**) and chlorine (**3h**) together with a trifluoromethoxy group (**3i**) gave comparable yields to the standard compound. The brominated compound **3j** was obtained in a somewhat lower yield due to debromination accompanying the desired product‐forming events.

Several *alpha‐*substituted carboxylic acids were successfully converted to the products. A phenyl, ethyl, and *n*‐butyl substituents were well compatible (**3k**, **3l,** and **3** **m**). An aliphatic cyclohexyl ring was also tolerated both with a CH_2_‐linker (**3o**) and when directly attached to the benzylic position (**3p**). The positive results obtained here were particularly encouraging as they demonstrate the significant selectivity of our method toward the C─N coupling over the alternative alkene formation. Particularly worth mentioning here is the conversion of 2,3‐diphenylpropanoic acid to the desired coupling product **3n** without any observable stilbene generation. The 1‐naphthyl substituted carboxylic acid also underwent the desired transformation, although the yield might have been slightly reduced by the direct excitation of the aromatic system (**3q**). Finally, we applied our method for the late‐stage modification of pharmaceutically active compounds, giving products **3r** and **3s** stemming from ibuprofen and flurbiprofen, respectively.

Regarding the limitations, tertiary carboxylic acids (such as **1t**) typically underwent decarboxylation, but most likely due to the steric hindrance, only homocoupling of the formed benzylic radical was observed. Addition of a free hydroxy group to the benzylic position (**1u**) effectively shut down the reaction probably by forming a bidentate ligand on bismuth from which no decarboxylation could take place. Amino acids (such as **1w**) formed insoluble species with bismuth in DCM and no further reactivity was observed.

### 2.3 Scope of the Nucleophiles

**Scheme 3:**
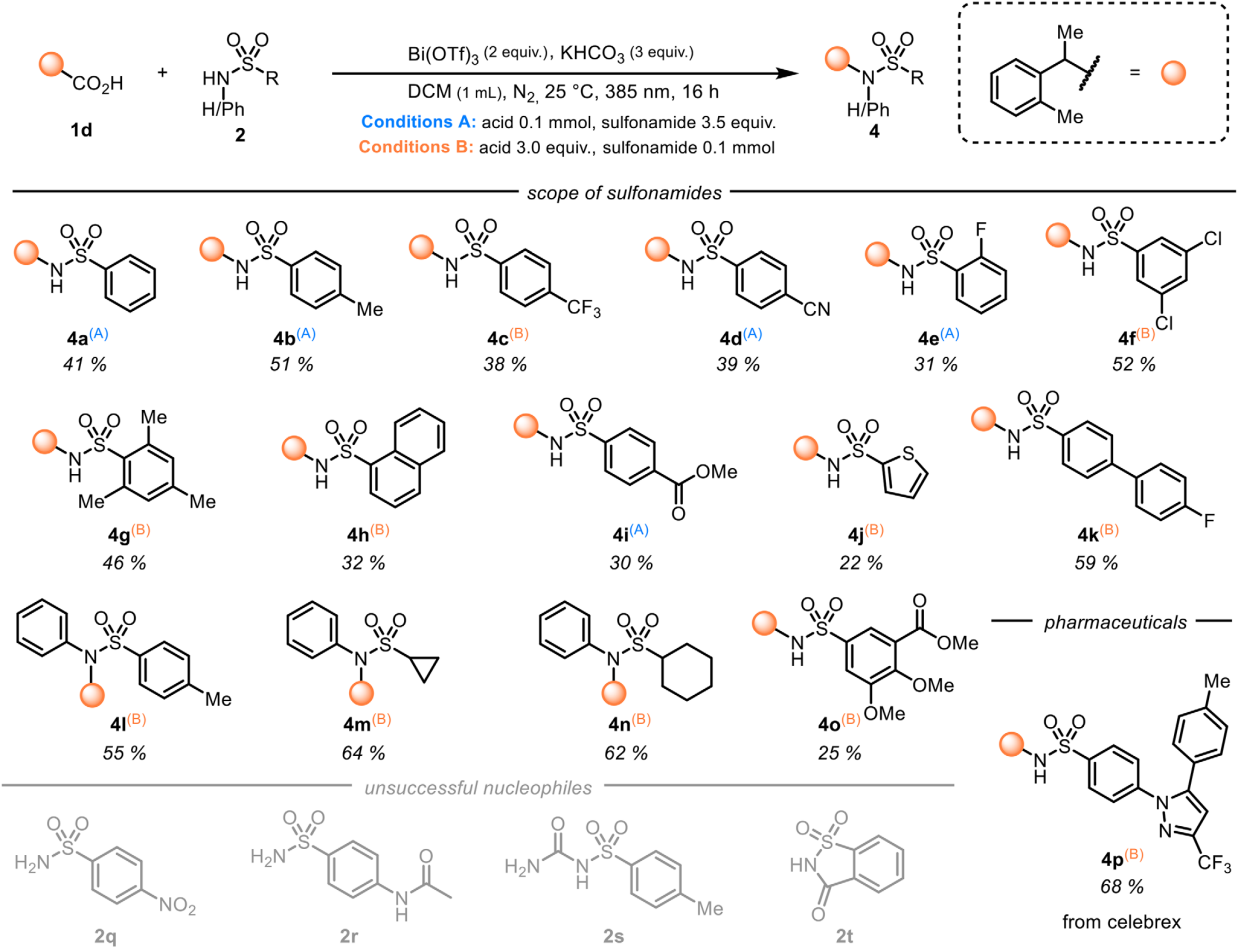
Scope of the nucleophiles.

We then turned our attention to the scope of the nucleophiles (Scheme 3). The phenyl‐substituted sulfonamide yielded the desired product **4a** in a synthetically useful 41% yield, whereas an addition of a methyl group to the *para*‐position gave a higher isolated yield of **4b**. In terms of electronic effects, *para*‐trifluoromethyl and ortho‐fluorine substituted sulfonamides (**4c** and **4e**) resulted in slightly lowered yields implying that extra electron density on the sulfonamide, which would also increase the nucleophilicity, is beneficial for the reaction. When electron‐withdrawing substituents were placed in nonconjugated *meta*‐positions, high yields were retained (**4f**). Notably, selective coupling reactions were also obtained in the presence of a competing nucleophilic CN‐group (**4d**) or a competing electrophilic ester‐group (**4i**). For the electron donating group, a mesityl ring and 1‐napthyl substituents (**4g** and **4** **h**) as well as a 4′F‐biphenyl group (**4k**) could also be included in the sulfonamide. The thiophene‐derived sulfonamide gave the desired product **4j** in 22% yield.

Steric demand at secondary *N*‐phenyl sulfonamides was well tolerated. *N*‐phenyl‐methylphenyl sulfonamide gave the tertiary product **4l** in a 55% yield. Moreover, sulfonamides with an aliphatic ring attached to the sulfonyl group were also compatible, giving products **4** **m** and **4n** in over 60% yield. Moreover, the highly substituted sulfonamide product **4o** could be obtained. Finally, the pharmaceutically active compound celebrex could be derivatized with our method, giving the product **4p** in a good yield of 68%.

With respect to the limitations on the sulfonamide side, a nitro substituent (**2q**) and an amide functionality (**2r**) were incompatible with the reaction, and no conversion of the starting materials was observed. The carbamoyl‐benzenesulfonamide **2s** and the cyclic sulfonamide saccharin **2t** failed to undergo the desired reaction, perhaps by forming an unreactive chelated ligand to bismuth.

### 2.4 Mechanistic Studies

**Scheme 4:**
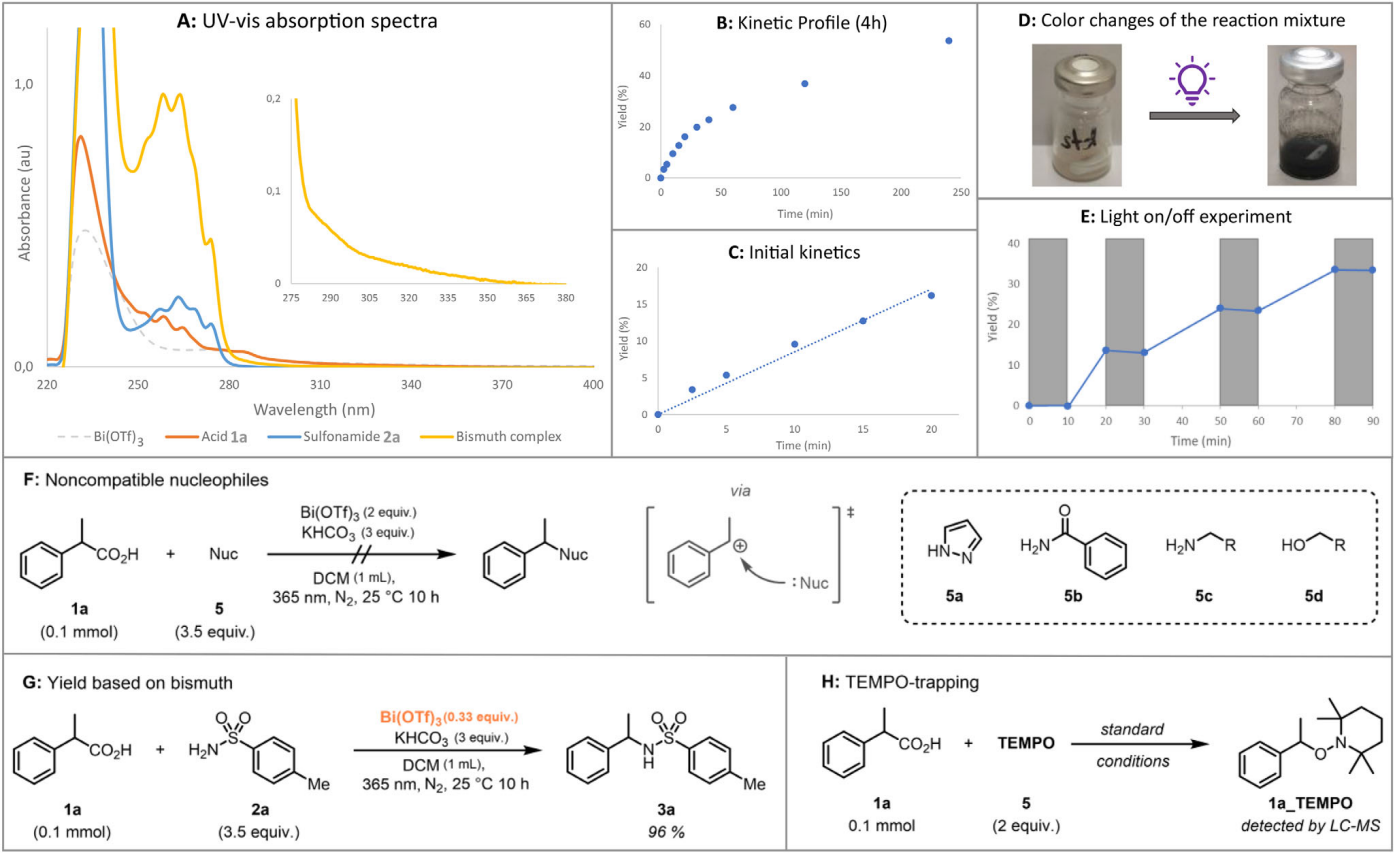
Key mechanistic studies. UV‐vis absorption spectra of the reactants and the *in situ*‐formed complex (A), kinetic profile of the reaction (B), initial kinetics (C), colour change of the reaction mixture (D), light on/off experiment (E), noncompatible nucleophiles tested (F), reaction using bismuth as limiting reagent (G) and TEMPO‐trapping study (H).

To gain a better understanding of our reaction, a set of mechanistic studies was carried out. We started by measuring the UV‐vis spectra of all the reaction components and their combination. As seen from Scheme 4a, Bi(OTf)_3_ had its absorption maximum at 230 nm and did not show any absorbance above 300 nm (gray dotted line). The carboxylic acid **1a** (orange line) and the sulfonamide **2a** (blue line) both exhibited their absorption band between 250 nm and 280 nm, but no absorption could be detected in the range of irradiation. Addition of Bi(OTf)_3_ to the mixture of carboxylic acid and sulfonamide significantly intensified the absorption features accompanied by prolonged tailing (yellow line). Indeed, the tailing of the absorbance reached the area of the irradiation wavelength, thus confirming the role of the *in situ*‐formed bismuth‐centred assembly as the light‐harvesting species (zoom‐in spectrum).

We then measured the kinetic profile of our reaction (Scheme 4b). From the measurement spanning over the first 4 hours of irradiation, we could see that the product formation was rapid at the beginning of the reaction (0–20 minutes) after which the rate of the reaction started to gradually decrease. The product formation was observed immediately upon the start of irradiation, thus supporting our hypothesis that the active bismuth assembly is pre‐formed in the mixture. The initial kinetics, in turn, followed a linear trend without detectable build‐up of intermediates (Scheme 4c). The accumulation of a black solid during the reaction suggests the full reduction of bismuth to its metallic form, perhaps via disproportionation of bismuth(II) and/or bismuth(I), both of which are described as unstable species in the literature (Scheme 4d).^[^ ^40^, ^41^ ^]^ Furthermore, the light on/off experiment shows the necessity of continuous irradiation for the reaction progress, thus rendering a radical chain mechanism unlikely (Scheme 4e).

The final C─N bond‐forming step could either follow an inner‐sphere reductive elimination pathway or go *via* an outer‐sphere mechanism. Literature reports of decarboxylative C─N coupling reactions with copper and iron suggest an outer‐sphere mecha‐nism.^[^ ^17^, ^42^ ^]^ With copper, a well‐established radical oxidation to a carbocation is proposed whereas with iron a chlorinated intermediate stemming from a radical ligand transfer could be detected. In our system, the radical ligand transfer mechanism was found unlikely on a stereoelectronic basis.

To evaluate the outer‐sphere ionic nucleophilic coupling mechanism proceeding *via* a carbocation, we rationalized that the presence of this intermediate would most likely lead to a broad compatibility of nucleophiles. Instead, with our bismuth system, alternative nucleophiles such as pyrazole, amides, amines, and alcohols could not be coupled so far (Scheme 4f). Furthermore, side reactions like esterification or desaturation *via* *alpha*‐deprotonation of a carbocation were rarely observed in our study. On the carboxylic acid side, sterically demanding substrates (such as **1t**), were not tolerated. Taken together, the C─N coupling step in our reaction most likely follows a reductive elimination pathway.

We believe that the exact coordination sphere around bismuth in our system is rather dynamic and not limited to certain coordination patterns since the excess of either carboxylic acid or sulfonamide is beneficial to the reaction. When bismuth is used as a limiting reagent, the highest efficiency of bismuth as a consecutive one electron oxidant is achieved (96% yield, bismuth as limiting reagent, Scheme 4g). Finally, the radical nature of the decarboxylation process was suggested by the trapping of the benzylic radical with TEMPO (Scheme 4h).

**Scheme 5:**
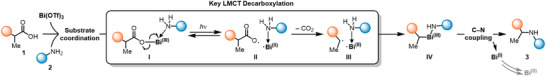
Proposed mechanism.

Based on our mechanistic considerations and the previous literature, ^[^ ^43^, ^44^ ^]^ the following mechanism is proposed (Scheme 5). The product‐forming sequence starts with the coordination of the respective carboxylic acid **1** to the bismuth center assisted by KHCO_3_. The formed bismuth(III) carboxylate assembly **I** is irradiated resulting in the cleavage of the bismuth carboxylate. The formed carboxyl radical **II** can then either recombine with the Bi(II) regenerating the complex **I**, or undergo an irreversible decarboxylation to the carbon‐centered radical **III**. The preassembly of the complex **I** together with the codependent manner of the radical generation, ensures the spatial proximity between the bismuth(II) radical and the carbon‐centered radical **III** thus favoring their recombination. The so‐formed organobismuth compound **IV** then undergoes a reductive elimination to forge the C─N bond upon the release of the product **3**. The bismuth(I) formed in this process is unstable and eventually undergoes disproportionation to metallic bismuth.

## 3 Conclusion

In summary, we report the first direct decarboxylation on bismuth, giving access to C─N coupling products with sulfonamides. The direct use of nonfunctionalized starting materials allows a straightforward and step‐economical method. Importantly, preassembly allowed us to overcome the challenges of controlling the reactivity of transient radicals, thus enabling a selective coupling process. While preassembly has remained as underutilized strategy, we hope that our report encourages its further utilization. Furthermore, we believe that the observation of efficient decarboxylation provides an additional entry point to radical chemistry on bismuth.

## Supporting Information

The authors have cited additional references within the Supporting Information.^[^ ^45^, ^46^, ^47^, ^48^, ^49^, ^50^, ^51^, ^52^, ^53^, ^54^, ^55^ ^]^

## Author Contributions

Elina K. Taskinen and Dominik Birnthaler developed the concept of the project. Vid Kermelj and Dominik Birnthaler explored the general idea. Elina K. Taskinen and Dominik Birnthaler optimized the reaction, synthetized the starting materials and the products, and carried out mechanistic studies. Elina K. Taskinen and Dominik Birnthaler wrote the manuscript and modified the draft according to the feedback from other authors. All authors agreed on the final version of the manuscript. Burkhard König supervised the work.

## Conflict of Interests

The authors declare no conflict of interest.

## Supporting information



Supporting Information

## Data Availability

The data that support the findings of this study are available in the supplementary material of this article.
