# Supplementary material for: Preassembly‐Controlled Radical Recombination at Bismuth: Decarboxylative C─N Coupling with Sulfonamides
Source: Chemistry. 2025 Apr 21;31(28):e202500396. doi: 10.1002/chem.202500396 (PMC12089901; doi:10.1002/chem.202500396)
Supplement: Supplementary file 1 — Supporting Information [file CHEM-31-e202500396-s001.docx]

Supporting Information for

**Preassembly-Controlled Radical Recombination at Bismuth:**

**Decarboxylative C–N Coupling with Sulfonamides**

Elina K. Taskinen, ‡^a^ Dominik Birnthaler, ‡^a^ Vid Kermelj^a,b^ and Burkhard König*^a^

‡These authors contributed equally

^[a]^ Faculty of chemistry and pharmacy, University of Regensburg, Universitätsstraβe 31,

93053 Regensburg, Germany

^[b]^ Faculty of Chemistry and Chemical Technology, University of Ljubljana,
Večna pot 113, 1000 Ljubljana, Slovenia

*Correspondence: [burkhard.koenig@ur.de](mailto:burkhard.koenig@ur.de)

Primary research data to this article can be accessed free of charge online in the

Radar4Chem Repository: doi:10.22000/aygcxy5kjzu130uy

**Table of Contents**

[1. General Information 4](#_Toc183702513)

[1.1 Photosetups 5](#_Toc183702514)

[1.1.1 365 nm (2.0 W) setup 5](#_Toc183702515)

[1.1.2 385 nm (0.5 W) setup 6](#_Toc183702516)

[1.1.3 Emission spectrum of the LEDs 7](#_Toc183702517)

[1.1.4 Reaction mixtures before and after irradiation 7](#_Toc183702518)

[1.2 GC-FID Calibration 8](#_Toc183702519)

[2. Extended optimization studies 9](#_Toc183702520)

[2.1 General procedure for the optimization studies 9](#_Toc183702521)

[2.2 Solvent screening 9](#_Toc183702522)

[2.3 Bismuth source screening 10](#_Toc183702523)

[2.4 Base screening 11](#_Toc183702524)

[2.5 Stoichiometry screening 12](#_Toc183702525)

[2.6 Concentration screening 13](#_Toc183702526)

[2.7 Irradiation source and temperature screening 13](#_Toc183702527)

[2.8 Bismuth loading screening 14](#_Toc183702528)

[2.9 Oxidant screening 15](#_Toc183702529)

[2.10 Control Experiments 15](#_Toc183702530)

[3. Synthesis and characterization of products 16](#_Toc183702531)

[3.1 General procedures for the photocatalyzed nucleophilic couplings 16](#_Toc183702532)

[3.1.1 Carboxylic acid as a limiting reagent (GP1) [see details for each substrate below] 16](#_Toc183702533)

[3.1.1 Sulfonamide as a limiting reagent (GP2) [see details for each substrate below] 16](#_Toc183702534)

[3.2 Scope of carboxylic acids 17](#_Toc183702535)

[3.3 Scope of nucleophiles 26](#_Toc183702536)

[4. Synthesis and characterization of starting materials 35](#_Toc183702537)

[4.1 Synthesis of benzylic carboxylic acids 35](#_Toc183702538)

[4.1.1 General Procedure for methylation of benzylic acids (GP3)^[1]^ 35](#_Toc183702539)

[4.1.2 General Procedure for alkylation of phenylacetic acid (GP4) 35](#_Toc183702540)

[4.1.3 General Procedure for the workup of methylation/alkylation reactions 35](#_Toc183702541)

[4.1.2 List of synthetized carboxylic acids 36](#_Toc183702542)

[4.1.3 List of synthetized unsuccessful carboxylic acids 40](#_Toc183702543)

[4.2 Synthesis of sulfonamide nucleophiles 42](#_Toc183702544)

[4.2.1 General Procedure for the sulfonamide synthesis from sulfonyl chlorides (GP5) 42](#_Toc183702545)

[4.2.2 List of synthetized sulfonamides 43](#_Toc183702546)

[4.2.3 List of synthetized unsuccessful sulfonamides 48](#_Toc183702547)

[5. Mechanistic studies 49](#_Toc183702548)

[5.1 UV-vis measurements 49](#_Toc183702549)

[5.1.1 Uv-vis in Acetonitrile 49](#_Toc183702550)

[5.1.2 Uv-vis in DCM 50](#_Toc183702551)

[5.2 Kinetic studies 52](#_Toc183702552)

[5.2.1 Reaction kinetics 52](#_Toc183702553)

[5.2.2 Initial kinetics 53](#_Toc183702554)

[5.2.3 Light on/off experiment 54](#_Toc183702555)

[5.3 Experiments towards later elementary steps 55](#_Toc183702556)

[5.3.1 TEMPO Trapping Studies 55](#_Toc183702557)

[5.3.2 Studies towards the presence/absence of benzylic carbocations 56](#_Toc183702558)

[5.3.3 BiCl_3_ as bismuth precatalyst 57](#_Toc183702559)

[5.4 Reactions using substoichiometric bismuth 58](#_Toc183702560)

[References 59](#_Toc183702561)

[NMR-Spectra of the isolated products 60](#_Toc183702562)

[NMR-Spectra of the synthetized starting materials 102](#_Toc183702563)

# 1. General Information

**Chemicals and solvents** were purchased from commercial suppliers (Sigma Aldrich, TCl, BLD Pharma, Alfa Aesar, Acros, Fluka or Thermo Fischer) and, unless otherwise noted, used without further purification. Specifically, for the benchmark reaction the following chemicals were used: Bi(OTf)_3_ (99% purity, Thermo Scientific), ($\pm$)-2-phenylpropionic acid (98% purity, ABCR), *p*-toluenesulfonamide (99% purity, Acros), KHCO_3_ (Merck) and DCM (99.8% purity, anhydrous, Sigma Aldrich). All air-and moisture sensitive reactions including photoreactions were set up using pre-dried N_2_ as shielding gas, whereas dry solvents were obtained from commercial suppliers. Evaporation of organic solvents was carried out in a rotary evaporator at temperatures below 40 °C and under reduced pressure.

**Analytical thin layer chromatography (TLC)** was routinely used for the monitoring of starting material synthesis and to quantify the eluation of product. Silica-gel pre-coated aluminium sheets (Macherey-Nagel, silica gel 60G/UV254, 0.2 mm) served as stationary phase, whereas the mobile phase consisted of solvents and solvent mixtures, the ratios of which are reported as v/v solutions. Visualization was performed with a 254 nm UV-light source combined with chromatographic dyes if so noted.

**Flash column chromatography (FCC)** was done either by hand or by a Biotage® IsoleraTM Spektra One machine. In the former case flash silica gel (Merck, particle size 40–63 μm, 230–440 mesh) was used as a stationary phase whereas in the latter case pre-packed Biotage® columns were used. For both techniques eluent mixtures, whenever possible consisting of pre-distilled petroleum ether (PE) and ethyl acetate (EtOAc), was used. For all eluents, the solvent ratios are reported as v/v ratios.

**GC-FID and GC-MS** measurements were performed on a GC 7890 from Agilent Technologies coupled with a FID detector. The system was equipped with a capillary column (HP-5MS UI, 30 m length, 0.25 mm diameter, 0.25 μM film) and run with He (flow rate 1mL/min) as a carrier gas. (**NOTE:** The use of H_2_ as carrier gas in this project was found to result in a complex GC-spectrum, most likely due to reactions within the GC. Therefore, if change of carrier gas is desired, we recommend to test the suitability of the new carrier by comparing against the spectrum obtained with helium). Unless otherwise noted, sample volume of 1 μL was injected (split injection, 40:1 split) at 280 °C with detector temperature 300 °C. The GC-MS measurements were done with a 7890AGC system with an Agilent 5975 MSD detector. For the GC program, the initial temperature of 40 °C was hold for 3 minutes, after which the temperature was increased on a rate of 15°C/min for over 16 minutes. The temperature was ramped until 280 °C, where the temperature was hold constant for 5 minutes. After this the temperature was increased with a rate of 25 °C/min for 48s, until the final temperature of 300 °C was reached and hold again for 5 minutes. Finally, data accusation was done using Agilent ChemStation Rev.C.01.04.

**Nuclear Magnetic Resonance Spectroscopy (NMR)** was recorded at room temperature using a Bruker Avance 400 (400 MHz for ^1^H, 101 MHz for ^13^C, 376 MHz for ^19^F) NMR spectrometer. All chemical shifts are reported δ-scale as part per million [ppm] (multiplicity, coupling constant *J*, number of protons) relative to the solvent residual peak. The coupling constants *J* are given in Herz [Hz] and the multiplicity of the signals are abbreviated as: singlet (s), broad singlet (br. s.), doublet (d), doublet of doublets (dd), doublet of doublet of doublets (ddd), triplet (t), doublet of triplets (dt), triplet of doublets (td), quartet (q), doublet of quartets (dq), pentet (p) and multiplet (m). For the NMR measurements deuterated solvents (CDCl_3_ from deutero, DMSO-*d*_6_ from Sigma Aldrich) were used. The spectra were processed using MestreNova 9.0.1.

**High Resolution Mass Spectra (HRMS)** were performed at the Central Analytical Laboratory of the departments of chemistry and pharmacy at the University of Regensburg. For measurements, > 1 mg of the sample submitted, which was later dissolved to acetonitrile, DCM or ethyl acetate. The samples were then injected either to a Jeol AccuTOF GCX or to an Agilent Q-TOF 6540 UHD instrument.

## 1.1 Photosetups

### 1.1.1 365 nm (2.0 W) setup

For the photoreactions run at 365 nm (1.5 W to 2.0 W) were performed using custom-made photoreactors depicted below (Figure S1). At the core of the photoreactors reside a metal block to which six single-spot LEDs of desired wavelength are mounted. On top of the LED block sits a hollow cooling block made out of stainless-steel through which a constant flow of water is possible. This cooling block also contains holes made to perfectly fit 5 mL crimp-cap vials thus providing a sideway contact of the reaction vessels to the cooling system. Magnetic stirring for the reactions is imposed from underneath the LED blocks using Heidolph MR 3000 stirring plates. Finally, the water cooling is sustained with a Julabo Curio Co unit. **NOTE:** Due to the modular nature of these setups, they can be


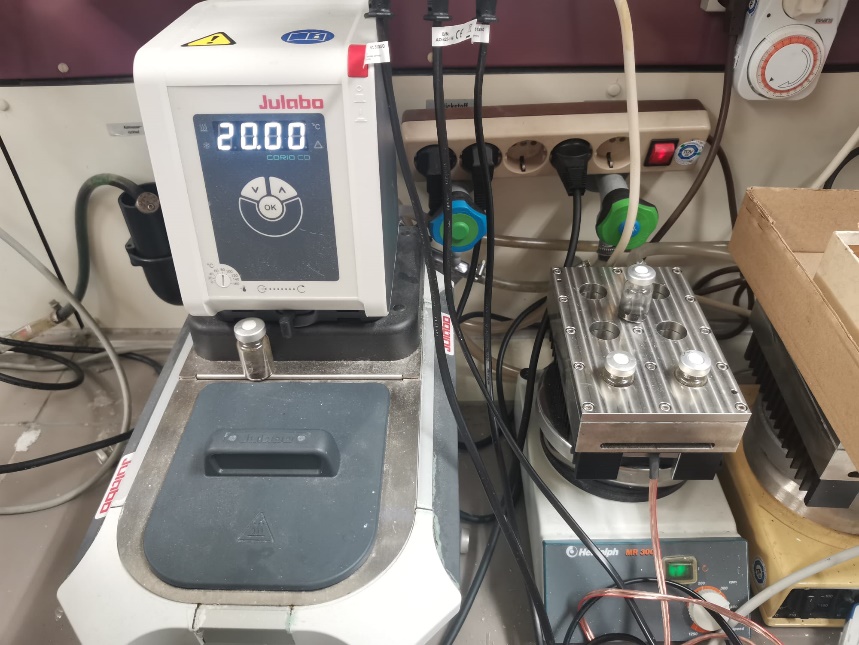

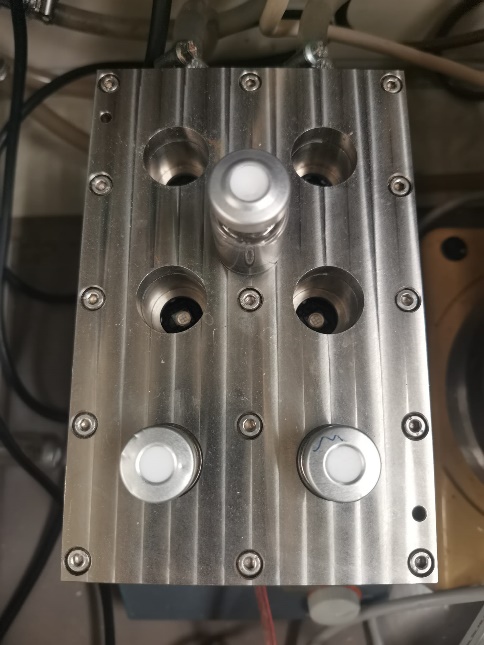

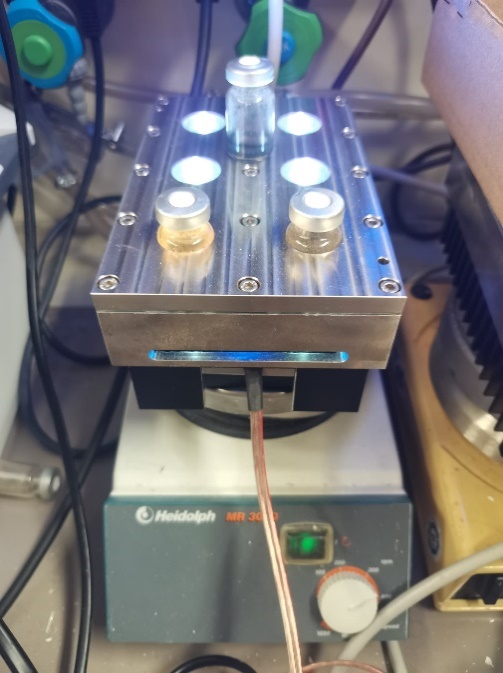


**Figure S1:** Pictures of the photosetup used for reactions run in 365 nm (1.5 W to 2.0 W optical power). General picture of the setup (left), picture from the top of the cooling block looking down to the LED plate (centre) and a sideway picture of the photosetup with lights on (right).

used to serve at any desired wavelength simply by changing the LED plate. **For safety**, goggles containing UV-and blue-light filters (“orange goggles”) should always be worn when working with the photoreactors. Empty vials (“dummy vials”) should be placed to fill each reaction spot, and the top of the setups should be covered during the reaction to prevent exposure to diffracted light.

### 1.1.2 385 nm (0.5 W) setup

For the photoreactions run at 385 nm (0.5 W) were performed using custom-made photoreactors designed to allow for an increased number of simultaneously run reactions (Figure S2). At the core of the photoreactors reside a stainless-steel block to which fifteen single-spot LEDs with emission centered around 385 nm are mounted. On top of the LED block sits a hollow cooling block made of stainless-steel, through which a constant flow of water is possible. This cooling block also contains fifteen holes made to perfectly fit 5 mL crimp-cap vials, thus providing a sideway contact of the reaction vessels to the cooling system. Magnetic stirring for the reactions is imposed from underneath the LED blocks using Cimarec i Poly 15-point stirring plates. Finally, the water cooling is sustained with a Julabo Curio Co water cooling unit. Due to the modular principle of the reactors, these several wavelengths can be utilized on these setups by changing the LED plate. **For safety**, goggles containing UV-and blue-light filters (“orange goggles”) should always be worn when working with the photoreactors. Empty vials (“dummy vials”) should be placed to fill each reaction spot, and the top of the setups should be covered during the reaction to prevent exposure to diffracted light.


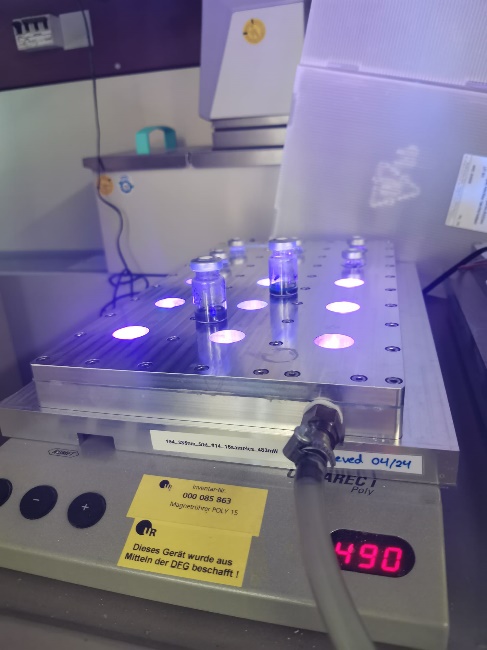

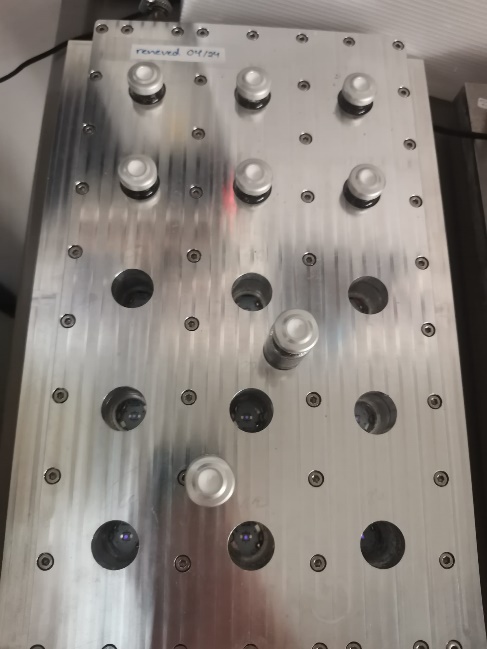

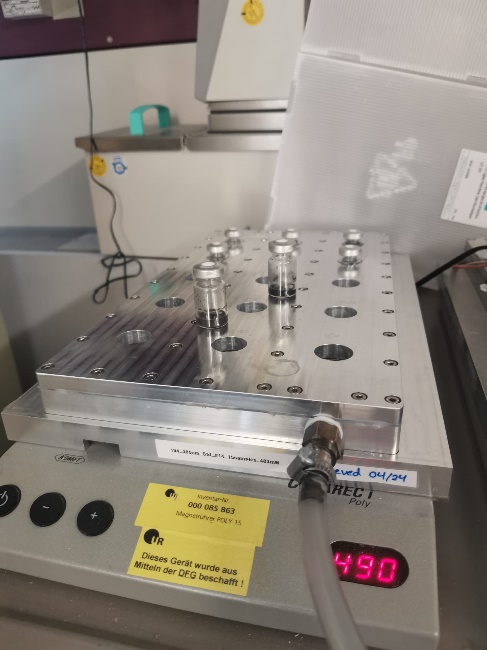


**Figure S2:** Pictures of the photosetup used for reactions run in 385 nm (0.5 W optical power). General picture of the setup (left), picture from the top of the cooling block looking down to the LED plate (centre) and a sideway picture of the photosetup with lights on (right).

### 1.1.3 Emission spectrum of the LEDs

The emission spectrum of the utilized LEDs (365 nm, Inolus and 385 nm, New Energy Luminus) was also measured to better characterize the actual emitted wavelengths of each setup. The measurement was carried out with Ocean Optics HR 2000+ spectrometer. The results are plotted in Figure S3.

**Figure S3:** Emission spectra of the 365 nm LEDs (left) and 385 nm LEDs (right)

As can be easily observed from Figure S3, both of the utilized LEDs have their emission maxima around the desired wavelength, yet tailing is present for both LEDs. For the 365 nm LED, the actual emission starts around 350 nm and tails closer to 400 nm. For the 385 nm LEDs, on the other hand, the tailing is broader to both directions thus covering the area from around 360 nm to 440 nm.

### 1.1.4 Reaction mixtures before and after irradiation

The success of the decarboxylative oxidative coupling can be qualitatively assessed by the look of the reaction after the irradiation period. Before irradiation, the reaction mixture is (in most cases depending on the carboxylic acid and sulfonamide reactants) a white milky solution mostly due to the incomplete solubility of Bi(OTf)_3_ (Figure S4, left). After a successful reaction, the formation of fully reduced bismuth (elemental bismuth, “bismuth black”) gives the mixture a very dark, often black color (Figure S4, right).


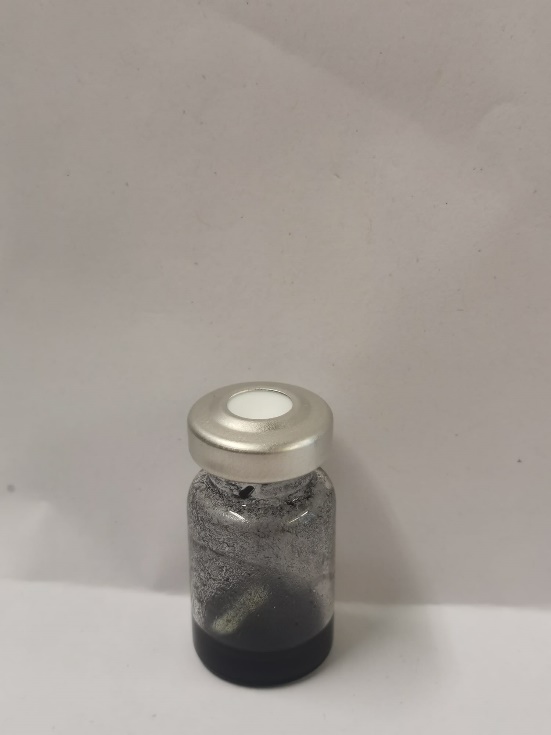

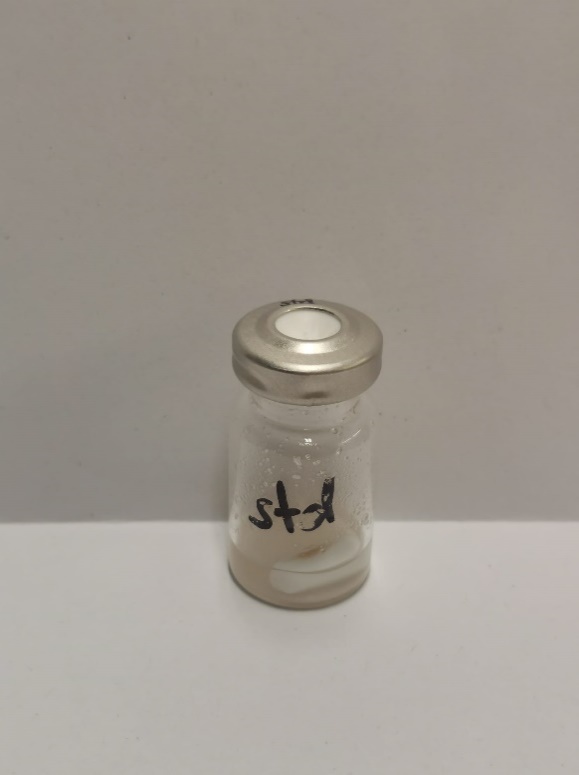


**Figure S4:** Pictures of the reaction mixture in a 5 mL crimp-cap vial before the reaction (left) and after irradiation (right).

## 1.2 GC-FID Calibration

For the calculation of the exact yields during the reaction optimization, a five-point calibration was carried out for the GC-FID. For this, a stock solution of the product **3a** in DCM was prepared, five samples were taken followed by dilution to the desired concentrations (see molarities below). Equal amounts of mesitylene were then added as internal standard, and the samples were submitted for GC-FID.

|  | **Figure S5:** Calibration curve plotted with the ratio of product peak area to the area of internal standard as the y-axis and molar amount of the product given in each sample as x-axis as obtained from the GC-FID. Linear fit across the points and zero-point intersection added to obtain the equation for the line. |  |
| --- | --- | --- |

After the GC-FID analysis, the obtained areas of the peaks corresponding to the product and internal standard were then collected and plotted against the amounts of the product in moles. The linear fit was then applied, and the obtained curve set to intersect the origin. The equation of this line (y = 1,0917x) was utilized to calculate all GC-FID yields measured afterwards. Finally, the correlation coefficient (R^2^) was analyzed to verify the accuracy of the calibration curve.

# 2. Extended optimization studies

## 2.1 General procedure for the optimization studies

4-Methylbenzenesulfonamide (*n* equiv.) and base (*m* equiv.) and were weighed into a 5 mL crimp-cap vial equipped with a stirring bar. The vial was sealed and set under an inert atmosphere by evacuating and back-filling with N_2_ three times. A 0.1 M stock solution of ($\pm$)-2-phenylpropanoic acid in a pre-dried solvent was prepared under N_2_ and 1 mL of this solution was added to the reaction mixture. Residual oxygen was then removed by freeze-pump thaw ($\times$3), after which the reaction was irradiated with 365 nm LEDs (2 W) while maintaining the temperature at 25 °C with a water-cooling block. After the given reaction time, a 100 μL of stock solution (1.0 M mesitylene in DCM) was added as internal standard and the reaction mixtures were filtered through a syringe filter. The reactions were then analyzed with GC-FID using helium as carrier gas and the final yields were calculated against a five-point calibration curve of product **3a** and mesitylene.

## 2.2 Solvent screening

**Table S1:** Full optimization of solvent.

| **Entry** | **Solvent** | **Yield** (%) |
| --- | --- | --- |
| 1 | DCM | 28 |
| 2 | DCE | 11 |
| 3 | CHCl_3_ | 9 |
| 4 | MeCN | *traces* |
| 5 | TFE | n.d. |
| 6 | HFIP | n.d. |
| 7 | Toluene | n.d. |
| 8 | H_2_O | *traces* |
| 9 | DMSO | n.d. |
| 10 | DMA | n.d. |
| 11 | THF | n.d. |
| 12 | EtOAc | *traces* |
| 13 | Acetone | *traces* |

|  | Reaction conditions: Bi(OTf)_3_ (130 mg, 0.20 mmol, 2.0 equiv.), 2-phenylpropanoic acid (30 mg, 0.20 mmol, 2.0 equiv.), NaHCO_3_ (8.2 mg, 0.10 mmol, 1.0 equiv.) and 4-methylphenylsulfonamide (17.2 mg, 0.10 mmol, 1.0 equiv.) were dissolved into solvent in case (1 mL) under N_2_. After irradiation with 365 nm (18 h) at 25 °C internal standard was added and reactions analyzed with GC-FID. Abbreviations used: DCM=dichloromethane, DCE=dichloroethane, CHCl_3_=chloroform, MeCN=acetonitrile, TFE=trifluoroethanol, HFIP=hexafluoroisopropanol, DMSO=dimethylsulfoxide, DMA=dimethylacetamide, THF=tetrahydrofuran, EtOAc=ethyl acetate. |  |
| --- | --- | --- |

## 2.3 Bismuth source screening

**Table S2:** Full optimization of the bismuth source.

| **Entry** | **Catalyst screening** | **Yield** (%) |
| --- | --- | --- |
| 1 | Bi(OTf)_3_ | 28 |
| 2 | BiCl_3_ | *traces* |
| 3 | BiF_3_ | n.d. |
| 4 | BiBr_3_ | n.d. |
| 5 | BiPh_3_ | 10 |
| 6 | Bi^(0)^ | n.d. |
| 7 | Bi_2_O_3_ | n.d. |
| 8 | BiOCl | n.d. |
| 9 | Bi(OAc)_3_ | n.d. |
| 10 | Bi(NO_3_)_3_ | n.d. |

|  | Reaction conditions: the bismuth source (0.20 mmol, 2.0 equiv.), 2-phenylpropanoic acid (30 mg, 0.20 mmol, 2.0 equiv.), NaHCO_3_ (8.2 mg, 0.10 mmol, 1.0 equiv.) and 4-methylphenylsulfonamide (17.2 mg, 0.10 mmol, 1.0 equiv.) were dissolved into DCM (1 mL) under N_2_. After irradiation with 365 nm (18 h) at 25 °C, internal standard was added and reactions analyzed with GC-FID. |  |
| --- | --- | --- |

## 2.4 Base screening

**Table S3:** Full optimization of bases.

| **Entry** | **Base** | **Yield** (%) |
| --- | --- | --- |
| 1 | none | *traces* |
| 2 | NaHCO_3_ | 28 |
| 3 | KHCO_3_ | 37 |
| 4 | K_2_CO_3_ | 14 |
| 5 | K_3_PO_4_ | 14 |
| 6 | K_2_HPO_4_ | 27 |
| 7 | Na_2_HPO_4_ | 7 |
| 8 | KO*t*Bu | 30 |
| 9 | DBU | *traces* |
| 10 | DABCO | 30 |
| 11 | 2,6-Lutidine | 5 |
| 12 | TBD | *traces* |
| 13 | TMG | *traces* |
| 14 | Tetramethylpiperidine | *traces* |

|  | Reaction conditions: Bi(OTf)_3_ (130 mg, 0.20 mmol, 2.0 equiv.), 2-phenylpropanoic acid (30 mg, 0.20 mmol, 2.0 equiv.), the base in question (0.10 mmol, 1.0 equiv.) and 4-methylphenylsulfonamide (17.2 mg, 0.10 mmol, 1.0 equiv.) were dissolved into DCM (1 mL) under N_2_. After irradiation with 365 nm (18 h) at 25 °C, internal standard was added and reactions analyzed with GC-FID. Abbreviations used: DBU=1,8-diazabicyclo[5.4.0]undec-7-ene, DABCO=1,4-diazabicyclo[2.2.2]octane, TBD=1,5,7-triazabicyclo[4.4.0]dec-5-en, TMG=tetramethylguanidine. |  |
| --- | --- | --- |

## 2.5 Stoichiometry screening

**Table S4:** Full screening of the reaction stoichiometry.

| **Entry** | **1a** (equiv.) | **2a** (equiv.) | **Base** (equiv.) | **Yield** (%) |
| --- | --- | --- | --- | --- |
| 1 | 1.0 | 1.0 | 1.0 | 38 |
| 2 | 2.0 | 1.0 | 0.5 | 27 |
| 3 | 2.0 | 1.0 | 1.5 | 38 |
| 4 | 2.0 | 1.0 | 2.0 | 42 |
| 5 | 2.0 | 1.0 | 2.5 | 22 |
| 6 | 3.0 | 1.0 | 1.0 | 45 |
| 7 | 3.0 | 1.0 | 2.0 | 60 |
| 8 | 3.0 | 1.0 | 3.0 | 46 |
| 9 | 4.0 | 1.0 | 3.0 | 16 |
| 10 | 1.0 | 2.0 | 2.0 | 16 |
| 11 | 1.0 | 2.0 | 3.0 | 48 |
| 12 | 1.0 | 2.0 | 1.0 | 40 |
| 13 | 1.0 | 3.0 | 2.0 | 25 |
| 14 | 1.0 | 3.0 | 3.0 | 62 |
| 15 | 1.0 | 4.0 | 2.0 | 62 |
| 16 | 1.0 | 4.0 | 3.0 | 31 |
| 17 | 1.0 | 3.5 | 2.0 | 52 |
| 18 | 1.0 | 3.5 | 3.0 | 60 |

|  | Reaction conditions: Bi(OTf)_3_ (130 mg, 0.20 mmol, 2.0 equiv.), 2-phenylpropanoic acid (x equiv.), KHCO_3_ (y equiv.) and 4-methylphenylsulfonamide (z equiv.) were dissolved into DCM (1 mL) under N_2_. After irradiation with 365 nm (18 h) at 25 °C, internal standard was added and reactions analyzed with GC-FID. |  |
| --- | --- | --- |

## 2.6 Concentration screening

**Table S5:** Full screening of reaction concentration.

| **Entry** | **n(acid)** (mmol) | **V(DCM)** (mL) | **Concentration** (M) | **Yield** (%) |
| --- | --- | --- | --- | --- |
| 1 | 0.05 | 1 | 0.05 | 31 |
| 2 | 0.075 | 1 | 0.075 | 33 |
| 3 | 0.15 | 1 | 0.15 | 51 |
| 4 | 0.2 | 1 | 0.2 | 30 |
| 5 | 0.1 | 0.5 | 0.2 | 20 |
| 6 | 0.1 | 0.75 | 0.13 | 31 |
| 7 | 0.1 | 1.0 | 0.1 | 60 |
| 8 | 0.1 | 1.5 | 0.66 | 49 |
| 9 | 0.1 | 2.0 | 0.05 | 47 |

|  | Reaction conditions: Bi(OTf)_3_ (2.0 equiv.), 2-phenylpropanoic acid (x mmol, 1.0 equiv.), KHCO_3_ (3.0 equiv.) and 4-methylphenylsulfonamide (3.5 equiv.) were dissolved into DCM (m mL) under N_2_. After irradiation with 365 nm (18 h) at 25 °C, internal standard was added and reactions analyzed with GC-FID. |  |
| --- | --- | --- |

## 2.7 Irradiation source and temperature screening

**Table S6:** Full screening of irradiation wavelength and temperature.

| **Entry** | **LED** | **Temperature** (°C) | **Yield** (%) |
| --- | --- | --- | --- |
| 1 | 365 nm (3W) | 25 | 39 |
| 2 | 365 nm (0.8W) | 25 | 4 |
| 3 | 340 nm (0.8W) | 25 | 6 |
| 4 | 385 nm (0.8W) | 25 | 44 |
| 5 | 385 nm (3W) | 25 | 43 |
| 6 | 365 nm (2W) | 10 | 34 |
| 7 | 365 nm (2W) | 15 | 39 |
| 8 | 365 nm (2W) | 20 | 55 |
| 9 | 365 nm (2W) | 25 | 60 |
| 10 | 365 nm (2W) | 30 | 42 |

|  | Reaction conditions: Bi(OTf)_3_ (130 mg, 0.2 mmol, 2.0 equiv.), 2-phenylpropanoic acid (15.0 mg, 0.1 mmol, 1.0 equiv.), KHCO_3_ (30.0 mg, 0.3 mmol, 3.0 equiv.) and 4-methylphenylsulfonamide (65.5 mg, 0.35 mmol, 3.5 equiv.) were dissolved into DCM (1 mL) under N_2_. After irradiation with the wavelength in question for the given times at given temperatures, internal standard was added and reactions analyzed with GC-FID. |  |
| --- | --- | --- |

## 2.8 Bismuth loading screening

**Table S7:** Full optimization of bismuth triflate loading.

| **Entry** | **Bi Loading** | **Yield** (%) |
| --- | --- | --- |
| 1 | 1.0 equiv. | 22 |
| 2 | 1.5 equiv. | 44 |
| 3 | 1.6 equiv. | 44 |
| 4 | 1.7 equiv. | 60 |
| 5 | 2.0 equiv. | 60 |
| 6 | 2.5 equiv. | 52 |

|  | Reaction conditions: Bi(OTf)_3_ (n equiv.), 2-phenylpropanoic acid (15.0 mg, 0.1 mmol, 1.0 equiv.), KHCO_3_ (30.0 mg, 0.3 mmol, 3.0 equiv.) and 4-methylphenylsulfonamide (65.5 mg, 0.35 mmol, 3.5 equiv.) were dissolved into DCM (1 mL) under N_2_. After irradiation with 365 nm for 10 h at 25 °C, internal standard was added and reactions analyzed with GC-FID. |  |
| --- | --- | --- |

## 2.9 Oxidant screening

**Table S8:** Full optimization of the terminal oxidant.

| **Entry** | **Oxidant** | **Yield** (%) |
| --- | --- | --- |
| 1 | Bi(OTf)_3_ | 60 |
| 2 | none | 11 |
| 3 | Air | n.d. |
| 4 | Oxone | *traces* |
| 5 | Na_2_S_2_O_8_ | *traces* |
| 6 | K_2_S_2_O_8_ | *traces* |
| 7 | (NH_4_)_2_S_2_O_8_ | 5 |
| 8 | NFSI | 11 |
| 9 | PIDA | 14 |
| 10 | Benzoyl peroxide | *traces* |

|  | Reaction conditions: Bi(OTf)_3_ (13.0 mg, 0.02 mmol, 20 mol%), 2-phenylpropanoic acid (15.0 mg, 0.1 mmol, 1.0 equiv.), KHCO_3_ (30.0 mg, 0.3 mmol, 3.0 equiv.), 4-methylphenylsulfonamide (65.5 mg, 0.35 mmol, 3.5 equiv.) and the terminal oxidant (2.0 equiv.) were dissolved into DCM (1 mL) under N_2_. After irradiation with 365 nm for 18 h at 25 °C, internal standard was added and reactions analyzed with GC-FID. Abbreviations used: NFSI=*N*-fluorobenzenesulfonimide, PIDA=(diazetoxyiodo)benzene. |  |
| --- | --- | --- |

## 2.10 Control Experiments

**Table S9:** Control experiments.

| **Entry** | **Variation** | **Yield** (%) |
| --- | --- | --- |
| 1 | Under air | *traces* |
| 2^a)^ | BiBr_3_ + Na(OTf)_3_ | n.d. |
| 3 | No bismuth | n.d. |
| 4 | No light | n.d. |
| 5 | 60 °C dark | n.d. |

|  | ^a)^ BiBr_3_ (2 equiv.) + Na(OTf)_3_ (2 equiv) used instead of Bi(OTf)_3_.  Reaction conditions: Bi(OTf)_3_ (130 mg, 0.20 mmol, 0.2 equiv.), 2-phenylpropanoic acid (15.0 mg, 0.1 mmol, 1.0 equiv.), KHCO_3_ (30.0 mg, 0.3 mmol, 3.0 equiv.) and 4-methylphenylsulfonamide (65.5 mg, 0.35 mmol, 3.5 equiv.) were dissolved into DCM (1 mL) under N_2_. After irradiation with 365 nm for 10 h at 25 °C, internal standard was added and reactions analyzed with GC-FID. |  |
| --- | --- | --- |

# 3. Synthesis and characterization of products

## 3.1 General procedures for the photocatalyzed nucleophilic couplings

### 3.1.1 Carboxylic acid as a limiting reagent (GP1) [see details for each substrate below]

Four identical reactions were set up as following: Sulfonamide (0.35 mmol, 3.50 equiv.), KHCO_3_ (30.0 mg, 0.30 mmol, 3.00 equiv.), Bi(OTf)_3_ (130 mg, 0.20 mmol, 2.00 equiv.) and, if solid, the corresponding carboxylic acid (0.10 mmol, 1.00 equiv.) were weighed into a 5 mL crimp-cap vial equipped with a stirring bar. The vial was sealed and set under an inert atmosphere by evacuating and back-filling with N_2_ ($\times$3). If liquid, a stock solution of the corresponding carboxylic acid was prepared in dry DCM (0.1 M), and either 1 mL of this solution or 1 mL of dry DCM was added *via* syringe to the reaction mixture. The reaction mixture was degassed three times with a freeze-pump thaw, after which the reaction was irradiated either with 365 nm (1.8 W) for 10 hours or with 385 nm (0.5 W) for 16 hours while maintaining the temperature at 25 °C with a water-cooling block.

After completion, the reactions were combined, and crude reaction mixture was absorbed to silica. The reaction solvent was then removed under reduces pressure. Purification by flash column chromatography on silica (typically Acetone/PE 20% → 40% or EtOAc/PE 20% → 40%) followed by solvent evaporation yielded the desired products.

### 3.1.1 Sulfonamide as a limiting reagent (GP2) [see details for each substrate below]

Four identical reactions were set up as following: Sulfonamide (0.10 mmol, 1.00 equiv.), KHCO_3_ (30.0 mg, 0.30 mmol, 3.00 equiv.), Bi(OTf)_3_ (130 mg, 0.20 mmol, 2.00 equiv.) and carboxylic acid (0.30 mmol, 3.00 equiv.) were weighed into a 5 mL crimp-cap vial equipped with a stirring bar. The vial was sealed and set under an inert atmosphere by evacuating and back-filling with N_2_ ($\times$3). If liquid, a stock solution of the corresponding carboxylic acid was prepared in dry DCM (0.1 M), and either 1 mL of this solution or 1 mL of dry DCM was added *via* syringe to the reaction mixture. The reaction mixture was degassed three times with a freeze-pump thaw, after which the reaction was irradiated either with 365 nm (1.8 W) for 10 hours or with 385 nm (0.5 W) for 16 hours while maintaining the temperature at 25 °C with a water-cooling block.

After completion, the reactions were combined, and crude reaction mixture was absorbed to silica. The reaction solvent was then removed under reduces pressure. Purification by flash column chromatography on silica (typically Acetone/PE 20% → 40% or EtOAc/PE 20% → 40%) followed by solvent evaporation yielded the desired products.

## 3.2 Scope of carboxylic acids

**Synthesis of** **4-methyl-*N*-(1-phenylethyl)benzenesulfonamide (3a)**

4-Methyl-*N*-(1-phenylethyl)benzenesulfonamide (**3a**) was synthetized according to **GP1** using ($\pm$)-2-phenylpropionic acid (in total 60.0 mg, 0.40 mmol, 1.0 equiv.) as the limiting reagent and irradiated at 365 nm for 10 h. After purification with reverse-phase flash column chromatography (H_2_O+0.5‰ TFA/MeCN, 10% → 100%) the title compound was obtained as a colorless oil which crystallized to white solid upon standing under ambient conditions (59 mg, 54%).

**R_f_** (silica, 50% EtOAc/PE): 0.87 [potassium permanganate];

**^1^H-NMR** (400 MHz, CDCl_3_): δ (ppm) 7.60–7.65 (m, 2H), 7.14–7.19 (m, 5H), 7.09–7.12 (m, 2H), 5.33 (d, *J* = 7.0 Hz, 1H), 4.45 (pent., *J* = 7.0 Hz, 1H), 2.37 (s, 3H), 1.41 (d, *J* = 7.0 Hz, 3H);

**^13^C-NMR** (101 MHz, CDCl_3_): δ (ppm) 143.1, 142.1, 137.7, 129.4, 128.5, 127.4, 127.1, 126.1, 53.7, 23.6, 21.5;

**HRMS** (ESI+): calc. [M+Na]^+^ for C_15_H_17_NO_2_SNa 298.0878, found 298.0874, Δ = 0.4 mDa.

**NOTE:** An improved chromatographic separation was observed upon changing the *p*-methyl group of the nucleophile to a *p*-methoxy group. Therefore, the nucleophile **2b** was further on used as the standard nucleophile.

**Synthesis of 4-methoxy-*N*-(1-phenylethyl)benzenesulfonamide (3b)**

4-Methoxy-*N*-(1-phenylethyl)benzenesulfonamide (**3b**) was synthetized according to **GP1** using ($\pm$)-2-phenylpropionic acid (in total 60.0 mg, 0.40 mmol, 1.0 equiv.) as the limiting reagent and 4-methoxybenzenesulfonamide (in total 262 mg, 1.40 mmol, 3.50 equiv.) as nucleophile in excess. The reaction was irradiated at 365 nm for 10 h. After purification with flash column chromatography (EtOAc/PE, 20% → 40%) the title compound was obtained as a colorless oil which slowly crystallized to a white solid upon standing under ambient conditions (59 mg, 51%).

**R_f_** (silica, 50% EtOAc/PE): 0.73 [potassium permanganate];

**^1^H-NMR** (400 MHz, CDCl_3_): δ (ppm) 7.637.67 (m, 2H), 7.17–7.22 (m, 3H), 7.08–7.11 (m, 2H), 6.82–6.86 (m, 2H), 4.90 (d, *J* = 6.7 Hz, 1H), 4.45 (pent., *J* = 6.7 Hz, 1H), 3.83 (s, 3H), 1.42 (d, *J* = 6.8 Hz, 3H);

**^13^C-NMR** (101 MHz, CDCl_3_): δ (ppm) 162.7, 142.0, 132.2, 129.2, 128.6, 127.5, 126.1, 114.0, 55.6, 53.6, 23.6;

**HRMS** (ESI+): calc. [M+Na]^+^ for C_15_H_17_NO_3_SNa 314.0827, found 314.0821, Δ = 0.6 mDa.

**Synthesis of 4-methoxy-*N*-(1-(*p*-tolyl)ethyl)benzenesulfonamide (3c)**

4-Methoxy-*N*-(1-(*p*-tolyl)ethyl)benzenesulfonamide (**3c**) was synthetized according to **GP1** using 2-(4-methylphenyl)propanoic acid (in total 65.6 mg, 0.40 mmol, 1.0 equiv.) as the limiting reagent and 4-methoxybenzenesulfonamide (in total 262 mg, 1.40 mmol, 3.50 equiv.) as nucleophile in excess. The reaction was irradiated at 385 nm for 16 h. After purification with flash column chromatography (Acetone/PE, 30%) the title compound was obtained as a pale solid (54 mg, 44%).

**R_f_** (silica, 50% EtOAc/PE): 0.80 [potassium permanganate];

**^1^H-NMR** (400 MHz, CDCl_3_): δ (ppm) 7.65–7.68 (m, 2H), 6.99 (s, 4H), 6.82–6.86 (m, 2H), 5.14 (br. s., 1H), 4.40 (pent., 6.2 Hz, 1H), 3.83 (s, 3H), 2.27 (s, 3H), 1.40 (d, *J* = 6.8 Hz, 3H);

**^13^C-NMR** (101 MHz, CDCl_3_): δ (ppm) 162.6, 139.2, 137.1, 132.3, 129.3, 129.2, 126.1, 113.9, 55.6, 53.4, 23.5, 21.0;

**HRMS** (ESI+): calc. [M+Na]^+^ for C_16_H_19_NO_3_SNa 328.0983, found 328.0982, Δ = 0.1 mDa.

**Synthesis of 4-methoxy-*N*-(1-(*m*-tolyl)ethyl)benzenesulfonamide (3d)**

4-Methoxy-*N*-(1-(*p*-tolyl)ethyl)benzenesulfonamide (**3d**) was synthetized according to **GP1** using 2-(4-methylphenyl)propanoic acid (in total 65.6 mg, 0.40 mmol, 1.0 equiv.) as the limiting reagent and 4-methoxybenzenesulfonamide (in total 262 mg, 1.40 mmol, 3.50 equiv.) as nucleophile in excess. The reaction was irradiated at 385 nm for 16 h. After purification with flash column chromatography (Acetone/PE, 30%) the title compound was obtained as a colorless oil (59 mg, 48%).

**R_f_** (silica, 50% EtOAc/PE): 0.77 [potassium permanganate];

**^1^H-NMR** (400 MHz, CDCl_3_): δ (ppm) 7.63–7.67 (m, 2H), 7.08 (t, *J* = 7.5 Hz, 1H), 6.90–6.97 (m, 2H), 6.84 (br. s., 1H), 6.80–6.84 (m, 2H), 5.33 (d, *J* = 6.8 Hz, 1H), 4.40 (pent., *J* = 6.8 Hz, 1H), 3.82 (s, 3H), 2.20 (s, 3H), 1.40 (d, *J* = 6.8 Hz, 3H);

**^13^C-NMR** (101 MHz, CDCl_3_): δ (ppm) 162.6, 142.0, 138.1, 132.4, 129.2, 128.4, 128.1, 126.9, 123.2, 113.9, 55.6, 53.7, 23.6, 21.3;

**HRMS** (ESI+): calc. [M+Na]^+^ for C_16_H_19_NO_3_SNa 328.0983, found 328.0978, Δ = 0.5 mDa.

**Synthesis of 4-methoxy-*N*-(1-(*o*-tolyl)ethyl)benzenesulfonamide (3e)**

4-Methoxy-*N*-(1-(*o*-tolyl)ethyl)benzenesulfonamide (**3e**) was synthetized according to **GP1** using 2-(4-methylphenyl)propanoic acid (in total 65.6 mg, 0.40 mmol, 1.0 equiv.) as the limiting reagent and 4-methoxybenzenesulfonamide (in total 262 mg, 1.40 mmol, 3.50 equiv.) as nucleophile in excess. The reaction was irradiated at 385 nm for 16 h. After purification with flash column chromatography (Acetone/PE, 30%) the title compound was obtained as a pale yellowish oil (70 mg, 57%).****

**R_f_** (silica, 50% EtOAc/PE): 0.78 [potassium permanganate];

**^1^H-NMR** (400 MHz, CDCl_3_): δ (ppm) 7.60–7.65 (m, 2H), 7.11–7.14 (m, 1H), 6.99–7.05 (m, 3H), 6.77–6.82 (m, 2H), 5.40 (d, *J* = 6.8 Hz, 1H), 4.72 (pent., *J* = 6.8 Hz, 1H), 3.81 (s, 3H), 2.21 (s, 3H), 1.37 (d, *J* = 6.8 Hz, 3H);

**^13^C-NMR** (101 MHz, CDCl_3_): δ (ppm) 162.6, 140.3, 134.4, 132.3, 130.4, 129.1, 127.1, 126.4, 125.4, 113.9, 55.6, 49.7, 23.1, 19.0;

**HRMS** (ESI+): calc. [M+Na]^+^ for C_16_H_19_NO_3_SNa 328.0983, found 328.0980, Δ = 0.3 mDa.

**Synthesis of** ***N*-(1-(4-(*tert*-butyl)phenyl)ethyl)-4-methoxybenzenesulfonamide (3f)**

*N*-(1-(4-(*tert*-butyl)phenyl)ethyl)-4-methoxybenzenesulfonamide (**3f**) was synthetized according to **GP1** using 2-(4-(*tert*-butyl)phenyl)propanoic acid (in total 82.4 mg, 0.40 mmol, 1.0 equiv.) as the limiting reagent and 4-methoxybenzenesulfonamide (in total 262 mg, 1.40 mmol, 3.50 equiv.) as nucleophile in excess. The reaction was irradiated at 385 nm for 16 h. After purification with flash column chromatography (Acetone/PE, 30%) the title compound was obtained as an off-white solid (78 mg, 56%).

**R_f_** (silica, 50% EtOAc/PE): 0.69 [potassium permanganate];

**^1^H-NMR** (400 MHz, CDCl_3_): δ (ppm) 7.52–7.57 (m, 2H), 7.07–7.10 (m, 2H), 6.91–6.95 (m, 2H), 6.68–6.73 (m, 2H), 5.30 (d, *J* = 6.9 Hz, 1H), 4.35 (pent., *J* = 6.9 Hz, 1H), 3.72 (s., 3H), 1.33 (d, *J* = 6.9 Hz, 3H), 1.17 (s, 9H);

**^13^C-NMR** (101 MHz, CDCl_3_): δ (ppm) 162.5, 150.2, 139.0, 132.4, 129.2, 125.9, 125.3, 113.8, 55.5, 53.4, 34.4, 31.3, 23.5;

**HRMS** (ESI+): calc. [M+Na]^+^ for C_19_H_25_NO_3_SNa 370.1453, found 370.1442, Δ = 1.1 mDa.

**Synthesis of *N*-(1-(4-fluorophenyl)ethyl)-4-methoxybenzenesulfonamide (3g)**

 *N*-(1-(4-Fluorophenyl)ethyl)-4-methoxybenzenesulfonamide **(3g)** was synthetized according to **GP1** using 2-(4-fluorophenyl)propanoic acid (in total 67.2 mg, 0.40 mmol, 1.0 equiv.) as the limiting reagent and 4-methoxybenzenesulfonamide (in total 262 mg, 1.40 mmol, 3.50 equiv.) as nucleophile in excess. The reaction was irradiated at 365 nm for 10 h. After purification with flash column chromatography (EtOAc/PE, 20% → 40%) the title compound was obtained as a pale orange oil (62 mg, 51%).

**R_f_** (silica, 50% EtOAc/PE): 0.75 [potassium permanganate];

**^1^H-NMR** (400 MHz, CDCl_3_): δ (ppm) 7.61–7.65 (m, 2H), 7.04–7.10 (m, 2H), 6.81–6.88 (m, 4H), 5.34 (br. s, 1H), 4.43 (q., 6.7 Hz, 1H), 3.83 (s, 3H), 1.38 (d, *J* = 6.7 Hz, 3H);

**^13^C-NMR** (101 MHz, CDCl_3_): δ (ppm) 162.7, 161.9 (d, *J* = 246 Hz), 137.9, (d, *J* = 3.1 Hz), 132.1, 129.2, 127.9 (d, *J* = 8.2 Hz), 115.2 (d, *J* = 21.7 Hz), 114.0, 55.6, 53.0, 23.6;

**^19^F-NMR** (376 MHz, CDCl_3_, PhCF_3_ as internal standard): δ (ppm) –116.0 – (–)116.1 (m)

**HRMS** (ESI+): calc. [M+Na]^+^ for C_15_H_16_FNO_3_SNa 332.0733, found 332.0727, Δ = 0.6 mDa.

**Synthesis of** ***N*-(1-(4-chlorophenyl)ethyl)-4-methoxybenzenesulfonamide (3h)**

*N*-(1-(4-Chlorophenyl)ethyl)-4-methoxybenzenesulfonamide **(3h)** was synthetized according to **GP1** using 2-(4-chlorophenyl)propanoic acid (in total 73.8 mg, 0.40 mmol, 1.0 equiv.) as the limiting reagent and 4-methoxybenzenesulfonamide (in total 262 mg, 1.40 mmol, 3.50 equiv.) as nucleophile in excess. The reaction was irradiated at 365 nm for 10 h. After purification with flash column chromatography (Acetone/PE, 30% → 40%) the title compound was obtained as a white solid (67 mg, 51%).

**R_f_** (silica, 50% EtOAc/PE): 0.78 [potassium permanganate];

**^1^H-NMR** (400 MHz, CDCl_3_): δ (ppm) 7.59–7.63 (m, 2H), 7.09–7.13 (m, 2H), 7.01–7.04 (m, 2H), 6.79–6.84 (m, 2H), 5.38–5.50 (m, 1H), 4.42 (pent., *J* = 6.9 Hz, 1H), 3.83 (s, 3H), 1.37 (d, *J* = 6.9 Hz, 3H);

**^13^C-NMR** (101 MHz, CDCl_3_): δ (ppm) 162.7, 140.77, 133.0, 132.0, 129.2, 128.5, 127.7, 124.1, 114.0, 55.6, 53.0, 23.5;

**HRMS** (ESI+): calc. [M+Na]^+^ for C_15_H_16_ClNO_3_SNa 348.0437, found 348.0428, Δ = 0.9 mDa.

**Synthesis of *N*-(1-(4-trifluoromethoxyphenyl)ethyl)-4-methoxybenzenesulfonamide (3i)**

*N*-(1-(4-Trifluoromethoxyphenyl)ethyl)-4-methoxybenzenesulfonamide (**3i**) was synthetized according to **GP1** using 2-(4-(trifluoromethyoxy)phenyl)propanoic acid (in total 72.9 mg, 0.30 mmol, 1.0 equiv.) as the limiting reagent and 4-methoxybenzenesulfonamide (in total 262 mg, 1.40 mmol, 3.50 equiv.) as nucleophile in excess. The reaction was irradiated at 365 nm for 10 h. After purification with flash column chromatography (Acetone/PE, 30% → 40%) the title compound was obtained as a pale yellow solid (54 mg, 50%).

**R_f_** (silica, 50% EtOAc/PE): 0.81 [potassium permanganate];

**^1^H-NMR** (400 MHz, CDCl_3_): δ (ppm) 7.58–7.62 (m, 2H), 7.11–7.15 (m, 2H), 6.97–7.02 (m, 2H), 6.77–6.82 (m, 2H), 5.48 (d, *J* = 7.0 Hz, 1H), 4.48 (pent., *J* = 7.0 Hz, 1H), 3.81 (s, 3H), 1.39 (d, *J* = 7.0 Hz, 3H);

**^13^C-NMR** (101 MHz, CDCl_3_): δ (ppm) 162.8, 148.2, 140.9, 132.0, 129.1, 127.7, 124.2, 121.7, 120.9, 120.3 (q, *J* = 257 Hz), 119.0, 116.6, 55.5, 53.0, 23.6;

**^19^F-NMR** (376 MHz, CDCl_3_, PhCF_3_ as internal standard): δ (ppm) – 58.97 (s)

**HRMS** (ESI+): calc. [M+Na]^+^ for C_16_H_16_F_3_NO_3_SNa 398.0650, found 398.0650, Δ = 0.0 mDa.

**Synthesis of *N*-(1-(4-bromophenyl)propyl)-4-methoxybenzenesulfonamide (3j)**

*N*-(1-(4-bromophenyl)propyl)-4-methoxybenzenesulfonamide (**3j**) was synthetized according to **GP1** using 2-(4-bromophenyl)butanoic acid (in total 97.2 mg, 0.40 mmol, 1.0 equiv.) as the limiting reagent and 4-methoxybenzenesulfonamide (in total 262 mg, 1.40 mmol, 3.50 equiv.) as nucleophile in excess. The reaction was irradiated at 365 nm for 10 h. After purification with flash column chromatography (EtOAc/PE, 20% → 40%) the title compound was obtained as a pale yellow solid (42 mg, 27%).

**R_f_** (silica, 50% EtOAc/PE): 0.85 [potassium permanganate];

**^1^H-NMR** (400 MHz, CDCl_3_): δ (ppm) 7.50–7.54 (m, 2H), 7.22–7.26 (m, 2H), 6.86–6.90 (m, 2H), 6.74–6.78 (m, 2H), 5.32 (d, *J* = 6.8 Hz, 1H), 4.14 (q., *J* = 7.1 Hz, 1H), 3.83 (s, 3H), 1.74 (sept., *J* = 7.1 Hz, 1H), 1.66 (sept. *J* = 7.2 Hz, 1H), 0.78 (t, *J* = 7.2 Hz, 3H);

**^13^C-NMR** (101 MHz, CDCl_3_): δ (ppm) 162.7, 139.8, 132.1, 131.4, 129.1, 128.5, 121.0, 113.8, 59.3, 55.6, 30.4, 10.4;

**HRMS** (ESI+): calc. [M+Na]^+^ for C_16_H_18_BrNO_3_SNa 406.0089, found 406.0085, Δ = 0.4 mDa.

**Synthesis of *N*-bezhydryl-4-methoxybenzenesulfonamide (3k)**

*N*-Bezhydryl-4-methylbenzenesulfonamide (**3k**) was synthetized according to **GP1** using diphenyl acetic acid (in total 84.8 mg, 0.40 mmol, 1.0 equiv.) as the limiting reagent and 4-methoxybenzenesulfonamide (in total 262 mg, 1.40 mmol, 3.50 equiv.) as nucleophile in excess. The reaction was irradiated at 385 nm for 16 h. After purification with flash column chromatography (Acetone/PE, 25%) the title compound was obtained as an off-white solid (82 mg, 58%).

**R_f_** (silica, 50% EtOAc/PE): 0.77 [potassium permanganate];

**^1^H-NMR** (400 MHz, CDCl_3_): δ (ppm) 7.58–7.62 (m, 2H), 7.18–7.24 (m, 6H), 7.10–7.13 (m, 4H), 6.77–6.80 (m, 2H), 5.56 (d, *J* = 7.2 Hz, 1H), 5.32 (d, *J* = 7.2 Hz, 1H), 3.82 (s, 3H);

**^13^C-NMR** (101 MHz, CDCl_3_): δ (ppm) 162.7, 140.6, 132.0, 129.3, 128.6, 127.6, 127.4, 113.9, 61.4, 55.6;

**HRMS** (ESI+): calc. [M+Na]^+^ for C_20_H_19_NO_3_SNa 376.0983, found 376.0981, Δ = 0.2 mDa.

**Synthesis of** **4-methoxy-*N*-(1-phenylpropyl)benzenesulfonamide (3l)**

4-Methoxy-*N*-(1-phenylpropyl)benzenesulfonamide (**3l**) was synthetized according to **GP1** using 2-phenylbutanoic acid (in total 65.6 mg, 0.40 mmol, 1.0 equiv.) as the limiting reagent and 4-methoxybenzenesulfonamide (in total 262 mg, 1.40 mmol, 3.50 equiv.) as nucleophile in excess. The reaction was irradiated at 365 nm for 10 h. After purification with flash column chromatography (EtOAc/PE, 20% → 40%) the title compound was obtained as a white solid (74 mg, 61%).

**R_f_** (silica, 50% EtOAc/PE): 0.81 [potassium permanganate];

**^1^H-NMR** (400 MHz, CDCl_3_): δ (ppm) 7.56–7.60 (m, 2H), 7.12–7.17 (m, 3H), 7.00–7.03 (m, 2H), 6.74–6.78 (m, 2H), 5.25–5.34 (m, 1H), 4.13–4.20 (m, 1H), 3.80 (s, 3H), 1.80 (sept., *J* = 7.2 Hz, 1H), 1.70 (sept., *J* = 7.2 Hz, 1H), 0.78 (t, *J* = 7.2 Hz, 3H);

**^13^C-NMR** (101 MHz, CDCl_3_): δ (ppm) 162.5, 140.8, 132.4, 129.1, 128.7, 127.3, 126.6, 113.8, 59.8, 55.6, 30.6, 10.5;

**HRMS** (ESI+): calc. [M+Na]^+^ for C_16_H_19_NO_3_SNa^+^ 328.0983, found 328.0978, Δ = 0.5 mDa.

**Synthesis of 4-methoxy-*N*-(1-phenylpentyl)benzenesulfonamide (3m)**

4-Methoxy-*N*-(1-phenylpentyl)benzenesulfonamide (**3m**) was synthetized according to **GP1** using 2-phenylhexanoic acid (in total 76.8 mg, 0.40 mmol, 1.0 equiv.) as the limiting reagent and 4-methoxybenzenesulfonamide (in total 262 mg, 1.40 mmol, 3.50 equiv.) as nucleophile in excess. The reaction was irradiated at 365 nm for 10 h. After purification with flash column chromatography (Acetone/PE, 20% → 30%) the title compound was obtained as a white solid (67 mg, 50%).

**R_f_** (silica, 50% EtOAc/PE): 0.86 [potassium permanganate];

**^1^H-NMR** (400 MHz, CDCl_3_): δ (ppm) 7.54–7.58 (m, 2H), 7.12–7.16 (m, 3H), 6.99–7.02 (m, 2H), 6.73–6.78 (m, 2H), 5.06–5.19 (m, 1H), 4.24 (q, *J* = 7.2 Hz, 1H), 3.80 (s, 3H), 1.70–1.80 (m, 1H), 1.61–1.70 (m, 1H), 1.17–1.27 (m, 3H), 1.04–1.13 (m, 1H), 0.80 (t, *J* = 7.2 Hz, 3H);

**^13^C-NMR** (101 MHz, CDCl_3_): δ (ppm) 162.5, 141.1, 132.4, 129.1, 128.4, 127.2, 126.5, 113.8, 58.3, 55.6, 37.4, 28.0, 22.2, 13.9;

**HRMS** (ESI+): calc. [M+Na]^+^ for C_18_H_23_NO_3_SNa^+^ 356.1296, found 356.1290, Δ = 0.6 mDa.

**Synthesis of** ***N*-(1,2-diphenylethyl)-4-methoxybenzenesulfonamide (3n)**

*N*-(1,2-Diphenylethyl)-4-methoxybenzenesulfonamide (**3n**) was synthetized according to **GP1** using 2-phenylhexanoic acid (in total 90.4 mg, 0.40 mmol, 1.0 equiv.) as the limiting reagent and 4-methoxybenzenesulfonamide (in total 262 mg, 1.40 mmol, 3.50 equiv.) as nucleophile in excess. The reaction was irradiated at 365 nm for 10 h. After purification with flash column chromatography (Acetone/PE, 20% → 30%) the title compound was obtained as a white solid (84 mg, 57%).

**R_f_** (silica, 50% EtOAc/PE): 0.86 [potassium permanganate];

**^1^H-NMR** (400 MHz, CDCl_3_): δ (ppm) 7.46–7.50 (m, 2H), 7.15–7.18 (m, 6H), 7.03–7.06 (m, 2H), 6.91–6.95 (m, 2H), 6.71–6.75 (m, 2H), 5.12–5.19 (m, 1H), 4.50 (q., *J* = 6.8 Hz, 1H), 3.81 (s, 3H), 2.96–3.00 (m, 2H);

**^13^C-NMR** (101 MHz, CDCl_3_): δ (ppm) 162.5, 140.5, 136.5, 131.8, 129.3, 129.1, 128.5, 128.3, 127.4, 126.8, 126.7, 113.4, 59.3, 55.6, 44.1;

**HRMS** (ESI+): calc. [M+Na]^+^ for C_21_H_21_NO_3_SNa 390.1140, found 390.1132, Δ = 0.8 mDa.

**Synthesis of** ***N*-(2-cyclohexyl-1-phenylethyl)-4-methoxybenzenesulfonamide (3o)**

*N*-(2-cyclohexyl-1-phenylethyl)-4-methoxybenzenesulfonamide (**3o**) was synthetized according to **GP1** using 3-cyclohexyl-2-phenylpropanoic acid (in total 92.9 mg, 0.40 mmol, 1.0 equiv.) as the limiting reagent and 4-methoxybenzenesulfonamide (in total 262 mg, 1.40 mmol, 3.50 equiv.) as nucleophile in excess. The reaction was irradiated at 365 nm for 10 h. After purification with flash column chromatography (Acetone/PE, 30%) the title compound was obtained as a pale yellowish oil (33 mg, 22%).

**R_f_** (silica, 50% EtOAc/PE): 0.74 [potassium permanganate];

**^1^H-NMR** (400 MHz, CDCl_3_): δ (ppm) 7.54–7.58 (m, 2H), 7.11–7.16 (m, 3H), 7.00–7.03 (m, 2H), 6.74–6.78 (m, 2H), 5.04 (d, *J* = 7.5 Hz, 1H), 4.35 (q., *J* = 7.5 Hz, 1H), 3.80 (s, 3H), 1.54–1.67 (m, 6H), 1.51 (sept., *J* = 7.1 Hz, 1H), 1.02–1.19 (m, 4H), 0.75–0.92 (m, 2H);

**^13^C-NMR** (101 MHz, CDCl_3_): δ (ppm) 162.5, 141.6, 132.4, 129.1, 128.4, 127.2, 126.4, 113.8, 55.7, 55.6, 45.7, 33.9, 33.2, 32.8, 26.4, 26.1, 26.0;

**HRMS** (ESI+): calc. [M+Na]^+^ for C_21_H_27_NO_3_SNa^+^ 396.1609, found 396.1608, Δ = 0.1 mDa.

**Synthesis of *N*-cyclohexyl(phenyl)methyl-4-methoxybenzenesulfonamide (3p)**

*N*-Cyclohexyl(phenyl)methyl-4-methoxybenzenesulfonamide (**3p**) was synthetized according to **GP1** using 2-cyclohexyl-2-phenylacetic acid (in total 87.2 mg, 0.40 mmol, 1.0 equiv.) as the limiting reagent and 4-methoxybenzenesulfonamide (in total 262 mg, 1.40 mmol, 3.50 equiv.) as nucleophile in excess. The reaction was irradiated at 365 nm for 10 h. After purification with flash column chromatography (Acetone/PE, 20% → 30%) the title compound was obtained as a white solid (75 mg, 52 %).

**R_f_** (silica, 50% EtOAc/PE): 0.83 [potassium permanganate];

**^1^H-NMR** (400 MHz, CDCl_3_): δ (ppm) 7.49–7.53 (m, 2H), 7.07–7.11 (m, 3H), 6.89–6.93 (m, 2H), 6.67–6.72 (m, 2H), 5.28 (d, *J* = 8.3 Hz, 1H), 3.78 (s, 3H), 1.93–1.98 (m, 1H), 1.69–1.76 (m, 2H), 1.53–1.63 (m, 3H), 1.24–1.28 (m, 1H), 1.03–1.11 (m, 2H), 0.82–0.96 (m, 2H);

**^13^C-NMR** (101 MHz, CDCl_3_): δ (ppm) 162.4, 140.0, 132.4, 129.1, 128.1, 127.0, 126.9, 113.7, 63.5, 55.5, 43.8, 29.8, 29.5, 26.2, 25.9 (2C);

**HRMS** (ESI+): calc. [M+Na]^+^ for C_20_H_25_NO_3_SNa 382.1453, found 382.1451, Δ = 0.2 mDa.

**Synthesis of 4-methoxy-*N*-(1-napthalen-1-yl)ethyl)benzenesulfonamide (3q)**

4-Methoxy-*N*-(1-napthalen-1-yl)ethyl)benzenesulfonamide (**3q**) was synthetized according to **GP1** using 2-(naphthalen-1-yl)propanoic acid (in total 60.0 mg, 0.30 mmol, 1.0 equiv.) as the limiting reagent and 4-methoxybenzenesulfonamide (in total 262 mg, 1.40 mmol, 3.50 equiv.) as nucleophile in excess. The reaction was irradiated at 385 nm for 16 h. After purification with flash column chromatography (Acetone/PE, 30%) the title compound was obtained as a white solid (27 mg, 26%).

**R_f_** (silica, 50% EtOAc/PE): 0.68 [potassium permanganate];

**^1^H-NMR** (400 MHz, CDCl_3_): δ (ppm) 7.89–7.93 (m, 1H), 7.78–7.81 (m, 1H), 7.66–7.70 (m, 1H), 7.57–7.61 (m, 2H), 7.43–7.47 (m, 2H), 7.35–7.38 (m, 1H), 7.28–7.33 (m, 1H), 6.67–6.72 (m, 2H), 5.28 (pent., *J* = 6.8 Hz, 1H), 5.14–5.20 (m, 1H), 3.78 (s, 3H), 1.59 (d, *J* = 6.8 Hz, 3H);

**^13^C-NMR** (101 MHz, CDCl_3_): δ (ppm) 162.6, 137.7, 133.8, 132.0, 130.2, 129.2, 128.8, 128.1, 126.3, 125.6, 125.3, 123.4, 122.6, 113.8, 55.5, 49.8, 23.3;

**HRMS** (ESI+): calc. [M+Na]^+^ for C_19_H_19_NO_3_SNa 364.0983, found 364.0980, Δ = 0.3 mDa.

**Synthesis of *N*-(1-(4-isobutylphenyl)ethyl)-4-methoxybenzenesulfonamide (3r)**

*N*-(1-(4-Isobutylphenyl)ethyl)-4-methylbenzenesulfonamide (**3r**) was synthetized according to **GP1** using 2-(4-isobutylphenyl)propanoic acid (rac. ibuprofen, in total 82.4 mg, 0.40 mmol, 1.0 equiv.) as the limiting reagent and 4-methoxybenzenesulfonamide (in total 262 mg, 1.40 mmol, 3.50 equiv.) as nucleophile in excess. The reaction was irradiated at 385 nm for 16 h. After purification with flash column chromatography (EtOAc/PE, 5% → 50%) the title compound was obtained as a white solid (87 mg, 63%).

**R_f_** (silica, 50% EtOAc/PE): 0.84 [potassium permanganate];

**^1^H-NMR** (400 MHz, CDCl_3_): δ (ppm) 7.62–7.67 (m, 2H), 6.95–7.01 (m, 4H), 6.81–6.85 (m, 2H), 4.92 (br. s., 1H), 4.39–4.46 (m, 1H), 3.82 (s, 3H), 2.39 (d, *J* = 6.9 Hz, 2H), 1.79 (sept., *J* = 6.9 Hz, 1H), 1.42 (d, *J* = 6.8 Hz, 3H), 0.86 (d, *J* = 6.9 Hz, 6H);

**^13^C-NMR** (101 MHz, CDCl_3_): δ (ppm) 162.6, 141.1, 139.3, 132.4, 129.2 (2C), 125.9, 113.9, 55.5, 53.4, 45.0, 30.2, 23.6, 22.3;

**HRMS** (ESI+): calc. [M+Na]^+^ for C_19_H_25_NO_3_SNa 370.1453, found 370.1453, Δ = 0.0 mDa.

**Synthesis of *N*-(1-(2-fluoro-[1,1’biphenyl]-4-yl)ethyl)-4-methylbenzenesulfonamide (3s)**

*N*-(1-(2-Fluoro-[1,1’biphenyl]-4-yl)ethyl)-4-methylbenzenesulfonamide (**3s**) was synthetized according to **GP1** using 2-(2-fluoro-[1,1’biphenyl]-4-yl)propanoic acid (rac. flurbiprofen, in total 97.6 mg, 0.40 mmol, 1.0 equiv.) as the limiting reagent and 4-methoxybenzenesulfonamide (in total 262 mg, 1.40 mmol, 3.50 equiv.) as nucleophile in excess. The reaction was irradiated at 385 nm for 16 h. After purification with flash column chromatography (EtOAc/PE, 5% → 50%) the title compound was obtained as a white solid (40 mg, 26%).

**R_f_** (silica, 50% EtOAc/PE): 0.80 [potassium permanganate];

**^1^H-NMR** (400 MHz, CDCl_3_): δ (ppm) 7.56–7.61 (m, 2H), 7.32–7.40 (m, 4H), 7.25–7.30 (m, 1H), 7.14–7.19 (m, 1H), 6.89 (dd, *J* = 8.0, 1.8 Hz, 1H), 6.79 (dd, *J* = 11.4, 1.8 Hz, 1H), 6.73–6.77 (m, 2H), 5.13–5.20 (m, 1H), 4.38–4.45 (m, 1H), 3.69 (s, 3H), 1.36 (d, *J* = 6.9 Hz, 3H);

**^13^C-NMR** (101 MHz, CDCl_3_): δ (ppm) 162.8, 159.5 (d, *J* = 250 Hz), 143.6 (d, *J* = 7.0 Hz), 135.3 (d, *J* = 1.0 Hz), 132.1, 130.7 (d, *J* = 3.9 Hz), 129.3, 128.8 (d, *J* = 2.9 Hz), 128.4, 128.0 (d, *J* = 13.5 Hz), 127.7, 122.2 (d, *J* = 3.3 Hz), 114.0 (d, *J* = 23.7 Hz), 113.9, 55.5, 53.0, 23.4;

**^19^F-NMR** (376 MHz, CDCl_3_, PhCF_3_ as internal standard): δ (ppm) –118.6 – (–)118.7 (m)

**HRMS** (ESI+): calc. [M+Na]^+^ for C_21_H_20_FNO_3_SNa 408.1046, found 408.1037, Δ = 0.9 mDa.

## 3.3 Scope of nucleophiles

**Synthesis of** ***N*-(1-(*o*-tolyl)ethyl)benzenesulfonamide (4a)**

*N*-(1-(*o*-Tolyl)ethyl)benzenesulfonamide (**4a**) was synthetized according to **GP1** using 2-(*o*-tolyl)propanoic acid (in total 65.7 mg, 0.40 mmol, 1.0 equiv.) as the limiting reagent and benzenesulfonamide (in total 220 mg, 1.40 mmol, 3.50 equiv.) as nucleophile. The reaction was irradiated at 385 nm for 16 h. After purification with flash column chromatography (Acetone/PE, 20% → 40%) the title compound was obtained as a colorless oil (45 mg, 41%).

**R_f_** (silica, 50% EtOAc/PE): 0.87 [potassium permanganate];

**^1^H-NMR** (400 MHz, CDCl_3_): δ (ppm) 7.67–7.71 (m, 2H), 7.47 (tt, *J* = 7.4, 1.2 Hz, 1H), 7.32–7.37 (m, 2H), 7.08–7.11 (m, 1H), 6.98–7.05 (m, 3H), 5.10 (d, *J* = 6.7 Hz, 1H), 4.76 (pent., *J* = 6.7 Hz, 1H), 2.19 (s, 3H), 1.40 (d, *J* = 6.7 Hz, 3H);

**^13^C-NMR** (101 MHz, CDCl_3_): δ (ppm) 140.6, 140.0, 134.5, 132.3, 130.4, 128.7, 127.3, 126.9, 126.4, 125.2, 49.8, 23.2, 18.9;

**HRMS** (ESI+): calc. [M+Na]^+^ for C_15_H_17_NO_2_SNa 298.0878, found 298.0874, Δ = 0.4 mDa.

**Synthesis of** **4-methyl-*N*-(1-(*o*-tolyl)ethyl)benzenesulfonamide (4b)**

4-Methyl-*N*-(1-(*o*-tolyl)ethyl)benzenesulfonamide (**4b**) was synthetized according to **GP1** using 2-(*o*-tolyl)propanoic acid (in total 65.7 mg, 0.40 mmol, 1.0 equiv.) as the limiting reagent and 4-methylbenzenesulfonamide (in total 240 mg, 1.40 mmol, 3.50 equiv.) as nucleophile. The reaction was irradiated at 385 nm for 16 h. After purification with flash column chromatography (Acetone/PE, 20% → 40%) the title compound was obtained as a colorless oil (59 mg, 51%).

**R_f_** (silica, 50% EtOAc/PE): 0.90 [potassium permanganate];

**^1^H-NMR** (400 MHz, CDCl_3_): δ (ppm) 7.58–7.62 (m, 2H), 7.12–7.15 (m, 3H), 6.98–7.06 (m, 3H), 5.37 (d, *J* = 6.6 Hz, 1H), 4.73 (pent., *J* = 6.6 Hz, 1H), 2.36 (s, 3H), 2.20 (s, 3H), 1.37 (d, *J* = 6.6 Hz, 3H);

**^13^C-NMR** (101 MHz, CDCl_3_): δ (ppm) 143.0, 140.4, 137.7, 134.4, 130.4, 129.3, 127.1, 127.0, 126.4, 125.4, 49.7, 23.1, 21.5, 19.0;

**HRMS** (ESI+): calc. [M+Na]^+^ for C_16_H_19_NO_2_SNa 312.1034, found 312.1031, Δ = 0.3 mDa.

**Synthesis of *N*-(1-(*o*-tolyl)ethyl)-4-(trifluoromethyl)benzenesulfonamide (4c)**

*N*-(1-(*o*-Tolyl)ethyl)-4-(trifluoromethyl)benzenesulfonamide (**4c**) was synthetized according to **GP2** using 4-(trifluoromethyl)benzenesulfonamide (in total 90.0 mg, 0.40 mmol, 1.0 equiv.) as the limiting reagent and 2-(*o*-tolyl)propanoic acid (in total 197 mg, 1.20 mmol, 3.0 equiv.) as acid in excess. The reaction was irradiated at 385 nm for 16 h. After purification with flash column chromatography (Acetone/PE, 20% → 40%) the title compound was obtained as an off-white solid (52 mg, 38%).

**R_f_** (silica, 50% EtOAc/PE): 0.95 [potassium permanganate];

**^1^H-NMR** (400 MHz, CDCl_3_): δ (ppm) 7.73 (d, *J* = 8.2 Hz, 2H), 7.53 (d, *J* = 8.2 Hz, 2H), 6.96–7.04 (m, 3H), 6.88–6.94 (m, 1H), 5.53 (d, *J* = 7.3 Hz, 1H), 4.87 (pent., *J* = 6.9 Hz, 1H), 2.26 (s, 3H), 1.43 (d, *J* = 6.9 Hz, 3H);

**^13^C-NMR** (101 MHz, CDCl_3_): δ (ppm) 144.1, 139.3, 134.4, 133.8 (q, *J* = 33 Hz), 130.4, 127.4, 127.3, 126.4, 125.6 (q, *J* = 3.7 Hz), 125.3, 123.2 (q, *J* = 272 Hz), 50.0, 29.7, 23.1, 19.0;

**^19^F-NMR** (376 MHz, CDCl_3_, PhCF_3_ as internal standard): δ (ppm) – 63.23 (s)

**HRMS** (ESI+): calc. [M+Na]^+^ for C_16_H_16_F_3_NO_2_SNa 366.0752, found 366.0748, Δ = 0.4 mDa.

**Synthesis of 4-cyano-*N*-(1-(*o*-tolyl)ethyl)benzenesulfonamide (4d)**

4-Cyano-*N*-(1-(*o*-tolyl)ethyl)benzenesulfonamide (**4d**) was synthetized according to **GP1** using 2-(*o*-tolyl)propanoic acid (in total 65.7 mg, 0.40 mmol, 1.0 equiv.) as the limiting reagent and 4-cyanobenzenesulfonamide (in total 255 mg, 1.40 mmol, 3.5 equiv.) as nucleophile in excess. The reaction was irradiated at 385 nm for 16 h. After purification with flash column chromatography (EtOAc/PE, 20% → 40%) the title compound was obtained as a pale oil which crystallized to a white solid upon standing under the ambient conditions (47 mg, 39%).

**R_f_** (silica, 50% EtOAc/PE): 0.76 [potassium permanganate];

**^1^H-NMR** (400 MHz, CDCl_3_): δ (ppm) 7.63–7.67 (m, 2H), 7.53–7.57 (m, 2H), 7.03–7.08 (m, 1H), 7.00–7.03 (m, 1H), 4.93 (d, *J* = 6.7 Hz, 1H), 4.88 (pent., *J* = 6.7 Hz, 1H), 2.25 (s, 3H), 1.44 (d, *J* = 6.7 Hz, 3H);

**^13^C-NMR** (101 MHz, CDCl_3_): δ (ppm) 144.9, 139.1, 134.7, 132.4, 130.6, 127.6, 127.4, 126.4, 125.2, 117.4, 115.8, 50.1, 23.2, 19.0;

**HRMS** (ESI+): calc. [M+Na]^+^ for C_16_H_16_N_2_O_2_SNa 323.0830, found 323.0826, Δ = 0.4 mDa.

**Synthesis of 2-fluoro-*N*-(1-(*o*-tolyl)ethyl)benzenesulfonamide (4e)**

2-Fluoro-*N*-(1-(*o*-tolyl)ethyl)benzenesulfonamide (**4e**) was synthetized according to **GP1** using 2-(*o*-tolyl)propanoic acid (in total 65.7 mg, 0.40 mmol, 1.0 equiv.) as the limiting reagent and 2-fluorobenzenesulfonamide (in total 210 mg, 1.40 mmol, 3.5 equiv.) as nucleophile in excess. The reaction was irradiated at 385 nm for 16 h. After purification with flash column chromatography (EtOAc/PE, 20% → 40%) the title compound was obtained as a colorless oil which crystallized to white solid upon standing under the ambient conditions (36 mg, 31%).

**R_f_** (silica, 50% EtOAc/PE): 0.86 [potassium permanganate];

**^1^H-NMR** (400 MHz, CDCl_3_): δ (ppm) 7.71 (td, *J* = 7.5, 1.8 Hz, 1H), 7.38–7.41 (m, 1H), 7.13–7.16 (m, 1H), 7.10 (dt, *J* = 7.7, 0.9 Hz, 1H), 6.97–7.05 (m, 3H), 6.92–6.97 (m, 1H), 5.15 (d, *J* = 7.6 Hz, 1H), 4.81 (pent., *J* = 7.0 Hz, 1H), 2.19 (s, 3H), 1.42 (d, *J* = 7.0 Hz, 3H);

**^13^C-NMR** (101 MHz, CDCl_3_): (ppm) 158.6 (d, *J* = 252 Hz), 139.6, 134.6 (d, *J* = 8.9 Hz), 134.4, 130.4, 129.7, 128.6 (d, *J* = 12.7 Hz), 126.8 (d, *J* = 102 Hz), 124.8, 124.1 (d, *J* = 3.8 Hz), 116.5 (d, *J* = 21.1 Hz), 49.9, 23.0, 18.9;

**^19^F-NMR** (376 MHz, CDCl_3_, PhCF_3_ as internal standard):

**HRMS** (ESI+): calc. [M+Na]^+^ for C_15_H_16_FNO_2_SNa 316.0784, found 316.0777, Δ = 0.7 mDa.

**Synthesis of** **3,5-dichloro-*N*-(1-(*o*-tolyl)ethyl)benzenesulfonamide (4f)**

3,5-Dichloro-*N*-(1-(*o*-tolyl)ethyl)benzenesulfonamide (**4f**) was synthetized according to **GP2** using 3,5-dichlorobenzenesulfonamide (in total 90.4 mg, 0.40 mmol, 1.0 equiv.) as the limiting reagent and 2-(*o*-tolyl)propanoic acid (in total 197 mg, 1.20 mmol, 3.0 equiv.) as acid in excess. The reaction was irradiated at 385 nm for 16 h. After purification with flash column chromatography (Acetone/PE, 20% → 40%) the title compound was obtained as a cream-white solid (72 mg, 52%).

**R_f_** (silica, 50% EtOAc/PE): 0.92 [potassium permanganate];

**^1^H-NMR** (400 MHz, CDCl_3_): δ (ppm) 7.40–7.42 (m, 2H), 7.33 (t, *J* = 1.8 Hz, 1H), 7.03–7.05 (m, 2H), 6.97–7.00 (m, 2H), 5.49 (br. s., 1H), 4.87 (pent., *J* = 7.0 Hz, 1H), 2.29 (s, 3H), 1.45 (d, *J* = 7.0 Hz, 3H);

**^13^C-NMR** (101 MHz, CDCl_3_): δ (ppm) 143.4, 138.9, 135.4, 134.5, 132.1, 130.5, 127.7, 126.3, 125.3, 125.2, 50.1, 23.1, 19.0;

**HRMS** (ESI+): calc. [M+Na]^+^ for C_15_H_15_Cl_2_NO_2_SNa 366.0098, found 366.0095, Δ = 0.3 mDa.

**Synthesis of 2,4,6-trimethyl-*N*-(1-(*o*-tolyl)ethyl)benzenesulfonamide (4g)**

2,4,6-Trimethyl-*N*-(1-(*o*-tolyl)ethyl)benzenesulfonamide (**4g**) was synthetized according to **GP2** using 2,4,6-trimethylbenzenesulfonamide (in total 79.7 mg, 0.40 mmol, 1.0 equiv.) as the limiting reagent and 2-(*o*-tolyl)propanoic acid (in total 197 mg, 1.20 mmol, 3.0 equiv.) as acid in excess. The reaction was irradiated at 385 nm for 16 h. After purification with flash column chromatography (Acetone/PE, 20% → 40%) the title compound was obtained as a cream-white solid (58 mg, 46%).

**R_f_** (silica, 50% EtOAc/PE): 0.92 [potassium permanganate];

**^1^H-NMR** (400 MHz, CDCl_3_): δ (ppm) 7.09–7.13 (m, 1H), 6.98–7.07 (m, 3H), 6.85 (s, 2H), 4.77 (d, *J* = 5.6 Hz, 1H), 4.71 (pent., *J* = 6.4 Hz, 1H), 2.53 (s, 6H), 2.26 (s, 3H), 2.17 (s, 3H), 1.36 (d, *J* = 6.4 Hz, 3H);

**^13^C-NMR** (101 MHz, CDCl_3_): δ (ppm) 141.9, 140.3, 138.8, 134.6, 134.5, 131.8, 130.5, 127.2, 126.2, 125.1, 49.5, 23.0, 22.8, 20.9, 18.8;

**HRMS** (ESI+): calc. [M+Na]^+^ for C_18_H_23_NO_2_SNa 340.1347, found 340.1345, Δ = 0.2 mDa.

**Synthesis of** ***N*-(1-(*o*-Tolyl)ethyl)naphthalene-1-sulfonamide (4h)**

*N*-(1-(*o*-Tolyl)ethyl)naphthalene-1-sulfonamide (**4h**) was synthetized according to **GP2** using napthalene-1-sulfonamide (in total 82.9 mg, 0.40 mmol, 1.0 equiv.) as the limiting reagent and 2-(*o*-tolyl)propanoic acid (in total 197 mg, 1.20 mmol, 3.0 equiv.) as acid in excess. The reaction was irradiated at 385 nm for 16 h. After purification with flash column chromatography (Acetone/PE, 30% → 40%) the title compound was obtained as a cream-white solid (41 mg, 32%).

**R_f_** (silica, 50% EtOAc/PE): 0.86 [potassium permanganate];

**^1^H-NMR** (400 MHz, CDCl_3_): δ (ppm) 8.22 (d, *J* = 1.4 Hz, 1H), 7.79–7.86 (m, 3H), 7.67 (dd, *J* = 8.7, 1.8 Hz, 1H), 7.54–7.63 (m, 2H), 7.06–7.10 (m, 1H), 6.98–6.95 (m, 3H), 4.94 (d, *J* = 6.6 Hz, 1H), 4.82 (pent., *J* = 6.6 Hz, 1H), 2.17 (s, 3H), 1.41 (d, *J* = 6.6 Hz, 3H);

**^13^C-NMR** (101 MHz, CDCl_3_): δ (ppm) 139.9, 137.4, 134.6, 134.5, 132.0, 130.4, 129.2, 129.1, 128.6, 128.3, 127.8, 127.3 (2C), 126.3, 125.1, 122.2, 49.9, 29.7, 23.1, 18.9;

**HRMS** (ESI+): calc. [M+Na]^+^ for C_19_H_19_NO_2_SNa 348.1034, found 348.1031, Δ = 0.3 mDa.

**Synthesis of** **methyl 4-(*N*-(1-(*o*-tolyl)ethyl)sulfamoyl)benzoate (4i)**

Methyl 4-(*N*-(1-(*o*-tolyl)ethyl)sulfamoyl)benzoate (**4i**) was synthetized according to **GP1** using 2-(*o*-tolyl)propanoic acid (in total 65.7 mg, 0.40 mmol, 1.0 equiv.) as the limiting reagent and methyl 4-sulfamoylbenzoate (in total 301 mg, 1.40 mmol, 3.5 equiv.) as nucleophile in excess. The reaction was irradiated at 385 nm for 16 h. After purification with flash column chromatography (EtOAc/PE, 20% → 40%) the title compound was obtained as an off-white solid (40 mg, 30%).

**R_f_** (silica, 50% EtOAc/PE): 0.73 [potassium permanganate];

**^1^H-NMR** (400 MHz, CDCl_3_): δ (ppm) 7.95–8.00 (m, 2H), 7.67–7.72 (m, 2H), 6.95–7.05 (m, 4H), 4.90 (d, *J* = 6.7 Hz, 1H), 4.82 (pent., *J* = 6.7 Hz, 1H), 3.94 (s, 3H), 2.22 (s, 3H), 1.42 (d, *J* = 6.7 Hz, 3H);

**^13^C-NMR** (101 MHz, CDCl_3_): δ (ppm) 165.6, 144.7, 139.5, 134.6, 133.4, 130.6, 129.9, 127.5, 126.9, 126.4, 125.2, 52.6, 49.9, 23.1, 19.0;

**HRMS** (ESI+): calc. [M+Na]^+^ for C_17_H_19_NO_4_SNa 356.0933, found 356.0927, Δ = 0.6 mDa.

**Synthesis of** ***N*-(1-(*o*-tolyl)ethyl)thiophene-2-sulfonamide (4j)**

*N*-(1-(*o*-Tolyl)ethyl)thiophene-2-sulfonamide (**4j**) was synthetized according to **GP2** using thiophene-2-sulfonamide (in total 65.2 mg, 0.40 mmol, 1.0 equiv.) as the limiting reagent and 2-(*o*-tolyl)propanoic acid (in total 197 mg, 1.20 mmol, 3.0 equiv.) as acid in excess. The reaction was irradiated at 385 nm for 16 h. After purification with flash column chromatography (EtOAc/PE, 20% → 40%) the title compound was obtained as a colorless oil which crystallized to white solid upon standing under the ambient conditions (25 mg, 22%).

**R_f_** (silica, 50% EtOAc/PE): 0.87 [potassium permanganate];

**^1^H-NMR** (400 MHz, CDCl_3_): δ (ppm) 7.46 (dd, *J* = 5.0, 1.3 Hz, 1H), 7.38 (dd, *J* = 3.8, 1.3 Hz, 1H), 7.14–7.17 (m, 1H), 7.04–7.10 (m, 3H), 5.11 (d, *J* = 5.7 Hz, 1H), 4.83 (pent., *J* = 6.6 Hz, 1H), 2.25 (s, 3H), 1.44 (d, *J* = 6.8 Hz, 3H);

**^13^C-NMR** (101 MHz, CDCl_3_): δ (ppm) 141.7, 140.0, 134.5, 132.1, 131.7, 130.5, 127.4, 127.1, 126.5, 125.1, 50.2, 23.2, 19.0;

**HRMS** (ESI+): calc. [M+Na]^+^ for C_13_H_15_NO_2_S_2_Na 304.0442, found 304.0434, Δ = 0.8 mDa.

**Synthesis of** **4’-fluoro-*N*-(1-(*o*-tolyl)ethyl-[1,1’-biphenyl]-4-sulfonamide (4k)**

4’-Fluoro-*N*-(1-(*o*-tolyl)ethyl-[1,1’-biphenyl]-4-sulfonamide (**4k**) was synthetized according to **GP2** using 4’-fluoro-[1,1’-biphenyl]-4-sulfonamide (in total 100.4 mg, 0.40 mmol, 1.0 equiv.) as the limiting reagent and 2-(*o*-tolyl)propanoic acid (in total 197 mg, 1.20 mmol, 3.0 equiv.) as acid in excess. The reaction was irradiated at 385 nm for 16 h. After purification with flash column chromatography (Acetone/PE, 20% → 40%) the title compound was obtained as a white solid (87 mg, 59%).

**R_f_** (silica, 50% EtOAc/PE): 0.73 [potassium permanganate];

**^1^H-NMR** (400 MHz, CDCl_3_): δ (ppm) 7.96–8.00 (m, 1H), 7.65–7.69 (m, 1H), 7.54–7.59 (m, 1H), 7.27–7.33 (m, 1H), 7.15–7.21 (m, 4H), 5.03 (br. s., 1H), 3.99 (q., *J* = 7.2 Hz, 1H), 2.38 (s, 3H), 1.49 (d, *J* = 7.2 Hz, 3H);

**^13^C-NMR** (101 MHz, CDCl_3_): δ (ppm) 180.0, 161.9, 144.8, 140.5, 137.0 (d, *J* = 246 Hz), 135.9, 130.6, 130.4 (d, *J* = 13.6 Hz), 129.0 (d, *J* = 8.5 Hz), 129.0 (d, *J* = 9.0 Hz), 127.4 (d, *J* = 41.3 Hz), 127.1, 126.5 (d, *J* = 9.6 Hz), 116.1 (d, *J* = 21.7 Hz), 41.0, 19.6, 17.6;

**^19^F-NMR** (376 MHz, CDCl_3_, PhCF_3_ as internal standard): δ (ppm) –114.4 – (–)114.5 (m)

**HRMS** (ESI+): calc. [M+Na]^+^ for C_21_H_20_FNO_2_SNa 392.1097, found 392.1082, Δ = 1.5 mDa.

**Synthesis of 4-methyl-*N*-phenyl-*N*-(1-(*o*-tolyl)ethyl)benzenesulfonamide (4l)**

4-Methyl-*N*-phenyl-*N*-(1-(*o*-tolyl)ethyl)benzenesulfonamide (**4l**) was synthetized according to **GP2** using 4-methyl-*N*-phenylsulfonamide (in total 98.2 mg, 0.40 mmol, 1.0 equiv.) as the limiting reagent and 2-(*o*-tolyl)propanoic acid (in total 197 mg, 1.20 mmol, 3.0 equiv.) as acid in excess. The reaction was irradiated at 385 nm for 16 h. After purification with flash column chromatography (EtOAc/PE, 20% → 40%) the title compound was obtained as a white solid (80 mg, 55%).

**R_f_** (silica, 50% EtOAc/PE): 0.90 [potassium permanganate];

**^1^H-NMR** (400 MHz, CDCl_3_): δ (ppm) 7.62–7.67 (m, 2H), 7.17–7.22 (m, 4H), 7.11–7.13 (m, 2H), 6.99–7.03 (m, 2H), 6.94–6.98 (m, 2H), 4.24 (q., *J* = 7.1 Hz, 1H), 2.37 (s, 3H), 2.17 (s, 3H), 1.54 (d, *J* = 7.1 Hz, 3H);

**^13^C-NMR** (101 MHz, CDCl_3_): δ (ppm) 143.7, 143.6, 143.5, 136.2, 136.0, 134.3, 130.4, 129.6, 128.5, 127.3, 126.6, 126.2, 126.1, 121.9, 40.4, 21.9, 21.6, 19.7;

**HRMS** (ESI+): calc. [M+Na]^+^ for C_22_H_23_NO_2_SNa 388.1347, found 388.1345, Δ = 0.2 mDa.

**Synthesis of *N*-phenyl-*N*-(1-(*o*-tolyl)ethyl)cyclopropanesulfonamide (4m)**

*N*-Phenyl-*N*-(1-(*o*-tolyl)ethyl)cyclopropanesulfonamide (**4m**) was synthetized according to **GP2** using *N*-phenylcyclopropanesulfonamide (in total 78.9 mg, 0.40 mmol, 1.0 equiv.) as the limiting reagent and 2-(*o*-tolyl)propanoic acid (in total 197 mg, 1.20 mmol, 3.0 equiv.) as acid in excess. The reaction was irradiated at 385 nm for 16 h. After purification with flash column chromatography (H_2_O+0.5‰ TFA/MeCN, 10% → 100%) the title compound was obtained as a colorless oil (81 mg, 64%).

**R_f_** (silica, 50% EtOAc/PE): 0.97 [potassium permanganate];

**^1^H-NMR** (400 MHz, CDCl_3_): δ (ppm) 7.13–7.31 (m, 9H), 6.88 (br. s., 1H), 4.33 (q., *J* = 7.2 Hz, 1H), 2.49 (tt, *J* = 8.0, 4.8 Hz, 1H), 2.26 (s, 3H), 1.63 (d, *J* = 7.2 Hz, 3H), 1.14–1.19 (m, 2H), 0.92–0.98 (m, 2H);

**^13^C-NMR** (101 MHz, CDCl_3_): δ (ppm) 143.7, 143.6, 136.0, 134.5, 130.5, 128.6, 126.6, 126.2, 126.1, 122.3, 40.5, 29.7, 22.1, 19.8, 5.6;

**HRMS** (ESI+): calc. [M+NH_4_]^+^ for C_18_H_21_NO_2_SNH_4_ 333.1637, found 333.1633, Δ = 0.4 mDa.

**Synthesis of *N*-phenyl-*N*-(1-(*o*-tolyl)ethyl)cyclohexanesulfonamide (4n)**

*N*-Phenyl-*N*-(1-(*o*-tolyl)ethyl)cyclohexanesulfonamide (**4m**) was synthetized according to **GP2** using *N*-phenylcyclohexanesulfonamide (in total 95.7 mg, 0.40 mmol, 1.0 equiv.) as the limiting reagent and 2-(*o*-tolyl)propanoic acid (in total 197 mg, 1.20 mmol, 3.0 equiv.) as acid in excess. The reaction was irradiated at 385 nm for 16 h. After purification with flash column chromatography (H_2_O+0.5‰ TFA/MeCN, 10% → 100%) the title compound was obtained as a yellow-orange oil (88 mg, 62 %).

**R_f_** (silica, 50% EtOAc/PE): 0.95 [potassium permanganate];

**^1^H-NMR** (400 MHz, CDCl_3_): δ (ppm) 7.27–7.31 (m, 1H), 7.21–7.26 (m, 1H), 7.11–7.19 (m, 6H), 6.99 (s, 1H), 4.32 (q., *J* = 7.1 Hz, 1H), 3.02 (tt, *J* = 12.0, 3.2 Hz, 1H), 2.27 (s, 3H), 2.19 (d, *J* = 12.0 Hz, 2H), 1.88 (d, *J* = 10.6 Hz, 2H), 1.67–1.72 (m, 1H), 1.63 (d, *J* = 7.1 Hz, 3H), 1.53–1.59 (m, 2H), 1.13–1.30 (m, 3H);

**^13^C-NMR** (101 MHz, CDCl_3_): δ (ppm) 130.5, 128.7 (2C), 126.6, 126.2, 126.1, 120.5 (2C), 60.3, 40.4, 26.3, 25.1, 22.1, 19.8;

**HRMS** (ESI+): calc. [M+Na]^+^ for C_21_H_27_NO_2_SNa 380.1660, found 380.1658, Δ = 0.2 mDa.

**Synthesis of methyl 2,3-dimethoxy-5-(*N*-(1-(*o*-tolyl)ethyl)sulfamoylbenzoate (4o)**

Methyl 2,3-dimethoxy-5-(*N*-(1-(*o*-tolyl)ethyl)sulfamoylbenzoate (**4o**) was synthetized according to **GP2** using methyl 2,3-dimethoxy-5-sulfamylbenzoate (in total 110.0 mg, 0.40 mmol, 1.0 equiv.) as the limiting reagent and 2-(*o*-tolyl)propanoic acid (in total 197 mg, 1.20 mmol, 3.0 equiv.) as acid in excess. The reaction was irradiated at 385 nm for 16 h. After purification with flash column chromatography (EtOAc/PE, 20% → 40%) the title compound was obtained as a colorless oil which crystallized to white solid upon standing under the ambient conditions (40 mg, 25%).

**R_f_** (silica, 50% EtOAc/PE): 0.77 [potassium permanganate];

**^1^H-NMR** (400 MHz, CDCl_3_): δ (ppm) 7.65 (d, *J* = 2.2 Hz, 1H), 7.15 (d, *J* = 2.2 Hz, 1H), 6.94–7.02 (m, 4H), 5.32 (d, *J* = 7.3 Hz, 1H), 4.81 (pent., *J* = 6.9 Hz, 1H), 3.89 (s, 3H), 3.87 (s, 3H), 2.23 (s, 3H), 1.42 (d, *J* = 6.9 Hz, 3H);

**^13^C-NMR** (101 MHz, CDCl_3_): δ (ppm) 165.0, 153.3, 152.2, 139.7, 135.6, 134.4, 130.3, 127.3, 126.2, 125.3, 121.5, 113.3, 110.0, 61.6, 56.1, 52.4, 49.9, 23.4, 19.0;

**HRMS** (ESI+): calc. [M+H]^+^ for C_19_H_24_NO_6_SNa 394.1324, found 394.1320, Δ = 0.4 mDa.

**Synthesis of** **4-(5-(*p*-tolyl)-3-(trifluoromethyl)-1*H*-pyrazol-1-yl)-*N*-(1-(*o*-tolyl)ethyl)benzenesulfonamide (4p)**

4-(5-(*p*-Tolyl)-3-(trifluoromethyl)-1*H*-pyrazol-1-yl)-*N*-(1-(*o*-tolyl) ethyl)benzenesulfonamide (**4x**) was synthetized according to **GP2** using 4-(5-(*p*-tolyl)-3-(trifluoromethyl)-1*H*-pyrazol-1-yl)benzenesulfonamide (celebrex, in total 152.4 mg, 0.40 mmol, 1.0 equiv.) as the limiting reagent and 2-(*o*-tolyl)propanoic acid (in total 197 mg, 1.20 mmol, 3.0 equiv.) as acid in excess. The reaction was irradiated at 385 nm for 16 h. After purification with flash column chromatography (EtOAc/PE, 20% → 40%) the title compound was obtained as a colorless oil which crystallized to white solid upon standing under the ambient conditions (136 mg, 68%).

**R_f_** (silica, 50% EtOAc/PE): 0.95 [potassium permanganate];

**^1^H-NMR** (400 MHz, CDCl_3_): δ (ppm) 7.63–7.67 (m, 2H), 7.29–7.32 (m, 2H), 7.15–7.18 (m, 2H), 7.03–7.11 (m, 6H), 7.67 (s, 1H), 5.07 (d, *J* = 6.6 Hz, 1H), 4.79 (pent., *J* = 6.7 Hz, 1H), 2.38 (s, 3H), 2.24 (s, 3H), 1.40 (d, *J* = 6.7 Hz, 3H);

**^13^C-NMR** (101 MHz, CDCl_3_): δ (ppm) 145.1, 144.1 (q, *J* = 38 Hz), 142.2, 140.2, 139.8, 139.7, 134.5, 130.6, 129.7, 128.7, 127.9, 127.6, 126.5, 125.8, 125.3, 125.1, 121.0 (q, *J* = 268 Hz), 49.9, 23.1, 21.3, 19.1;

**^19^F-NMR** (376 MHz, CDCl_3_, PhCF_3_ as internal standard): δ (ppm) – 63.5 (s)

**HRMS** (ESI+): calc. [M+H]^+^ for C_26_H_25_F_3_N_3_O_2_S 500.1620, found 500.1617, Δ = 0.3 mDa.

# 4. Synthesis and characterization of starting materials

## 4.1 Synthesis of benzylic carboxylic acids

### 4.1.1 General Procedure for methylation of benzylic acids (GP3)^[1]^

The phenyl acetic acid derivative (20 mmol, 1.00 equiv.) was added into a flame-dried Schlenk flask equipped with stirring bar under N_2_. Dry THF (100 mL, 0.2 M) was added *via* syringe and the mixture was cooled down to –78 °C. At this temperature, *n*-butyllithium (1.6 M in hexanes, 26 mL, 42 mmol, 2.10 equiv.) was added in a dropwise manner. During the addition of the first equivalent, the reaction mixture turned white and turbid, the second equivalent gave the reaction a yellow-orange color as indication of the benzylic deprotonation. These color changes can be used to qualitatively follow the reaction progress after the addition of the electrophile. In cases where the substrate fell out of the solution due to the low solubility of the lithium carboxylate, the reaction was shaken in between the addition of the second BuLi-equivalent to improve the re-solvation of the anionic intermediate. To ensure full deprotonation, the mixture was allowed to come to –20 °C over 1–2 hours, after which it was cooled down again to –78 °C before the addition of MeI (1.37 mL, 22 mmol, 1.10 equiv.). The reaction mixture was then stirred for two more hours while allowing to gently warm up.

### 4.1.2 General Procedure for alkylation of phenylacetic acid (GP4)

($\pm$)2-Phenylacetic acid (2.0 g, 15.0 mmol, 1.0 equiv.) was added into a flame-dried Schlenk flask equipped with stirring bar under N_2_. Dry THF (75 mL, 0.2 M) was added *via* syringe and the mixture was cooled down to –78 °C. At this temperature, *n*-butyllithium (1.6 M in hexanes, 20 mL, 21 mmol, 2.10 equiv.) was added in a dropwise manner. The reaction mixture was then stirred for 90 minutes while allowing to gently warm up, after which it was cooled back to –78 °C and the bromide (1.1 equiv.) was added dropwise. The mixture was allowed to warm up again towards room temperature, and after (typically) two hours.

### 4.1.3 General Procedure for the workup of methylation/alkylation reactions

The reaction was quenched with sat. NH_4_Cl (10 mL) and acidified with 2M HCl to pH 1. Et_2_O (50 mL) was added, and the phases were separated. The aqueous layer was extracted with Et_2_O (3 $\times$ 30 mL), and the combined organic layers were washed with sat. aq. sodium dithionite solution (2$\times$ 50 mL) and brine (50 mL). The organic layers were dried over Mg_2_SO_4_, the drying reagent filtered off and the solvent evaporated under reduced pressure. The crude product was purified by flash column chromatography (MeOH/DCM, 5% → 10%).

### 4.1.2 List of synthetized carboxylic acids

**Synthesis of 2-(*m*-tolyl)propanoic acid (1d)**

Synthetized according to **GP3** using 2-(*m*-tolyl)acetic acid (3.00 g, 20.0 mmol, 1.00 equiv.) as starting material. After purification with flash column chromatography (MeOH/DCM, 5%), the title compound was obtained as a yellowish oil which eventually crystallized to a pale solid under ambient conditions (1.45 g, 44%).

**R_f_** (silica, 5% MeOH/DCM) 0.26 [bromocresol green]

**^1^H-NMR** (400 MHz, CDCl_3_): δ (ppm) 11.96 (br. s., 1H), 7.34–7.39 (m, 1H), 7.21–7.30 (m, 3H), 3.85 (q, *J* = 7.15 Hz, 1H), 2.49 (s, 3H), 1.65 (d, *J* = 7.15 Hz, 3H);

**^13^C-NMR** (101 MHz, CDCl_3_): δ (ppm) 181.5, 139.9, 138.5, 128.8, 128.5, 128.3, 124.8, 45.5, 21.5, 18.2;

**HRMS** (EI+): calc. [M]^∙+^ for C_10_H_12_O_2_^∙+^ 164.08318, found 164.08304, Δ = 0.14 mDa.

**Synthesis of 2-(*o*-tolyl)propanoic acid (1e)**

Synthetized according to **GP3** using 2-(*o*-tolyl)acetic acid (3.00 g, 20.0 mmol, 1.00 equiv.) as a starting material. After purification with flash column chromatography (MeOH/DCM, 5%), the title compound was obtained as a white hard solid (2.11 g, 64%).

**R_f_** (silica, 5% MeOH/DCM) 0.44 [bromocresol green]

**^1^H-NMR** (400 MHz, CDCl_3_): δ (ppm) 7.28–7.31 (m, 1H), 7.16–7.23 (m, 3H), 3.99 (q, *J* = 7.15 Hz, 1H), 2.39 (s, 3H), 1.50 (d, *J* = 7.15 Hz, 3H);

**^13^C-NMR** (101 MHz, CDCl_3_): δ (ppm) 180.7, 138.3, 135.9, 130.6, 127.2, 126.6, 126.5, 41.1, 19.7, 17.5;

**HRMS** (EI+): calc. [M]^∙+^ for C_10_H_12_O_2_^∙+^ 164.08318, found 164.08321, Δ = – 0.03 mDa.

**Synthesis of** **2-(4-(*tert*-butyl)phenyl)propanoic acid (1f)**

Synthetized according to **GP3** using 2-(4-*tert*-butyl)phenylacetic acid (3.85 g, 20.0 mmol, 1.00 equiv.) as a starting material. After purification with flash column chromatography (MeOH/DCM, 5%), the title compound was obtained as an off-white solid (1.20 g, 29%).

**R_f_** (silica, 5% MeOH/DCM) 0.41 [bromocresol green]

**^1^H-NMR** (400 MHz, CDCl_3_): δ (ppm) 7.25–7.29 (m, 2H), 7.15–7.19 (m, 2H), 3.63 (q., *J* = 7.1 Hz, 1H), 1.42 (d, *J* = 7.1 Hz, 3H), 1.23 (s, 9H);

**^13^C-NMR** (101 MHz, CDCl_3_): δ (ppm) 181.0, 150.3, 136.6, 127.2, 125.6, 44.9, 34.5, 31.3, 18.0;

**HRMS** (EI+): calc. [M]^∙+^ for C_13_H_18_O_2_^∙+^ 206.13013, found 206.13037, Δ = – 0.24 mDa.

**Synthesis of 2-(4-fluorophenyl)propanoic acid (1g)**

Synthetized according to **GP3** using 2-(4-fluorophenyl)acetic acid (3.80 g, 20.0 mmol, 1.00 equiv.) as starting material. After purification with flash column chromatography (MeOH/DCM, 5%) and drying in high vacuo, the title compound was obtained as a yellow oil (2.9 g, 86%).

**R_f_** (silica, 5% MeOH/DCM) 0.64 [bromocresol green]

**^1^H-NMR** (400 MHz, CDCl_3_): δ (ppm) 11.42 (br. s., 1H), 7.27–7.33 (m, 2H), 7.00–7.05 (m, 2H), 3.70–3.77 (m, 1H), 1.49–1.53 (m, 3H);

**^13^C-NMR** (101 MHz, CDCl_3_): δ (ppm) 181.0, 162.2 (d, *J* = 246 Hz), 131.0 (d, *J* = 8.1 Hz), 129.2 (d, *J* = 7.8 Hz), 115.5 (d, *J* = 21.5 Hz), 44.7, 18.2;

**^19^F-NMR** (376 MHz, CDCl_3_, PhCF_3_ as internal standard): δ (ppm) –115.31 – (–)115.40 (m)

**HRMS** (EI+): calc. [M]^∙+^ for C_9_H_9_FO_2_^∙+^ 168.05811, found 168.05833, Δ = – 0.22 mDa.

**Synthesis of** **2-(4-chlorophenyl)propanoic acid (1h)**

Synthetized according to a modified literature procedure over two steps.**^[2]^** 2-(4-chlorophenyl)acetonitrile (3.82 mL, 30.0 mmol, 1.00 equiv.) was dissolved to THF (30 mL) cooled to 0 °C. NaH (60% in mineral oil, 1.32 g, 33.0 mmol, 1.10 equiv.) was added portionwise and the reaction mixture was stirred for 3 h while allowing to warm towards room temperature. After this the mixture was cooled again to 0 °C and MeI (2.05 mL, 33.0 mmol, 1.10 equiv.) was added dropwise. Cooling bath was then removed and the reaction was stirred overnight at room temperature. The reaction was quenched with sat. NH_4_Cl (20 mL), diluted with water (200 mL) and extracted with EtOAc (3 $\times$ 50 mL). The combined organic phases were washed with water (100 mL) and brine (100 mL), dried over Mg_2_SO_4_, filtrated and the solvent was removed under reduced pressure. The so-obtained crude product (3.98 g, 80 %) was used in the following step without further purification.

For the hydrolysis of the nitrile, the afore mentioned crude product (3.98 g, 24.0 mmol, 1.00 equiv.) was dissolved into ethylene glycol (40 mL) and KOH (14 g, 150 mmol, 6 equiv.) was added. The reaction was then refluxed at 168 °C overnight. After cooling to room temperature water and ethyl acetate were added, the reaction was acidified with 2M HCl (pH=1) and the phases then separated. The aqueous phase was extracted with EtOAc (3$\times$), the combined organic phases dried over Mg_2_SO_4_, filtered and concentrated under reduced pressure. Purification with flash column chromatography (EtOAc/PE, 30%) followed by removal of the solvents afforded the title compound as a white solid (1.36 g, 31 %; overall yield over two steps 25%).

**R_f_** (silica, 5% MeOH/DCM) 0.36 [bromocresol green]

**^1^H-NMR** (400 MHz, CDCl_3_): δ (ppm) 7.26–7.35 (m, 5H), 3.75 (q, *J* = 7.2 Hz, 1H), 1.53 (d, *J* = 7.2 Hz, 3H);

**^13^C-NMR** (101 MHz, CDCl_3_): δ (ppm) 179.9, 138.1, 133.3, 129.0, 128.8, 128.6, 127.3, 44.7, 26.2, 18.1;

**HRMS** (EI+): calc. [M]^∙+^ for C_9_H_9_ClO_2_^∙+^ 184.02856, found 184.02935, Δ = – 0.79 mDa.

**Synthesis of 2-(4-trifluoromethoxy)phenyl)propanoic acid (1i)**

Synthetized according to **GP3** using 2-(4-trifluoromethoxy)phenyl)acetic acid (780 mg, 3.5 mmol, 1.00 equiv.) as starting material. After purification with flash column chromatography (MeOH/DCM, 5%) and drying in high vacuo, the title compound was obtained as a yellow oil (606 mg, 73%).

**R_f_** (silica, 5% MeOH/DCM) 0.80 [bromocresol green]

**^1^H-NMR** (400 MHz, CDCl_3_): δ (ppm) 11.52 (br. s., 1H), 7.35–7.40 (m, 2H), 7.20 (app. d., *J* = 8.0 Hz, 2H), 3.78 (q, *J* = 7.2 Hz, 1H), 1.54 (d, *J* = 7.2 Hz, 3H);

**^13^C-NMR** (101 MHz, CDCl_3_): δ (ppm) 180.8, 148.5, 138.3, 129.1, 121.1, 120.4 (q., *J* = 257 Hz) 44.8, 18.0;

**^19^F-NMR** (376 MHz, CDCl_3_, PhCF_3_ as internal standard): δ (ppm) – 58.90 (s)

**HRMS** (EI+): calc. [M]^∙+^ for C_10_H_9_F_3_O_2_^∙+^ 234.04983, found 234.04999, Δ = – 0.16 mDa.

**Synthesis of** **2-phenylhexanoic acid (1m)**

Synthetized according to **GP4** using phenylacetic acid (2.0 g, 15.0 mmol, 1.0 equiv.) as limiting reagent and *n*-bromobutane (1.1 equiv.) as electrophilic coupling partner. After purification with flash column chromatography (MeOH/DCM, 2.5%) the title compound was obtained as a yellow oil (2.29 g, 79 %).

**R_f_** (silica, 5% MeOH/DCM) 0.75 [bromocresol green]

**^1^H-NMR** (400 MHz, CDCl_3_): δ (ppm) 11.11 (br. s, 1H), 7.28–7.37 (m, 5H), 3.57 (t, *J* = 7.7 Hz, 1H), 2.06–2.16 (m, 1H), 1.77–1.87 (m, 1H), 1.19–1.41 (m, 4H), 0.90 (t, *J* = 7.2 Hz, 3H);

**^13^C-NMR** (101 MHz, CDCl_3_): δ (ppm) 180.4, 138.6, 128.6, 128.1, 127.4, 51.6, 32.8, 29.6, 22.4, 13.8;

**HRMS** (EI+): calc. [M]^∙+^ for C_12_H_16_O_2_^∙+^ 192.11448, found 192.11447, Δ = 0.01 mDa.

**Synthesis of** **2,3-diphenylpropanoic acid (1n)**

Synthetized according to **GP4** using phenylacetic acid (2.0 g, 15.0 mmol, 1.0 equiv.) as limiting reagent and benzyl bromide (1.1 equiv.) as electrophilic coupling partner. After purification with flash column chromatography (MeOH/DCM, 2.5%) the title compound was obtained as a white solid (1.25 g, 37 %).

**R_f_** (silica, 5% MeOH/DCM) 0.28 [bromocresol green]

**^1^H-NMR** (400 MHz, CDCl_3_): δ (ppm) 7.10–7.27 (m, 8H), 7.04–7.08 (m, 2H), 3.81 (dd, *J* = 8.7, 7.0 Hz, 1H), 3.36 (dd, *J* = 14.2, 8.4 Hz, 1H), 2.99 (dd, *J* = 14.2, 7.0 Hz, 1H);

**^13^C-NMR** (101 MHz, CDCl_3_): δ (ppm)

**HRMS** (EI+): calc. [M]^∙+^ for C_15_H_14_O_2_^∙+^ 226.09883, found 226.09852, Δ = 0.31 mDa.

**Synthesis of** **3-cyclohexyl-2-phenylpropanoic acid (1o)**

Synthetized according to **GP4** using phenylacetic acid (2.0 g, 15.0 mmol, 1.0 equiv.) as limiting reagent and (bromomethyl)cyclohexane (1.1 equiv.) as electrophilic coupling partner. After purification with normal phase flash column chromatography (MeOH/DCM, 5 %) on silica followed by a reverse-phase column chromatography (H_2_O+0.5‰ TFA/MeCN, 10% → 100%), the title compound was obtained as a white solid (875 mg, 25 %).

**R_f_** (silica, 5% MeOH/DCM) 0.45 [bromocresol green]

**^1^H-NMR** (400 MHz, CDCl_3_): δ (ppm) 7.24–7.33 (m, 5H), 3.70 (t, *J* = 7.8 Hz, 1H), 1.93–2.02 (m, 1H), 1.71–1.77 (m, 1H), 1.57–1.70 (m, 4H), 1.09–1.22 (m, 4H), 0.84–0.96 (m, 2H);

**^13^C-NMR** (101 MHz, CDCl_3_): δ (ppm) 179.0, 138.8, 128.7, 128.1, 127.4, 48.5, 40.7, 35.1, 33.3, 32.9, 26.5, 26.1, 26.0;

**HRMS** (EI+): calc. [M]^∙+^ for C_15_H_20_O_2_^∙+^ 232.14578, found 232.14632, Δ = – 0.54 mDa.

**Synthesis of** **2-cyclohexyl-2-phenylacetic acid (1p)**

Synthetized according to **GP4** using phenylacetic acid (2.0 g, 15.0 mmol, 1.0 equiv.) as limiting reagent and cyclohexyl bromide (1.1 equiv.) as electrophilic coupling partner. After purification with normal phase flash column chromatography (MeOH/DCM, 5 %) on silica followed by a reverse-phase column chromatography (H_2_O+0.5‰ TFA/MeCN, 10% → 100%), the title compound was obtained as a white solid (1.03 g, 32 %).

**R_f_** (silica, 5% MeOH/DCM) 0.38 [bromocresol green]

**^1^H-NMR** (400 MHz, CDCl_3_): δ (ppm) 10.5 (br. s., 1H), 7.28–7.39 (m, 5H), 3.26 (d, *J* = 10.9 Hz, 1H), 2.05 (qt, *J* = 10.9, 3.3 Hz, 1H), 1.91–1.98 (m, 1H), 1.75–1.82 (m, 1H), 1.60–1.70 (m, 2H), 1.29–1.41 (m, 2H), 1.06–1.24 (m, 3H), 0.73–0.83 (m, 1H);

**^13^C-NMR** (101 MHz, CDCl_3_): δ (ppm) 180.4, 137.3, 128.7, 128.5, 127.4, 58.9, 40.7, 32.0, 30.3, 26.3, 26.0, 25.9;

**HRMS** (EI+): calc. [M] ^∙+^ for C_14_H_18_O_2_ 218.13013, found 218.13019, Δ = – 0.16 mDa.

**Synthesis of 2-(naphthalen-1-yl)propanoic acid (1q)**

Synthetized according to **GP3** using 2-(napthalen-1-yl)acetic acid (3.72 g, 20.0 mmol, 1.00 equiv.) as starting material. After purification with flash column chromatography (MeOH/DCM, 5%) and drying in high-vacuo, the title compound was obtained as an off-white solid (1.09 g, 18%).

**R_f_** (silica, 5% MeOH/DCM) 0.29 [bromocresol green]

**^1^H-NMR** (400 MHz, CDCl_3_): δ (ppm) 8.10 (d, *J* = 8.4 Hz, 1H), 7.88 (dd, *J* = 7.9, 1.4 Hz, 1H), 7.79 (d, *J* = 7.9 Hz, 1H), 7.44–7.58 (m, 4H), 4.55 (q, *J* = 7.2 Hz, 1H), 1.68 (d, *J* = 7.2 Hz, 3H);

**^13^C-NMR** (101 MHz, CDCl_3_): δ (ppm) 180.7, 135.9, 133.9, 131.3, 129.0, 128.0, 126.5, 125.7, 125.5, 124.6, 123.0, 41.0, 17.8;

**HRMS** (EI+): calc. [M]^∙+^ for C_13_H_12_O_2_^∙+^ 200.08318, found 200.08357, Δ = – 0.39 mDa.

### 4.1.3 List of synthetized unsuccessful carboxylic acids

**Synthesis of 1-phenylcyclopentane-1-carboxylic acid (S1)**

Synthetized in a two-step manner starting from 2-phenylacetonitrile following a literature-reported procedure.^[3]^ NaH (60% suspension in mineral oil, 1.92 g, 48 mmol, 2.4 equiv.) was suspended into DMF (14 mL) and cooled to 0 °C. A solution of 2-phenylacetonitrile (2.34 g, 20.00 mmol, 1.00 equiv.) in DMF (22 mL) was added dropwise to the suspension and the reaction was stirred for 1 h while allowing to warm towards room temperature. 1,4-Dibromobutane (5.16 g, 24 mmol, 1.2 equiv.) was added dropwise at room temperature, after which the reaction was stirred for 5 hours. The reaction was then quenched with water (10 mL). The mixture was extracted with EtOAc (3 $\times$ 15 mL), the combined organic layers washed with brine (20 mL) and eventually dried over MgSO_4_. The drying agent was filtered off and the solvent removed under reduced pressure. After purification with flash column chromatography (EtOAc/PE, 10%), the 1-phenylcyclopentane-1-carbonitrile was obtained as a yellow oil (1.03 g, 30%).

For the hydrolysis of the nitrile group into carboxylic acid, the previously obtained 1-phenylcyclopentane-1-carbonitrile (1.03 g, 6.00 mmol, 1.00 equiv.) was dissolved in ethylene glycol (10 mL) and KOH (2.0 g, 36.0 mmol, 6.00 equiv.) was added. The reaction was then refluxed at 168 °C overnight. After cooling to room temperature water and ethyl acetate were added, the reaction was acidified with 2M HCl (pH=1) and the phases then separated. The aqueous phase was extracted with EtOAc (3$\times$), the combined organic phases dried over Mg_2_SO_4_, filtered and concentrated under reduced pressure. Purification with flash column chromatography (EtOAc/PE, 30%), the 1-phenylcyclopentane-1-carboxylic acid was obtained as a white solid (0.45 g, 39%).

**R_f_** (silica, 5% MeOH/DCM) 0.35 [bromocresol green]

**^1^H-NMR** (400 MHz, CDCl_3_): δ (ppm) 11.2 (br. s., 1H), 7.38–7.42 (m, 2H), 7.29–7.34 (m, 2H), 7.22–7.27 (m, 1H), 2.63–2.70 (m, 2H), 1.88–1.97 (m, 2H), 1.71–1.77 (m, 4H);

**^13^C-NMR** (101 MHz, CDCl_3_): δ (ppm) 182.0, 142.8, 128.3, 127.1, 110.0, 58.8, 36.0, 23.6;

**HRMS** (EI+): calc. [M]^∙+^ for C_12_H_14_O_2_^∙+^ 190.09883, found 190.09862, Δ = 0.21 mDa.

**Synthesis of** **2-phenylpent-4-enoic acid (S2)**

Synthetized according to **GP4** using phenylacetic acid (2.0 g, 15.0 mmol, 1.0 equiv.) as limiting reagent and allyl bromide (1.1 equiv.) as electrophilic coupling partner. After purification with flash column chromatography (MeOH/DCM, 2.5%) the title compound was obtained as a white solid (1.44 g, 54 %).

**R_f_** (silica, 5% MeOH/DCM) 0.39 [bromocresol green]

**^1^H-NMR** (400 MHz, CDCl_3_): δ (ppm) 11.00 (br.s., 1H), 7.28–7.38 (m, 5H), 5.77 (ddt, *J* = 17.2, 10.3, 6.8 Hz, 1H), 5.13 (dq, *J* = 17.2, 1.6 Hz, 1H), 5.04–5.08 (m, 1H), 3.69 (dd, *J* = 8.8, 7.2 Hz, 1H), 2.83–2.91 (m, 1H), 2.54–2.62 (m, 1H);

**^13^C-NMR** (101 MHz, CDCl_3_): δ (ppm) 179.8, 137.8, 134.9, 128.7, 128.1, 127.6, 117.3, 51.4, 37.1;

**HRMS** (EI+): calc. [M]^∙+^ for C_11_H_12_O_2_^∙+^ 176.08318, found 176.08274, Δ = 0.44 mDa.

**Synthesis of** **2-(thiophen-2-yl)propanoic acid (S3)**

Synthetized according to **GP3** using 2-(thiophen-2-yl)acetic acid (2.84 g, 20.0 mmol, 1.00 equiv.) as starting material. After purification with flash column chromatography (MeOH/DCM, 5%) and drying in high-vacuo, the title compound was obtained as a yellow oil (1.09 g, 18%).

**R_f_** (silica, 5% MeOH/DCM) 0.65 [bromocresol green]

**^1^H-NMR** (400 MHz, CDCl_3_): δ (ppm) 9.16 (br. s., 1H), 7.23 (dd, *J* = 5.1, 1.3 Hz, 1H), 6.99–7.01 (m, 1H), 6.97 (dd, *J* = 5.1, 3.5 Hz, 1H), 4.04 (q., *J* = 7.3 Hz, 1H), 1.62 (d, *J* = 7.3 Hz, 3H);

**^13^C-NMR** (101 MHz, CDCl_3_): δ (ppm) 179.7, 142.0, 126.7, 125.2, 124.6, 40.7, 19.1;

**HRMS** (EI+): calc. [M]^∙+^ for C_7_H_8_O_2_S^∙+^ 156.02395, found 156.02396, Δ = – 0.01 mDa.

## 4.2 Synthesis of sulfonamide nucleophiles

### 4.2.1 General Procedure for the sulfonamide synthesis from sulfonyl chlorides (GP5)

Typically the prepared sulfonamides were synthetized as following (see details at each sulfonamide): The respective sulfonyl chloride (1.00 equiv.) was either dissolved directly into the ammonium hydroxide (25 % solution in water) or into the noted organic solvent under ambient conditions. If not used as a solvent, ammonium hydroxide was added, and the reaction was stirred first at room temperature followed by heating up to 100 °C. The reaction was followed with thin-layer chromatography (TLC) and when full conversion of the starting material was indicated, the reaction was cooled down to room temperature and quenched by adding a 1:1 mixture of water and ethyl acetate. The phases were separated and the aqueous phase extracted with EtOAc (3$\times$), and the combined organic phases were washed with brine. The organic phases were then dried over MgSO_4_, the drying agent filtered off and solvent removed under reduced pressure upon which a white solid was obtained. The purity of the crude product was determined with ^1^H-NMR spectroscopy and if needed the product was purified by passing through a short silica plug eluating with 1:1 EtOAc/PE. Removal of the solvent then gave the final product.

### 4.2.2 List of synthetized sulfonamides

**Synthesis of** **4-trifluoromethylbenzenesulfonamide (2c)**

Synthetized according to a modified literature-reported procedure.^[4]^ 4-Trifluoromethylbenzenesulfonyl chloride (2.46 g, 10.0 mmol, 1.00 equiv.) was dissolved in ammonium hydroxide (25% solution in H_2_O, 4 mL) under ambient conditions. The reaction mixture was stirred overnight at room temperature, after which the reaction was quenched by addition of a mixture of water and ethyl acetate (1:1, 10 mL). The phases were separated, aqueous phase extracted with EtOAc (3 $\times$10 mL) and combined organic layers washed with brine (20 mL). After drying over Mg_2_SO_4_ the drying agent was filtered off and the solvent removed under reduced pressure. The crude product was purified by a short column (EtOAc/PE, 50%) and after removal of solvent the title compound was obtained as a white solid (1.85 g, 82 %).

**R_f_** (silica, 50% EtOAc/PE) 0.73 [potassium permanganate];

**^1^H-NMR** (400 MHz, CDCl_3_): δ (ppm) 8.06 (d, *J* = 8.3 Hz, 2H), 7.98 (d, *J* = 8.3 Hz, 2H), 7.64 (s, 2H);

**^13^C-NMR** (101 MHz, CDCl_3_): δ (ppm) 148.3, 132.18 (q, *J* = 32.2 Hz), 127.1, 126.7 (q, *J* = 3.8 Hz), 124.1 (q, *J* = 274.0 Hz);

**^19^F-NMR** (376 MHz, CDCl_3_, PhCF_3_ as internal standard): δ (ppm) – 63.39 (s)

**HRMS** (ESI+): calc. [M+H]^+^ for C_7_H_7_F_3_NO_2_S^+^ 226.0150, found 226.0145, Δ = 0.5 mDa.

**Synthesis of** **4-cyanobenzenesulfonamide (2d)**

Synthetized according to a modified literature procedure.^[5]^ 4-cyanobenzenesulfonyl chloride (1.5 g, 7.4 mmol, 1.0 equiv.) was dissolved in 25% ammonium hydroxide (10 mL) at room temperature. The mixture was then heated up and refluxed at 100 °C for 1 h. After cooling back to room temperature, the formed white precipitate was filtered, and the crude product was washed with water (3 $\times$ 10 mL). The residual water was removed *via* lyophilization after which the title compound was obtained as an off-white powder (1.0 g, 74%).

**R_f_** (silica, 50% EtOAc/PE) 0.51 [potassium permanganate];

**^1^H-NMR** (400 MHz, CDCl_3_): δ (ppm) 8.07–8.10 (m, 2H), 7.97–8.00 (m, 2H), 7.59 (br. s., 2H), 3.43 (s, 3H);

**^13^C-NMR** (101 MHz, CDCl_3_): δ (ppm) 148.4, 133.7, 126.9, 118.3, 114.8;

**HRMS** (ESI+): calc. [M+H]^+^ for C_7_H_7_N_2_O_2_S^+^ 183.0228, found 183.0221, Δ = 0.7 mDa.

**Synthesis of** **3,5-dichlorobenzenesulfonamide (2f)**

Synthetized according to a literature-reported procedure.^[6]^ 3,5-Dichlorobenzenesulfonyl chloride (1.47 g, 6.00 mmol, 1.00 equiv.) was dissolved in ammonium hydroxide (25% solution in H_2_O, 16 mL) under ambient conditions. The reaction was stirred at room temperature for 3 h, after which it was quenched by addition of a mixture of water and ethyl acetate (1:1, 12 mL). The formed layers were separated, the aqueous layer extracted with EtOAc (3 $\times$10 mL) and combined organic layers washed with brine (20 mL). After drying over Mg_2_SO_4_ the drying agent was filtered off and the solvent removed under reduced pressure. The crude product was purified by a short column (EtOAc/PE, 50%) and after removal of solvent the title compound was obtained as a white solid (305 mg, 23 %).

**R_f_** (silica, 50% EtOAc/PE) 0.83 [potassium permanganate];

**^1^H-NMR** (400 MHz, CDCl_3_): δ (ppm) 7.92–7.94 (m, 1H), 7.80 (d, *J* = 1.9 Hz, 2H), 7.65 (br. s., 2H);

**^13^C-NMR** (101 MHz, CDCl_3_): δ (ppm) 147.5, 135.2, 131.9, 124.8;

**HRMS** (ESI+): calc. [M+H]^+^ for C_6_H_6_Cl_2_NO_2_S^+^ 225.9496, found 225.9491, Δ = 0.5 mDa.

**Synthesis of** **2,4,6-trimethylbenzenesulfonamide (2g)**

Synthetized according to a modified literature-reported procedure.^[4]^ 2,4,6-Trimethoxybenzenesulfonyl chloride (1.09 g, 5.00 mmol, 1.00 equiv.) was dissolved in ammonium hydroxide (25% solution in H_2_O, 25 mL) at 0 °C. After 15 minutes the cooling bath was removed, and the reaction was stirred for 4 hours at room temperature. The reaction was quenched by addition of a mixture of water and ethyl acetate (1:1, 10 mL) and the formed layers were separated. The aqueous layer was acidified with 2M HCl (pH=1) after which it was extracted with EtOAc (2 $\times$ 10 mL). The combined organic layers were washed with brine, dried over Mg_2_SO_4_, the drying agent filtered off and the solvent removed under reduced pressure. The crude product was purified by passing through a short silica plug eluting with 50% EtOAc/PE. After evaporation of the solvent the product was obtained as an off-white solid (659 mg, 66 %).

**R_f_** (silica, 50% EtOAc/PE) 0.75 [potassium permanganate];

**^1^H-NMR** (400 MHz, CDCl_3_): δ (ppm) 7.17 (br. s., 2H), 6.99 (s, 3H), 2.55 (s, 3H);

**^13^C-NMR** (101 MHz, CDCl_3_): δ (ppm) 141.0, 138.7, 137.6, 131.8, 23.0, 20.8;

**HRMS** (ESI+): calc. [M+Na]^+^ for C_9_H_13_NO_2_SNa^+^ 222.0565, found 222.0560, Δ = 0.5 mDa.

**Synthesis of** **naphthalene-1-sulfonamide (2h)**

Synthetized according to a literature-known procedure.^[7]^ Napthalene-1-sulfonyl chloride (904 mg, 4.00 mmol, 1.00 equiv.) was dissolved in THF (13 mL). Ammonium hydroxide (25% solution in H_2_O, 8.7 mL) was added slowly to the mixture under ambient conditions, after which the reaction was stirred for three hours at room temperature. THF was then removed under reduced pressure, and the crude was dissolved in a mixture of EtOAc and H_2_O. The layers were then separated and the aqueous layer was extracted with EtOAc (3 $\times$ 10 mL). The combined organic layers were dried over Mg_2_SO_4_, the drying agent filtered off and the solvent removed under reduced pressure. The formed pale solid was washed with hexane and Et_2_O and after drying under high vacuo the title compound was obtained as a white solid.

**R_f_** (silica, 50% EtOAc/PE) 0.58 [potassium permanganate];

**^1^H-NMR** (400 MHz, CDCl_3_): δ (ppm) 8.44 (d, *J* = 1.5 Hz, 2H), 8.11–8.16 (m, 2H), 8.03–8.06 (m, 1H), 7.90 (dd, *J* = 8.7, 1.8 Hz, 1H), 7.65–7.72 (m, 2H), 7.46 (br. s., 2H);

**^13^C-NMR** (101 MHz, CDCl_3_): δ (ppm) 141.7, 134.3, 132.2, 129.5 (2C), 128.9, 128.3, 127.9, 126.2, 122.6;

**HRMS** (ESI+): calc. [M+Na]^+^ for C_10_H_9_N_2_O_2_SNa^+^ 244.0282, found 244.0245, Δ = 3.7 mDa.

**Synthesis of** **methyl 4-sulfamoylbenzoate (2i)**

Synthetized according to a modified literature-reported procedure.^[8]^ 4-Sulfamoylbenzoic acid (2.00 g, 9.94 mmol, 1.00 equiv.) was dissolved in MeOH (18 mL) followed by the addition of conc. H_2_SO_4_ (0.5 mL). The reaction was then refluxed for 7 h, after which it was cooled down to room temperature. Methanol was removed under reduced pressure, and the residue dissolved in CHCl_3_. The organic layer was then washed with H_2_O, aq. sat. Na_2_CO_3_ solution and finally again with H_2_O. The organic phase was dried over Mg_2_SO_4_, the drying agent filtered off and solvent removed under reduced pressure. Purification with flash column chromatography (EtOAc/PE, 20% → 80%) gave the title compound as a white solid (1.6 g, 75%).

**R_f_** (silica, 50% EtOAc/PE) 0.54 [potassium permanganate];

**^1^H-NMR** (400 MHz, CDCl_3_): δ (ppm) 8.12–8.15 (m, 2H), 7.94–7.98 (m, 2H), 7.56 (s, 2H), 3.89 (s, 3H);

**^13^C-NMR** (101 MHz, CDCl_3_): δ (ppm) 165.7, 148.5, 132.8, 130.3, 126.5, 53.0;

**HRMS** (ESI+): calc. [M+H]^+^ for C_8_H_10_NO_4_S^+^ 216.0331, found 216.0324, Δ = 0.7 mDa.

**Synthesis of** **4’fluoro-[1,1’-biphenyl]-4-sulfonamide (2k)**

Synthetized according to a modified patented procedure.^[9]^ 4’fluoro-[1,1’-biphenyl]-4-sulfonyl chloride (1.0 g, 3.7 mmol, 1.0 equiv.) was dissolved in 1,4-dioxane (10 mL) at room temperature. Ammonium hydroxide (25% solution in H_2_O, 5.8 mL, 37.0 mmol, 10.0 equiv.) was added and the reaction was stirred for 1 h under ambient conditions. The reaction was quenched by adding a mixture of water and hexane (1:1, 10 mL) followed by solid NaCl. The mixture was further stirred for 30 minutes, after which the formed white precipitate was filtered and the washed with H_2_O (3 $\times$ 10 mL). The residual water was then removed *via* lyophilization after which the title compound was obtained as an off-white solid (678 mg, 73%).

**R_f_** (silica, 50% EtOAc/PE) 0.63 [potassium permanganate];

**^1^H-NMR** (400 MHz, CDCl_3_): δ (ppm) 7.84–7.93 (m, 4H), 7.77–7.82 (m, 2H), 7.42 (br. s., 2H), 7.32–7.38 (m, 2H),

**^13^C-NMR** (101 MHz, CDCl_3_): δ (ppm) 162.9 (d, *J* = 247 Hz), 143.1 (d, *J* = 64 Hz), 135.6 (d, *J* = 3.1 Hz), 129.7, 129.6, 127.6, 126.8, 116.4 (d, *J* = 21.2 Hz);

**^19^F-NMR** (376 MHz, CDCl_3_, PhCF_3_ as internal standard): δ (ppm) –116.71 – (–)116.79 (m)

**HRMS** (EI+): (ESI+): calc. [M+Na]^+^ for C_12_H_10_FNO_2_SNa^+^ 274.0314, found 274.0310, Δ = 0.4 mDa.

**Synthesis of *N*-phenylcyclopropanesulfonamide (2m)**

Synthetized according to a modified literature procedure.^[10]^ Cyclopropanesulfonyl chloride (1.0 mL, 9.90 mmol, 1.0 equiv.) was dissolved to a mixture of pyridine (2 mL) and DCM (2 mL). Aniline (0.9 mL, 9.90 mmol, 1.0 equiv.) was added to the mixture under ambient conditions, after which the reaction was heated to reflux at 50 °C overnight. After cooling down to room temperature, the mixture was diluted with Et_2_O (20 mL) and the organic layer was washed with water (2 $\times$ 20 mL) and brine (20 mL).

The organic layer was dried over Mg_2_SO_4_, the drying agent filtered off and the organic layer concentrated under reduced pressure. The crude product was purified with flash column chromatography (EtOAc/PE) and after removal of solvents the title compound was obtained as a white solid.

**R_f_** (silica, 50% EtOAc/PE) 0.87 [potassium permanganate];

**^1^H-NMR** (400 MHz, CDCl_3_): δ (ppm) 7.32–7.38 (m, 2H), 7.26–7.30 (m, 2H), 7.17–7.22 (m, 1H), 6.77 (br. s., 1H), 2.46–2.54 (m, 1H), 1.15–1.21 (m, 2H), 0.93–0.99 (m, 2H);

**^13^C-NMR** (101 MHz, CDCl_3_): δ (ppm) 136.8, 129.5, 125.5, 121.8, 29.8, 5.61;

**HRMS** (ESI+): calc. [M+H]^+^ for C_9_H_12_NO_2_S^+^ 198.0589, found 198.0583, Δ = 0.6 mDa.

**Synthesis of** ***N*-phenylcyclopentanesulfonamide (2n)**

Synthetized according to a modified literature procedure.^[10]^ Cyclohexanesulfonyl chloride (1.5 g, 8.20 mmol, 1.0 equiv.) was dissolved to a mixture of pyridine (2 mL) and DCM (2 mL). Aniline (0.84 mL, 9.20 mmol, 1.1 equiv.) was added to the mixture under ambient conditions, after which the reaction was heated to reflux at 50 °C overnight. After cooling down to room temperature, the mixture was diluted with Et_2_O (20 mL) and the organic layer was washed with water (2 $\times$ 20 mL) and brine (20 mL). The organic layer was dried over Mg_2_SO_4_, the drying agent filtered off and the organic layer concentrated under reduced pressure. The crude product was purified with flash column chromatography (EtOAc/PE) and after removal of solvents the title compound was obtained as a pale yellow solid (1.51 g, 77 %).

**R_f_** (silica, 50% EtOAc/PE) 0.74 [potassium permanganate];

**^1^H-NMR** (400 MHz, CDCl_3_): δ (ppm) 7.32–7.37 (m, 2H), 7.25–7.29 (m, 1H), 7.11–7.18 (m, 2H), 3.04 (tt, *J* = 12.1, 3.5 Hz, 1H), 2.19 (d, *J* = 13.0 Hz, 2H), 1.84–1.91 (m, 2H), 1.54–1.70 (m, 3H), 1.14–1.29 (m, 3H);

**^13^C-NMR** (101 MHz, CDCl_3_): δ (ppm) 137.4, 129.6, 124.6, 120.0, 60.4, 26.3, 25.1, 25.0;

**HRMS** (ESI+): calc. [M+Na]^+^ for C_12_H_17_NO_2_SNa 262.0878, found 262.0874, Δ = 0.4 mDa.

**Synthesis of** **methyl 2,3-dimethoxy-5-sulfamoylbenzoate (2o)**

Synthetized according to a literature-reported procedure.^[8]^ 5-(Aminosulfonyl)-2,3-dimethoxybenzoic acid (5.1 g, 20 mmol, 1.00 equiv.) MeOH (15 mL) followed by the addition of conc. H_2_SO_4_ (0.3 mL). The reaction was then refluxed for 7 h, after which it was cooled down to room temperature. Methanol was removed under reduced pressure, and the residue dissolved in CHCl_3_. The organic layer was then washed with H_2_O, aq. sat. Na_2_CO_3_ solution and finally again with H_2_O. The organic phase was dried over Mg_2_SO_4_, the drying agent filtered off and solvent removed under reduced pressure. Recrystallization from a mixture of MeOH and H_2_O yielded the final product as a white solid (1.58 g, 29% yield over three steps).

**R_f_** (silica, 50% EtOAc/PE) 0.36 [potassium permanganate];

**^1^H-NMR** (400 MHz, CDCl_3_): δ (ppm) 7.66 (d, *J* = 16.6, 2.2 Hz, 2H), 7.42 (br. s., 2H), 3.92 (s, 3H), 3.87 (s, 3H), 3.83 (s, 3H);

**^13^C-NMR** (101 MHz, CDCl_3_): δ (ppm) 165.5, 153.7, 150.9, 140.1, 126.1, 119.3, 113.3, 61.7, 56.9, 53.0;

**HRMS** (ESI+): calc. [M+H]^+^ for C_10_H_14_NO_6_S^+^ 276.0542, found 276.0535, Δ = 0.7 mDa.

### 4.2.3 List of synthetized unsuccessful sulfonamides

**Synthesis of 4-nitrobenzenesulfonamide (2q)**

Synthetized according to a modified literature-reported procedure.^[4]^ 4-Nitrobenzenesulfonyl chloride (1.04 g, 5.00 mmol, 1.00 equiv.) was dissolved in ammonium hydroxide (25% solution in H_2_O, 25 mL) at 0 °C. After 15 minutes the cooling bath was removed, and the reaction was stirred for 4 hours at room temperature. The reaction was quenched by addition of a mixture of water and ethyl acetate (1:1, 10 mL) and the formed layers were separated. The aqueous layer was acidified with 2M HCl (pH=1) after which it was extracted with EtOAc (2 $\times$ 10 mL). The combined organic layers were washed with brine, dried over Mg_2_SO_4_, the drying agent filtered off and the solvent removed under reduced pressure. The formed white crude product (which was not adequately pure based on ^1^H NMR analysis) was then passed through a short silica plug (EtOAc/PE, 50%) to remove the formed sulfonic acid. After evaporation of the solvent the product was obtained as a white solid (315 mg, 31 %).

**R_f_** (silica, 50% EtOAc/PE) 0.60 [potassium permanganate];

**^1^H-NMR** (400 MHz, CDCl_3_): δ (ppm) 8.39–8.43 (m, 2H), 8.05–8.09 (m, 2H), 7.73 (br. s., 2H);

**^13^C-NMR** (101 MHz, CDCl_3_): δ (ppm) 149.9, 149.7, 127.7, 124.9;

**HRMS** (ESI+): calc. [M+H]^+^ for C_6_H_7_N_2_O_4_S^+^ 203.0127, found 203.0120, Δ = 0.7 mDa.

# 5. Mechanistic studies

## 5.1 UV-vis measurements

### 5.1.1 Uv-vis in Acetonitrile

All UV-vis measurements in MeCN were carried out in a 0.1 cm $\times$ 1.0 cm quartz cuvette with 0.1 cm light path and an internal volume of about 0.2 mL. The spectra were recorded with Agilent Cary 4000 UV-vis spectrophotometer scanning the spectral window from 900 nm to 200 nm using 11 nm data intervals. The temperature was hold constant at 25.6 °C during the measurements with water-cooling running through the probe chamber. The samples were prepared as following: Bi(OTf)_3_ (13.0 mg, 0.2 mmol) was set under an inert atmosphere through evacuation and back-filling with N_2_ (3$\times$). Dry MeCN (1 mL) was added, and the formed stock solution was shaken to ensure even distribution of components. Then, 0.25 mL of this stock solution was transferred to another vial under N_2_, and the vial was filled up to a final volume of 1.0 mL with dry MeCN. 0.2 mL from the final solution was then transferred to the afore described quartz cuvette for measurements. With respect to the other components, 2-phenylpropanoic acid (2.75 μL, 0.05 mmol) or 4-methylsulfonamide (3.0 mg, 0.05 mmol) were both dissolved into dry MeCN (1 mL) in separate vials under N_2_ to form the samples of appropriate concentrations. Other components, KHCO_3_ (5 mg) and H_2_O (5 μL), were then added stepwise to the 1 mL sample volumes, shaken, and 0.2 mL transferred again *via* syringe to the cuvette. For the measurement of the complex, a stock solution containing 0.2 mmol of Bi(OTf)_3_, 2-phenylpropanoic acid **1a** and 4-methylsulfonamide **2a** in MeCN (1 mL) was first formed followed by 1/4 dilution as described for the pure bismuth triflate. Background spectrum of pure MeCN was measured first and subtracted from other spectra.

|  | **Figure S6:** UV-vis spectrum of the reaction components: Bi(OTf)_3_ as a gray dotted line, 2-phenylpropanoic acid (**1a**) as an orange line, 4-methylphenylsulfonamide (**2a**) as a blue line and the combination of the above as a yellow line. All components measured in 0.05 mmol dissolved in 0.5 mL of MeCN. Samples of acid **1a**, sulfonamide **2a** and complex also containing KHCO_3_ (5 mg) as a base and H_2_O (5 μL). |  |
| --- | --- | --- |

Due to the well-soluble nature of Bi(OTf)_3_ and other reaction components in MeCN, relatively concentrated samples could be prepared. It should be noted that polar protic solvents, such as MeCN, are known to coordinate to metal centers, and therefore the existence of acetonitrile ligands in the measured bismuth compounds cannot be ruled out. However, to enhance the coordination of the desired ligands (carboxylic acids and sulfonamides), concentrated solutions were prepared for the UV-vis studies. As can be seen from Figure S6, Bi(OTf)_3_ has its broad absorption maximum around 230 nm and tails until 280 nm. The 2-phenylpropanoic acid (**1a**) and 4-methylphenylsulfonamide (**2a**) both have a more complex absorption spectra centered around 260 nm and tailing towards 300 nm. When Bi(OTf)_3_ is added to the mixture containing both components, a significant enhancement in the absorbance is observed around 260 nm, with the absorption of the complex tailing to longer wavelengths than that of either components alone.

### 5.1.2 Uv-vis in DCM

To obtain a better representation of the species present in the reaction mixtures, we changed the solvent to DCM. Unlike in MeCN, the bismuth species have only a limited solubility in the medium. For this reason, a notably more diluted solutions were measured combined with an increased light path length. For the measurements in DCM, a 1.0 cm $\times$ 1.0 cm quartz cuvette was utilized. Stock solutions of Bi(OTf)_3_, carboxylic acid **1a** and sulfonamide **2a** were all prepared separately in 0.1 M concentration in DCM, followed by dilution with DCM to the final concentration of 0.002 M. The bismuth complex was prepared by mixing all three components in a 1:1:1 ratio (0.1 M each in DCM) followed by dilution to 0.002 M. The spectra were recorded with Agilent Cary 4000 UV-vis spectrophotometer scanning the spectral window from 900 nm to 200 nm using 11 nm data intervals. The temperature was hold constant at 25.6 °C during the measurements with water-cooling running through the probe chamber. The results can be seen in Figure S7.

|  | **Figure S7:** UV-vis spectrum of the reaction components in DCM: Bi(OTf)_3_ as a gray dotted line, 2-phenylpropanoic acid (**1a**) as an orange line, 4-methylphenylsulfonamide (**2a**) as a blue line and the combination of the above as a yellow line. All components measured in 0.02 mmol concentrations. |  |
| --- | --- | --- |

As seen from Figure S7, the absorption spectra of each component in DCM closely resembles the respective spectra measured in MeCN both in the terms of the shape of the absorption band as well as the location of the absorption maxima. Here as well the addition of Bi(OTf)_3_ to the mixture of acid **1a** and sulfonamide **2a** causes a significant enhancement of absorption at the area covering the absorption maximum. In this case, however, the exact form of the absorption band slightly differs from that seen in MeCN perhaps due to the lack of the solvent ligands in DCM.

To gain a more precise look to the absorption spectrum around the irradiation wavelength (365 nm), we decided to measure the previously formed bismuth complex (1:1:1 of acid **1a**, sulfonamide **2a** and Bi(OTf)_3_, 0.002 M for each in DCM) with a 4 cm $\times$ 2 cm quartz cuvette (pathlength 4 cm, sample volume 6 mL). The measurement was carried out with Agilent Cary 60 UV-vis spectrophotometer at 25 °C in the scanning window from 1100 nm to 190 nm using the data interval of 0.5 nm. The result of this measurement can be seen in Figure S8.

|  | **Figure S8:** UV-vis spectrum of the bismuth complex containing Bi(OTf)_3_, 2-phenylpropanoic acid (**1a**) and 4-methylphenylsulfonamide (**2a**) as 0.02 mmol concentration each in DCM. To gain a better look to the absorbance around the irradiation wavelength (365 nm), a 4.0 cm quartz cuvette was utilized. |  |
| --- | --- | --- |

Comparing the UV-vis spectrum obtained using the 4 cm pathlength to the one obtained with 1 cm pathlength gratifyingly shows significantly better-defined spectral region above 300 nm. Importantly, slight tailing of the UV-vis absorption of the complex can be easily observed starting around 360 nm which intersects the emission spectrum of the LEDs utilized (see section 1.1). Moreover, the low absorption coefficient around the irradiation wavelength in part accounts for the relatively high intensity needed for the product formation (Table S6). Although the exact intensity of the absorption around 365 nm is naturally dependent on the concentration of the complex, the UV-vis spectrum above demonstrates clearly how the absorption tails to the critical regions.

## 5.2 Kinetic studies

For the kinetic studies, one reaction vial was prepared as following: ($\pm$)-2-phenylpropanoic acid (15.1 mg, 0.10 mmol, 1.0 equiv.), KHCO_3_ (30 mg, 0.30 mmol, 3.0 equiv.) and *p*-toluenesulfonamide (59 mg, 0.35 mmol, 3.5 equiv.) were weighed into a 5 mL crimp cap vial. The vial was then sealed and set under an inert atmosphere by evacuating and backfilling with pre-dried N_2_ ($\times$3). Anhydrous DCM (1 mL) was added, and the reaction mixture was degassed by freeze-pump thaw ($\times$3). Then reaction was then irradiated with 365 nm (1.5 W) LEDs.

To obtaining each data point, a 25 μL sample was drawn from the reaction mixture, to which 250 μL of the stock solution containing internal standard was added (0.01 M mesitylene in DCM). Finally, the samples were analyzed by GC-FID and the yield were calculated against the calibration curve of the product and mesitylene.

### 5.2.1 Reaction kinetics

To obtain an overall picture of the reaction kinetics, the rate of the reaction was first monitored for the first four hours, the result of which mostly resembles a logarithmic reaction kinetic (Figure S9). A notable feature of this reaction is indeed its rapid initial kinetics, which results in a 28 % yield during the first hour of the reaction. It thus seems like the turbidness of the reaction mixture does not prevent the adequate amounts of light from entering the reaction mixture during the first hour of the reaction time.

| **Time** (min) | **Yield** (%) |
| --- | --- |
| 0 | 0 |
| 2.5 | 3.4 |
| 5 | 5.4 |
| 10 | 9.5 |
| 15 | 13 |
| 20 | 16 |
| 30 | 20 |
| 40 | 23 |
| 60 | 28 |
| 120 | 37 |
| 240 | 54 |

**Figure S9:** Kinetic profile of the reaction during the first four hours.

As a part of the observation of the fast initial kinetic is also the significant reduction of the product forming rate after the first hour. This decrease in the productive reaction can in to at least some extend be hypothesized to come from the formation of fully reduced Bi^(0)^ (also referred to as “bismuth black”) which results into a significant darkening of the reaction mixture therefore diminishing the light penetration (see section 1.1.4).

### 5.2.2 Initial kinetics

To gain a deeper look to the initial kinetics of the reaction, the rate measurement was repeated during the first twenty minutes (Figure S10). During this time period the reaction could be said to follow a linear reaction rate although slight features of logarithmic rate could also be argued to appear. The single points obtained from each time point could also be joined with a direct line with very high experimental correlation coefficient.

| **Time** (min) | **Yield** (%) |
| --- | --- |
| 0 | 0 |
| 2.5 | 3.4 |
| 5 | 5.4 |
| 10 | 9.5 |
| 15 | 13 |
| 20 | 16 |

**Figure S10:** Initial reaction kinetics.

Strikingly, small amounts of product can be observed already after 2.5 minutes of irradiation time implying that the reaction starts (almost) immediately when the light is turned on. Moreover, the linear kinetic is often expected in the cases where the product is directly formed from the reactants without the need to build up any intermediates or to activate a pre-catalytic species. It is worth mentioning that a linear kinetics was also observed in our previous work with the BiCl_3_-catalyzed Giese-coupling reactions^[12]^, which further supports the use of simple bismuth salts as efficient catalytic species requiring light as the sole source of activation.

### 5.2.3 Light on/off experiment

Finally, we concluded the kinetic experiments by carrying out a light on/off- experiment. Due to the turbid nature of our initial reaction mixture, we considered quantum yield measurement to give results that would be uncertain or difficult to interpret. Therefore, we decided to monitor the product formation by irradiating the reaction mixture 10 or 20 minutes followed by a ten-minute dark period. Results of this experiment can be seen in Figure S11.


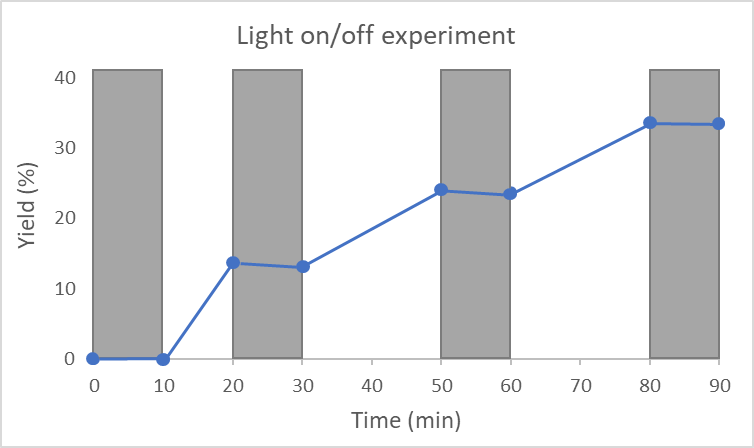


**Figure S11:** Light on/off experiment measuring the rate of product formation.

As can be seen from Figure S11, the reaction clearly progresses only when the light is on. This in turn implies that the reaction is dependent on continuous irradiation and a radical chain, if present at all, is short and does not serve as the main product-forming mechanism. Gratifyingly, the formed product also appears to be rather stable during the dark phases and no significant degradation was observed when the light was off.

## 5.3 Experiments towards later elementary steps

### 5.3.1 TEMPO Trapping Studies

After establishing the nature of the light-harvesting species, we next turned our attention towards identification of some of the reaction intermediates. We started by carrying out a TEMPO-trapping experiment, which was set up as following: 2-phenylpropanoic acid (15.0 mg, 0.10 mmol, 1.00 equiv.), Bi(OTf)_3_ (130 mg, 0.20 mmol, 2.00 equiv.), KHCO_3_ (30.0 mg, 0.30 mmol, 3.00 equiv) and TEMPO (32.0 mg, 0.2 mmol, 2.00 equiv.) were weighed into a 5 mL crimp-cap vial equipped with a stirring bar. The vial was then sealed and set under an inert atmosphere by evacuating and back-filling with N_2_ for three times. DCM (1 mL) was added *via* syringe against a flow of N_2_, and the reaction mixture was further degassed by freeze-pump thaw (3$\times$). The reaction was then irradiated with 365 nm LEDs for 18 hours while keeping the temperature at 25 °C by water-cooling.

After completion of the reaction, the solids were removed by filtering with a syringe filter. The so-obtained crude reaction mixture was then analyzed by LC-MS.

**Scheme S1:** TEMPO-trapping experiment.

As can be seen from Scheme S1, the TEMPO-trapping study was able to identify the expected benzylic radical formed from the radical decarboxylation. The found *m/z* of this trapped intermediate was in good agreement with the expected mass of the [M+H]^+^ ion. Importantly, this was the only product formed during the reaction, and no traces of the radical homocoupling or Kharasch products were observed.

### 5.3.2 Studies towards the presence/absence of benzylic carbocations

To gather indirect evidence on the presence or absence of a benzylic carbocation, we decided to turn towards testing different nucleophiles in our reaction. We rationalized that in the case of a “free” carbocation intermediate, nucleophilic coupling with various *N*-and *O*-centred nucleophiles should be feasible. For this, we set up the reactions according to the **GP1** but instead of 4-methylbenzenesulfonamide we tested a benzylamide (**6a**), selected primary amines **6b** and pyrazole **6c** as *N*-centred nucleophiles. For the *O*-centred nucleophiles, different alcohols were tried (**6d**–**6e**) together with the homocoupling to the 2-phenylpropanoic acid (**6f**) to form the benzylic ester (Kharasch product). The results are summarized in Scheme S2.

**Scheme S2:** List of unsuccessful nucleophiles.

Upon changing the sulfonamide nucleophiles other *N*-centred nucleophiles we observed clear suppression on the LMCT reactivity. Similar results were also obtained with alcohols as *O*-centred nucleophiles. While this hampering of reactivity might stem from numerous reasons, we believe that factors worth considering include: 1) full saturation of the bismuth centre by tight coordination of alternative nucleophiles, thus leaving no room for the coordination of carboxylic acids, 2) diminishing from the UV-vis absorption properties *via* changing the ligands, and 3) the inability of other nucleophiles to undergo C–N coupling because no carbocation intermediates are present and the reductive elimination strongly favours the sulfonamides. While many of the afore-mentioned factors might explain the lack of reactivity with other nucleophiles, we were also unable to observe the Kharasch product stemming from nucleophilic attack of the 2-phenylpropanoic acid – an event likely to happen in the absence of other reactants. This, together with the absence of desaturation products formed by *alpha* deprotonation of the carbocation, indicate that a carbocationic intermediate is most likely not present. It also should be noted that successful CN coupling was observed with MeCN which also can coordinate to bismuth although in a reversible manner.

### 5.3.3 BiCl_3_ as bismuth precatalyst

To differentiate between the possible outer-sphere mechanisms (radical ligand transfer *vs.* radical oxidation), we set up further reactions utilizing BiCl_3_ as bismuth precursor (Scheme S3). If our reaction would follow the radical ligand transfer, a benzyl chloride **8** would be formed as an intermediate followed by nucleophilic substitution by the sulfonamides. When BiCl_3_ was used for the decarboxylation of the acid **1a**, the reaction yielded a low concentration of the radical homocoupling product while C–N coupling product **3a** could not be detected (Scheme 3a). We then attempted to generate the intermediate **8** *via* a HAT reaction from ethylbenzene with over-stoichiometric amount of chlorine source (Scheme 3b). However, the desired product could not be observed. Taken together, these results suggest that the radical ligand transfer, as suggested with iron chloride, did not represent the key mechanistic pathway in our reaction.

**Scheme S3:** Mechanistic studies using bismuth(III)chlorides in LMCT decarboxylation (a) and HAT coupling (b).

## 5.4 Reactions using substoichiometric bismuth

We set out to study the effects of using bismuth as a limiting reagent in our reaction to gain further insight into the efficiency of bismuth in our reaction. The reactions were set up following **GP1**, though lowered bismuth loading was used as described in Table S10.

**Table S10:** Full optimization of bismuth triflate loading.

| **Entry** | **Bi Loading** | **Yield** (based on acid, %) | **Yield** (based on bismuth, %) |
| --- | --- | --- | --- |
| 1 | 0.33 equiv. | 31 | 0.94 |
| 2 | 0.66 equiv. | 45 | 0.68 |

|  | Reaction conditions: Bi(OTf)_3_ (n equiv.), 2-phenylpropanoic acid (15.0 mg, 0.1 mmol, 1.0 equiv.), KHCO_3_ (30.0 mg, 0.3 mmol, 3.0 equiv.) and 4-methylphenylsulfonamide (65.5 mg, 0.35 mmol, 3.5 equiv.) were dissolved into DCM (1 mL) under N_2_. After irradiation with 365 nm for 10 h at 25 °C, internal standard was added and reactions analyzed with GC-FID. |  |
| --- | --- | --- |

With 0.33 equivalents of bismuth, the yield of 94% yield was obtained (Entry 1). This result suggests a high activity of bismuth, serving as an efficient subsequent one electron oxidizing agent. Moreover, this result can also be seen as supporting our mechanistic hypothesis that the bismuth centre participating the radical generation further traps the substrate radical resulting into the product formation. When 0.66 equivalents of bismuth was used, 68% yield was obtained (Entry 2).

# References

[1] F. Mandrelli, A. Blond, T. James, H. Kim, B. List, *Angew. Chem. Int. Ed.*, **2019**, *58*, 11479–11482.

[2] S. Arseniyadis, K. S. Kyler and D. S. Watt, *Organic Reactions*, **2005**, [doi.org/10.1002/ 0471264180.or031.01](https://doi.org/10.1002/0471264180.or031.01)

[3] H. Chi, H. Li, B. Liu, R. Ye, H. Wang, Y.-L. Guo, Q. Tan, B. Xu, *iScience*, **2019**, *21*, 650–663.

[4] Y. Zheng, J. Mao, J. Chen, G. Rong, D. Liu, H. Yan, Y. Chi and X. Xu, *RSC Adv.*, **2015**, *5*, 50113–50117.

[5] J. M. Curle, M. C. Perieteanu, P. G. Humphreys, A. R. Kennedy and N. C. O. Tomkinson, *Org. Lett.*, **2020**, *22*, 1659–1664.

[6] K. Namba, X. Zheng, K. Motoshima, H. Kobayashi, A. Tai, E. Takahashi, K. Sasaki, K. Okamoto and H. Kakuta, *Bioorg. Med. Chem.*, **2008**, *16*, 6131–6144.

[7] M. J. Tilby, D. F. Dewez, L. R. E. Pantaine, A. Hall, C. Martínez-Lamenca and M. C. Willis, *ACS Catal.*, **2022**, *12*, 6060–6067.

[8] C. Bhushan Mishra, S. Kumari, A. Angeli, S. Bua, M. Buonanno, S. M. Monti, M. Tiwari and C. T. Supuran, *Eur. J. Med. Chem.*, **2018**, *156*, 430–443.

[9] A. Glaettli, W. Grammenos, B. Mueller, J. K. Lohmann and M. Vrettou, BASF, Preparation of heterocyclo-fused pyridin-4-ylmethylsulfonamides as agrochemical fungicides, World Intellectual Property Organization, WO 2009/144159 A1 (published 03. December 2009).

[10] M. D. Bartberger, H. P. Beck, X. Chen, R. V. Connors, J. Deignan, J. A. Duquette, J. Eksterowicz, B. M. Fox, J. Fu, A. Gonzalez Buenrostro, F. Gonzales Lopez de Turiso, D. J. Gustin, J. A. Heath, M. G. Johnson, F. Kayser, D. J. Kopecky, Y. Li, Z. Li, Z. Ma, J. McIntosh, J. C. Medina, J. T. Mihalic, S. H. Olson, Y. Rew, P. M. Roveto, M. J. Schmitt, D. Sun, X. Wang, M. Wanska, C. Weathers and X. Yan, Amgen Inc., Preparation of heterocyclic compounds, especially substituted morpholinones, as MDM2 inhibitors for the treatment of cancer, World Intellectual Property Organization, WO 2013/049250 A1 (published 04. April 2013).

[11] I. L. Conlon, B. Drennen, M. E. Lanning, S. Hughes, R. Rothhaas, P. T. Wilder, A. D. MacKerell Jr. and S. Fletcher, *ChemMedChem*, **2020**, *15*, 1691–1698.

[12] D. Birnthaler, R. Narobe, E. Lopez-Berguno, C. Haag and B. König, *ACS Catal.*, **2023**, *13*, 1125–1132.

# NMR-Spectra of the isolated products


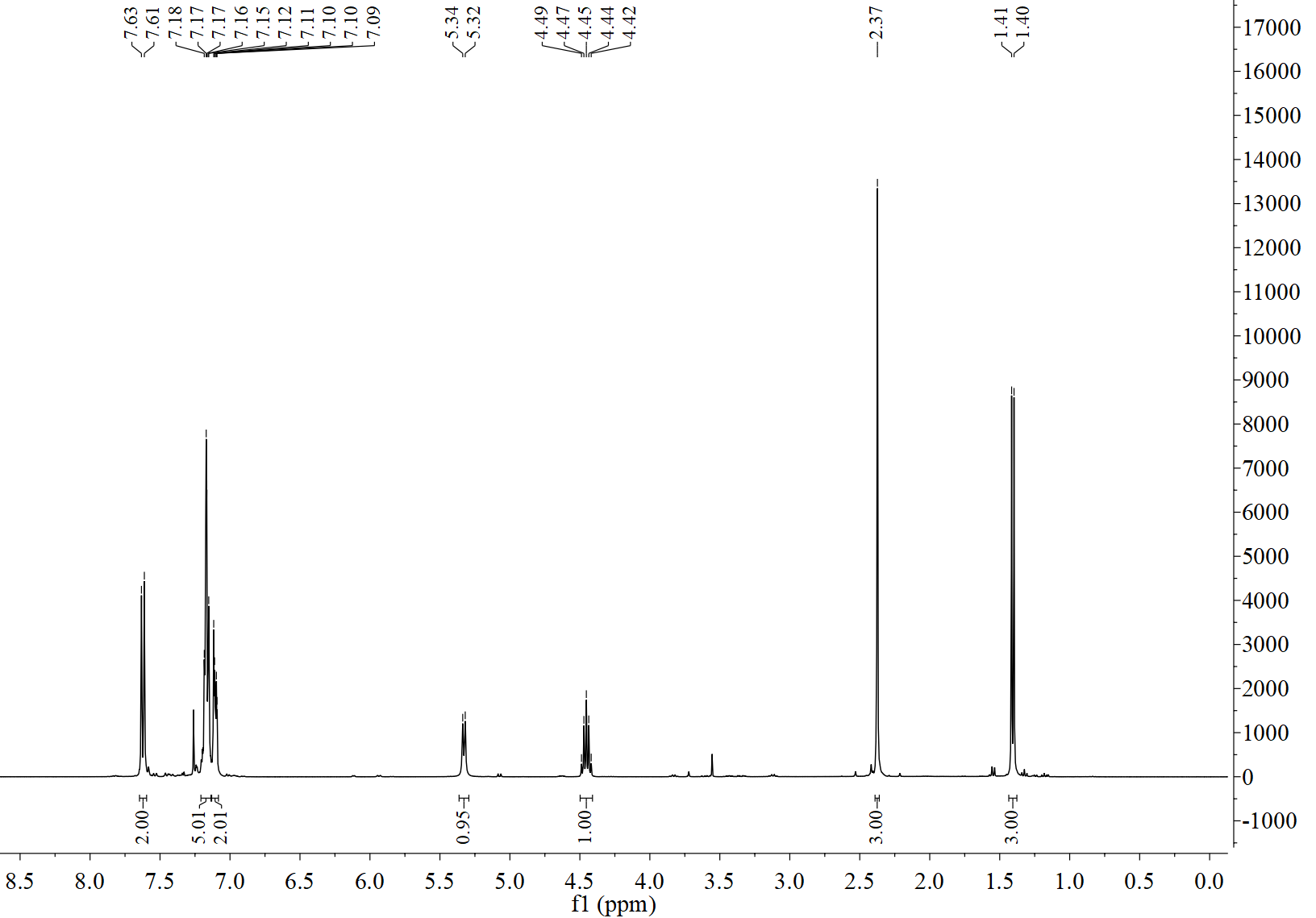


**Figure S12:** ^1^H-NMR (400 MHz, CDCl_3_) of 4-methyl-*N*-(1-phenylethyl)benzenesulfonamide (**3a**).


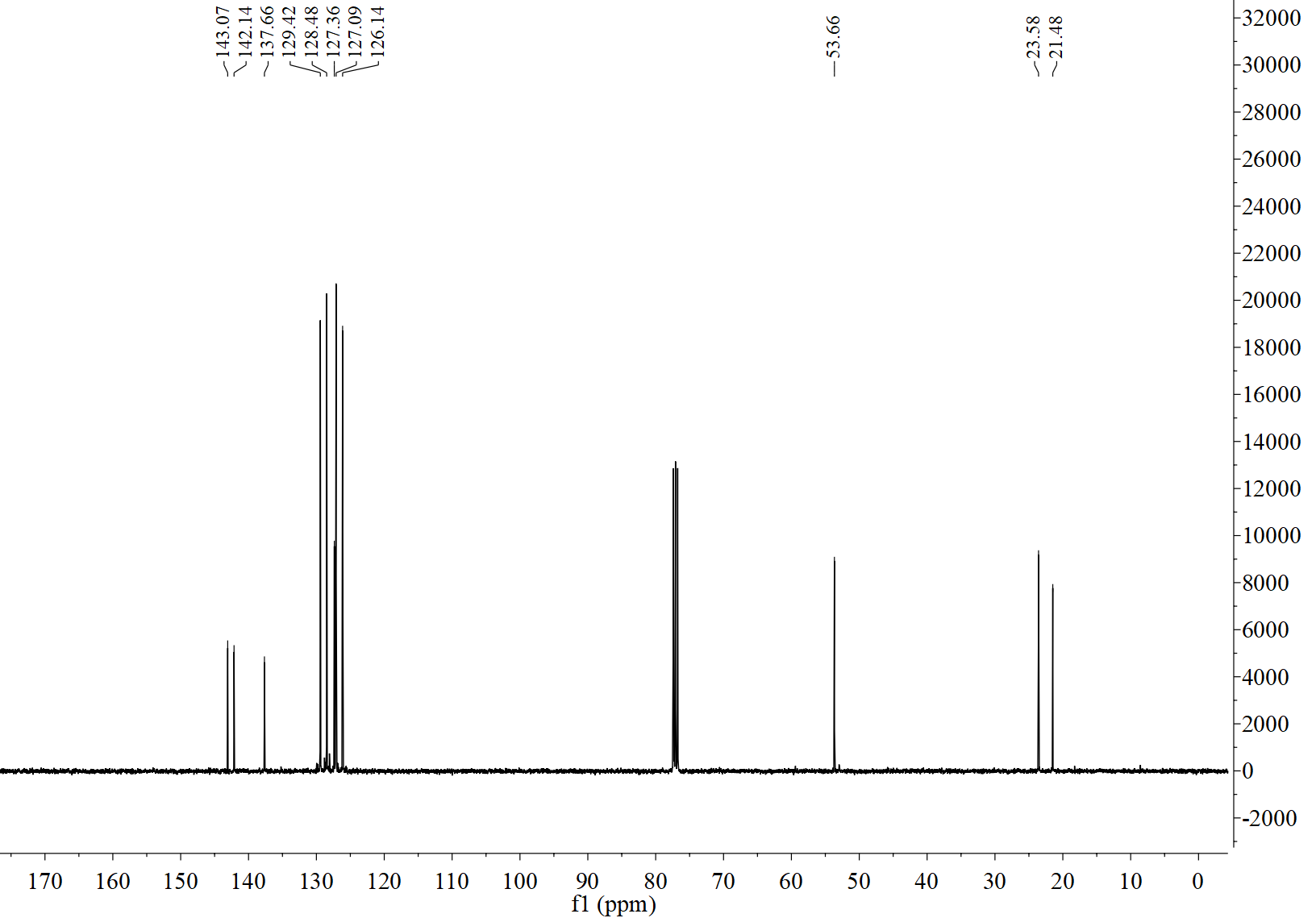


**Figure S13:** ^13^C-NMR (101 MHz, CDCl_3_) of 4-methyl-*N*-(1-phenylethyl)benzenesulfonamide (**3a**).


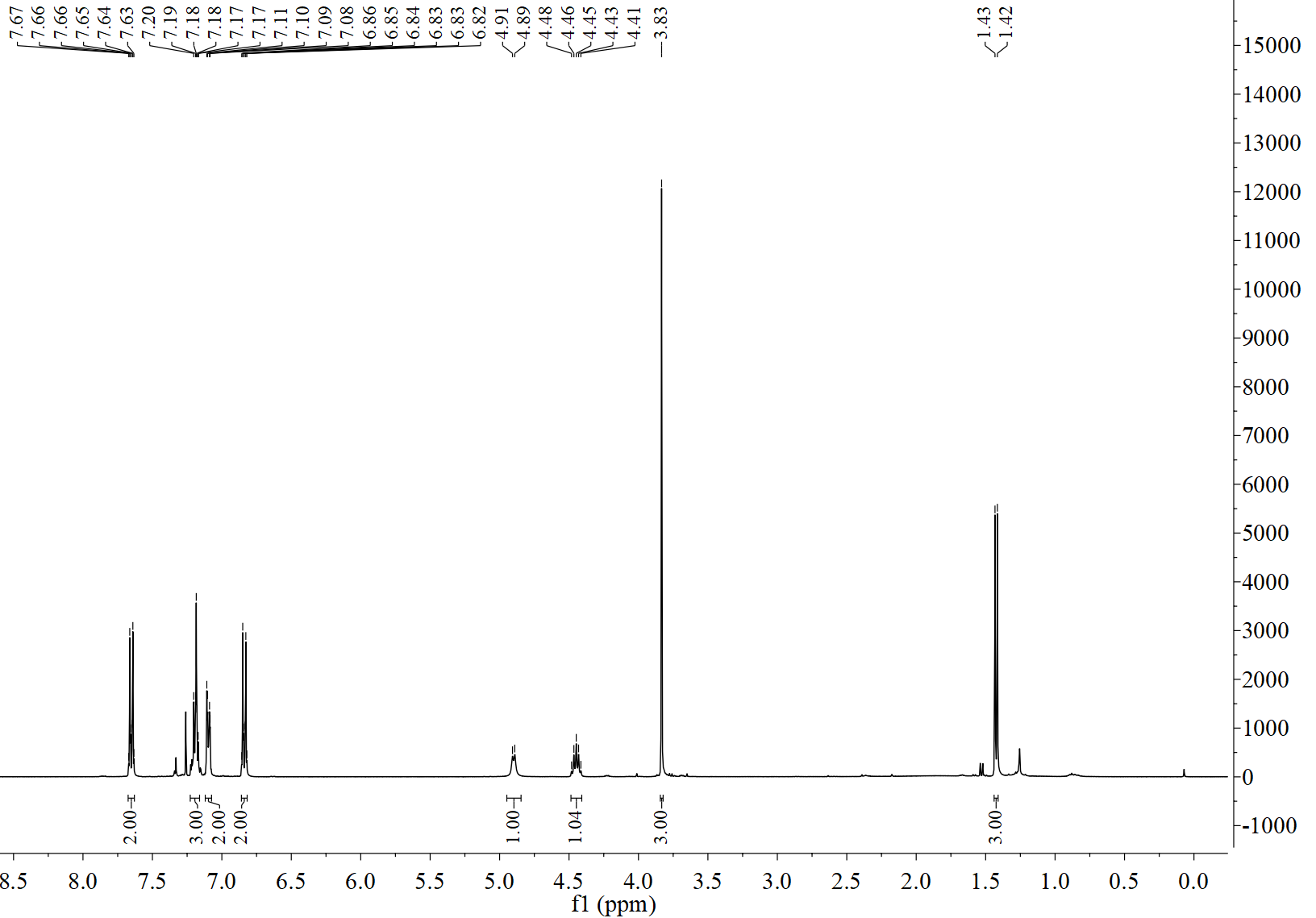


**Figure S14:** ^1^H-NMR (400 MHz, CDCl_3_) of 4-methoxy-*N*-(1-phenylethyl)benzenesulfonamide (**3b**).


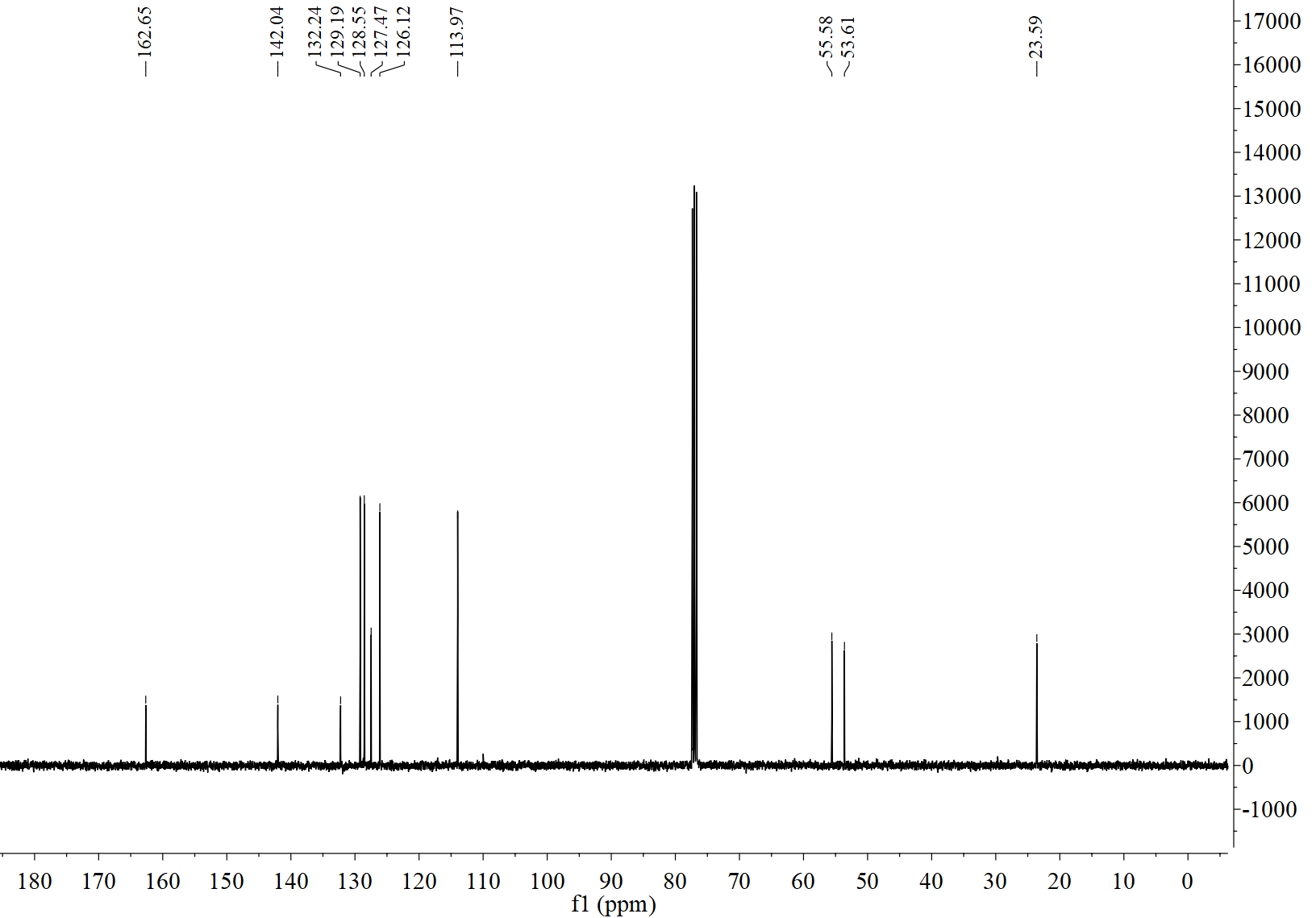


**Figure S15:** ^13^C-NMR (101 MHz, CDCl_3_) of 4-methoxy-*N*-(1-phenylethyl)benzenesulfonamide (**3b**).


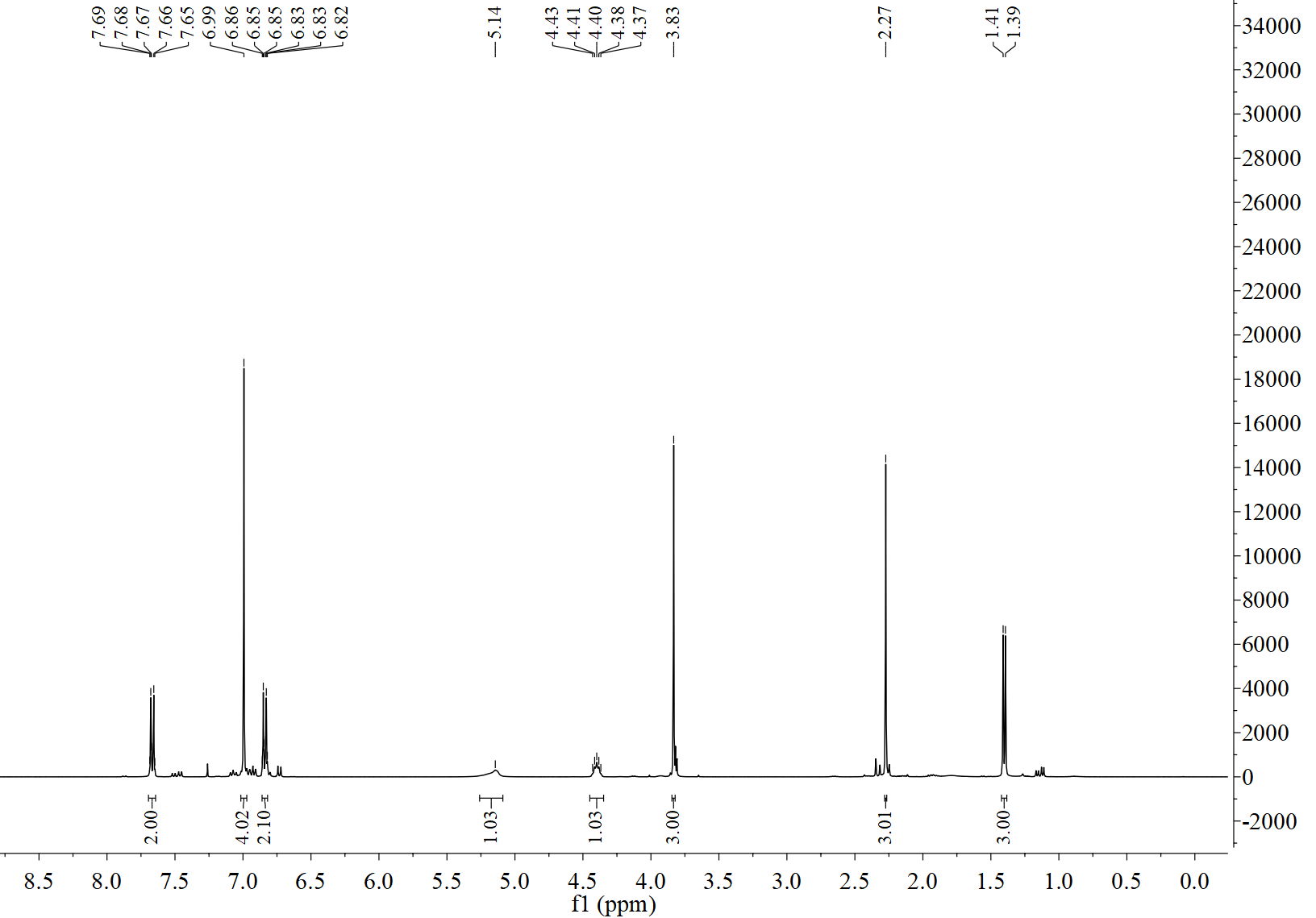


**Figure S16:** ^1^H-NMR (400 MHz, CDCl_3_) of 4-methoxy-*N*-(1-(*p*-tolyl)ethyl)benzenesulfonamide (**3c**).


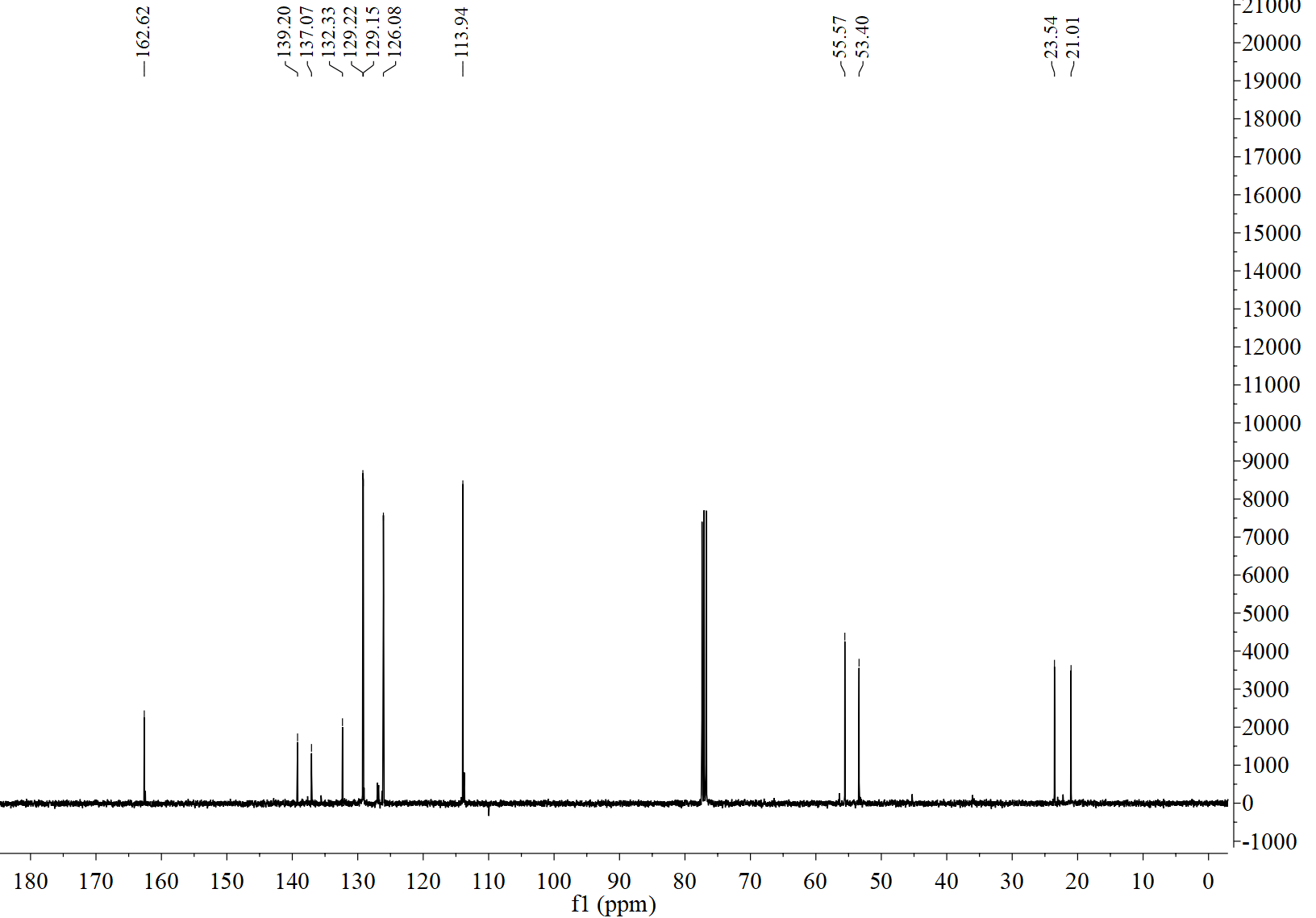


**Figure S17:** ^13^C-NMR (101 MHz, CDCl_3_) of 4-methoxy-*N*-(1-(*p*-tolyl)ethyl)benzenesulfonamide (**3c**).


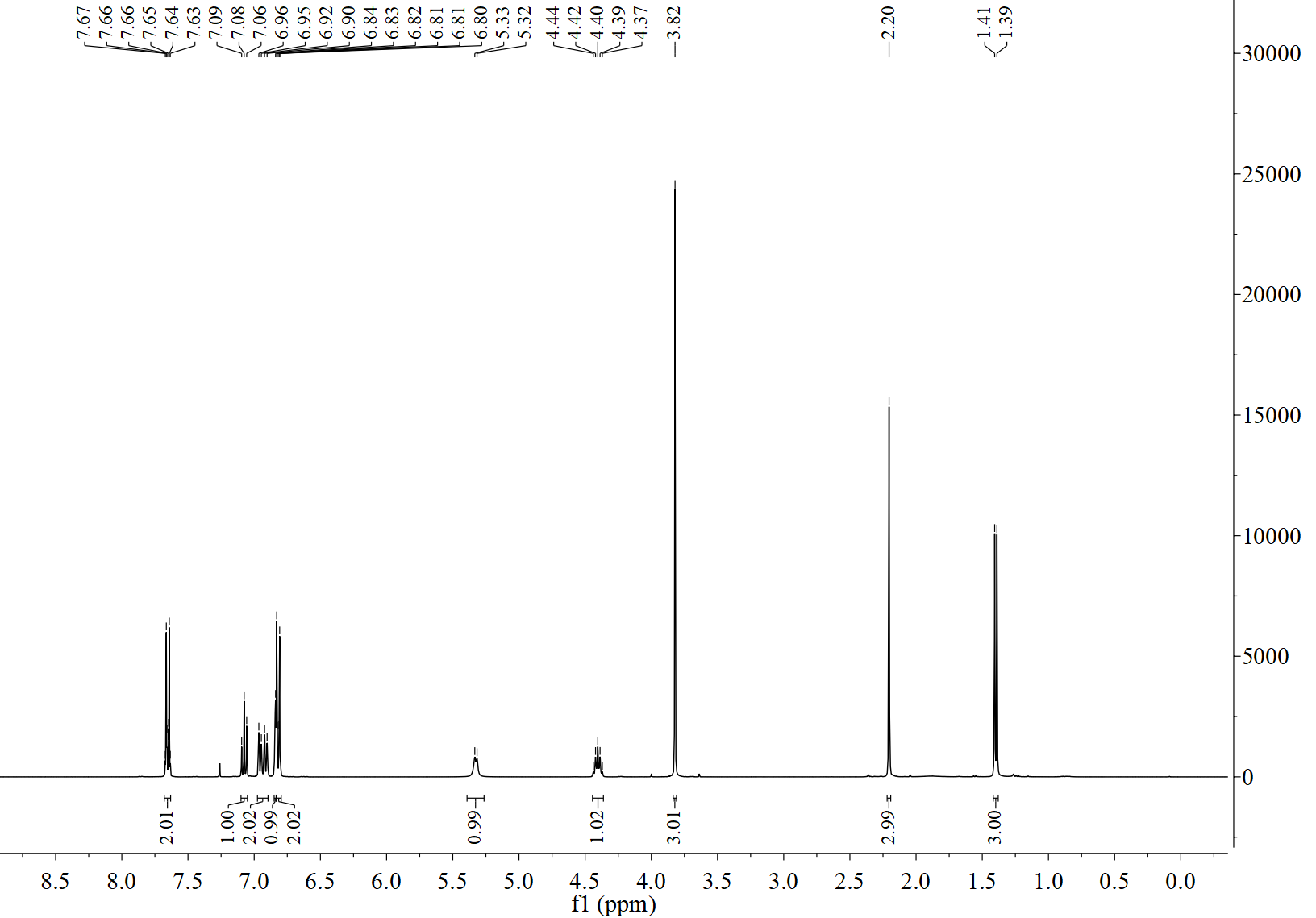


**Figure S18:** ^1^H-NMR (400 MHz, CDCl_3_) of 4-methoxy-*N*-(1-(*m*-tolyl)ethyl)benzenesulfonamide (**3d**).


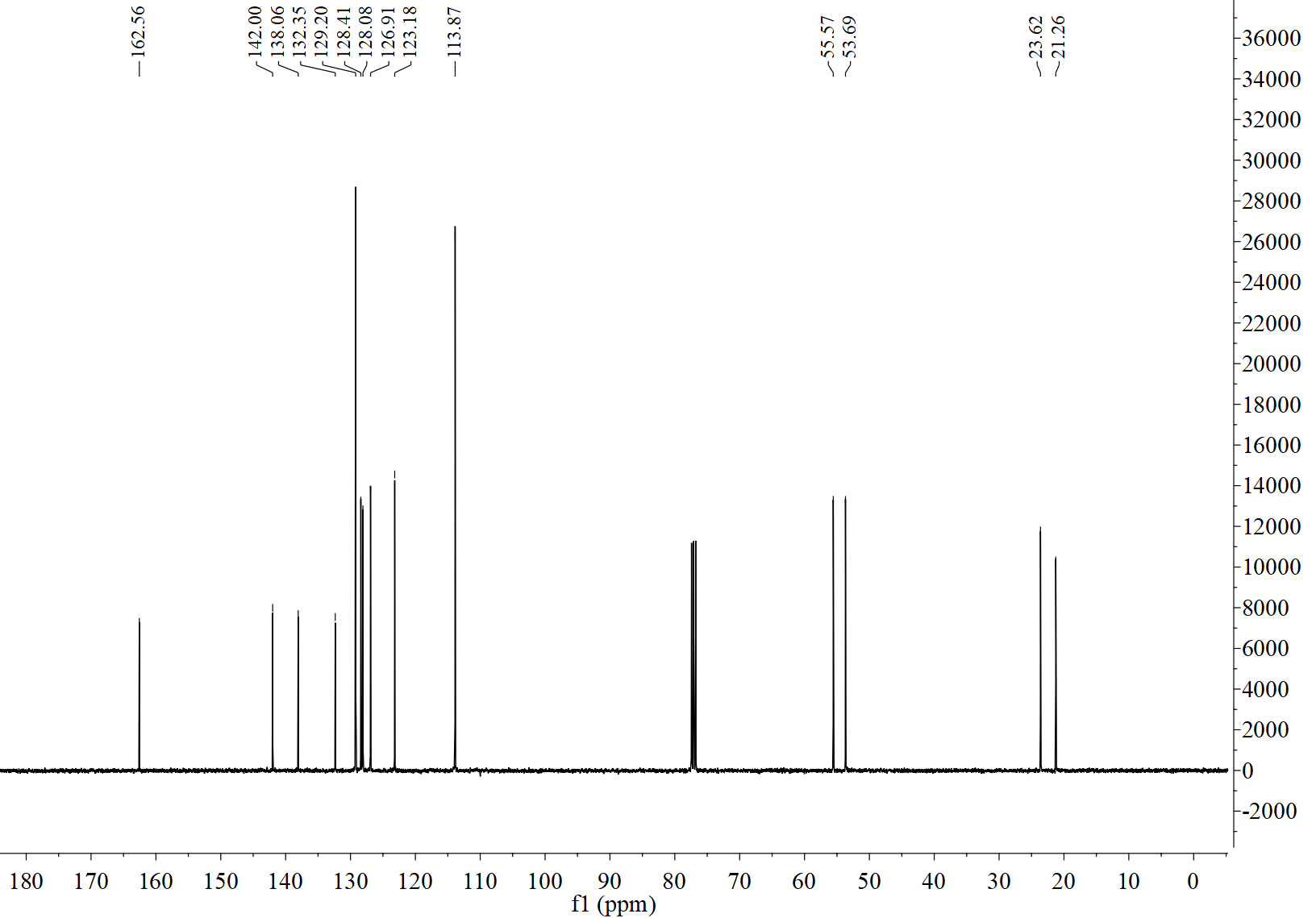


**Figure S19:** ^13^C-NMR (101 MHz, CDCl_3_) of 4-methoxy-*N*-(1-(*m*-tolyl)ethyl)benzenesulfonamide (**3d**).


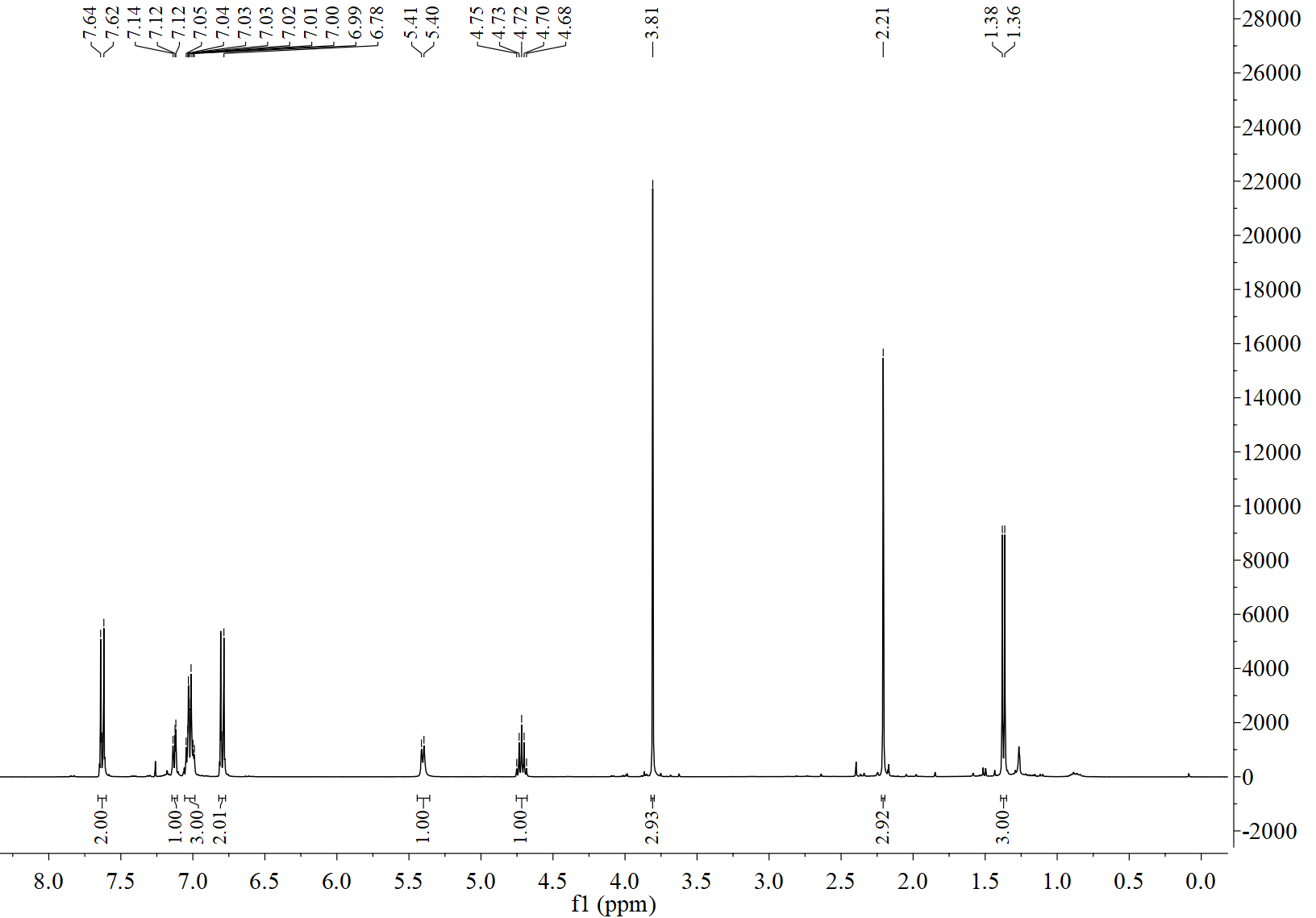


**Figure S20:** ^1^H-NMR (400 MHz, CDCl_3_) of 4-methoxy-*N*-(1-(*o*-tolyl)ethyl)benzenesulfonamide (**3e**).


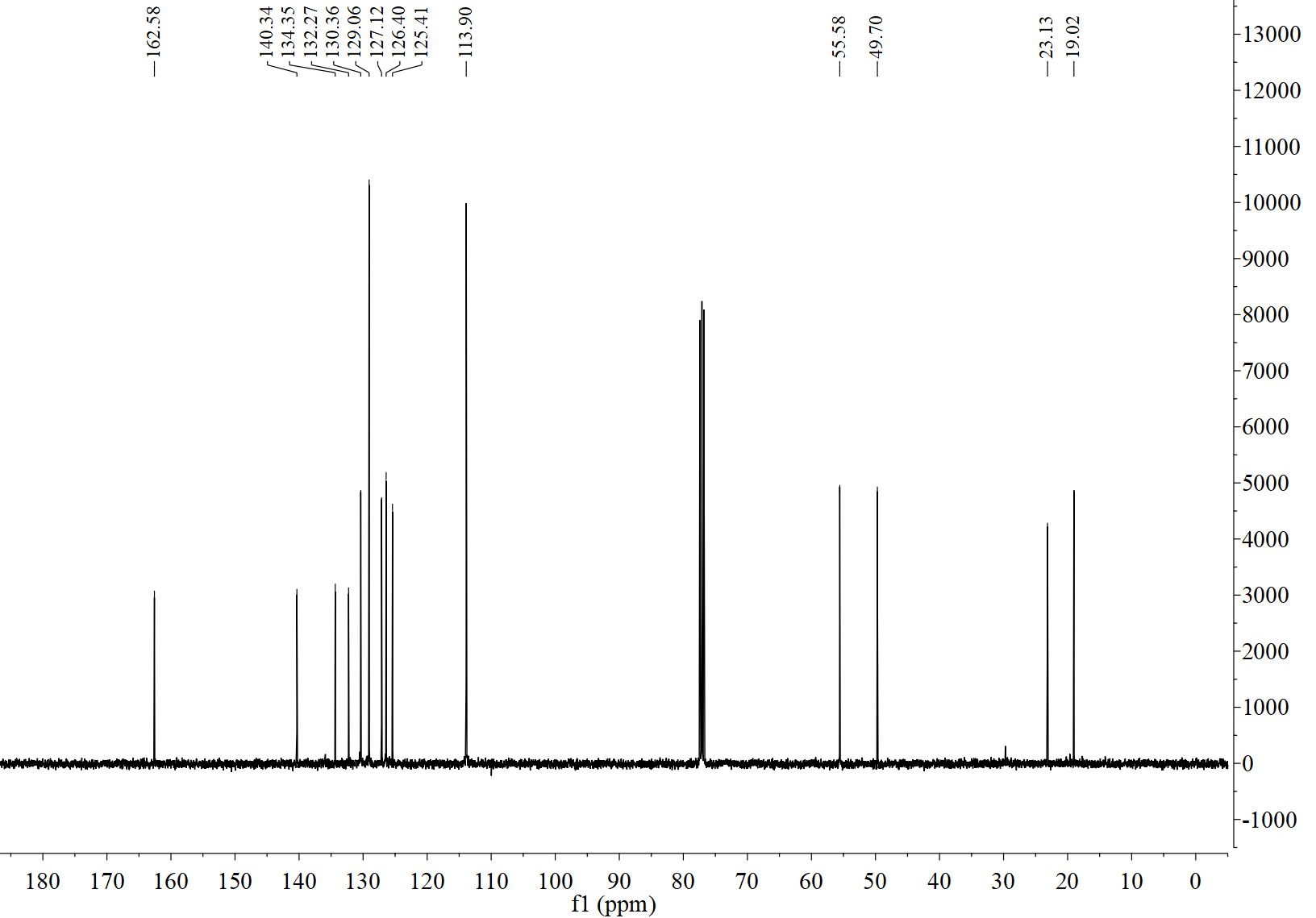


**Figure S21:** ^13^C-NMR (101 MHz, CDCl_3_) of 4-methoxy-*N*-(1-(*o*-tolyl)ethyl)benzenesulfonamide (**3e**).


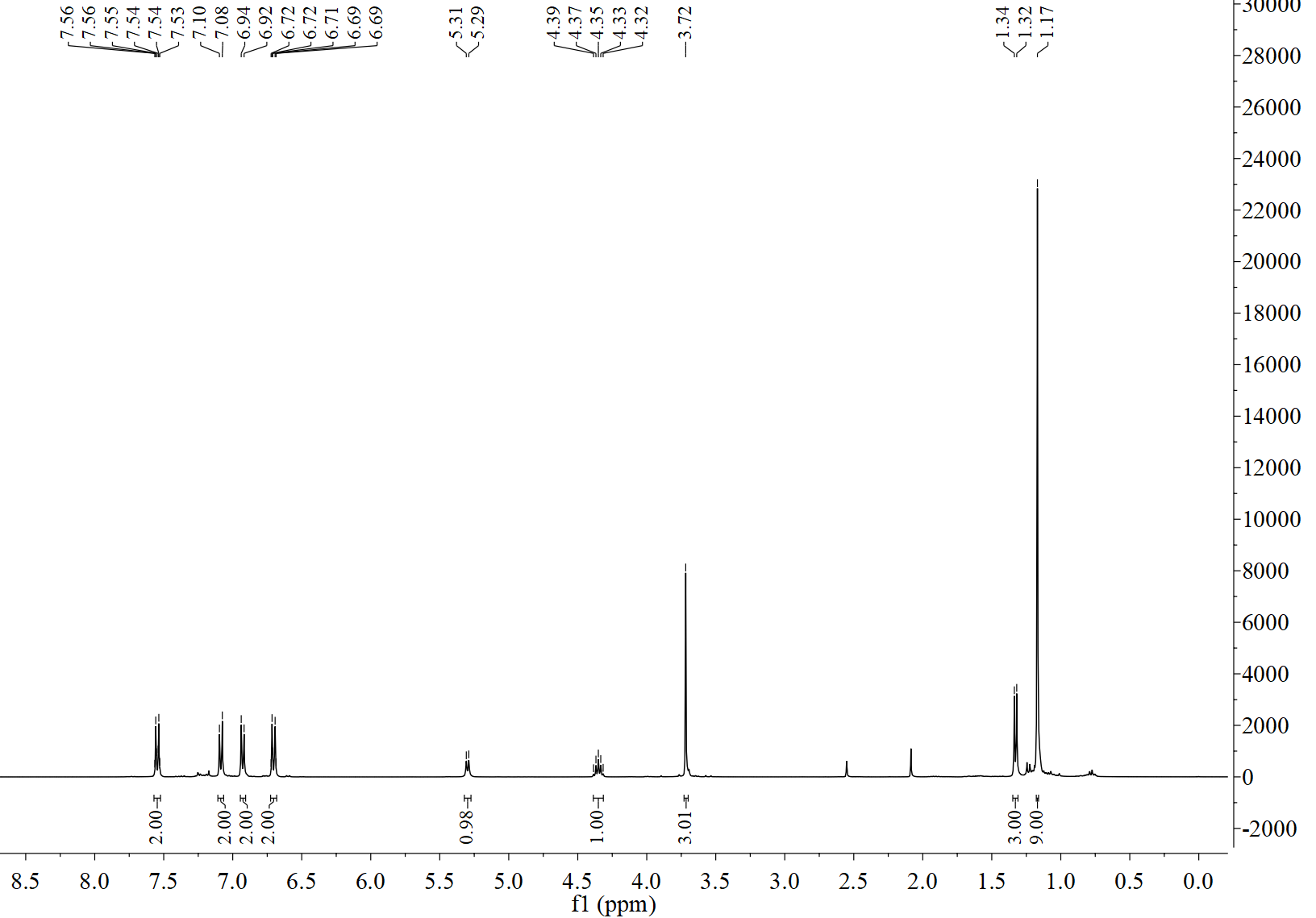


**Figure S22:** ^1^H-NMR (400 MHz, CDCl_3_) of *N*-(1-(4-(*tert*-butyl)phenyl)ethyl)-4-methoxybenzenesulfonamide (**3f**).


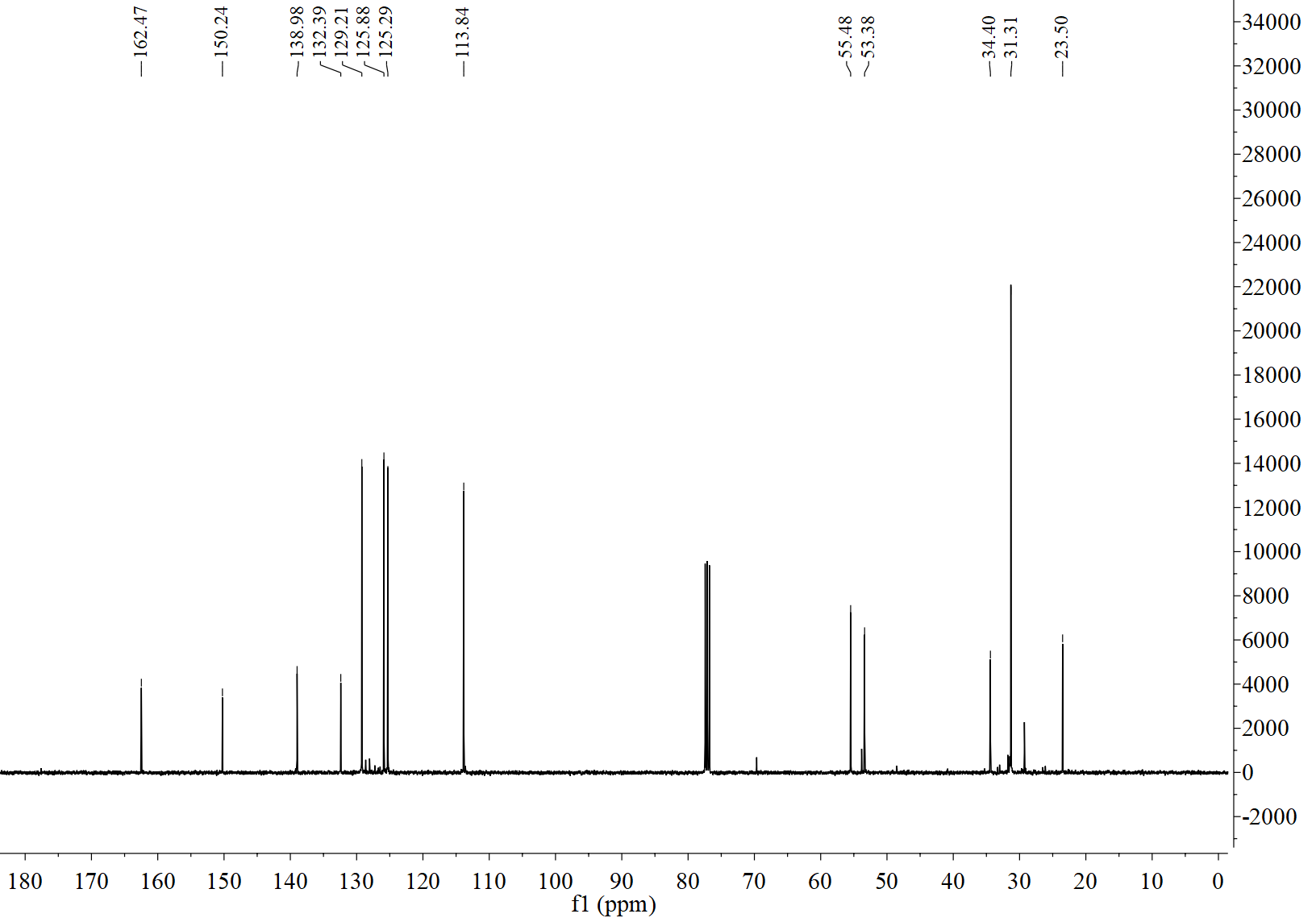


**Figure S23:** ^13^C-NMR (101 MHz, CDCl_3_) of *N*-(1-(4-(*tert*-butyl)phenyl)ethyl)-4-methoxybenzenesulfonamide (**3f**).


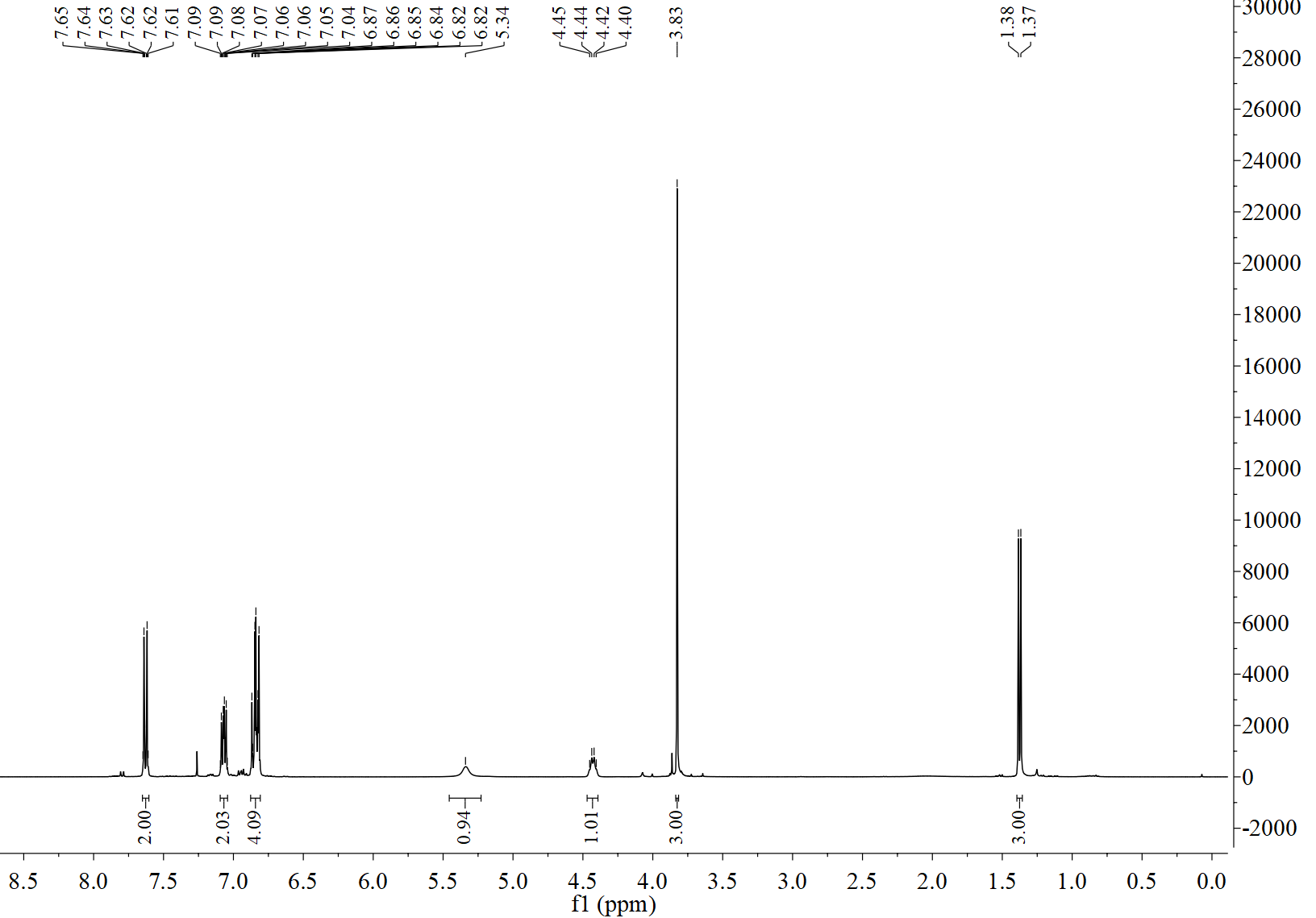


**Figure S24:** ^1^H-NMR (400 MHz, CDCl_3_) of *N*-(1-(4-fluorophenyl)ethyl)-4-methoxybenzenesulfonamide (**3g**).


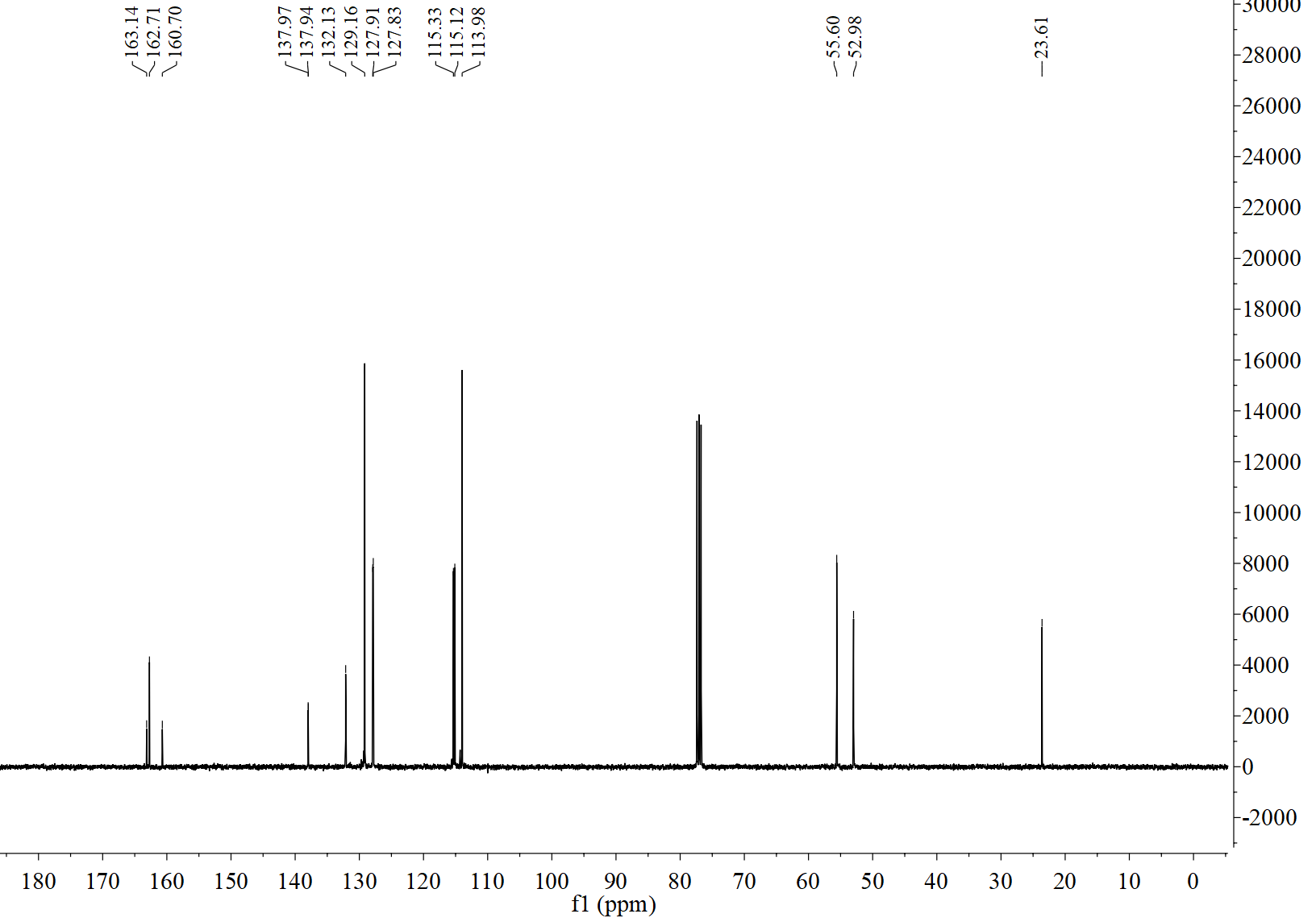


**Figure S25:** ^13^C-NMR (101 MHz, CDCl_3_) of *N*-(1-(4-fluorophenyl)ethyl)-4-methoxybenzenesulfonamide (**3g**).


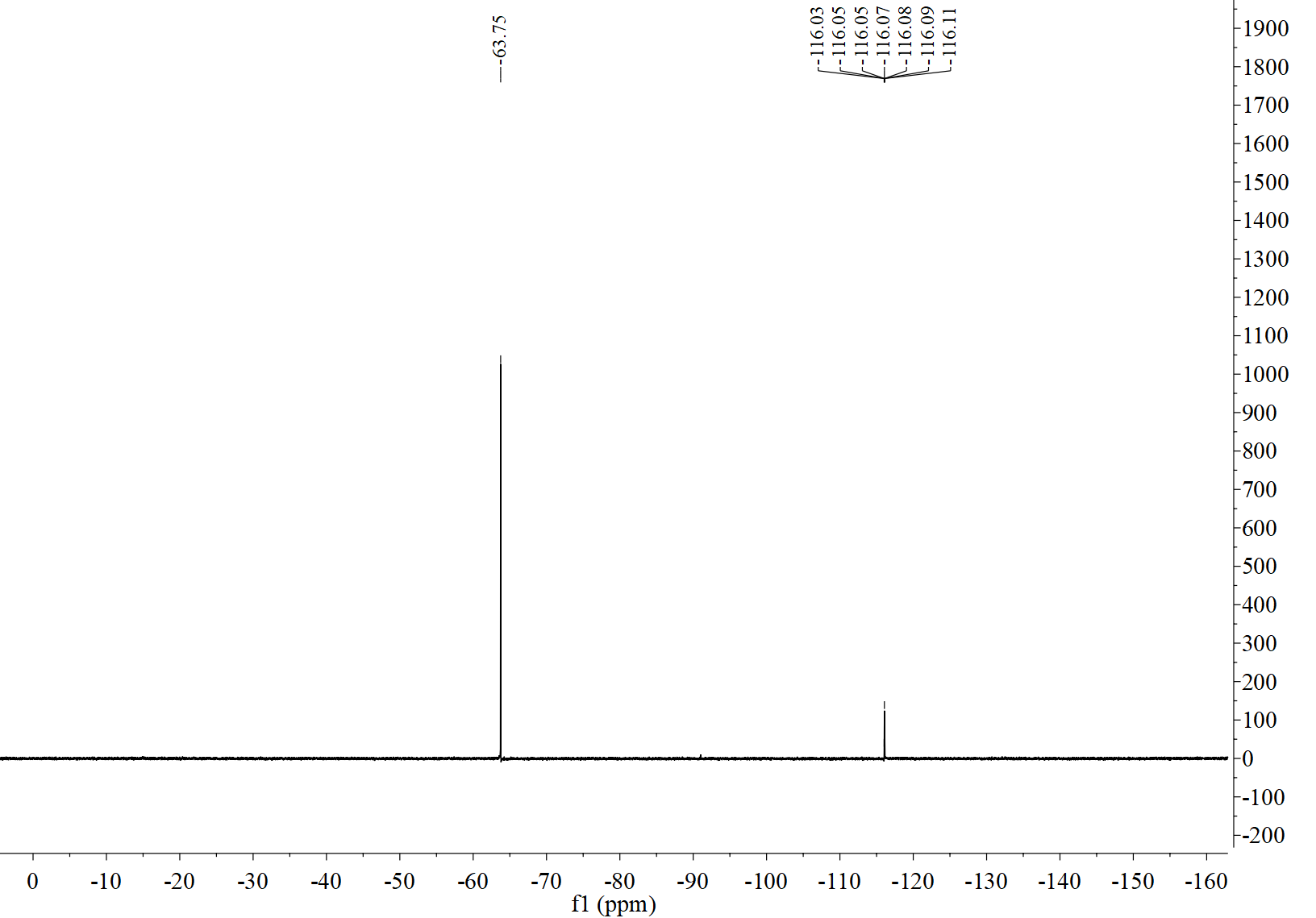


PhCF_3_

**Figure S26:** ^19^F-NMR (376 MHz, CDCl_3_) of *N*-(1-(4-fluorophenyl)ethyl)-4-methoxybenzenesulfonamide (**3g**) with PhCF_3_ as internal standard.


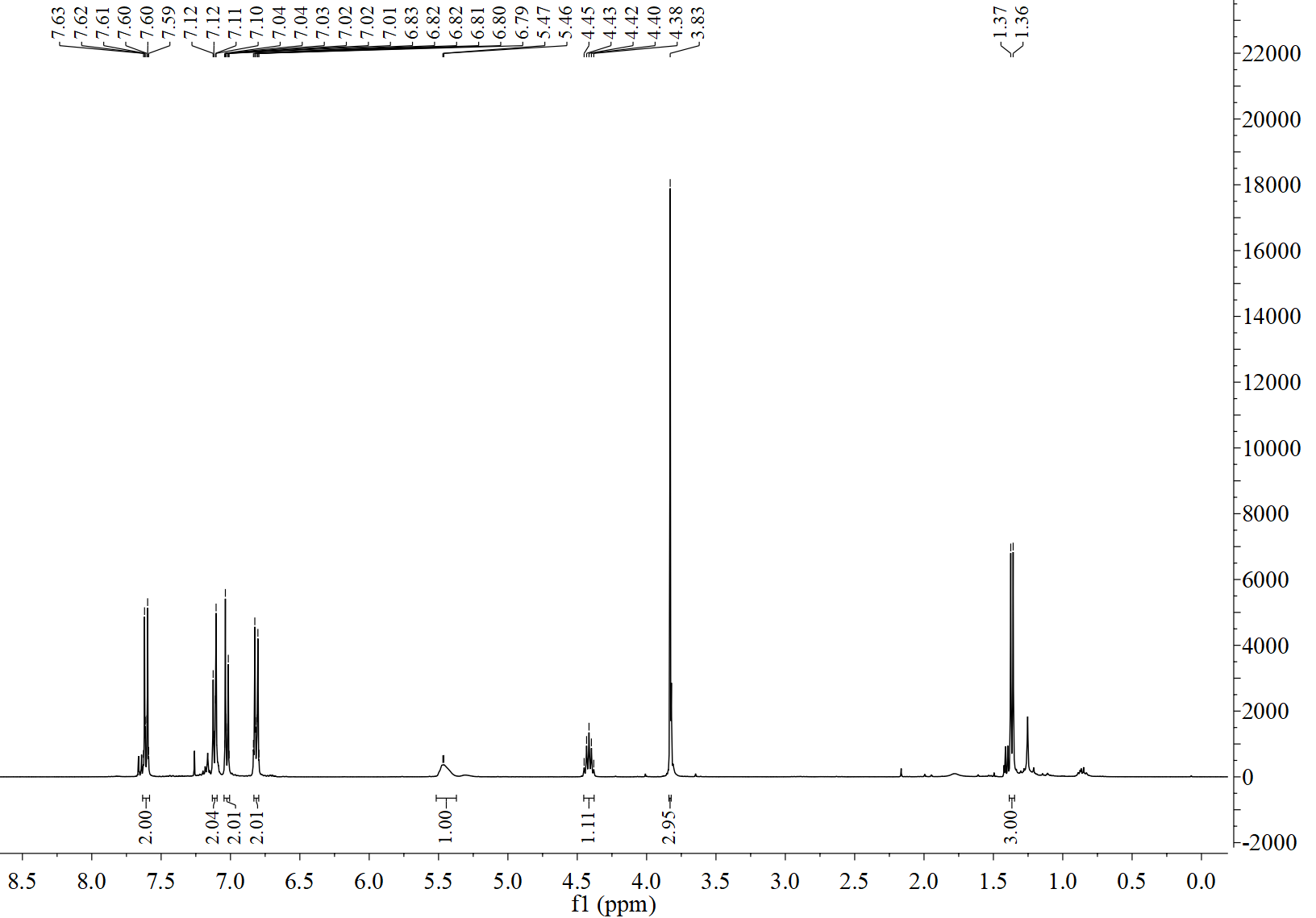


**Figure S27:** ^1^H-NMR (400 MHz, CDCl_3_) of *N*-(1-(4-chlorophenyl)ethyl)-4-methoxybenzenesulfonamide (**3h**).


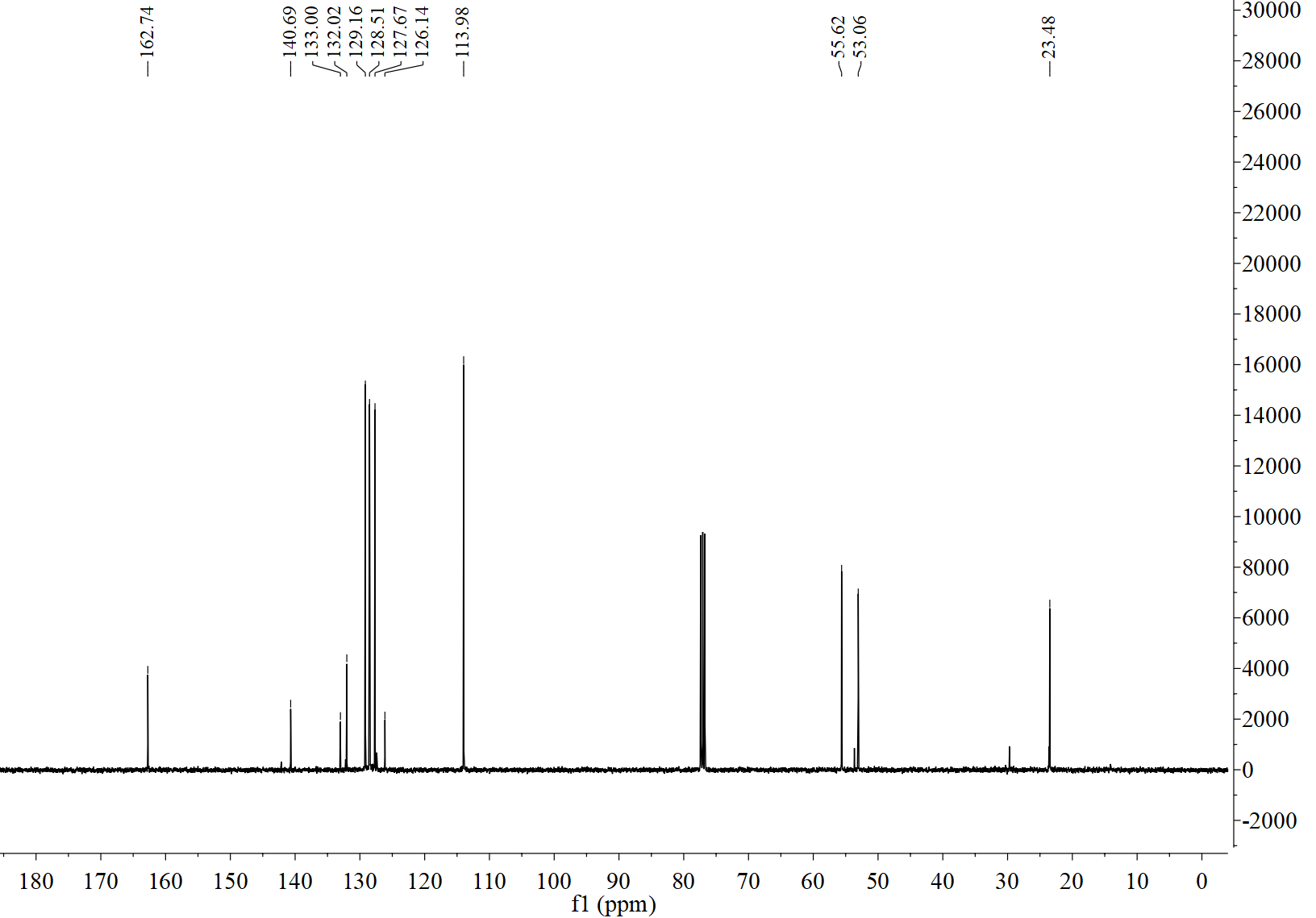


**Figure S28:** ^13^C-NMR (101 MHz, CDCl_3_) of *N*-(1-(4-chlorophenyl)ethyl)-4-methoxybenzenesulfonamide (**3h**).


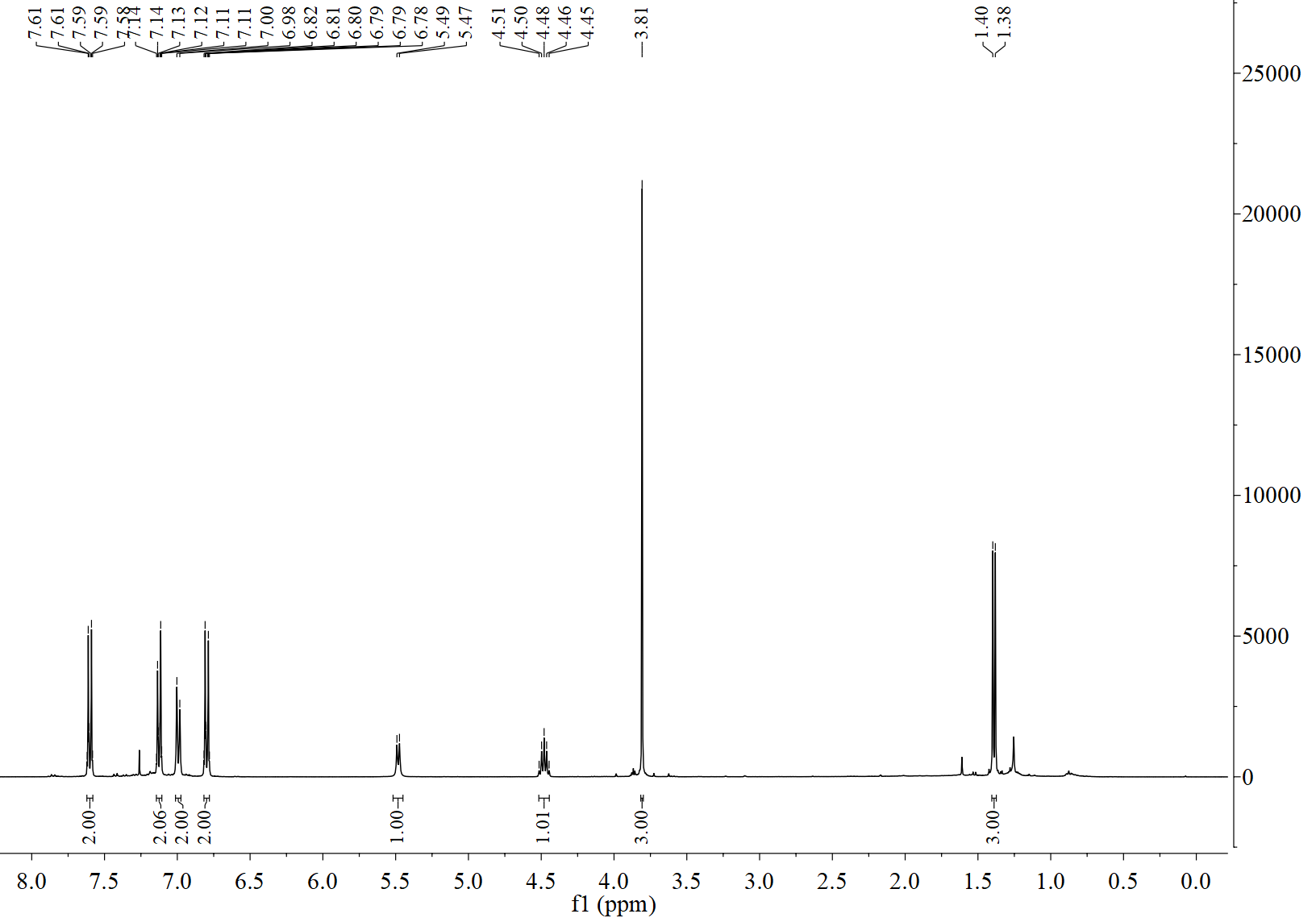


**Figure S29:** ^1^H-NMR (400 MHz, CDCl_3_) of *N*-(1-(4-trifluoromethoxyphenyl)ethyl)-4-methoxybenzenesulfonamide (**3i**).


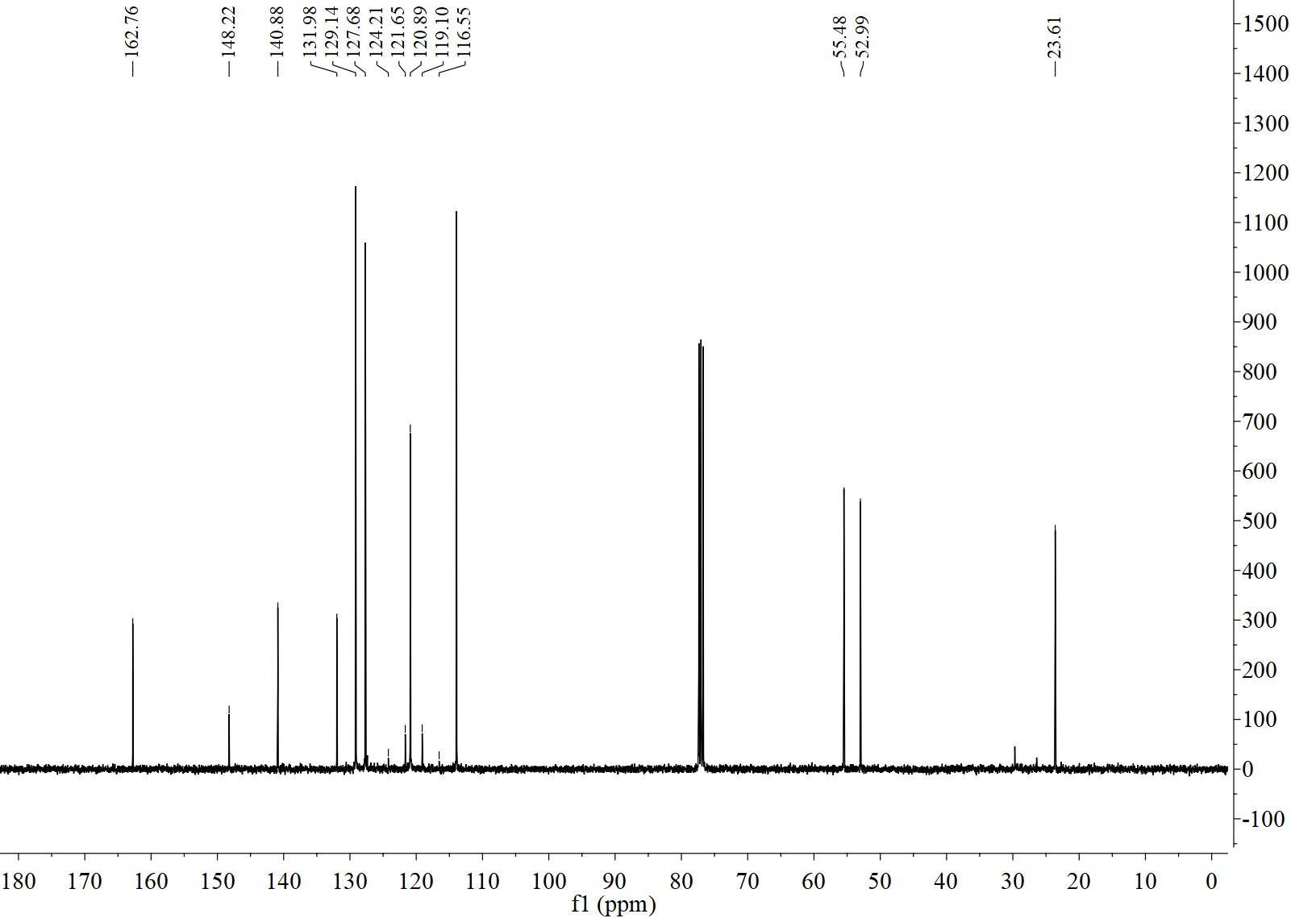


**Figure S30:** ^13^C-NMR (101 MHz, CDCl_3_) of *N*-(1-(4-trifluoromethoxyphenyl)ethyl)-4-methoxybenzenesulfonamide (**3i**)


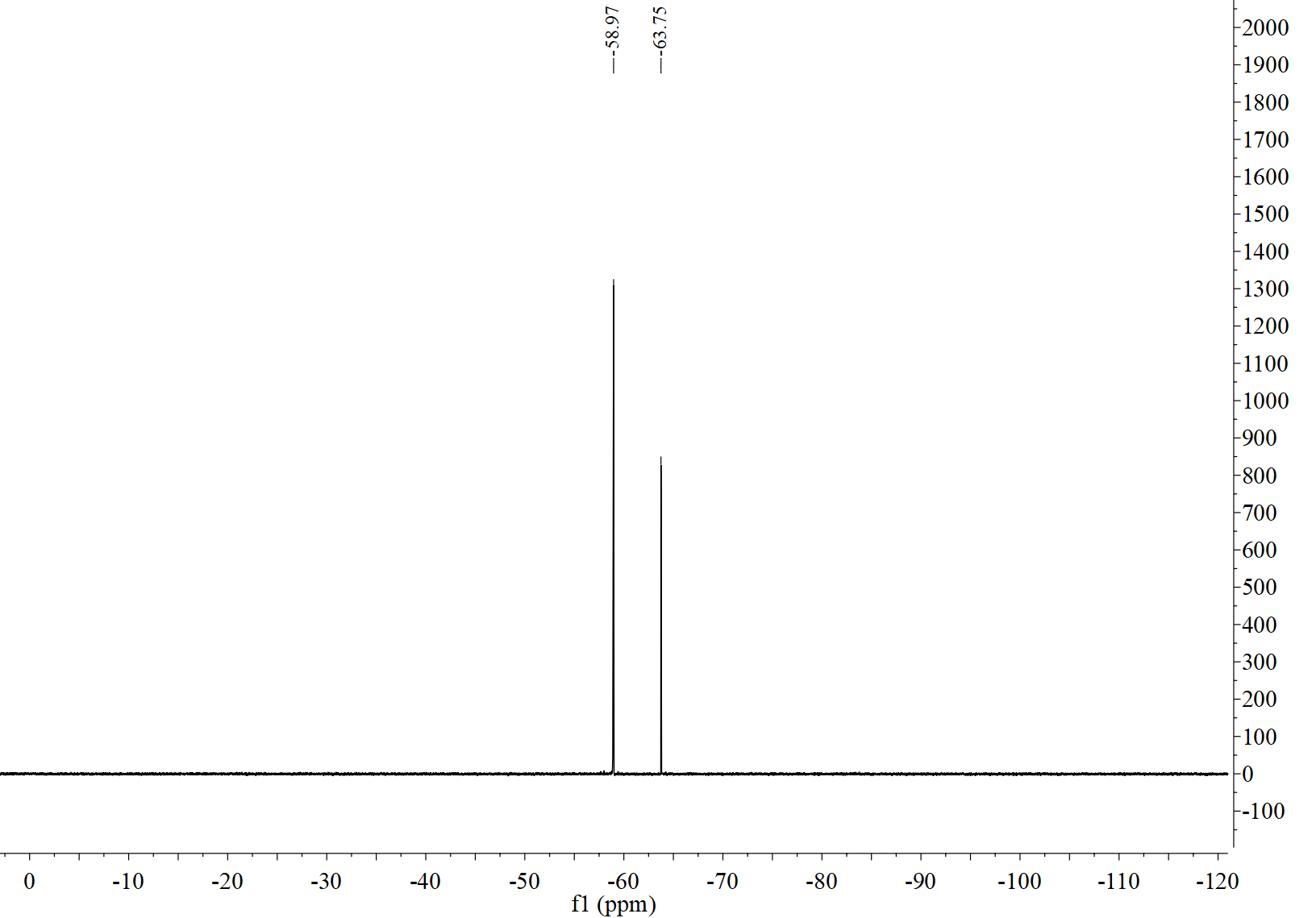


PhCF_3_

**Figure S31:** ^19^F-NMR (376 MHz, CDCl_3_) of *N*-(1-(4-trifluoromethoxyphenyl)ethyl)-4-methoxybenzenesulfonamide (**3i**) with PhCF_3_ as internal standard.


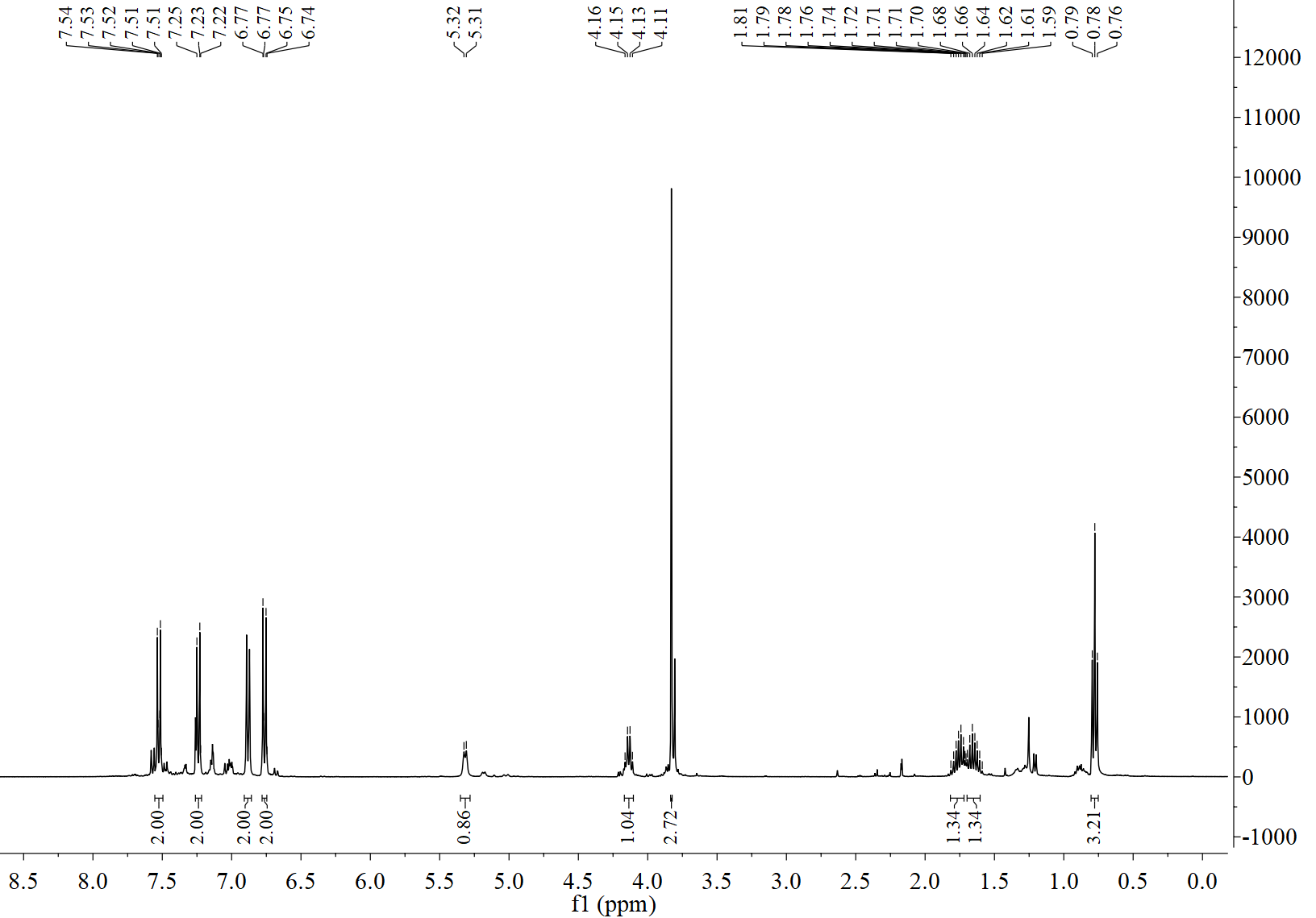


**Figure S32:** ^1^H-NMR (400 MHz, CDCl_3_) of *N*-bezhydryl-4-methylbenzenesulfonamide (**3j**).


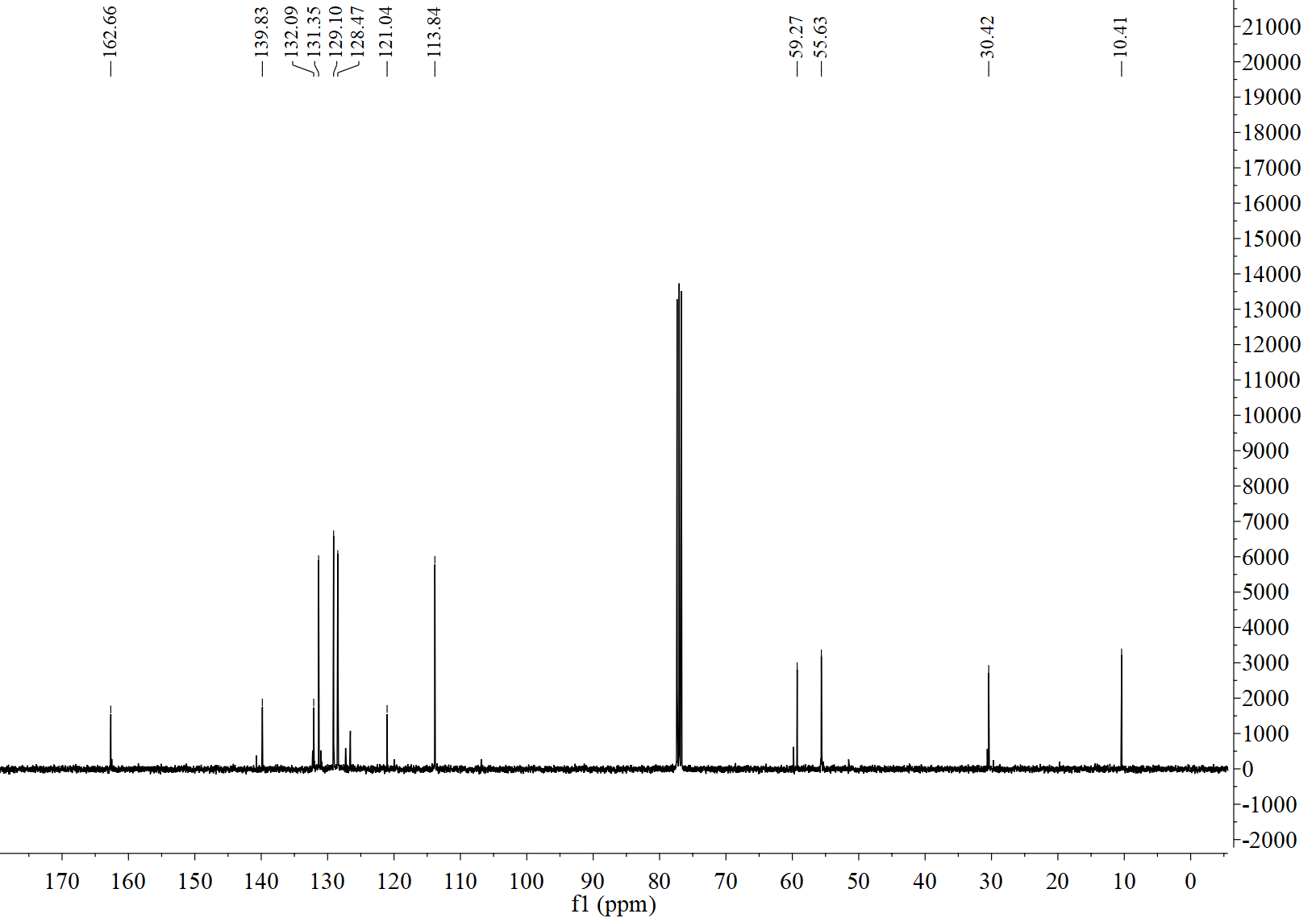


**Figure S33:** ^13^C-NMR (101 MHz, CDCl_3_) of *N*-bezhydryl-4-methylbenzenesulfonamide (**3j**).


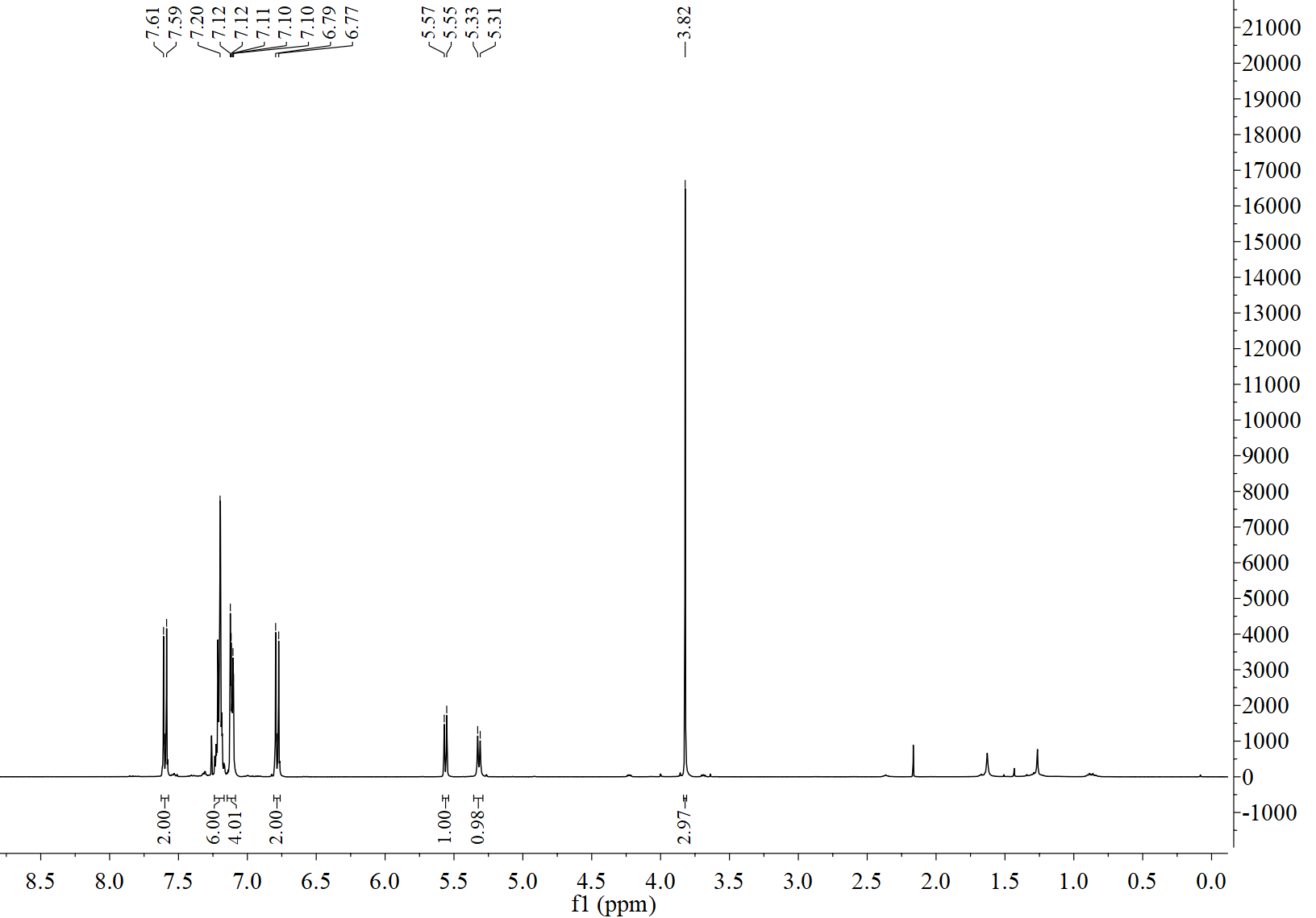


**Figure S34:** ^1^H-NMR (400 MHz, CDCl_3_) of *N*-bezhydryl-4-methylbenzenesulfonamide (**3k**).


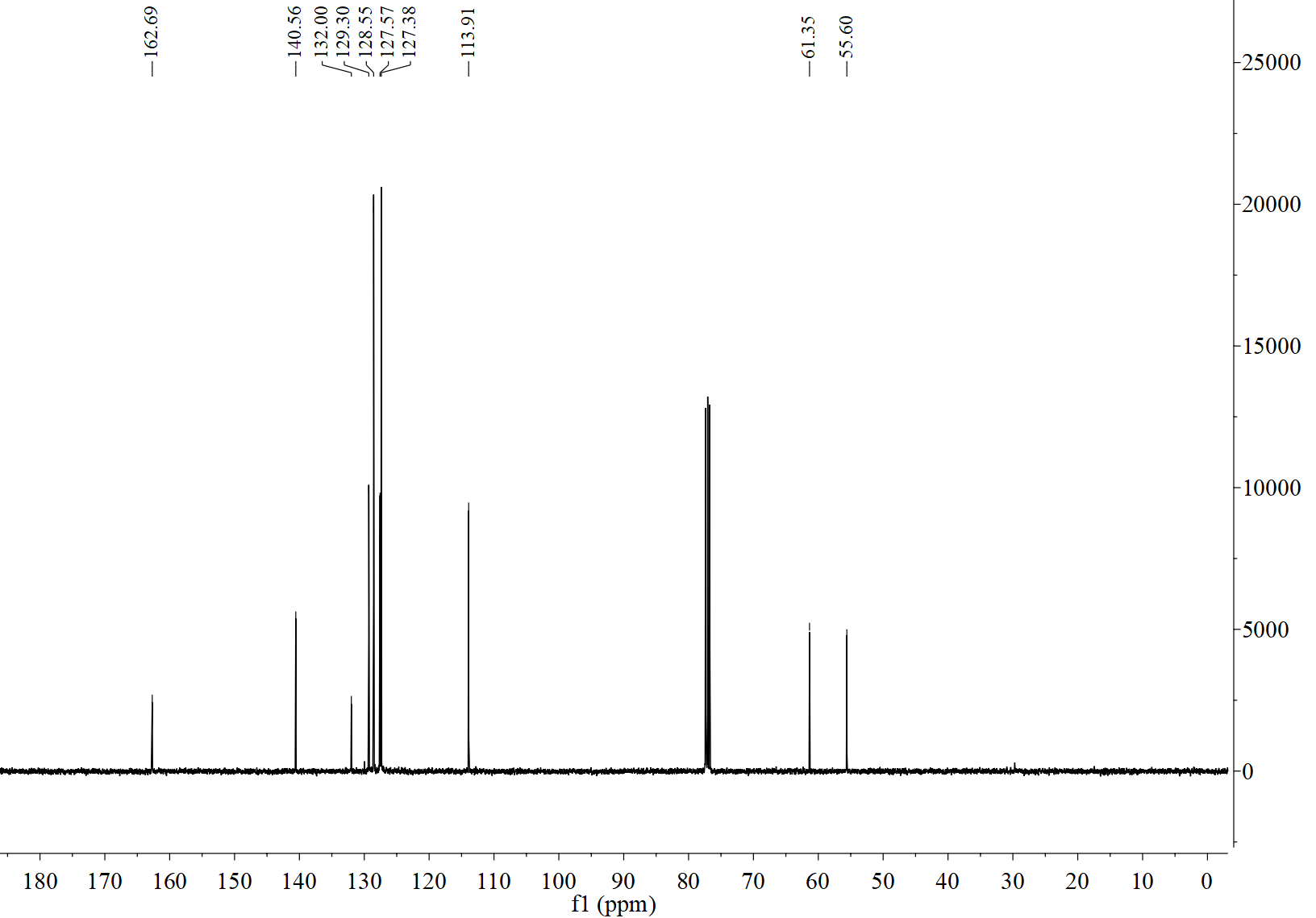


**Figure S35:** ^13^C-NMR (101 MHz, CDCl_3_) of *N*-bezhydryl-4-methylbenzenesulfonamide (**3k**).


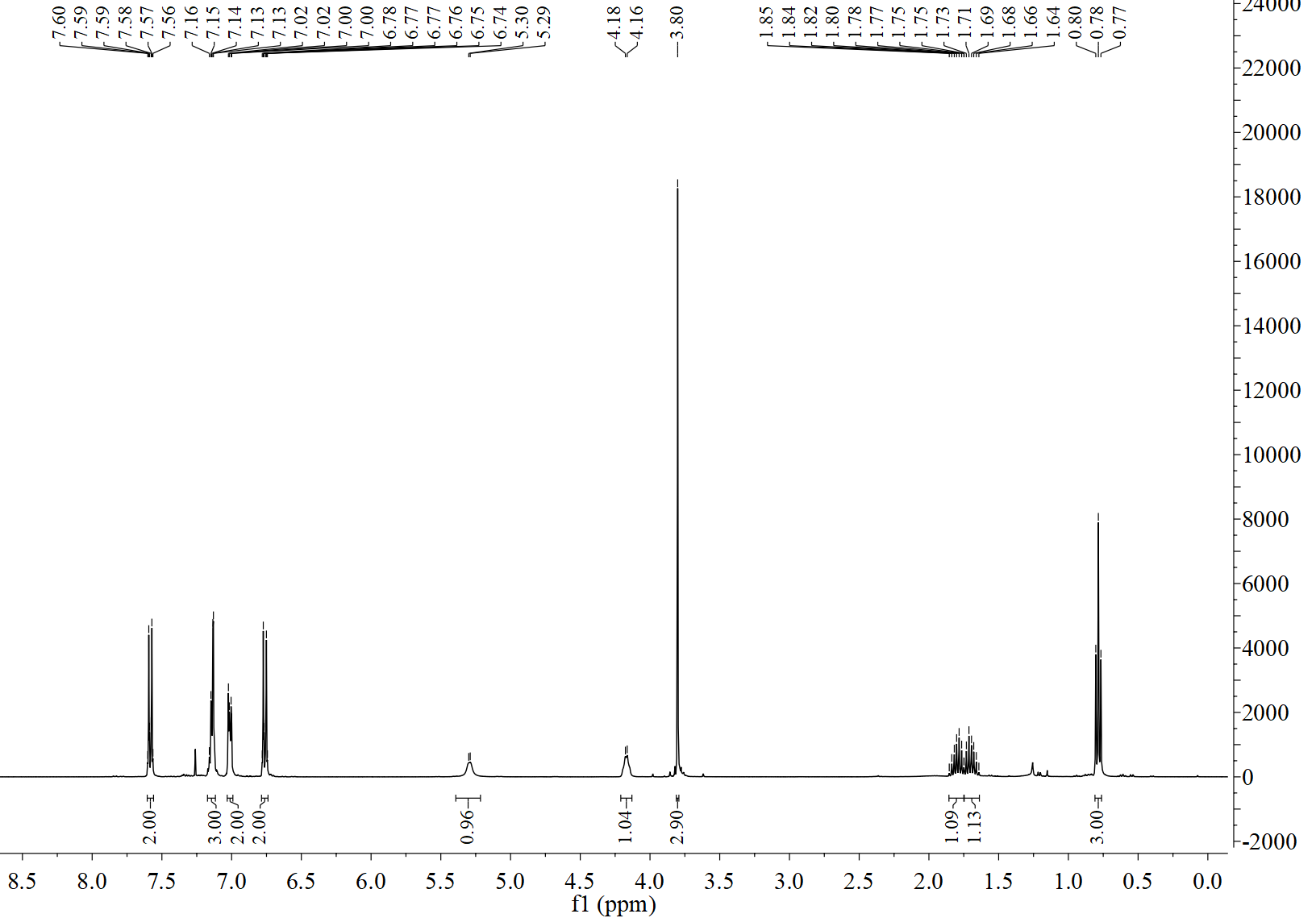


**Figure S36:** ^1^H-NMR (400 MHz, CDCl_3_) of 4-methoxy-*N*-(1-phenylpropyl)benzenesulfonamide (**3l**).


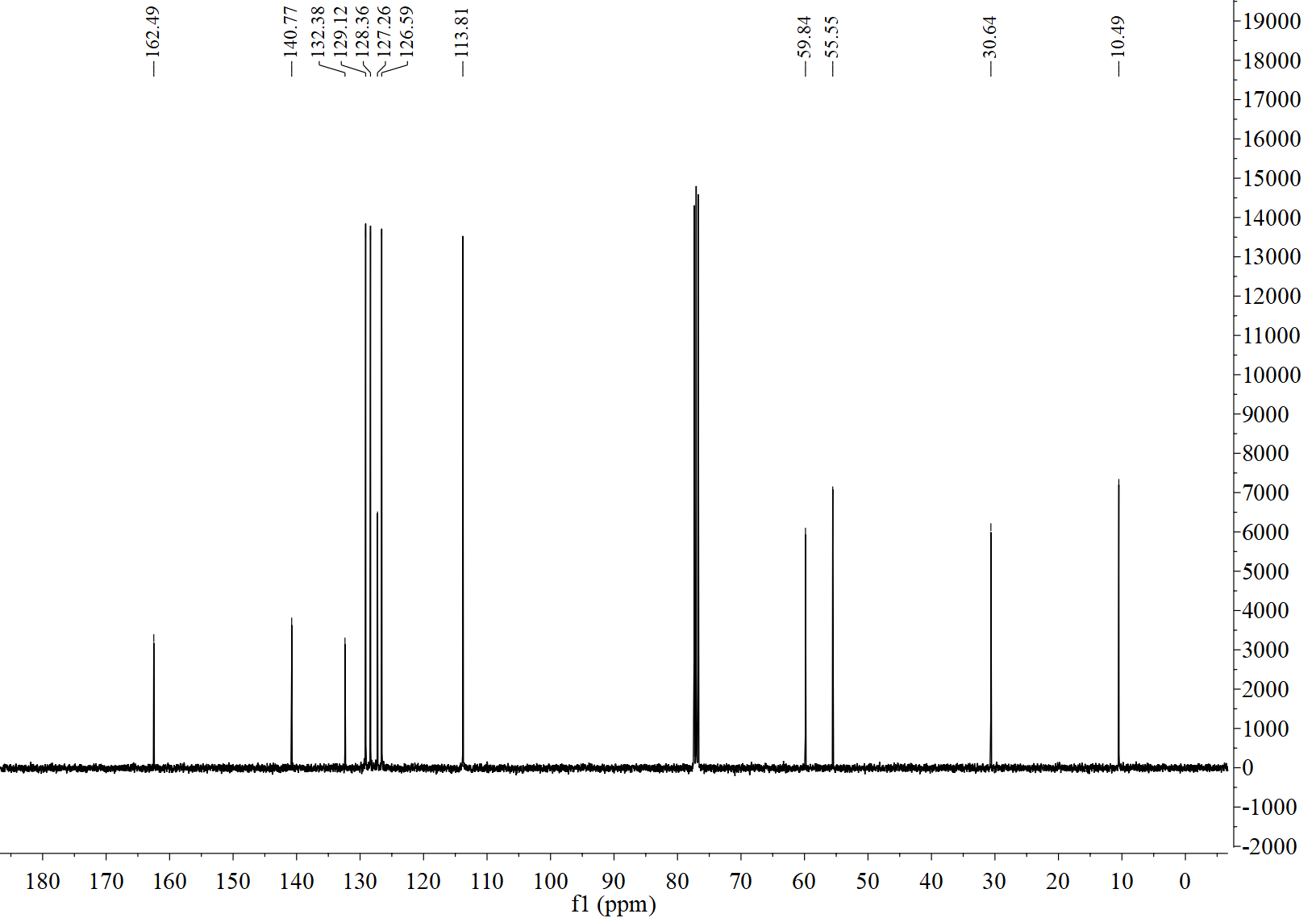


**Figure S37:** ^13^C-NMR (101 MHz, CDCl_3_) of 4-methoxy-*N*-(1-phenylpropyl)benzenesulfonamide (**3l**).


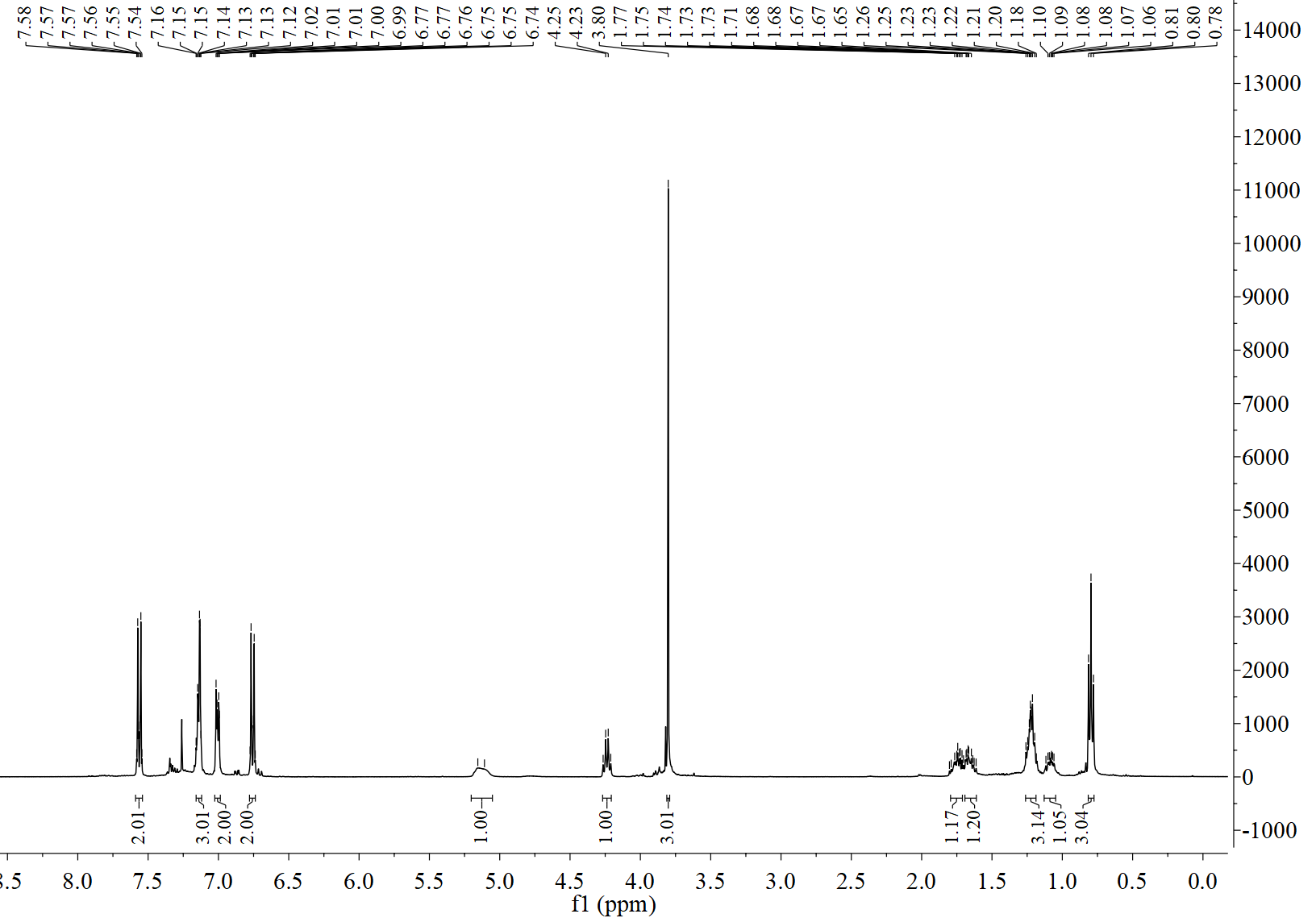


**Figure S38:** ^1^H-NMR (400 MHz, CDCl_3_) of 4-methoxy-*N*-(1-phenylpentyl)benzenesulfonamide (**3m**).


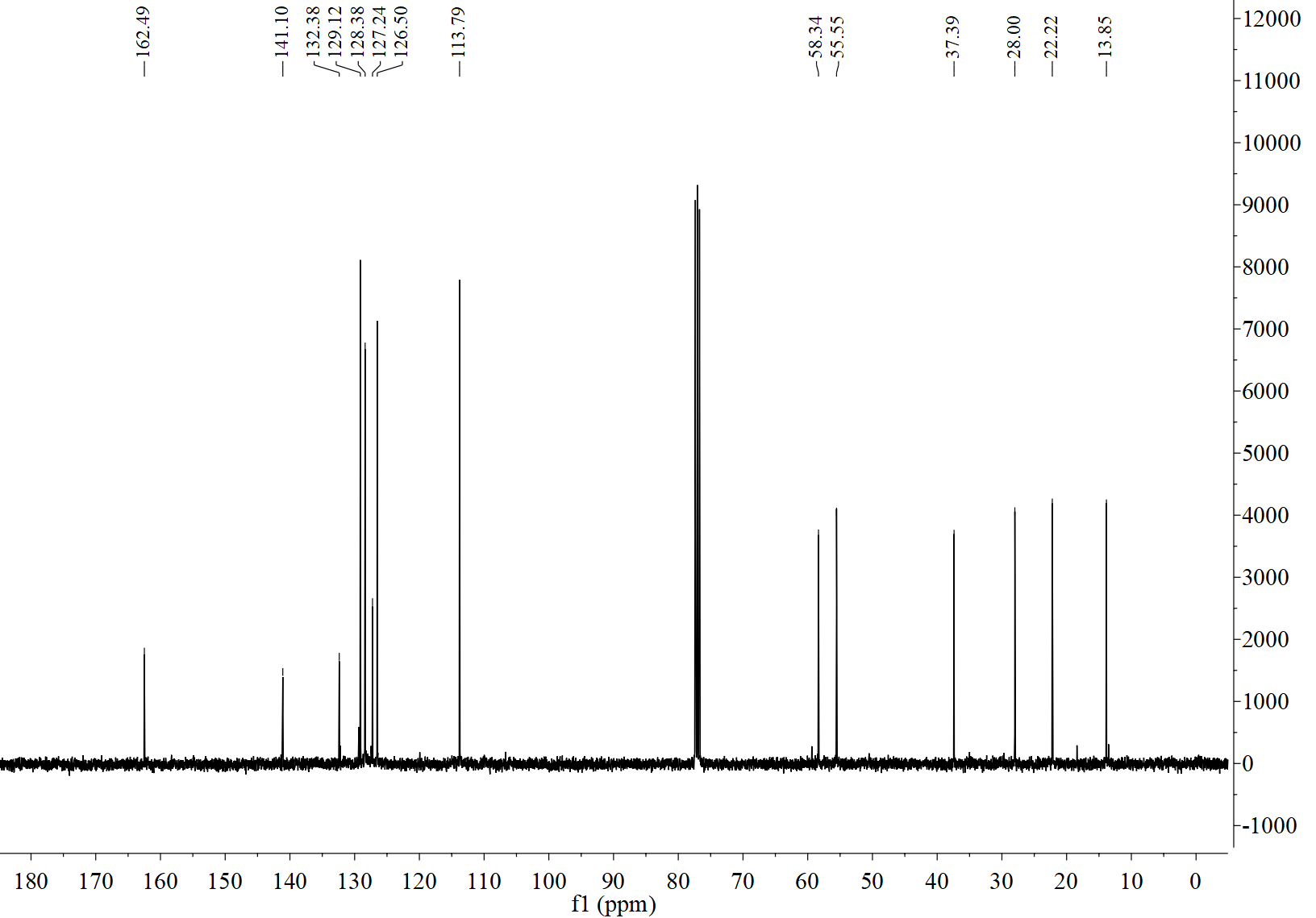


**Figure S39:** ^13^C-NMR (101 MHz, CDCl_3_) of 4-methoxy-*N*-(1-phenylpentyl)benzenesulfonamide (**3m**).


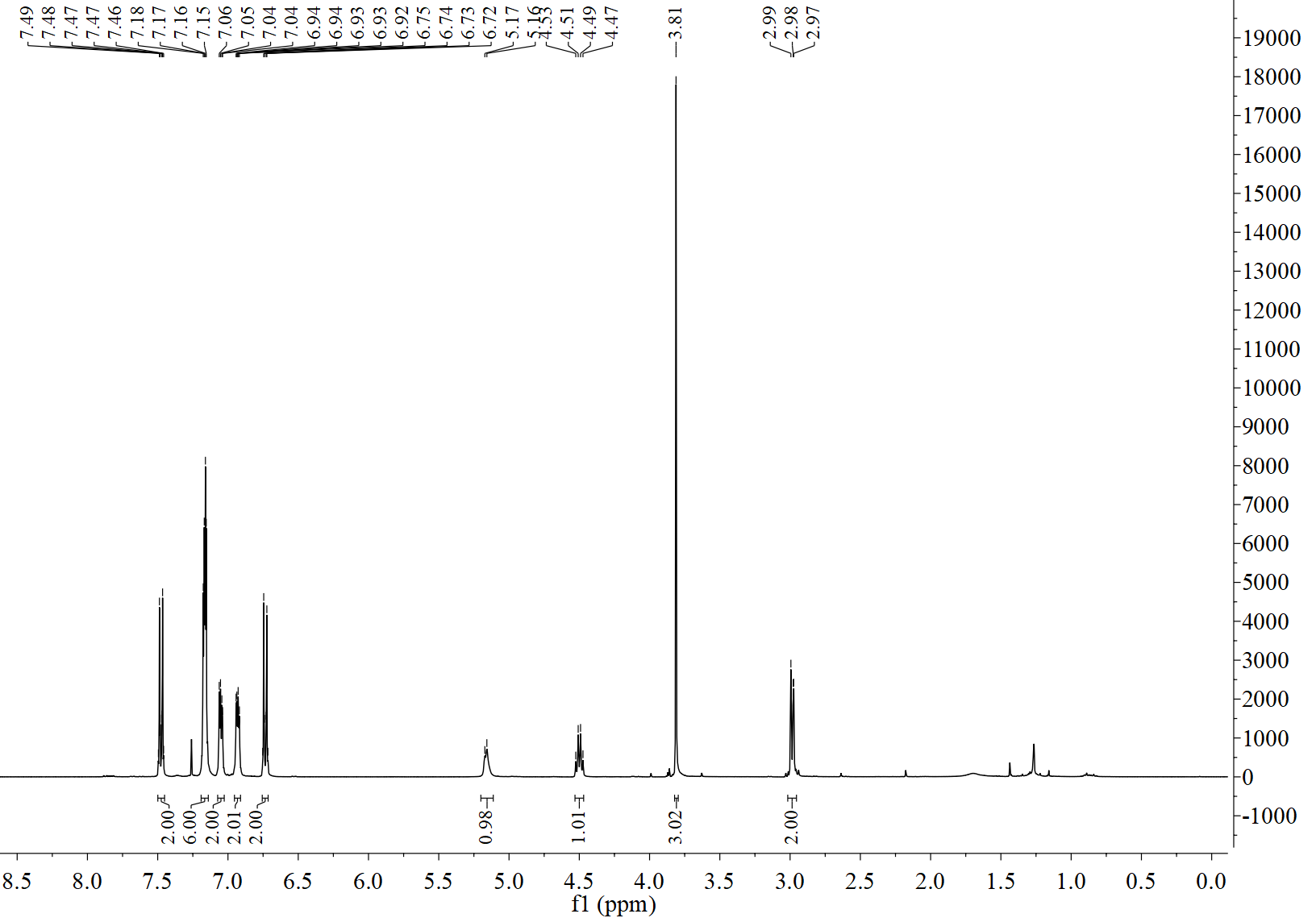


**Figure S40:** ^1^H-NMR (400 MHz, CDCl_3_) of *N*-(1,2-diphenylethyl)-4-methoxybenzenesulfonamide (**3n**).


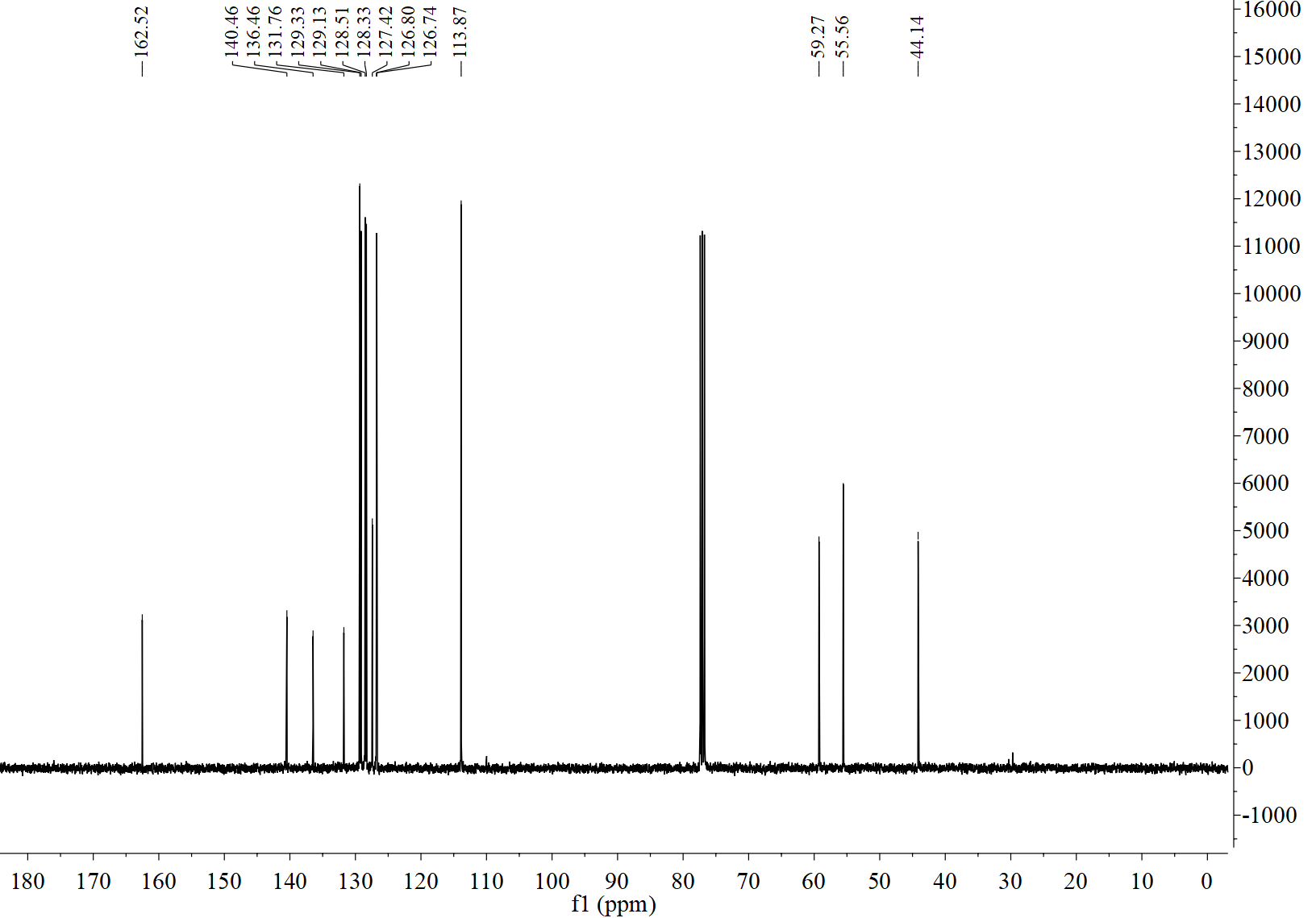


**Figure S41:** ^13^C-NMR (101 MHz, CDCl_3_) of *N*-(1,2-diphenylethyl)-4-methoxybenzenesulfonamide (**3n**).


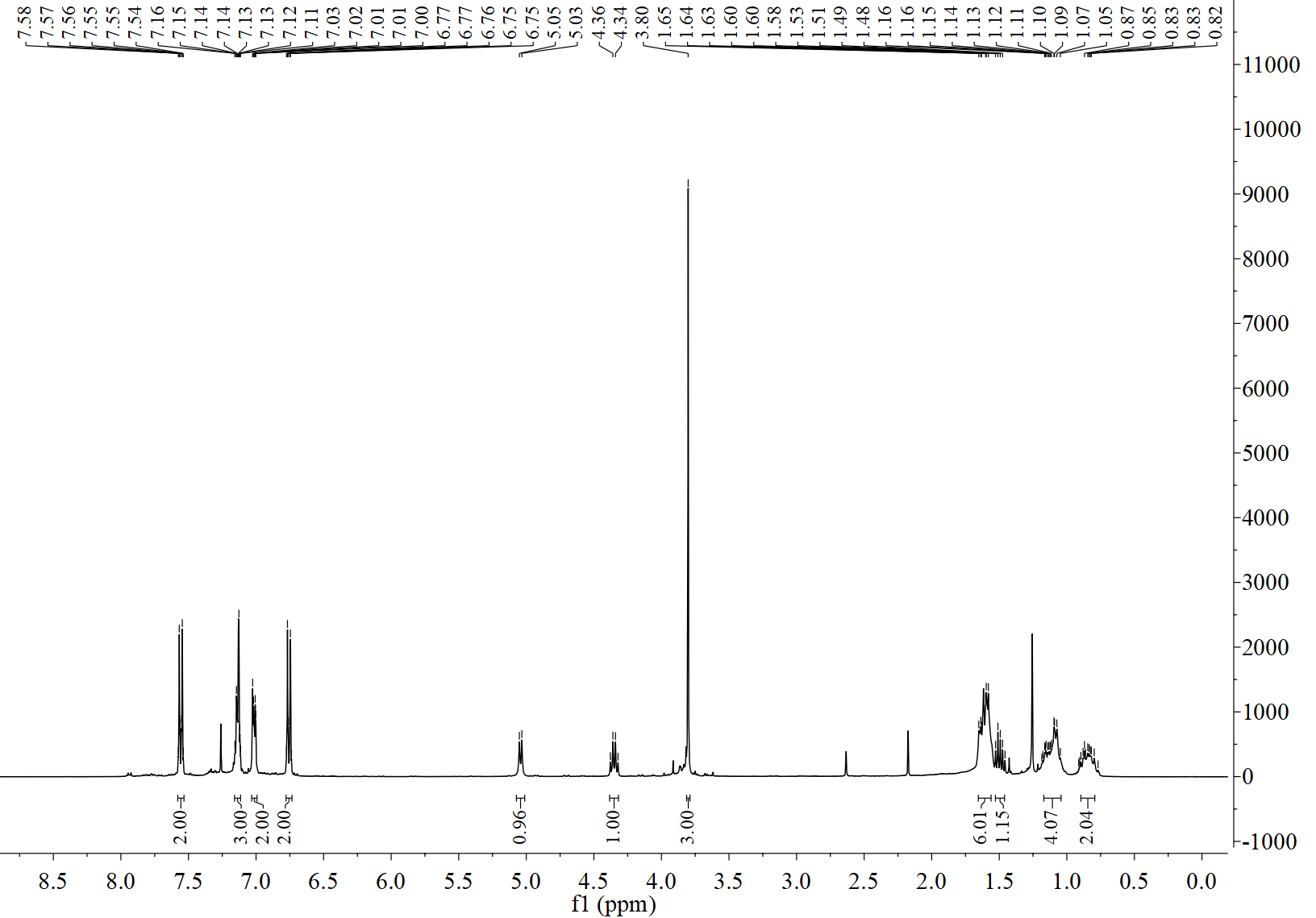


**Figure S42:** ^1^H-NMR (400 MHz, CDCl_3_) of *N*-(2-cyclohexyl-1-phenylethyl)-4-methoxybenzenesulfonamide (**3o**).


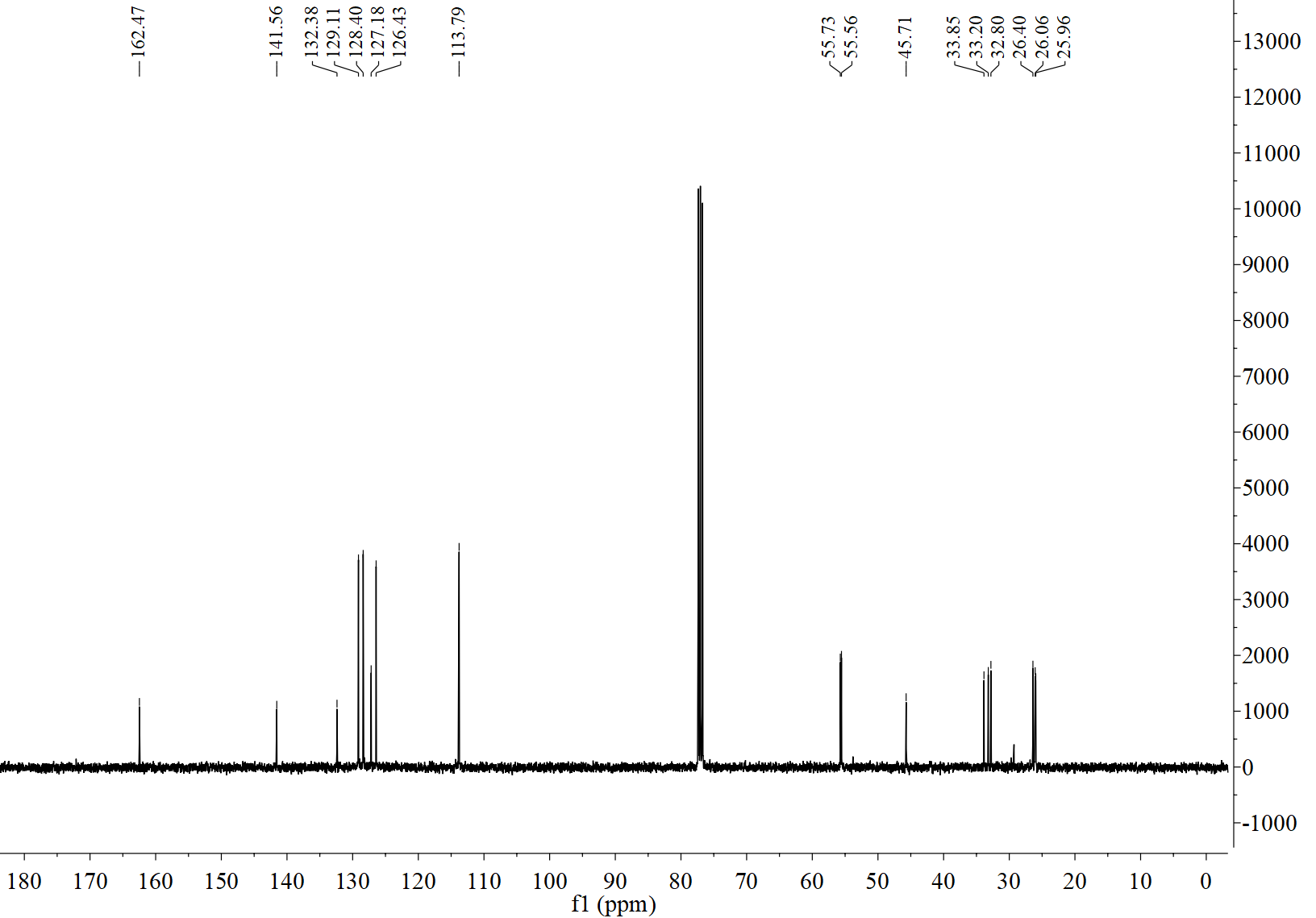


**Figure S43:** ^13^C-NMR (101 MHz, CDCl_3_) of *N*-(2-cyclohexyl-1-phenylethyl)-4-methoxybenzenesulfonamide (**3o**).


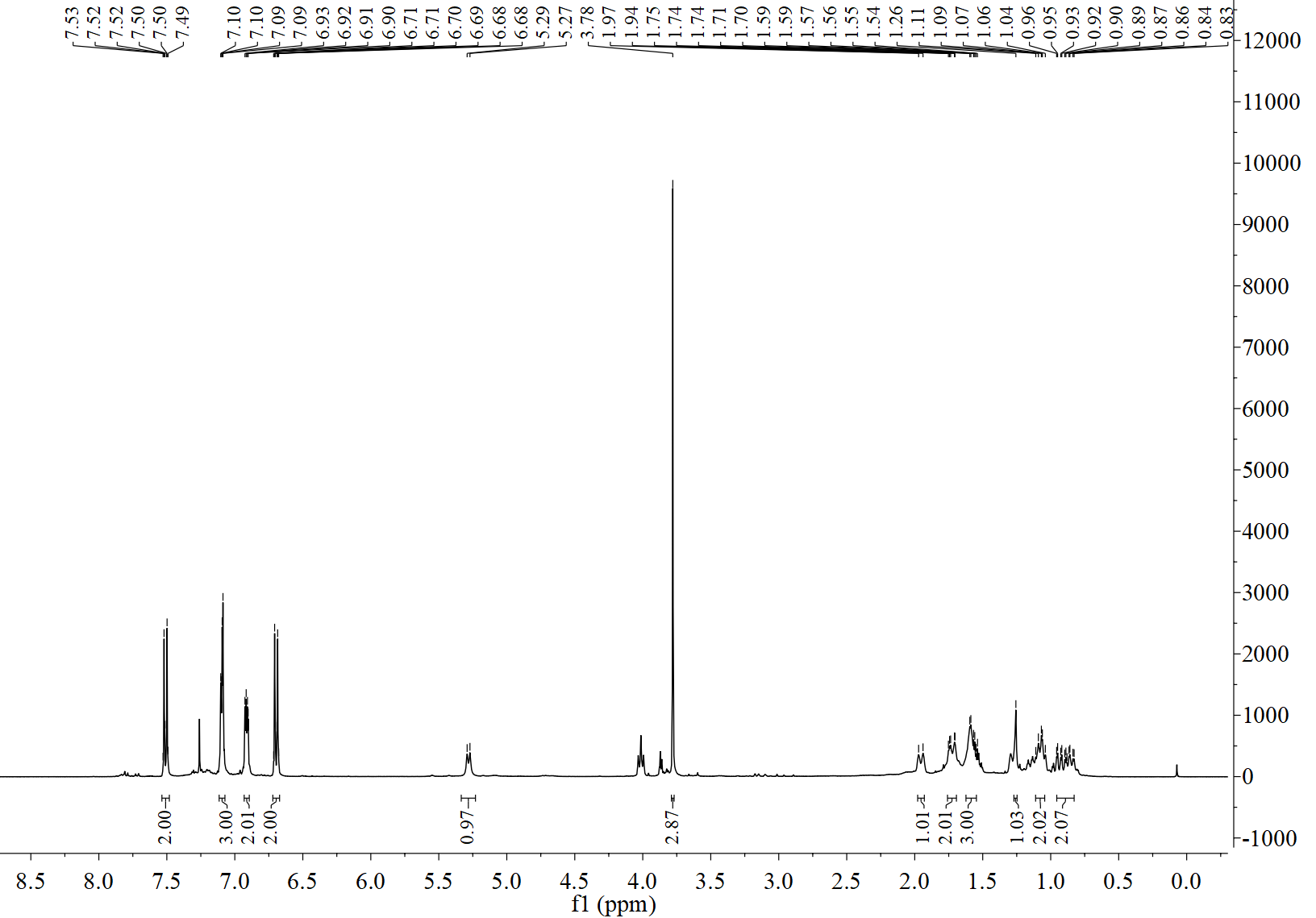


**Figure S44:** ^1^H-NMR (400 MHz, CDCl_3_) of *N*-cyclohexyl(phenyl)methyl-4-methoxybenzenesulfonamide (**3p**).


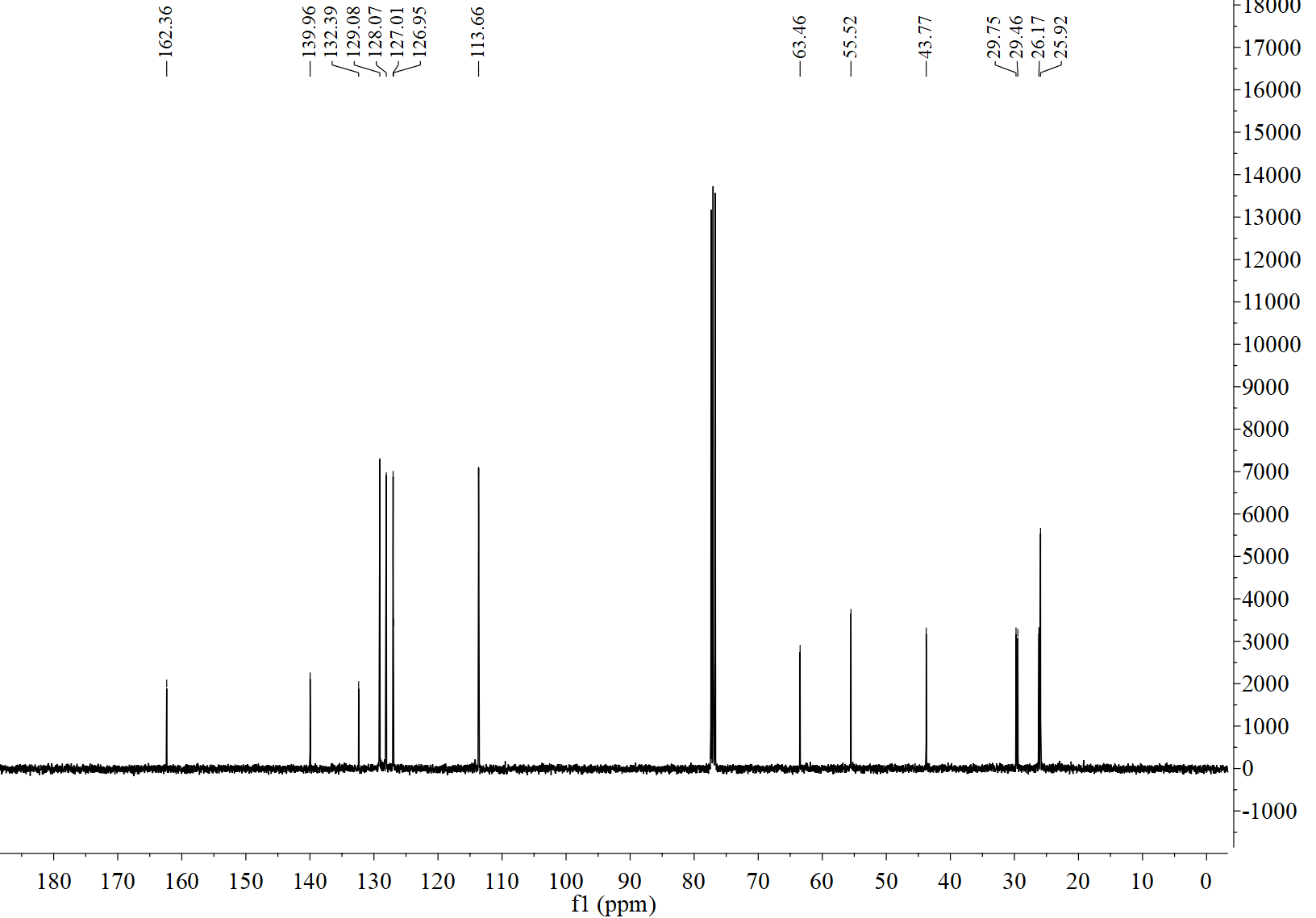


**Figure S45:** ^13^C-NMR (101 MHz, CDCl_3_) of *N*-cyclohexyl(phenyl)methyl-4-methoxybenzenesulfonamide (**3p**).


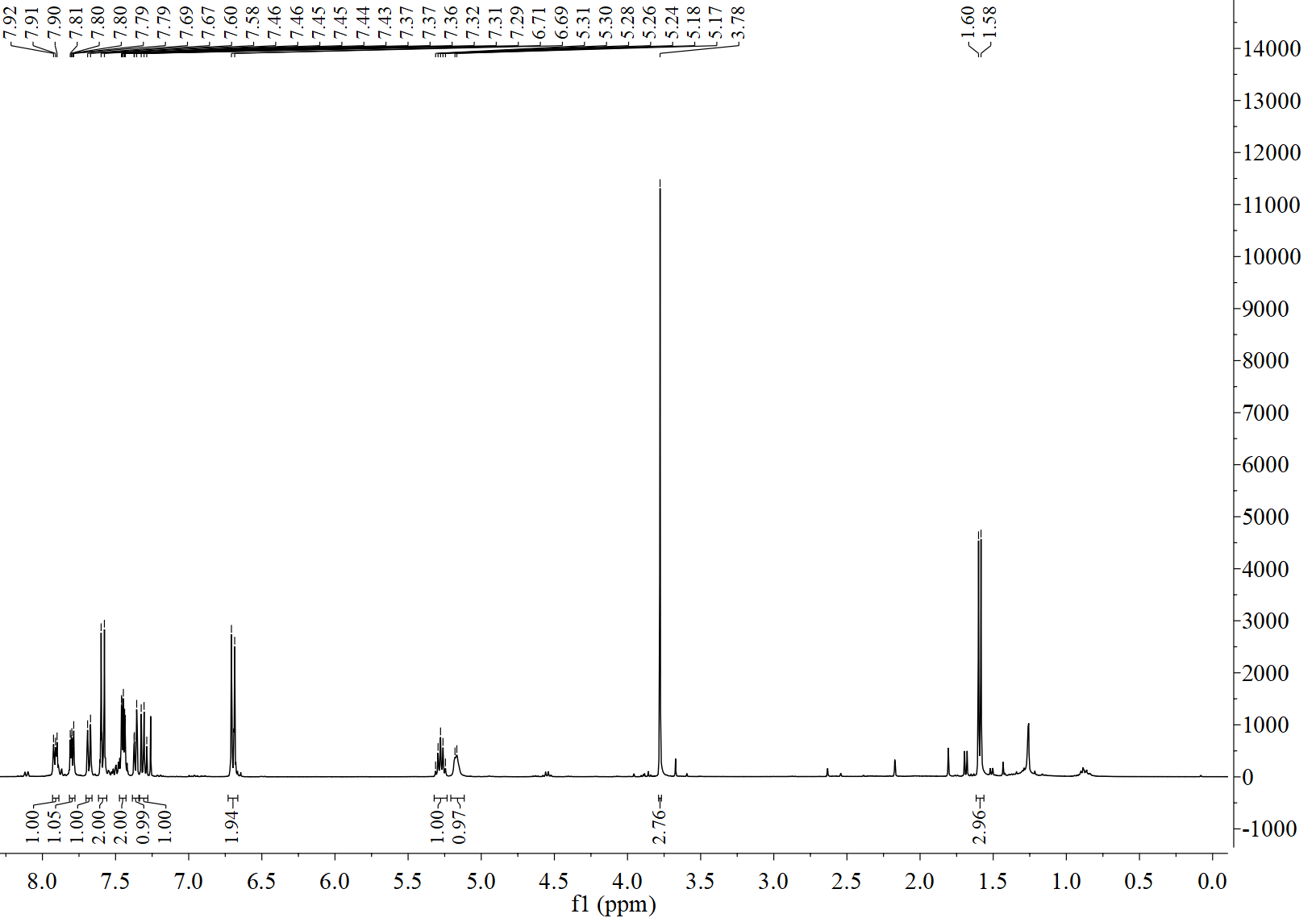


**Figure S46:** ^1^H-NMR (400 MHz, CDCl_3_) of 4-methoxy-*N*-(1-napthalen-1-yl)ethyl)benzenesulfonamide (**3q**).


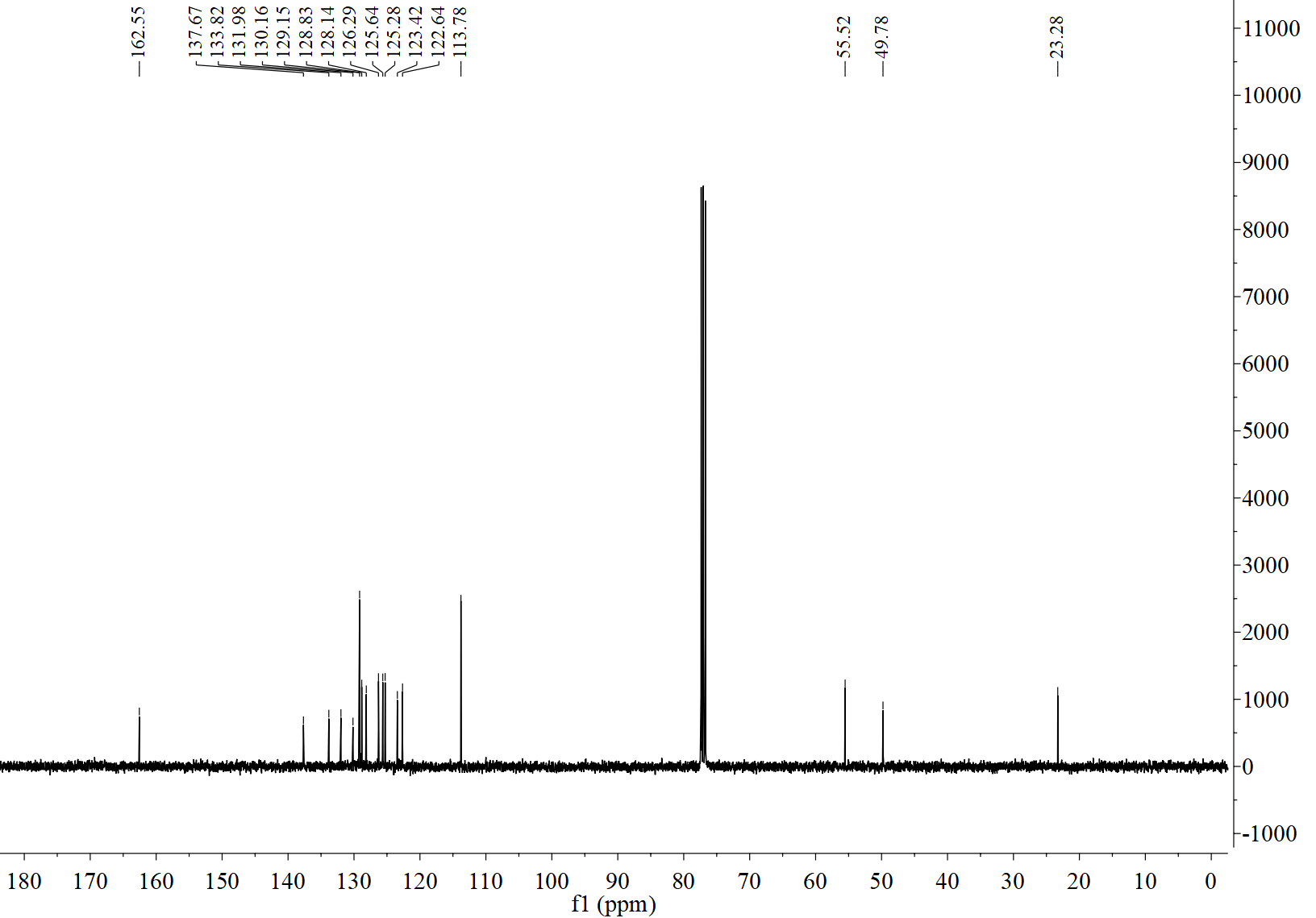


**Figure S47:** ^13^C-NMR (101 MHz, CDCl_3_) of 4-methoxy-*N*-(1-napthalen-1-yl)ethyl)benzenesulfonamide (**3q**).


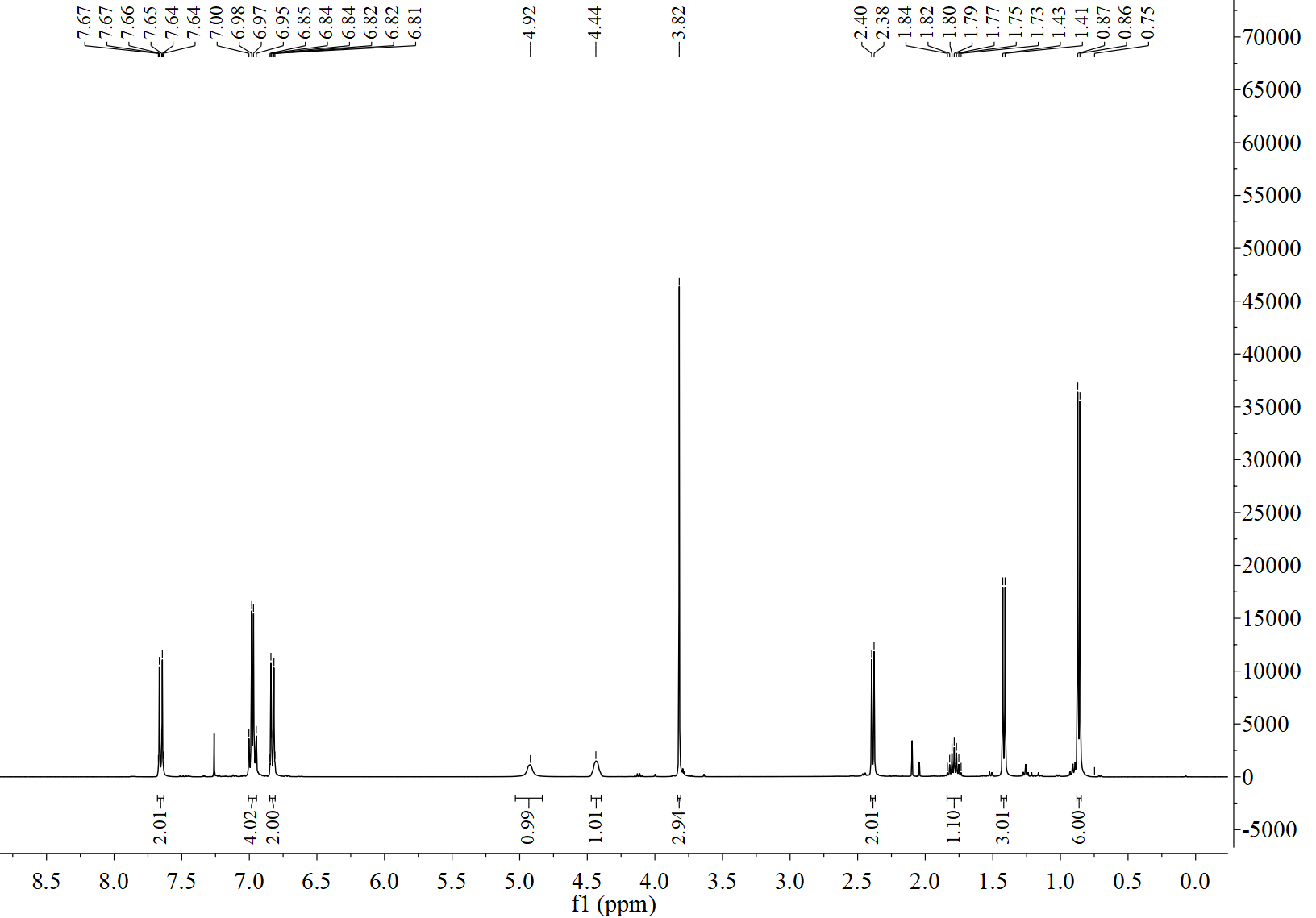


**Figure S48:** ^1^H-NMR (400 MHz, CDCl_3_) of *N*-(1-(4-isobutylphenyl)ethyl)-4-methylbenzenesulfonamide (**3r**).


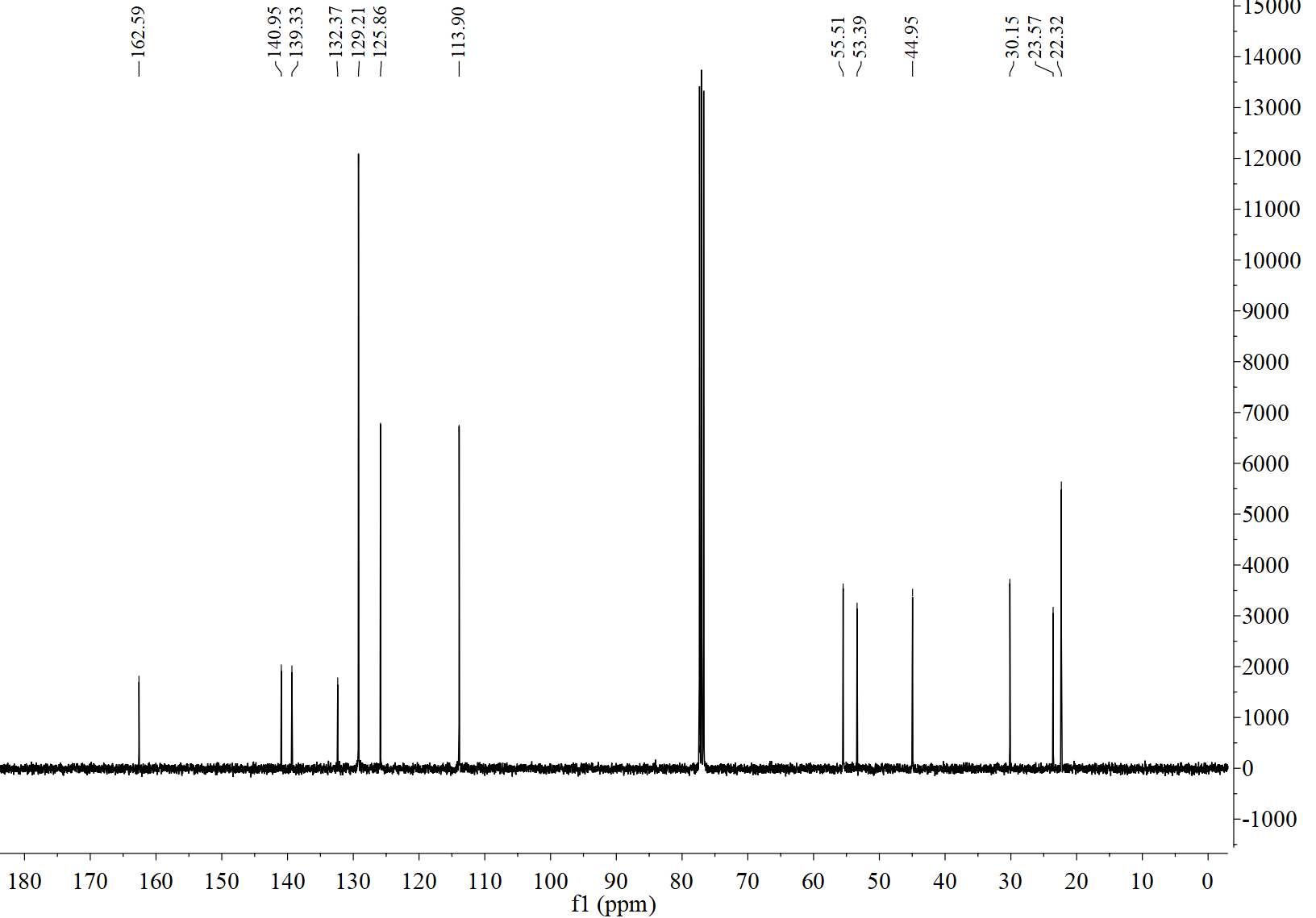


**Figure S49:** ^13^C-NMR (101 MHz, CDCl_3_) of *N*-(1-(4-isobutylphenyl)ethyl)-4-methylbenzenesulfonamide (**3r**).


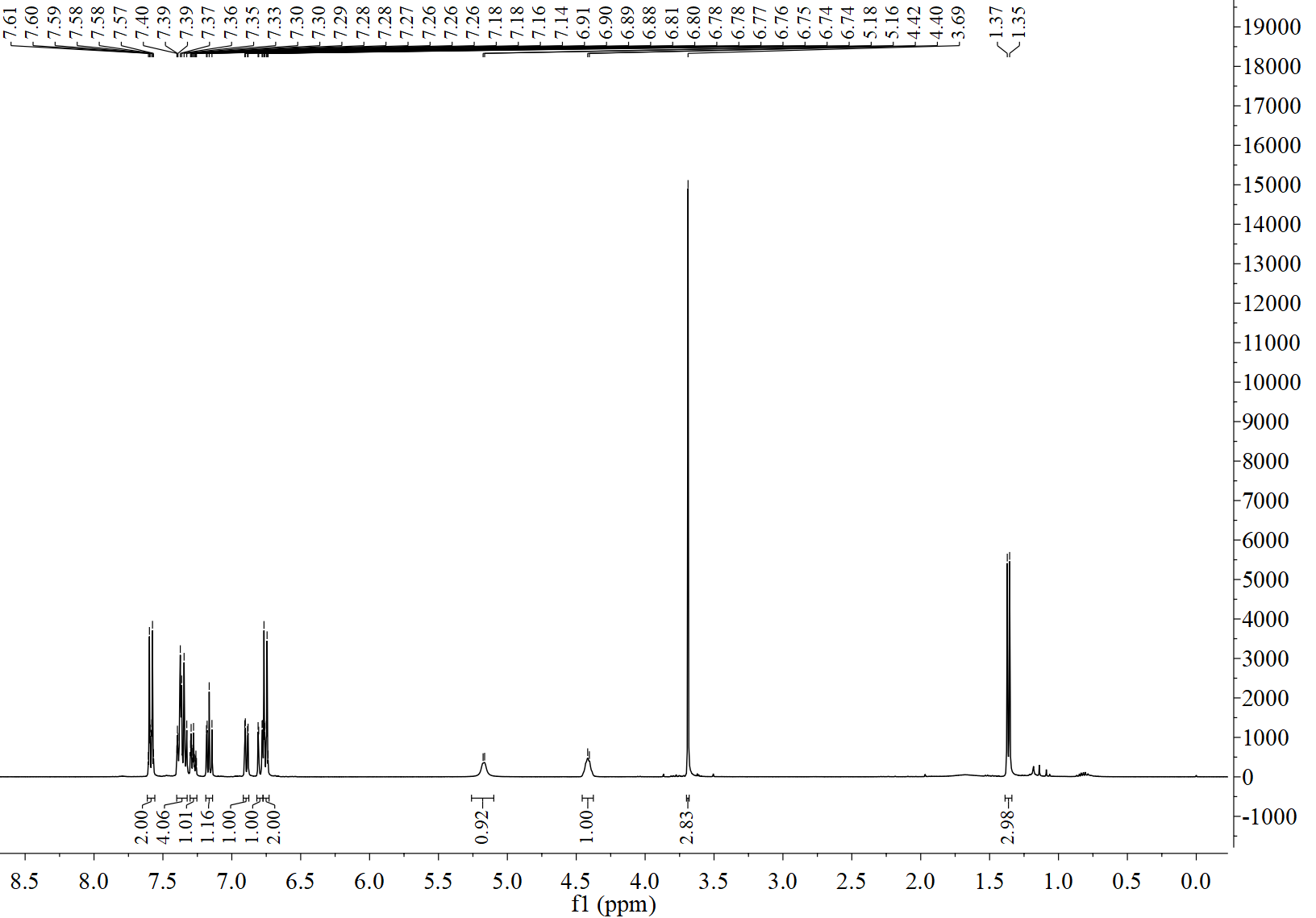


**Figure S50:** ^1^H-NMR (400 MHz, CDCl_3_) of *N*-(1-(2-fluoro-[1,1’biphenyl]-4-yl)ethyl)-4-methylbenzenesulfonamide (**3s**).


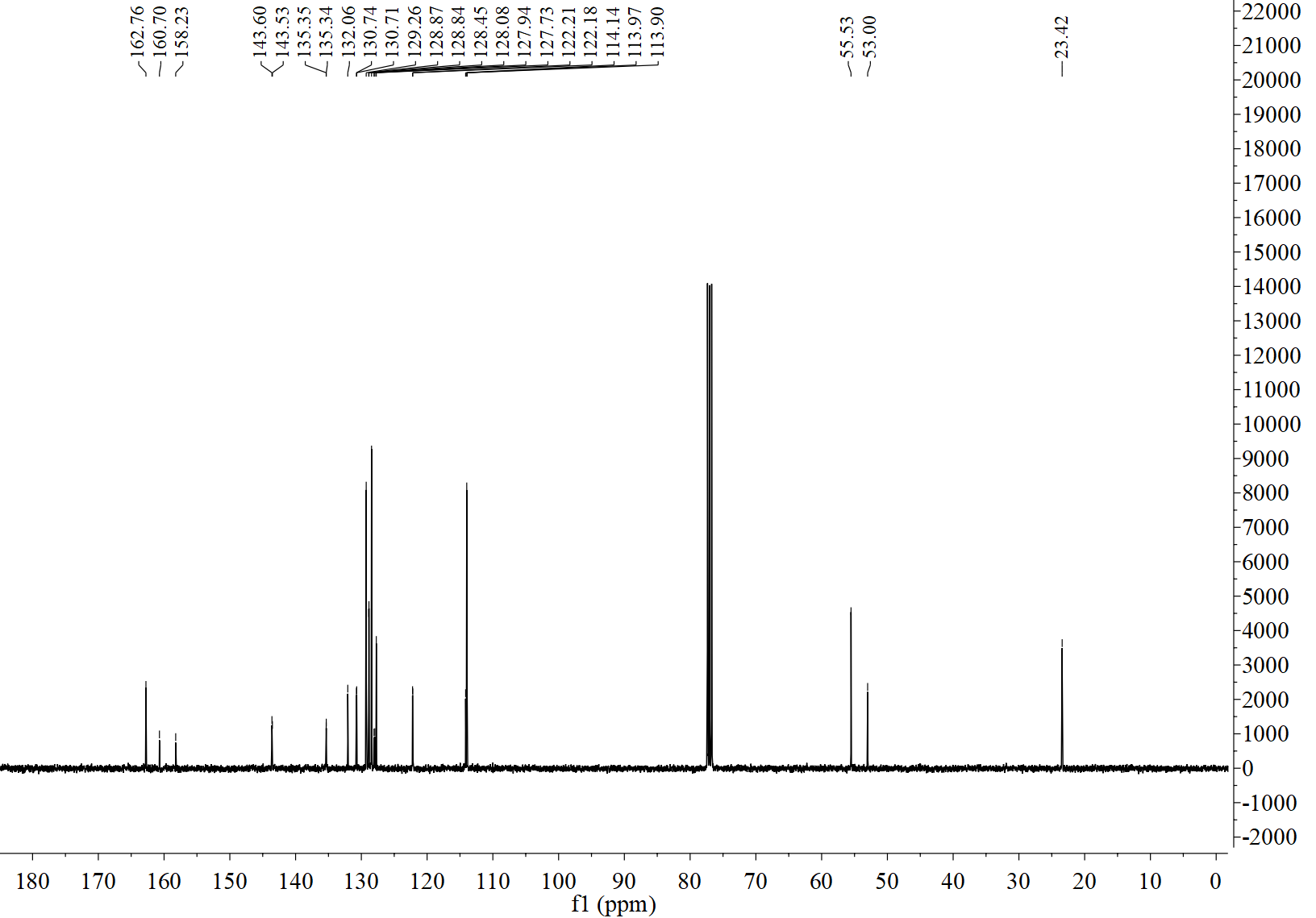


**Figure S51:** ^13^C-NMR (101 MHz, CDCl_3_) of *N*-(1-(2-fluoro-[1,1’biphenyl]-4-yl)ethyl)-4-methylbenzenesulfonamide (**3s**).


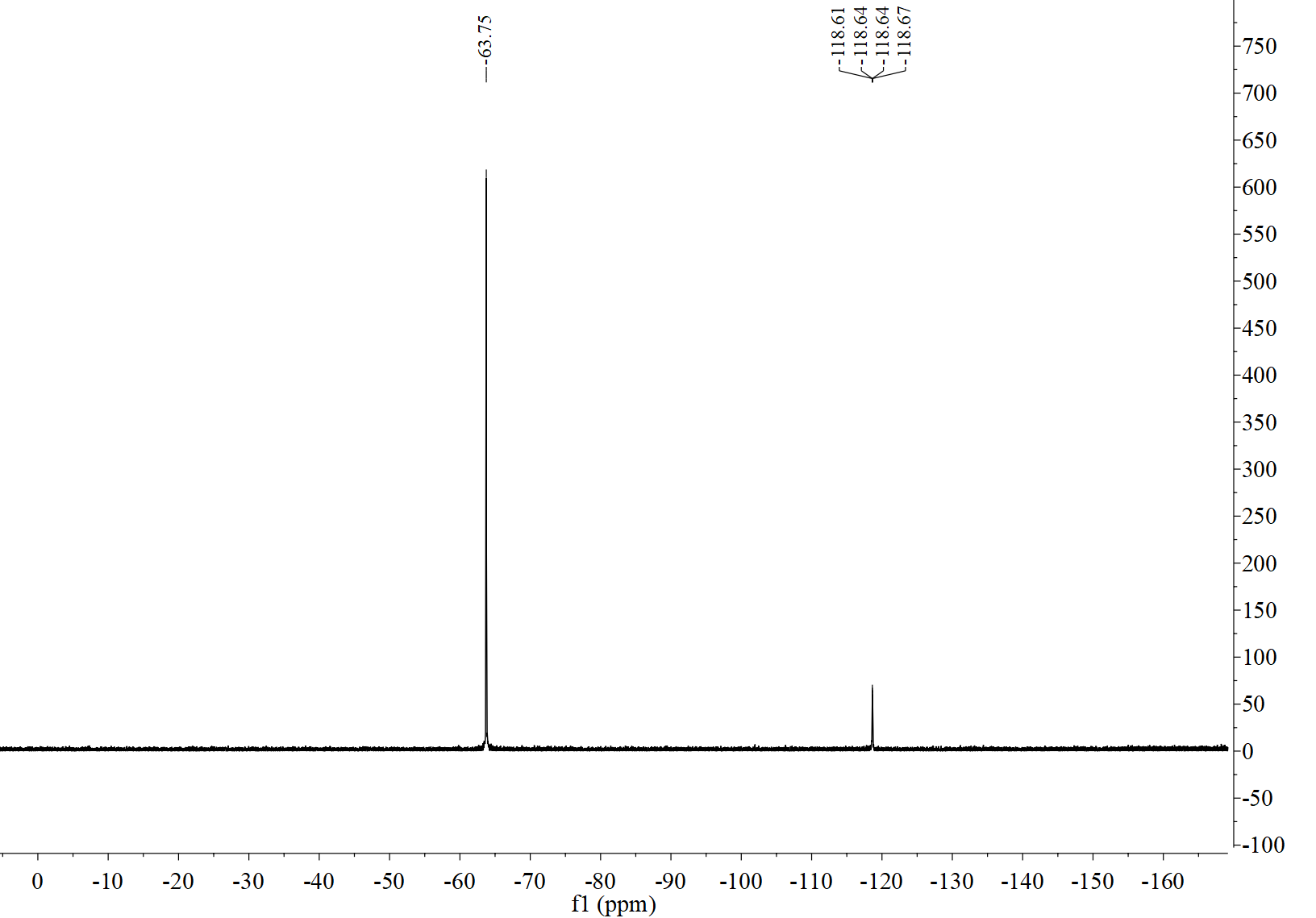


PhCF_3_

**Figure S52:** ^19^F-NMR (376 MHz, CDCl_3_) of *N*-(1-(2-fluoro-[1,1’biphenyl]-4-yl)ethyl)-4-methylbenzenesulfonamide (**3s**) with PhCF_3_ as internal standard.


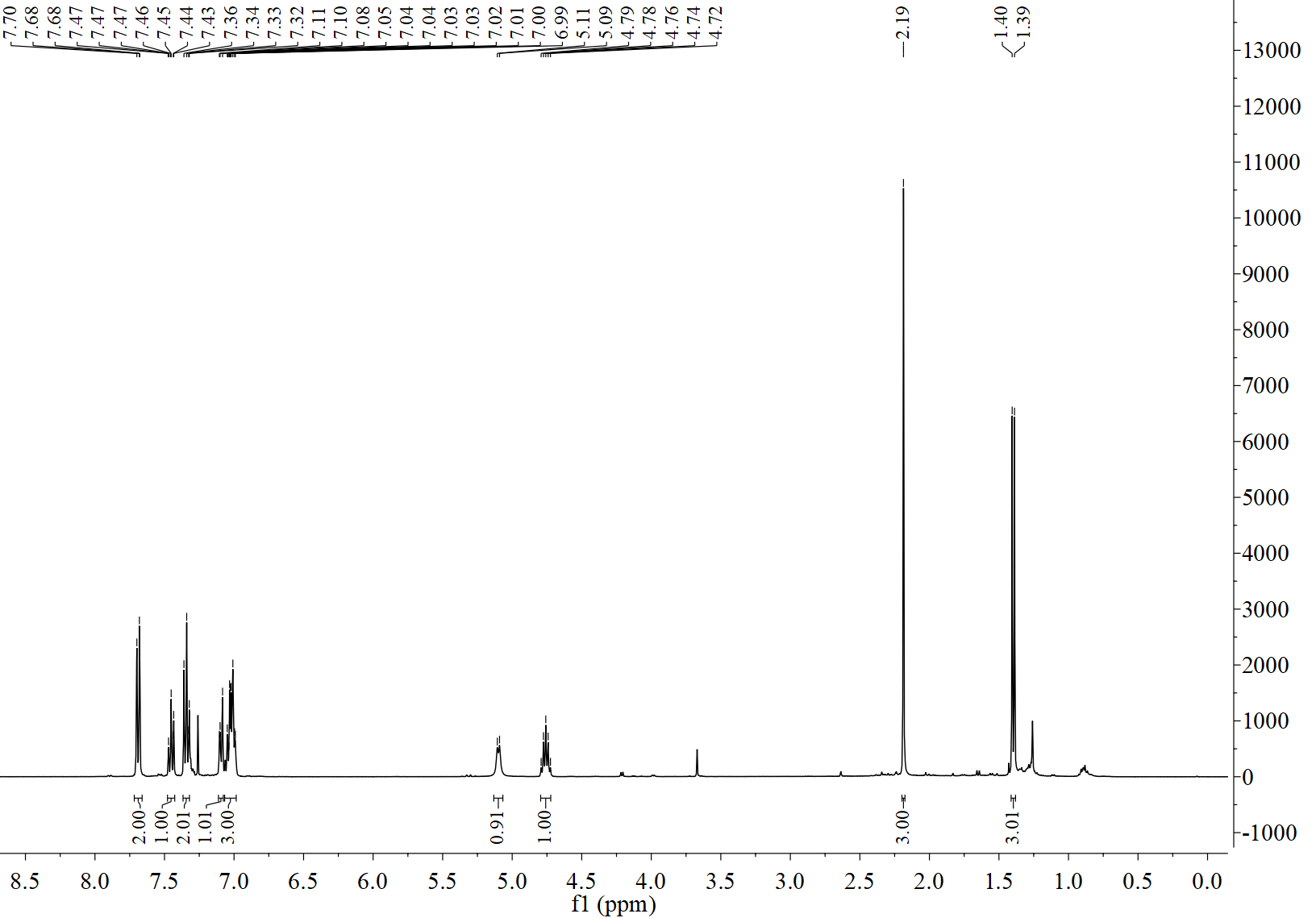


**Figure S53:** ^1^H-NMR (400 MHz, CDCl_3_) of *N*-(1-(*o*-tolyl)ethyl)benzenesulfonamide (**4a**).


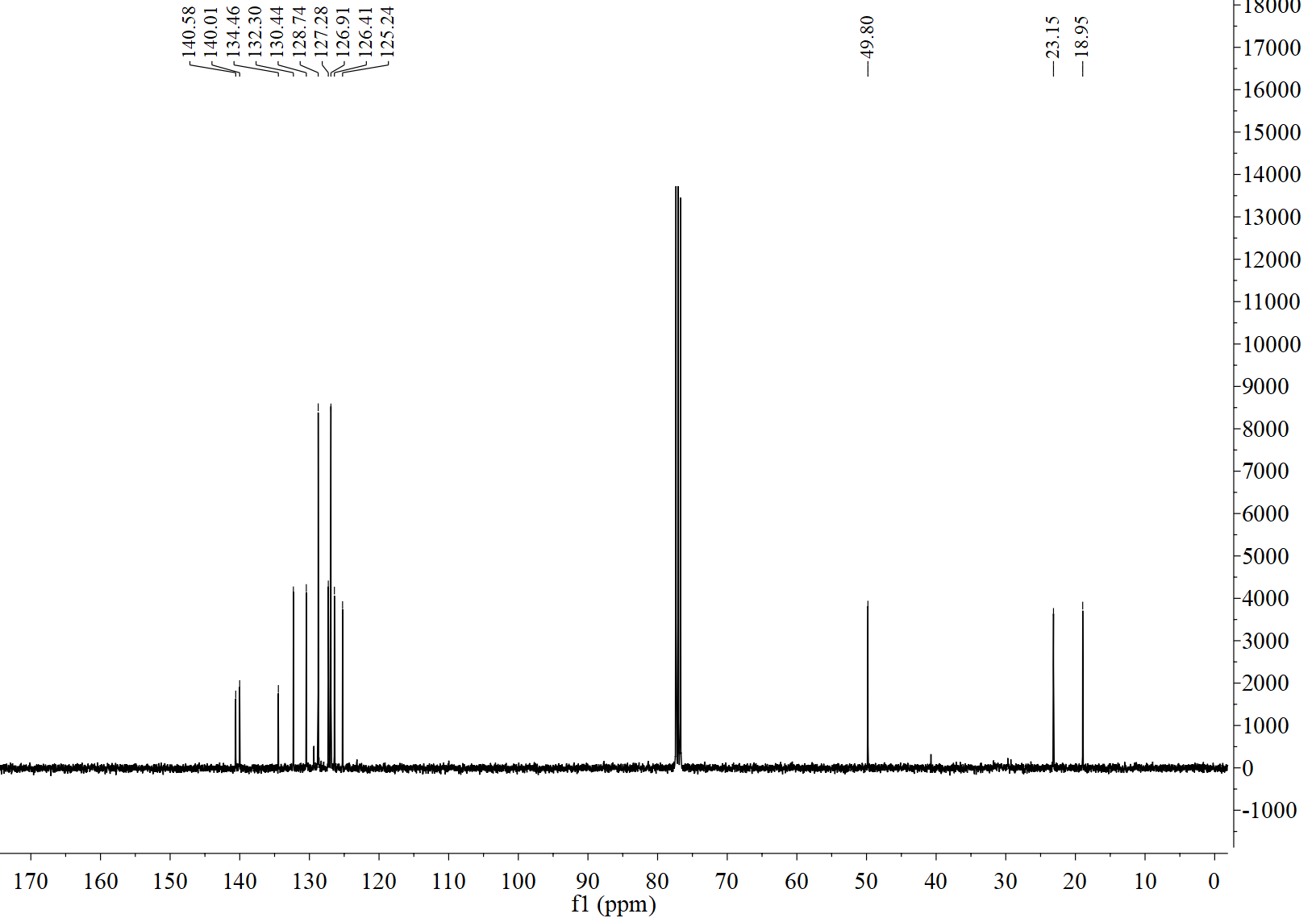


**Figure S54:** ^13^C-NMR (101 MHz, CDCl_3_) of *N*-(1-(*o*-tolyl)ethyl)benzenesulfonamide (**4a**).


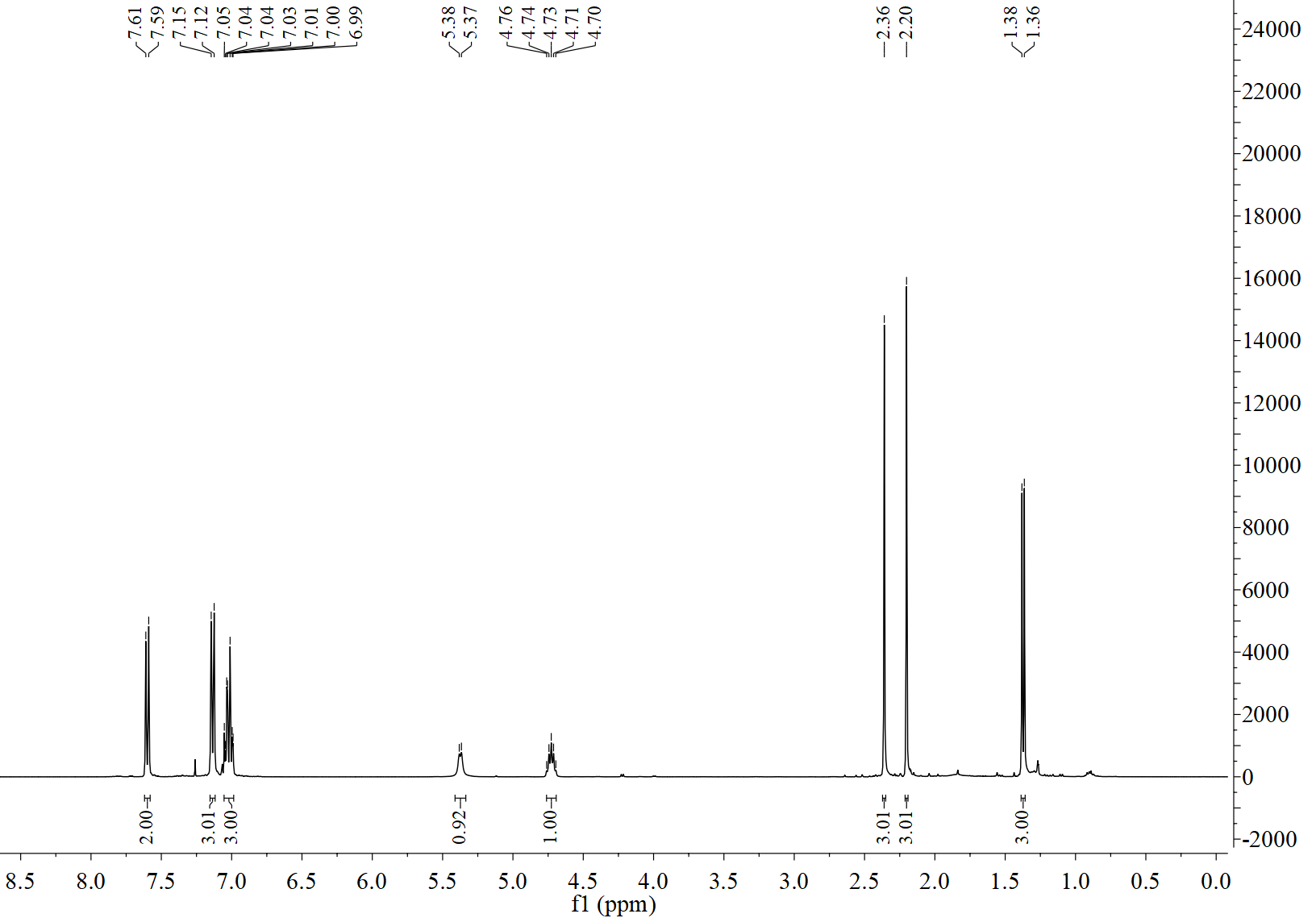


**Figure S55:** ^1^H-NMR (400 MHz, CDCl_3_) of 4-methyl-*N*-(1-(*o*-tolyl)ethyl)benzenesulfonamide (**4b**).


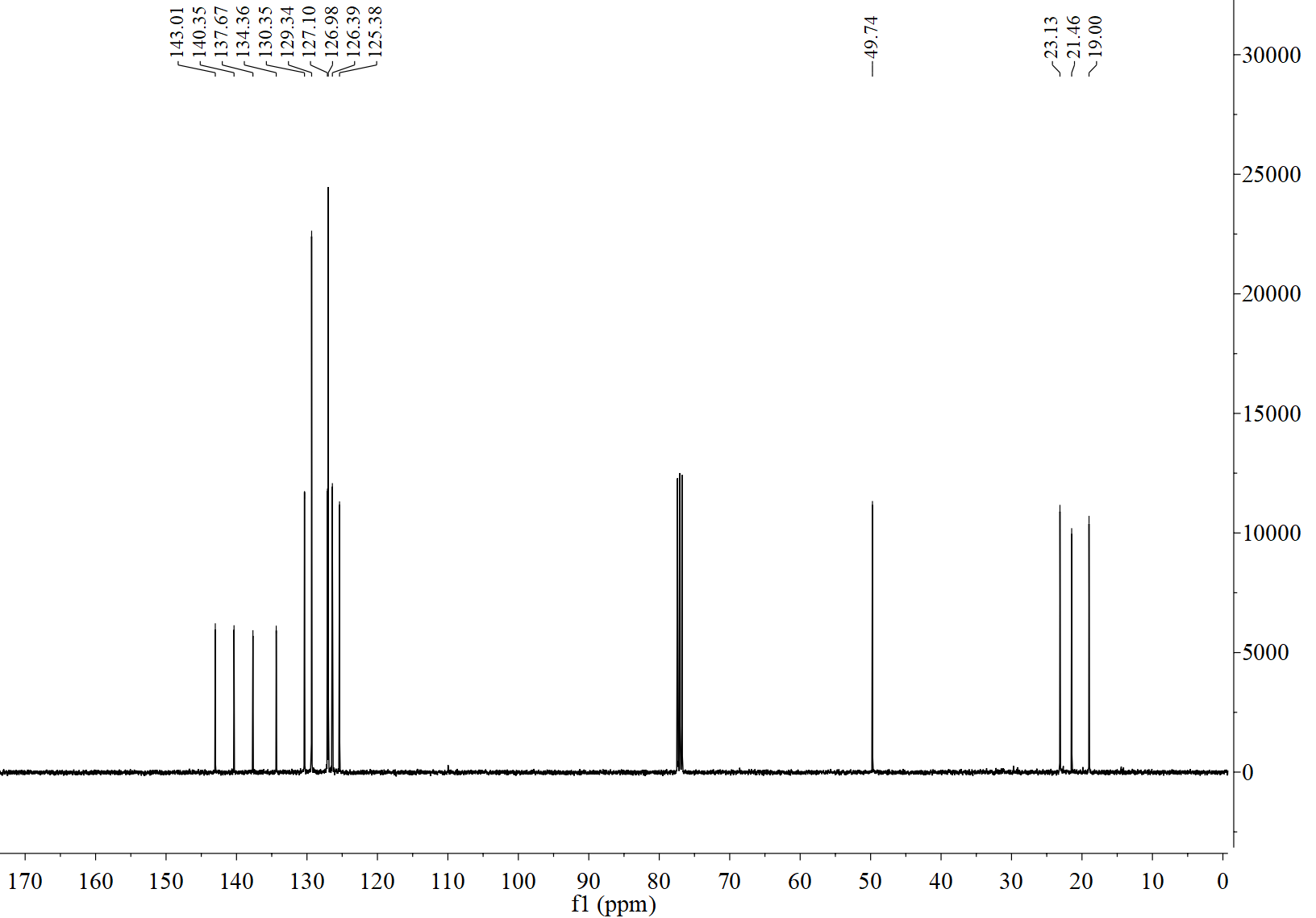


**Figure S56:** ^13^C-NMR (101 MHz, CDCl_3_) of 4-methyl-*N*-(1-(*o*-tolyl)ethyl)benzenesulfonamide (**4b**).


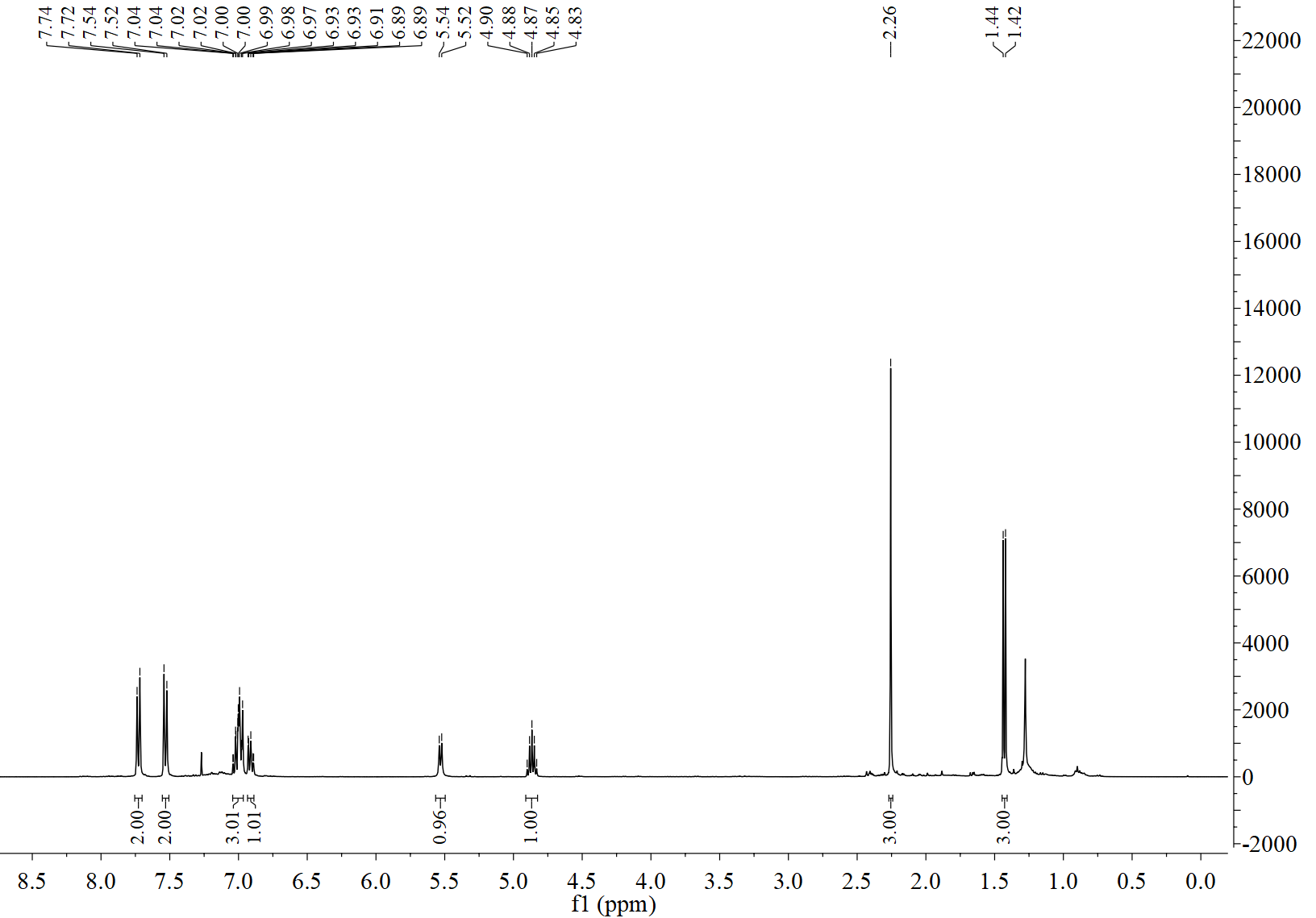


**Figure S57:** ^1^H-NMR (400 MHz, CDCl_3_) of *N*-(1-(*o*-tolyl)ethyl)-4-(trifluoromethyl)benzenesulfonamide (**4c**).


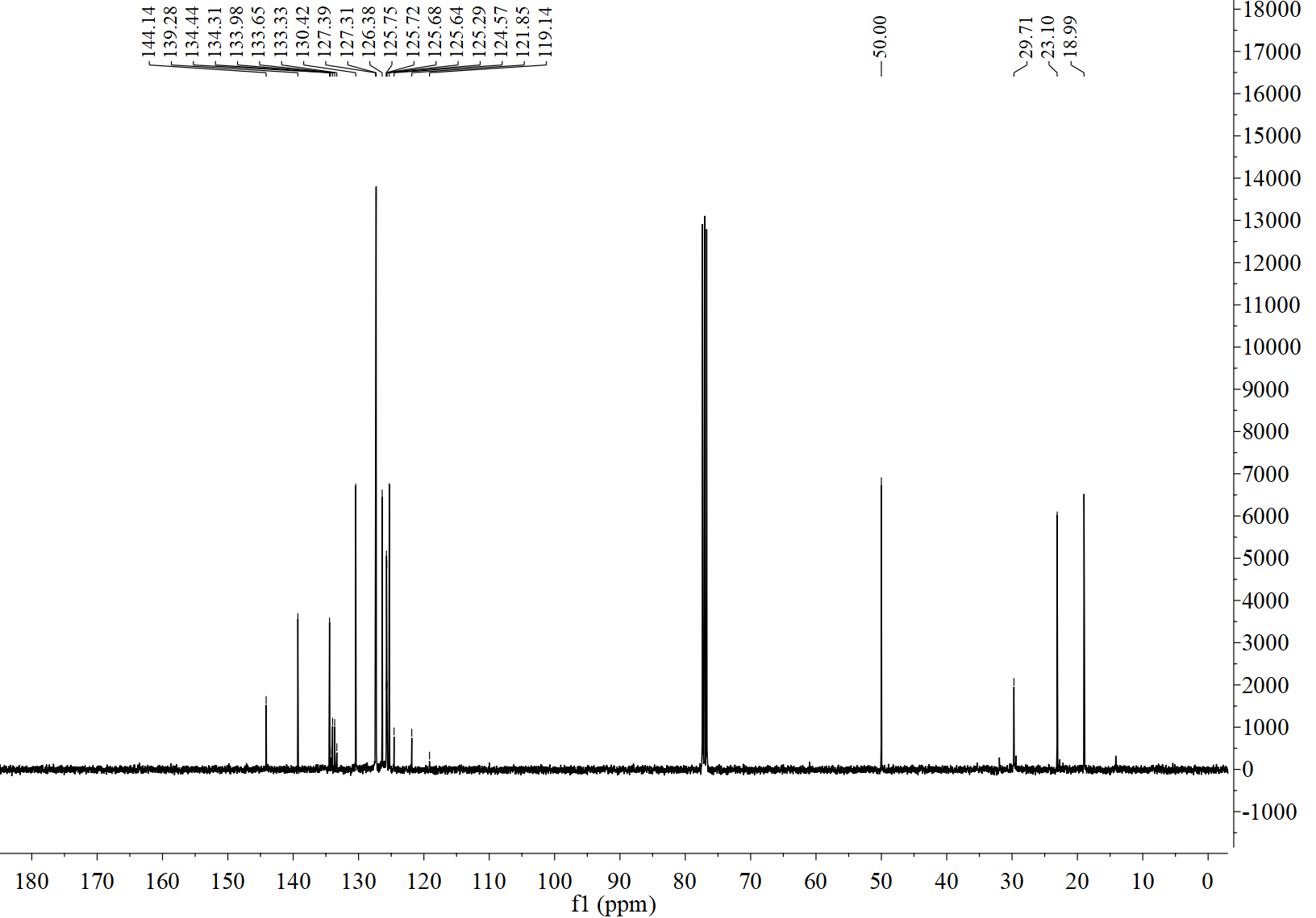


**Figure S58:** ^13^C-NMR (101 MHz, CDCl_3_) of *N*-(1-(*o*-tolyl)ethyl)-4-(trifluoromethyl)benzenesulfonamide (**4c**).


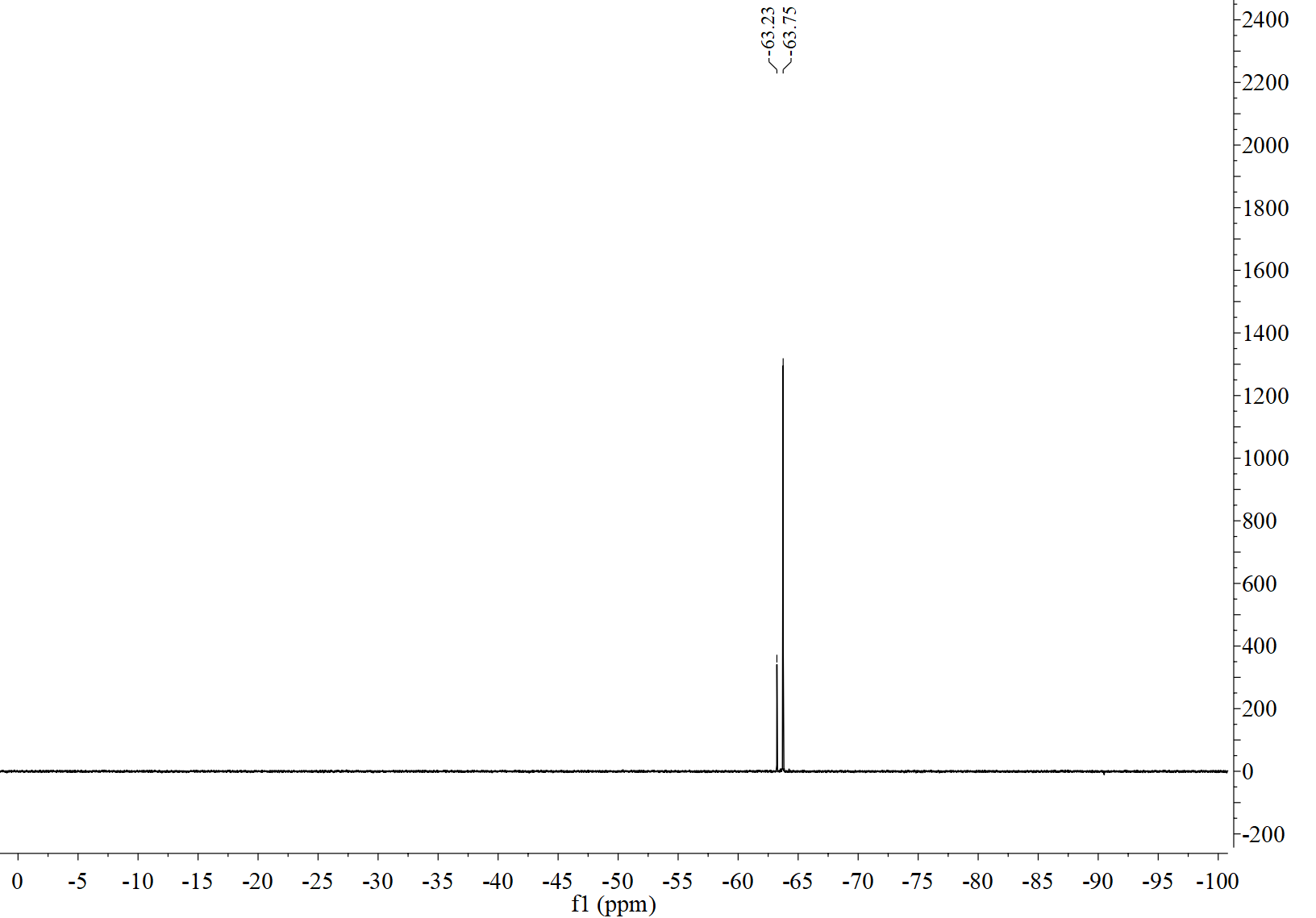


PhCF_3_

**Figure S59:** ^19^F-NMR (376 MHz, CDCl_3_) of *N*-(1-(*o*-tolyl)ethyl)-4-(trifluoromethyl)benzenesulfonamide (**4c**) with PhCF_3_ as internal standard.


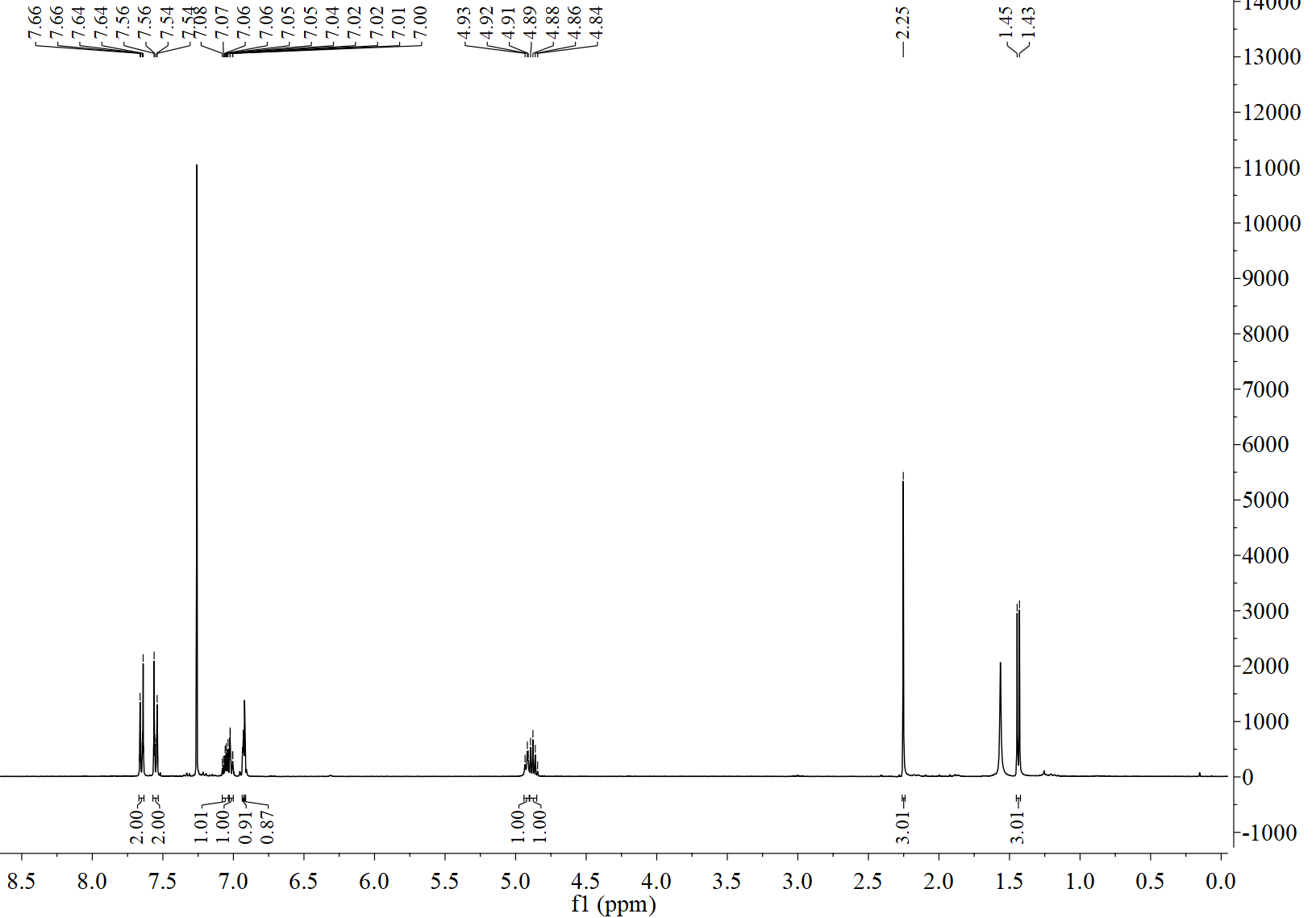


**Figure S60:** ^1^H-NMR (400 MHz, CDCl_3_) of 4-cyano-*N*-(1-(*o*-tolyl)ethyl)benzenesulfonamide (**4d**).


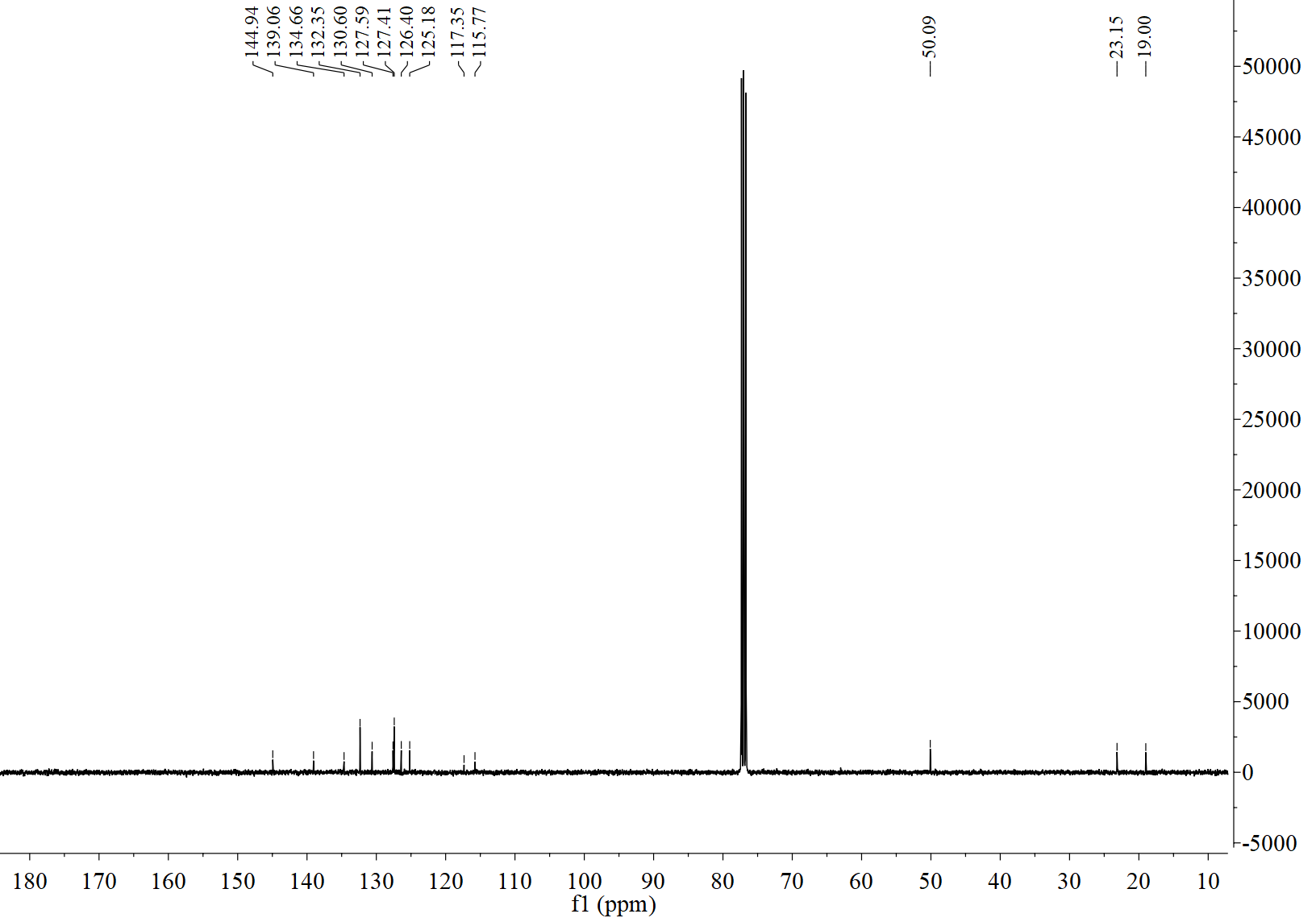


**Figure S61:** ^13^C-NMR (101 MHz, CDCl_3_) of 4-cyano-*N*-(1-(*o*-tolyl)ethyl)benzenesulfonamide (**4d**).


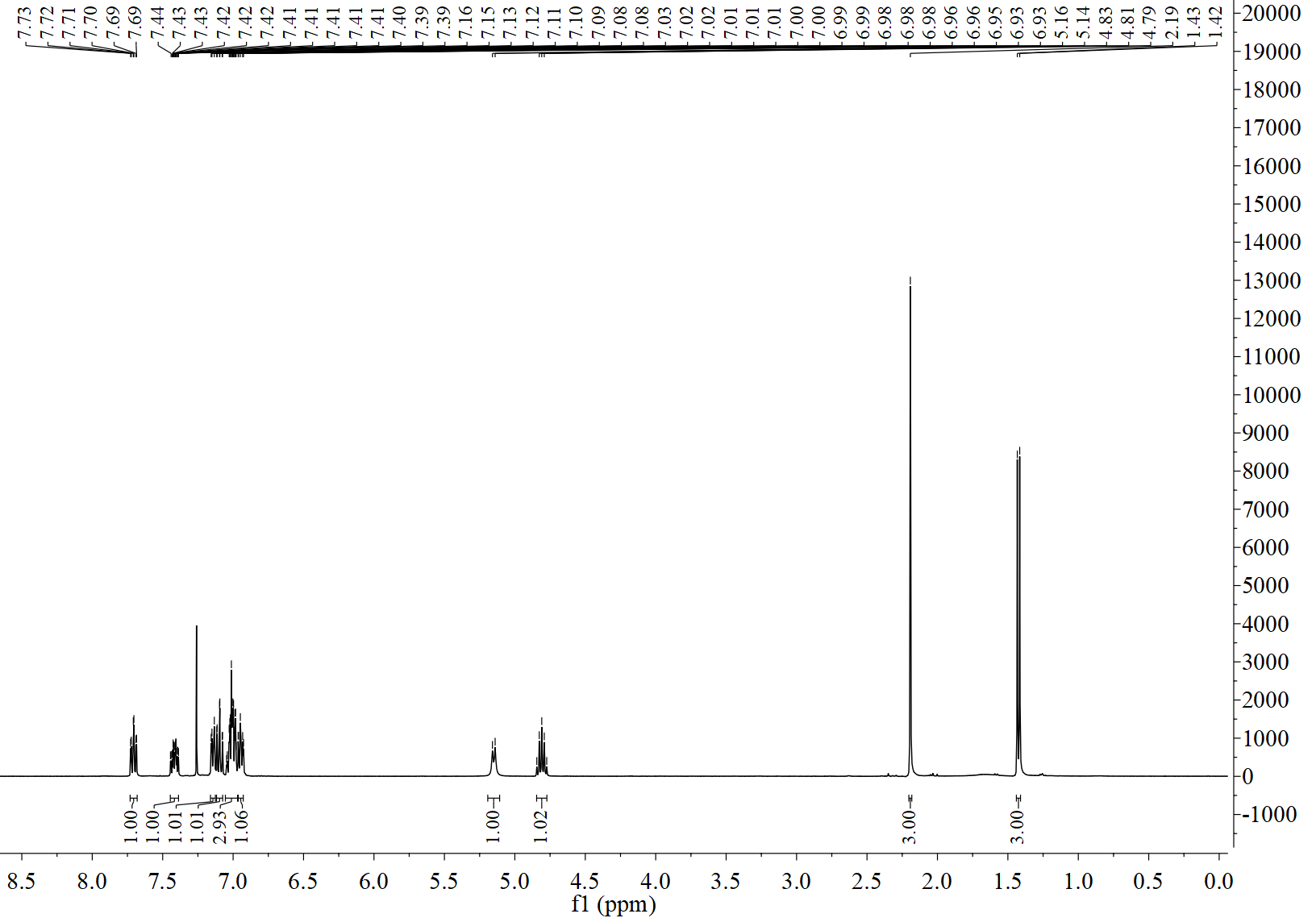


**Figure S62:** ^1^H-NMR (400 MHz, CDCl_3_) of 2-fluoro-*N*-(1-(*o*-tolyl)ethyl)benzenesulfonamide (**4e**).


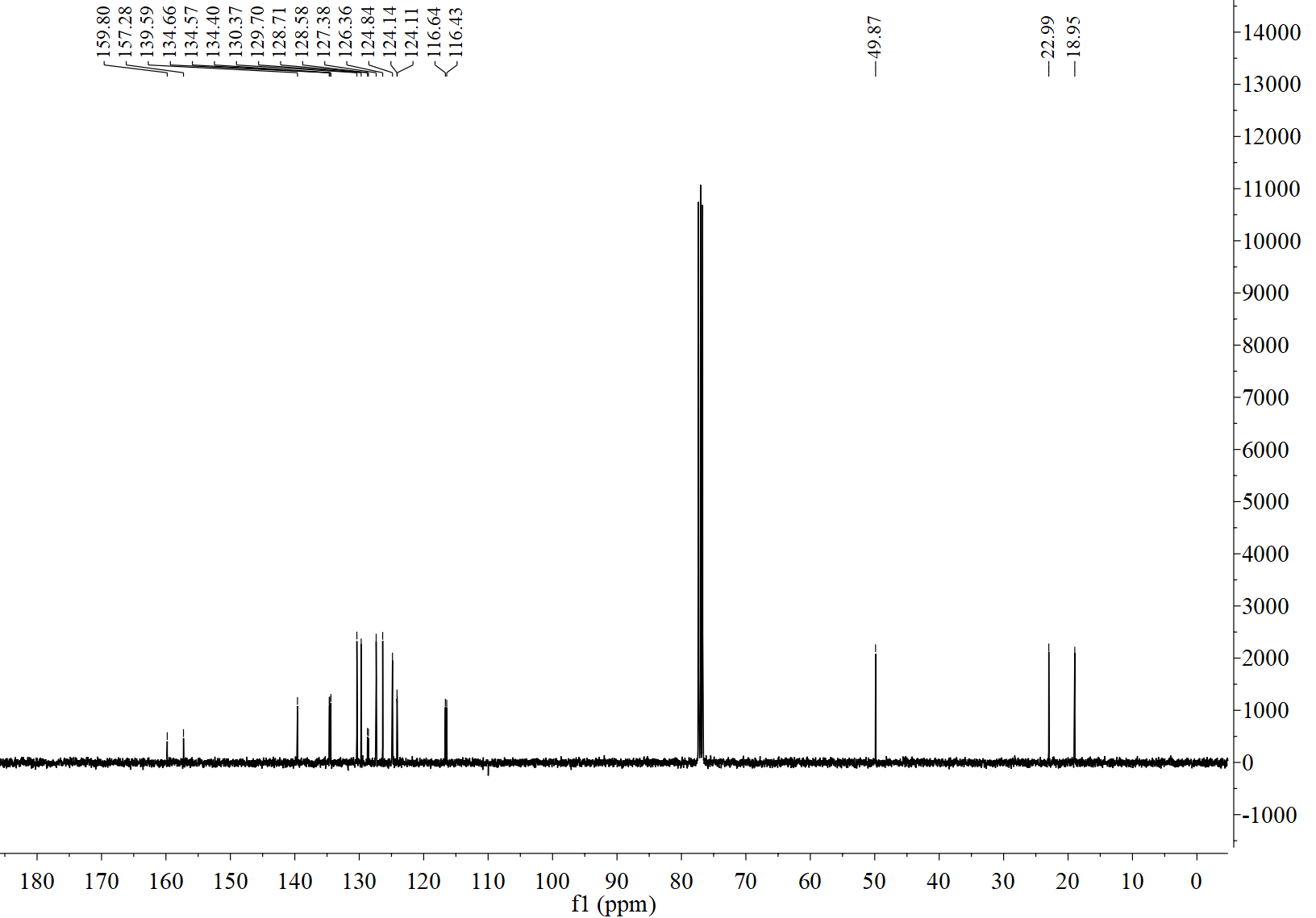


**Figure S63:** ^13^C-NMR (101 MHz, CDCl_3_) of 2-fluoro-*N*-(1-(*o*-tolyl)ethyl)benzenesulfonamide (**4e**).


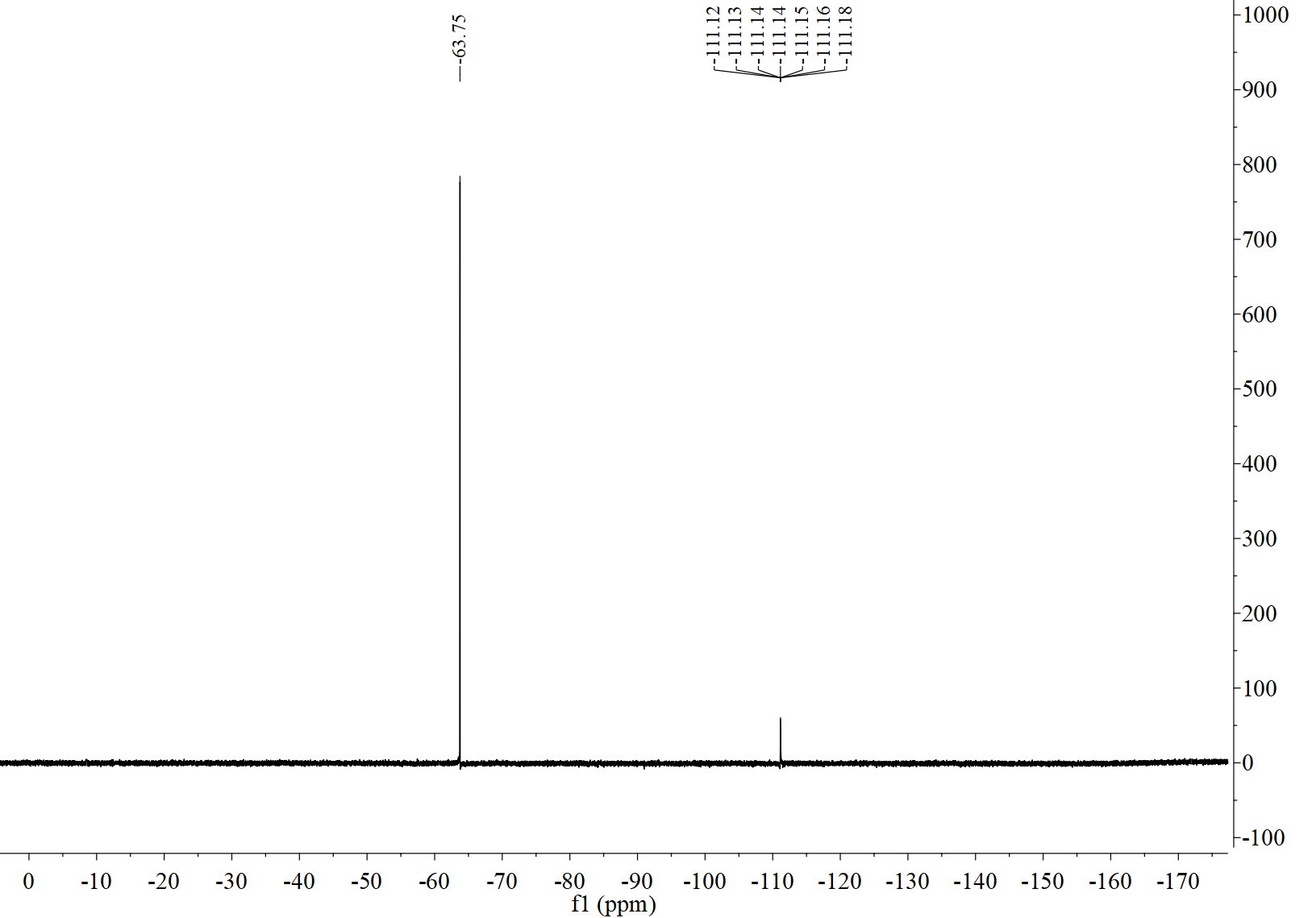


PhCF_3_

**Figure S64:** ^19^F-NMR (376 MHz, CDCl_3_) of 2-fluoro-*N*-(1-(*o*-tolyl)ethyl)benzenesulfonamide (**4e**) with PhCF_3_ as internal standard.


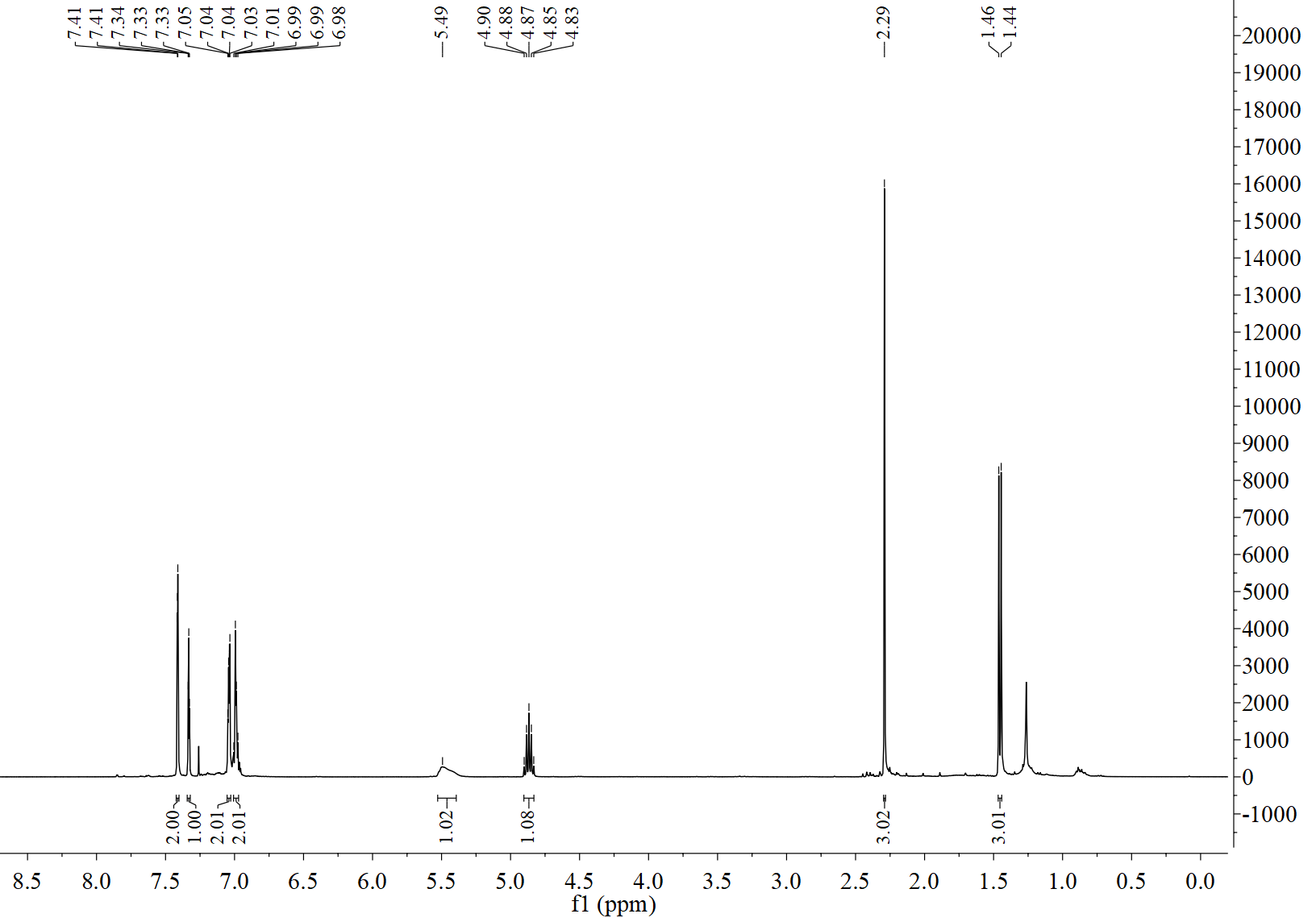


**Figure S65:** ^1^H-NMR (400 MHz, CDCl_3_) of 3,5-dichloro-*N*-(1-(*o*-tolyl)ethyl)benzenesulfonamide (**4f**).


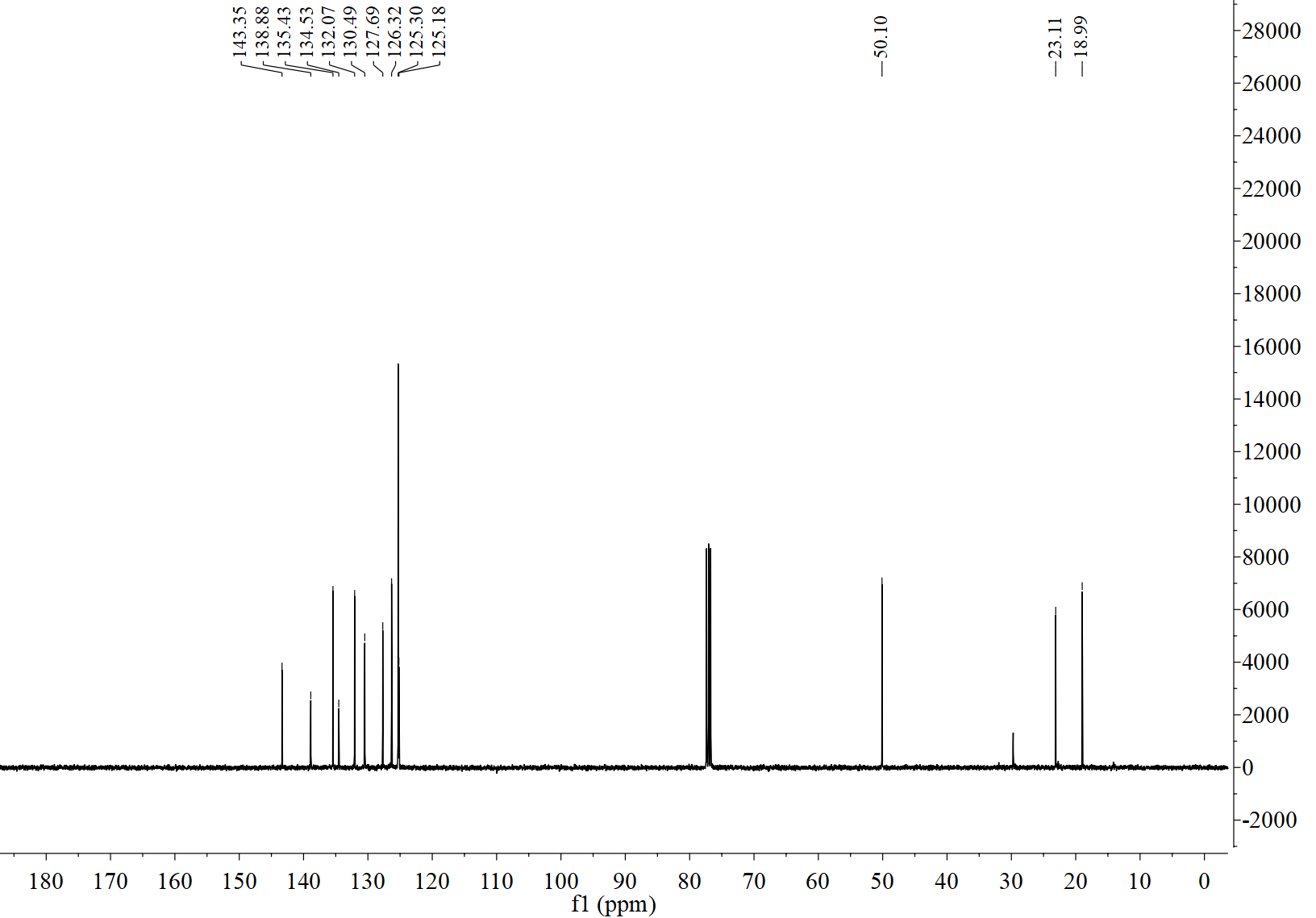


**Figure S66:** ^13^C-NMR (101 MHz, CDCl_3_) of 3,5-dichloro-*N*-(1-(*o*-tolyl)ethyl)benzenesulfonamide (**4f**).


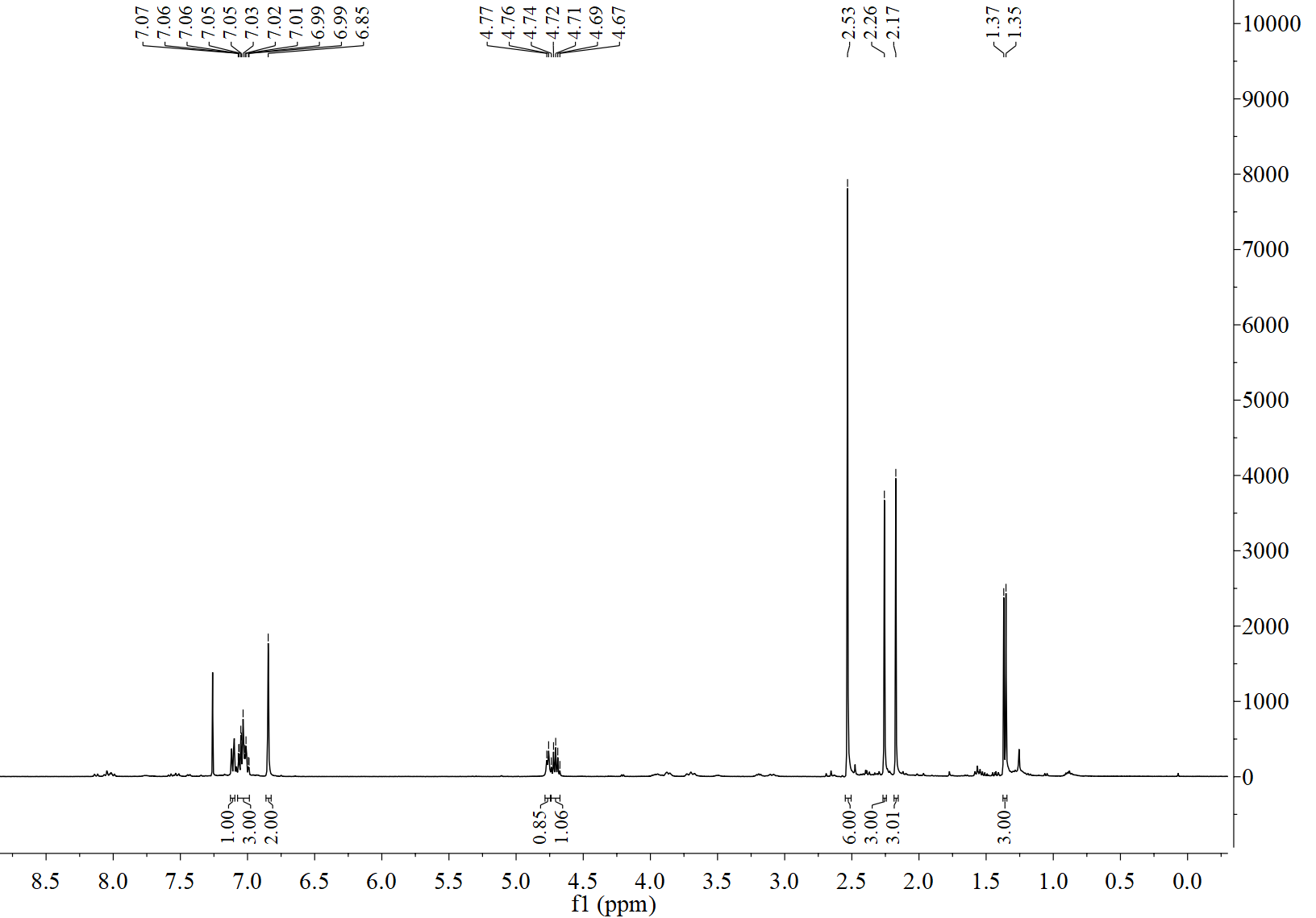


**Figure S67:** ^1^H-NMR (400 MHz, CDCl_3_) of 2,4,6-trimethyl-*N*-(1-(*o*-tolyl)ethyl)benzenesulfonamide (**4g**).


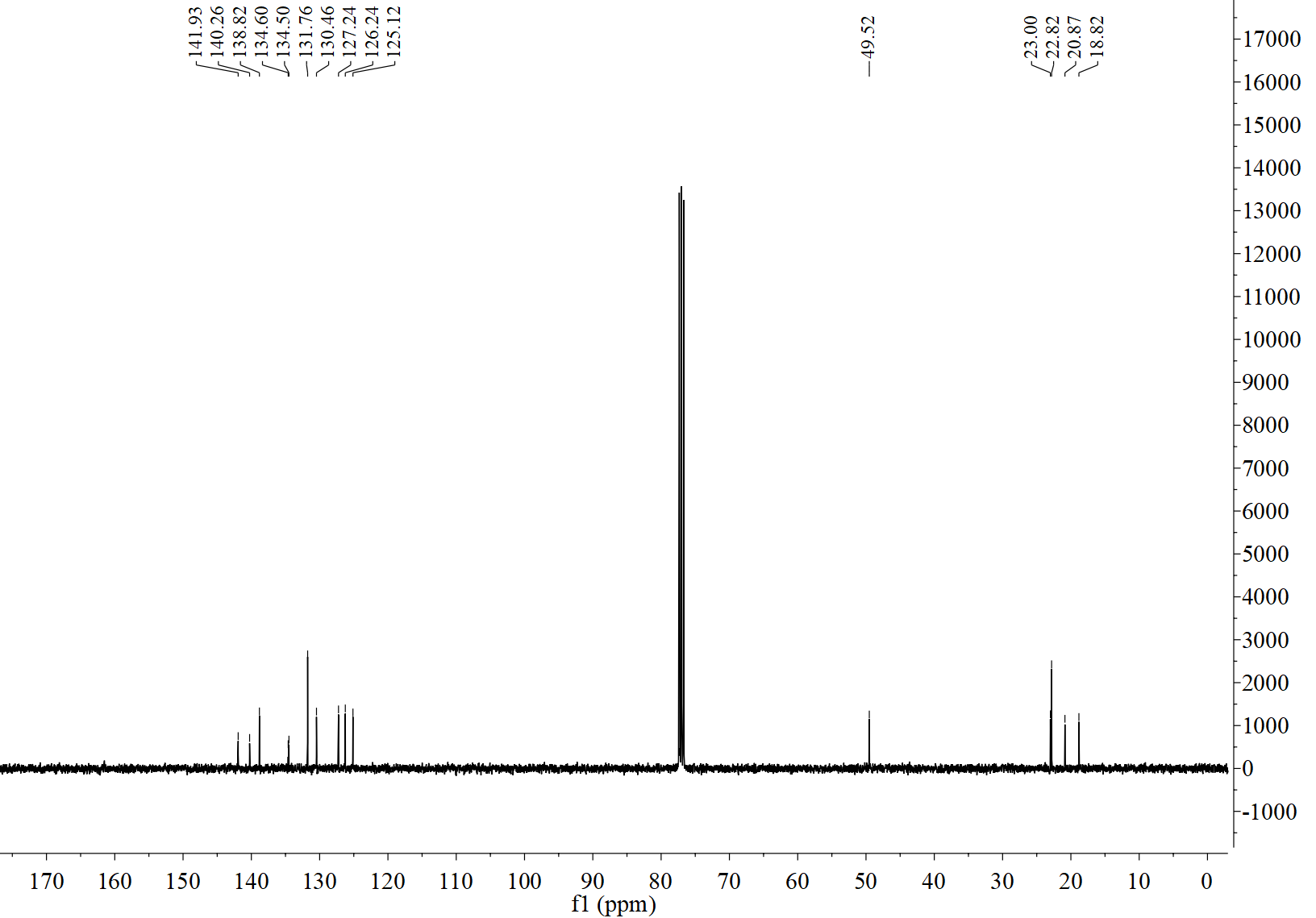


**Figure S68:** ^13^C-NMR (101 MHz, CDCl_3_) of 2,4,6-trimethyl-*N*-(1-(*o*-tolyl)ethyl)benzenesulfonamide (**4g**).


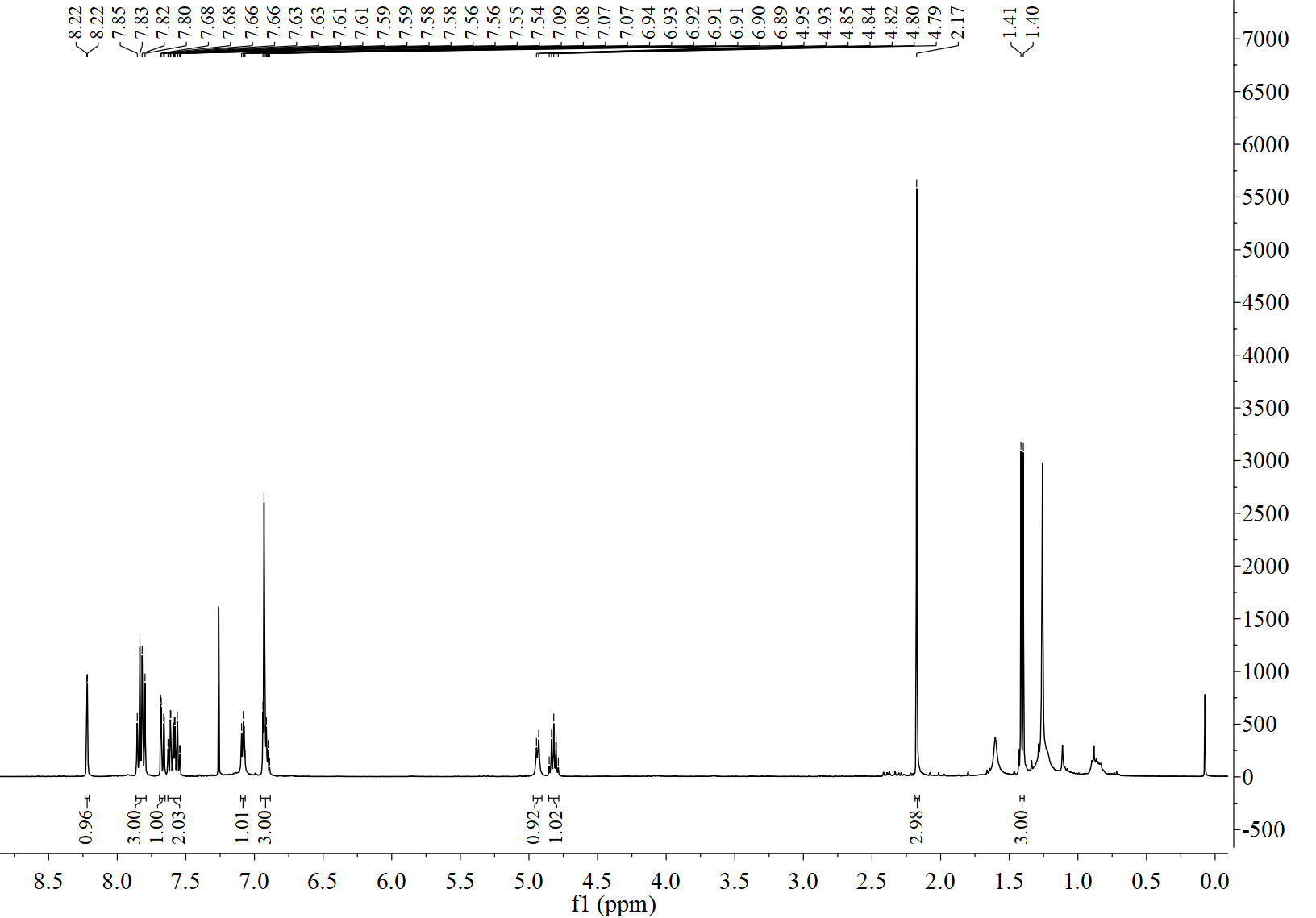


**Figure S69:** ^1^H-NMR (400 MHz, CDCl_3_) of *N*-(1-(*o*-tolyl)ethyl)naphthalene-1-sulfonamide (**4h**).


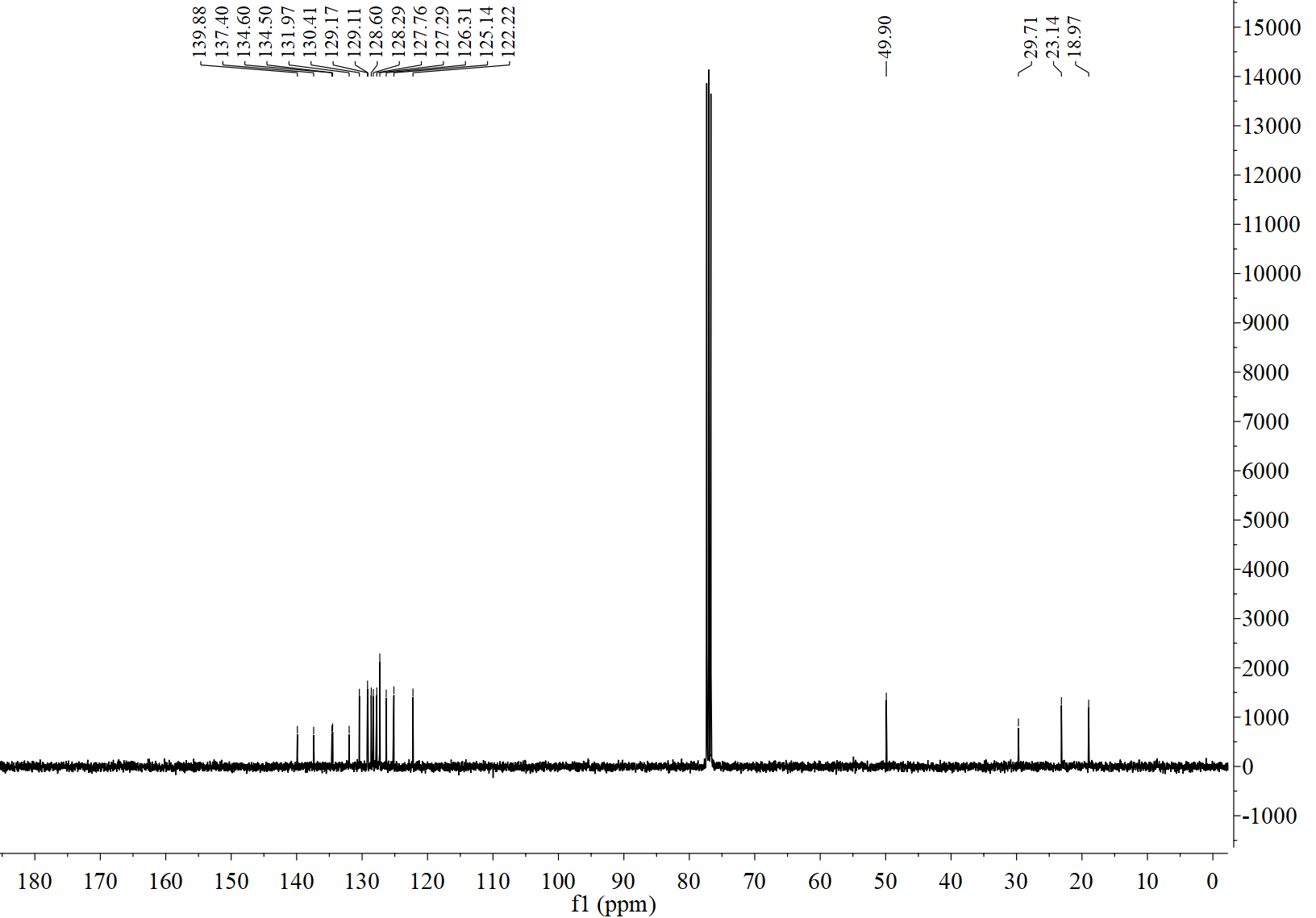


**Figure S70:** ^13^C-NMR (101 MHz, CDCl_3_) of *N*-(1-(*o*-tolyl)ethyl)naphthalene-1-sulfonamide (**4h**).


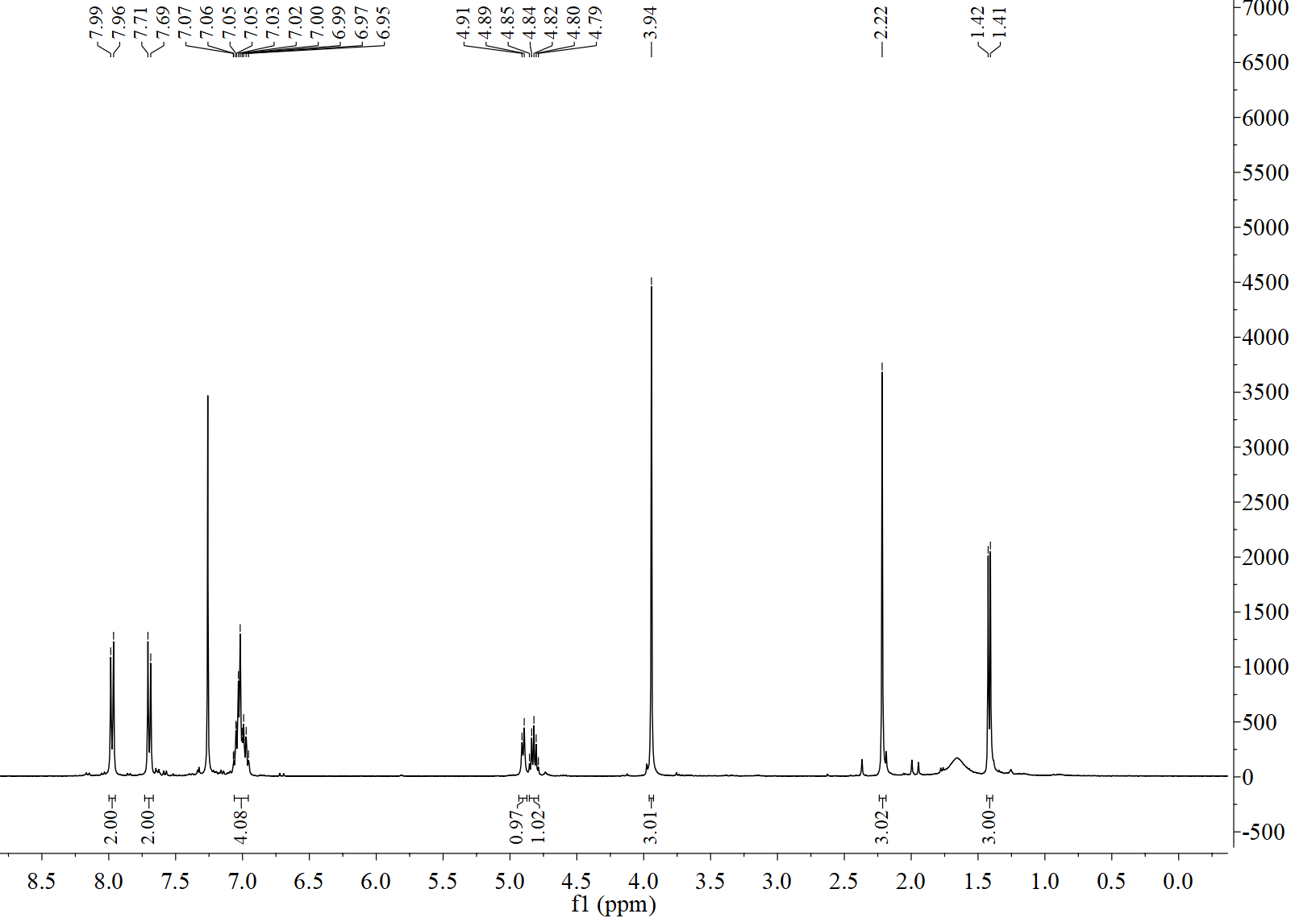


**Figure S71:** ^1^H-NMR (400 MHz, CDCl_3_) of methyl 4-(*N*-(1-(*o*-tolyl)ethyl)sulfamoyl)benzoate (**4i**).


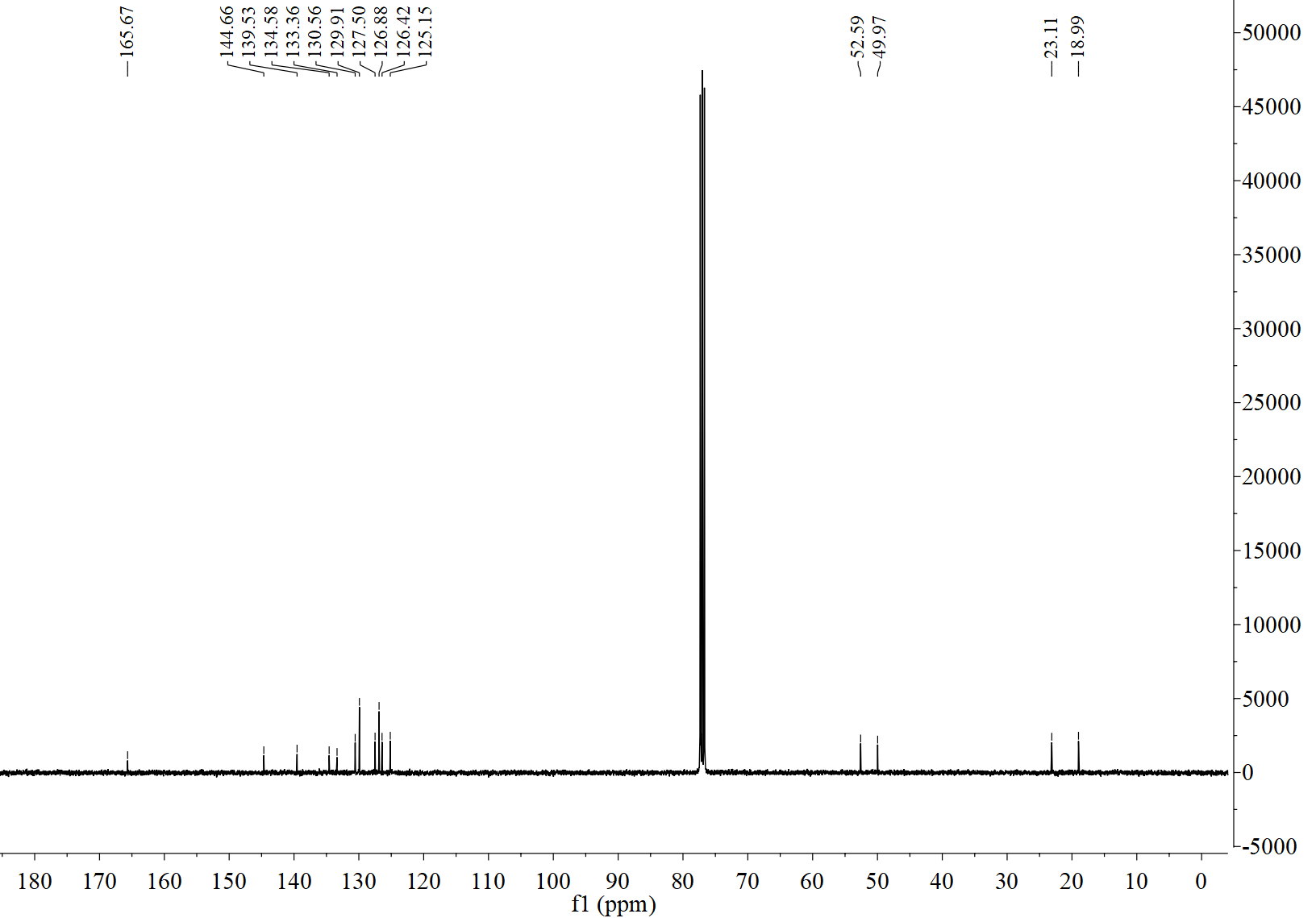


**Figure S72:** ^13^C-NMR (101 MHz, CDCl_3_) of methyl 4-(*N*-(1-(*o*-tolyl)ethyl)sulfamoyl)benzoate (**4i**).


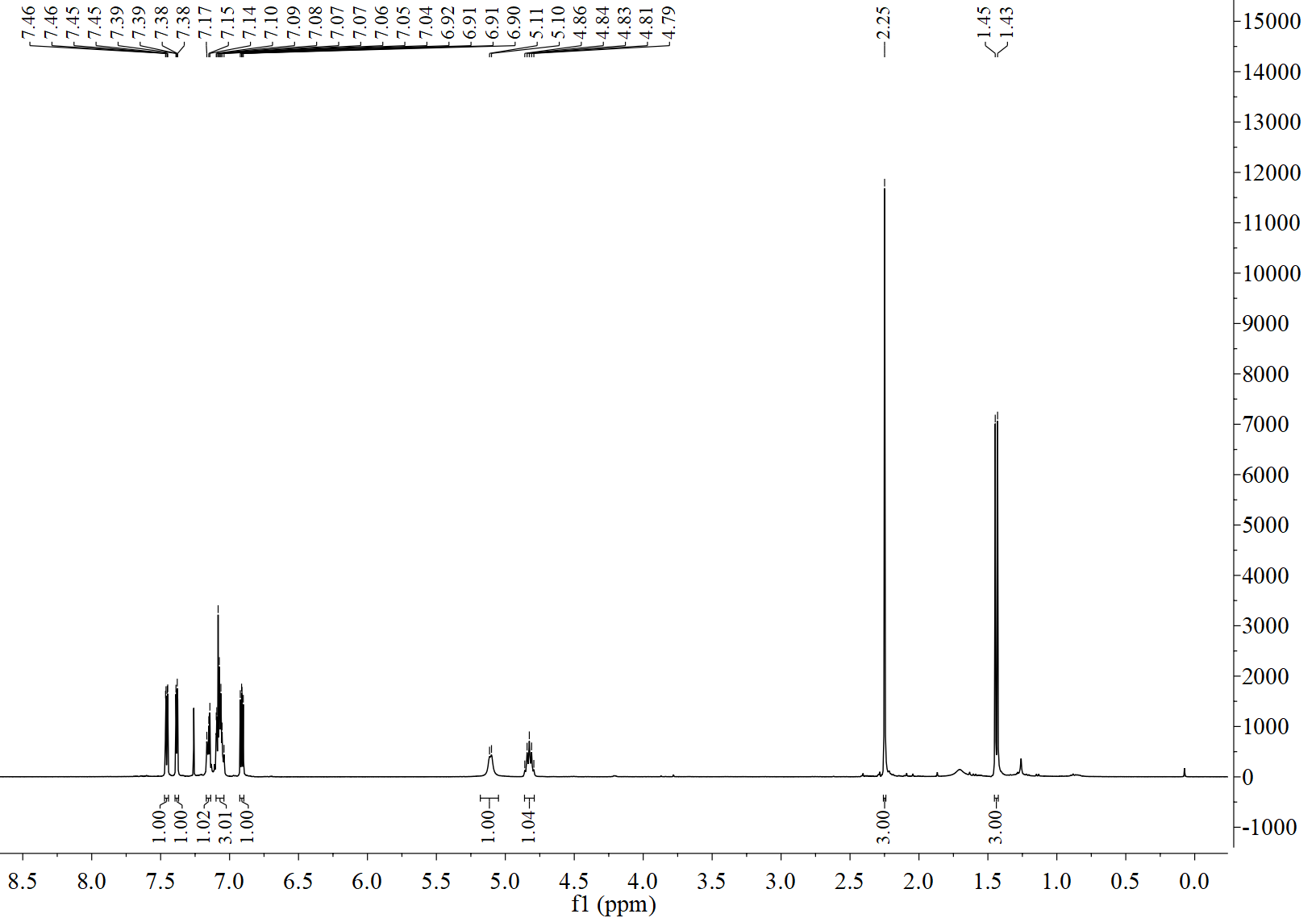


**Figure S73:** ^1^H-NMR (400 MHz, CDCl_3_) of *N*-(1-(*o*-tolyl)ethyl)thiophene-2-sulfonamide (**4j**).


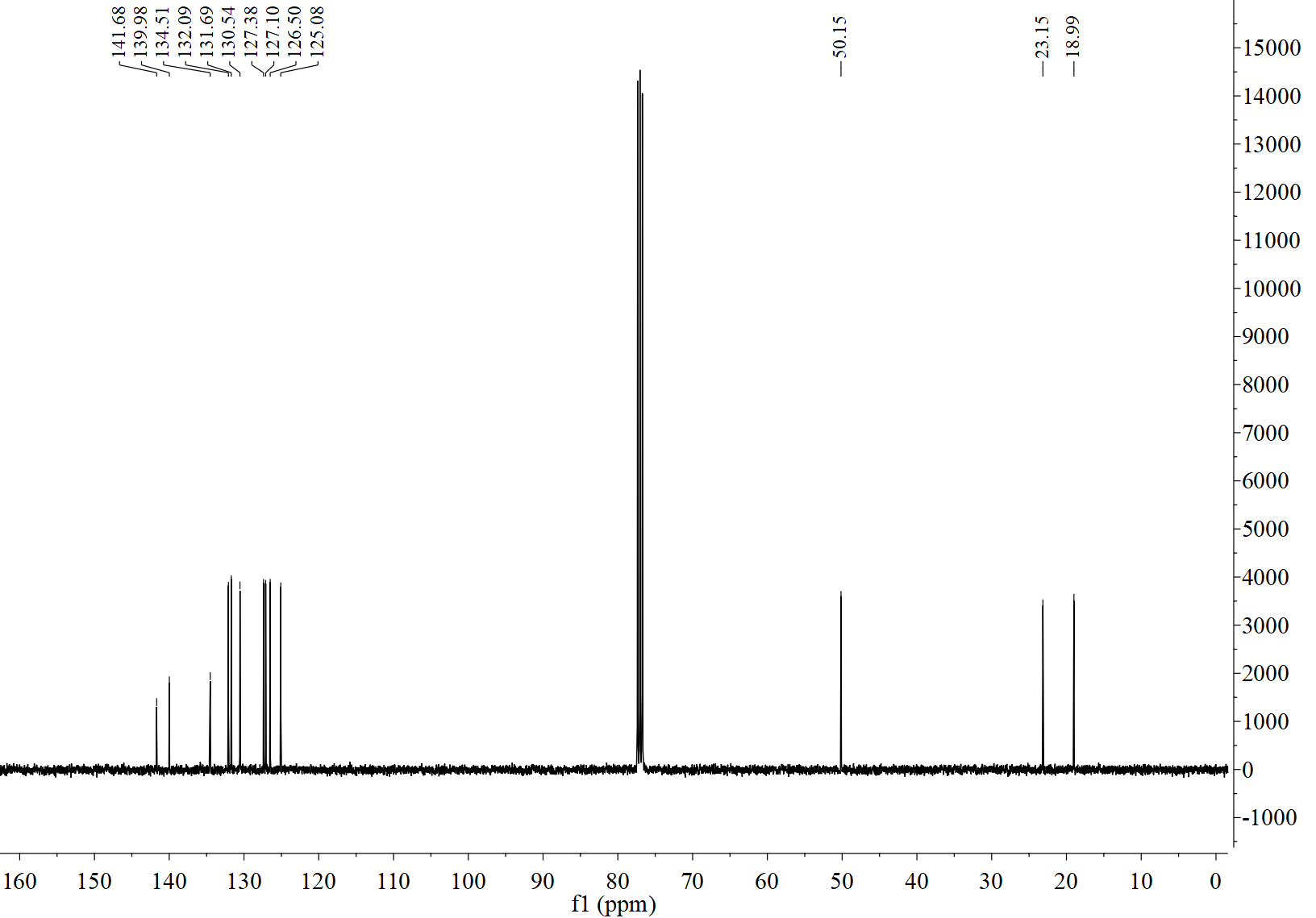


**Figure S74:** ^13^C-NMR (101 MHz, CDCl_3_) of *N*-(1-(*o*-tolyl)ethyl)thiophene-2-sulfonamide (**4j**).


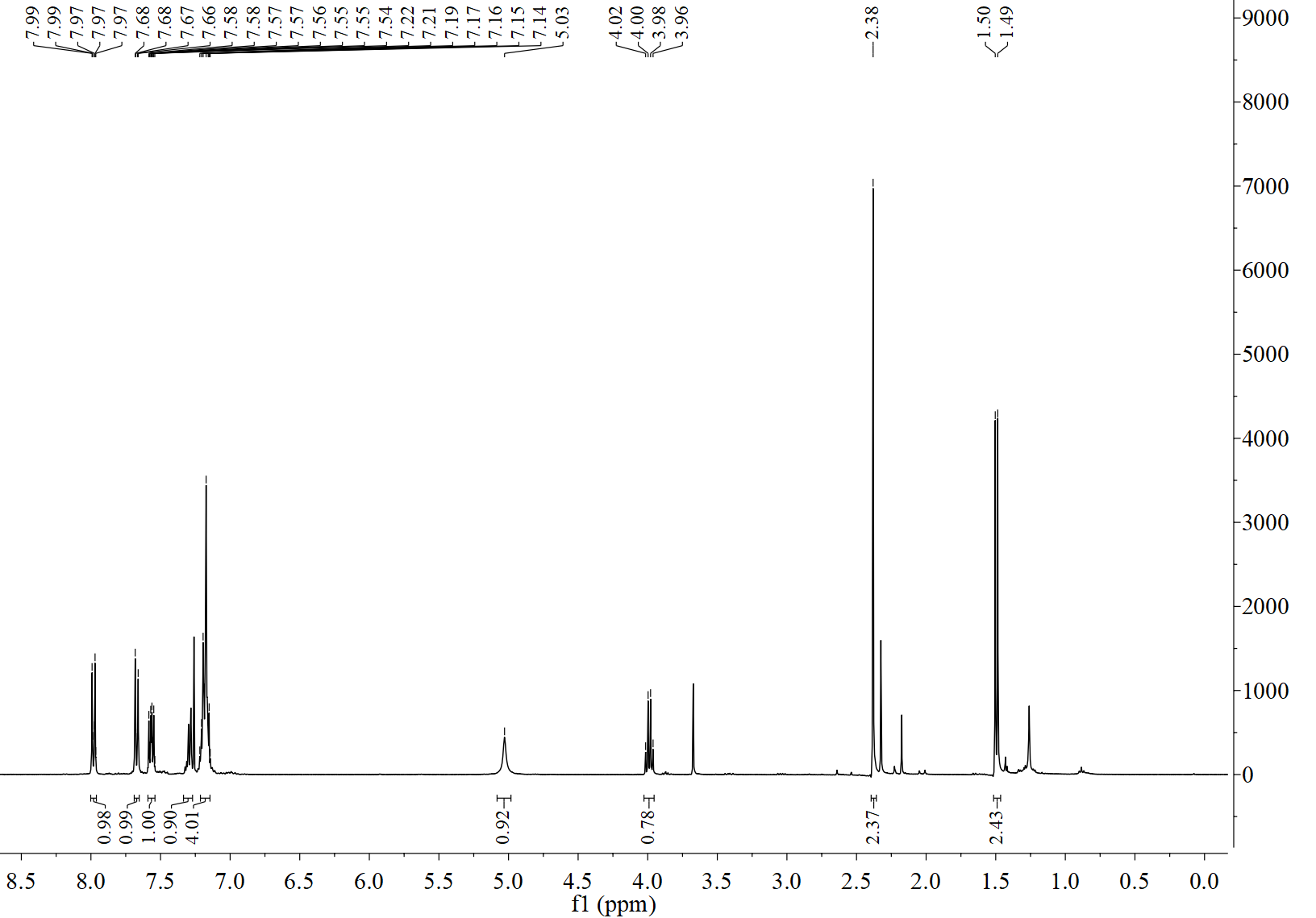


**Figure S75:** ^1^H-NMR (400 MHz, CDCl_3_) of 4’-fluoro-*N*-(1-(*o*-tolyl)ethyl-[1,1’-biphenyl]-4-sulfonamide (**4k**).


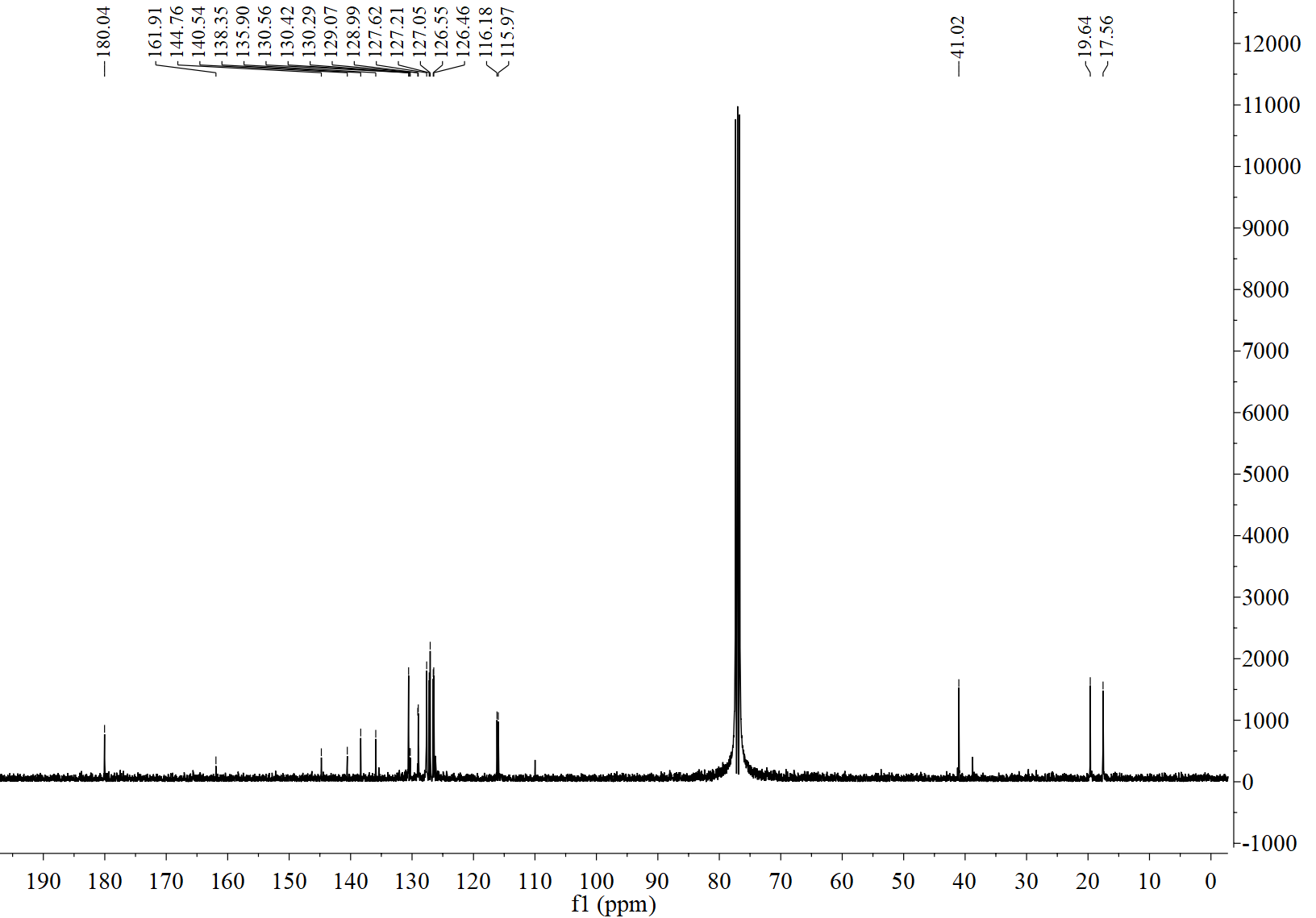


**Figure S76:** ^13^C-NMR (101 MHz, CDCl_3_) of 4’-fluoro-*N*-(1-(*o*-tolyl)ethyl-[1,1’-biphenyl]-4-sulfonamide (**4k**).


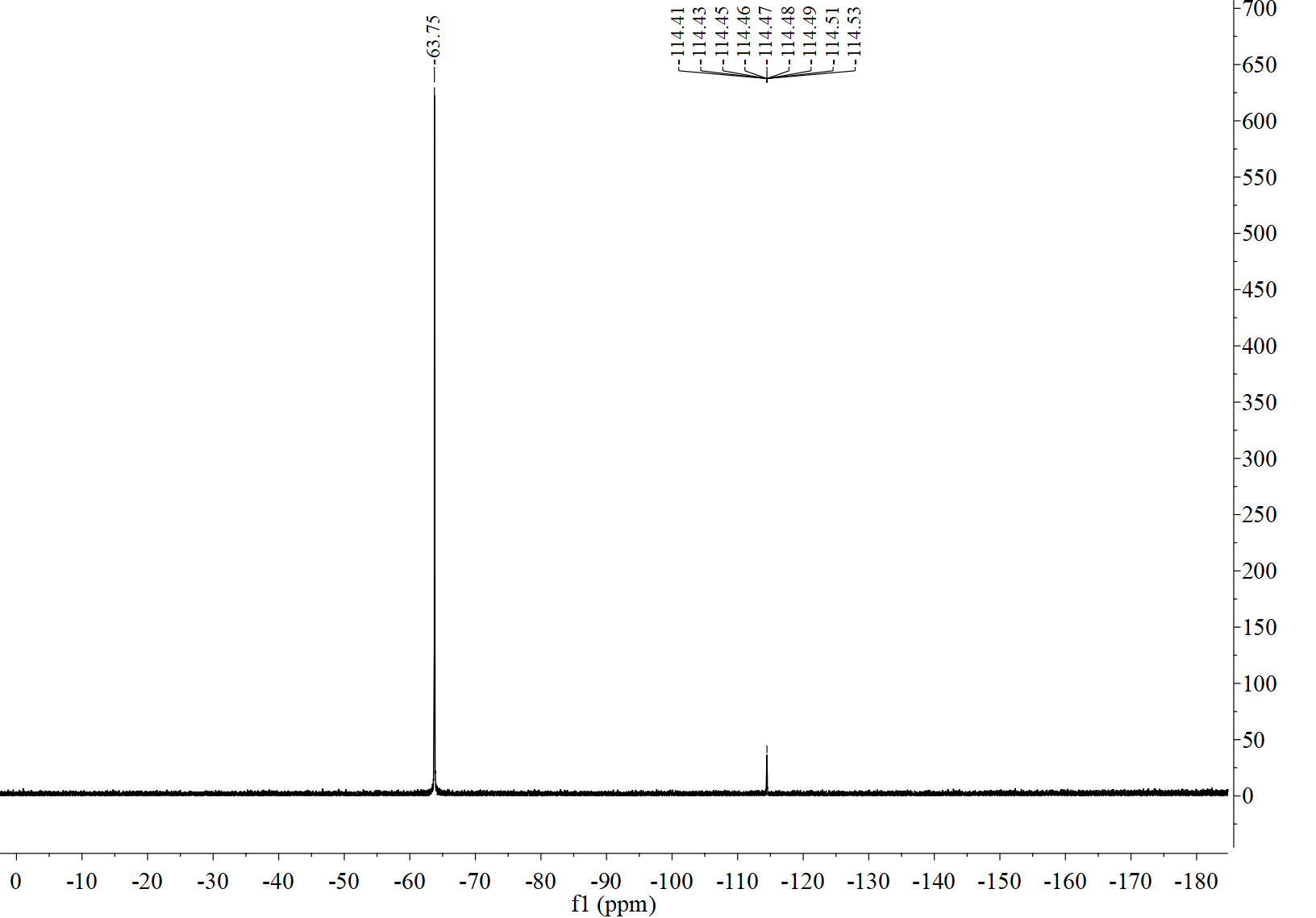


PhCF_3_

**Figure S77:** ^19^F-NMR (376 MHz, CDCl_3_) of 4’-fluoro-*N*-(1-(*o*-tolyl)ethyl-[1,1’-biphenyl]-4-sulfonamide (**4k**) with PhCF_3_ as internal standard.


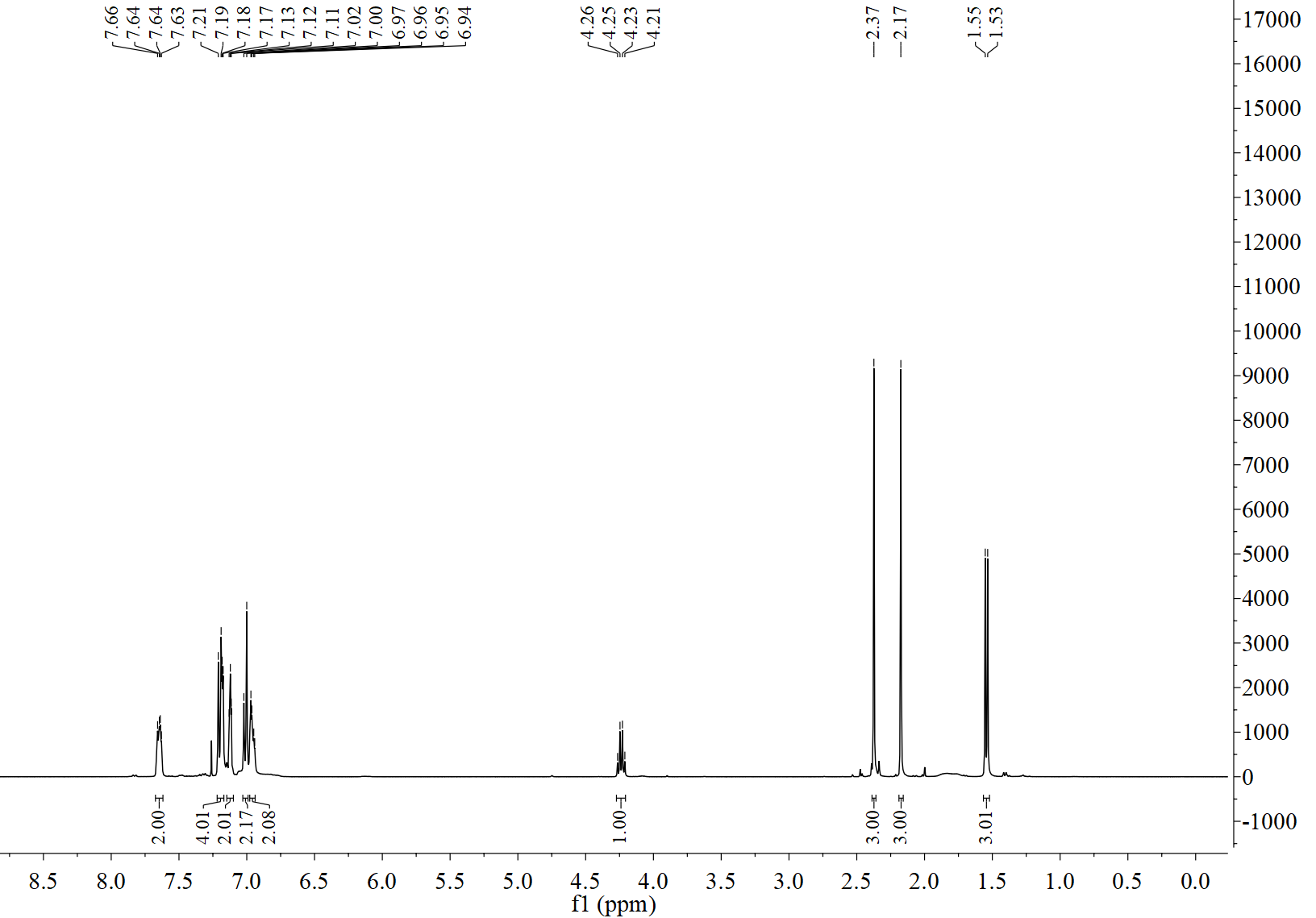


**Figure S78:** ^1^H-NMR (400 MHz, CDCl_3_) of 4-methyl-*N*-phenyl-*N*-(1-(*o*-tolyl)ethyl)benzenesulfonamide (**4l**).


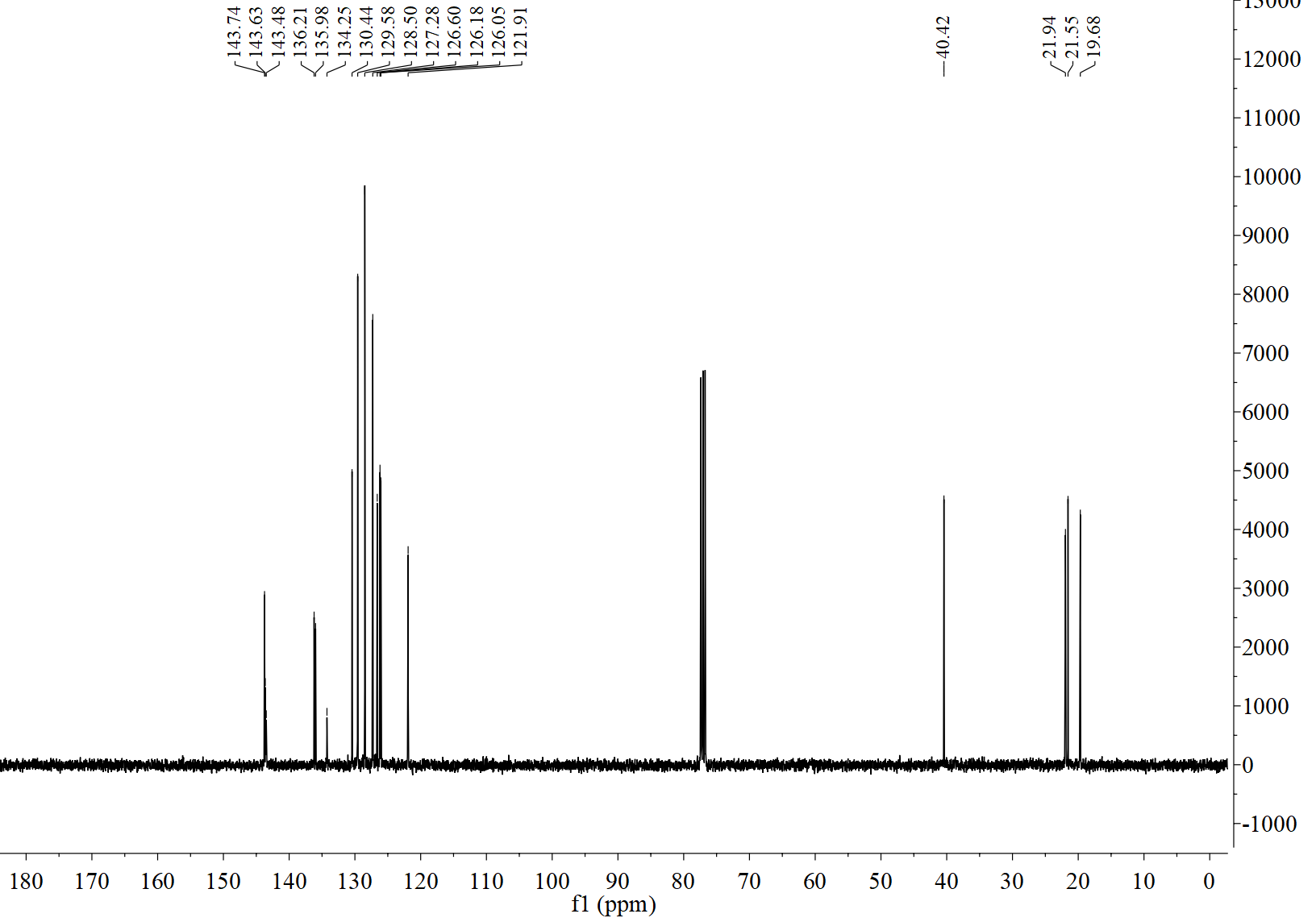


**Figure S79:** ^13^C-NMR (101 MHz, CDCl_3_) of 4-methyl-*N*-phenyl-*N*-(1-(*o*-tolyl)ethyl)benzenesulfonamide (**4l**).


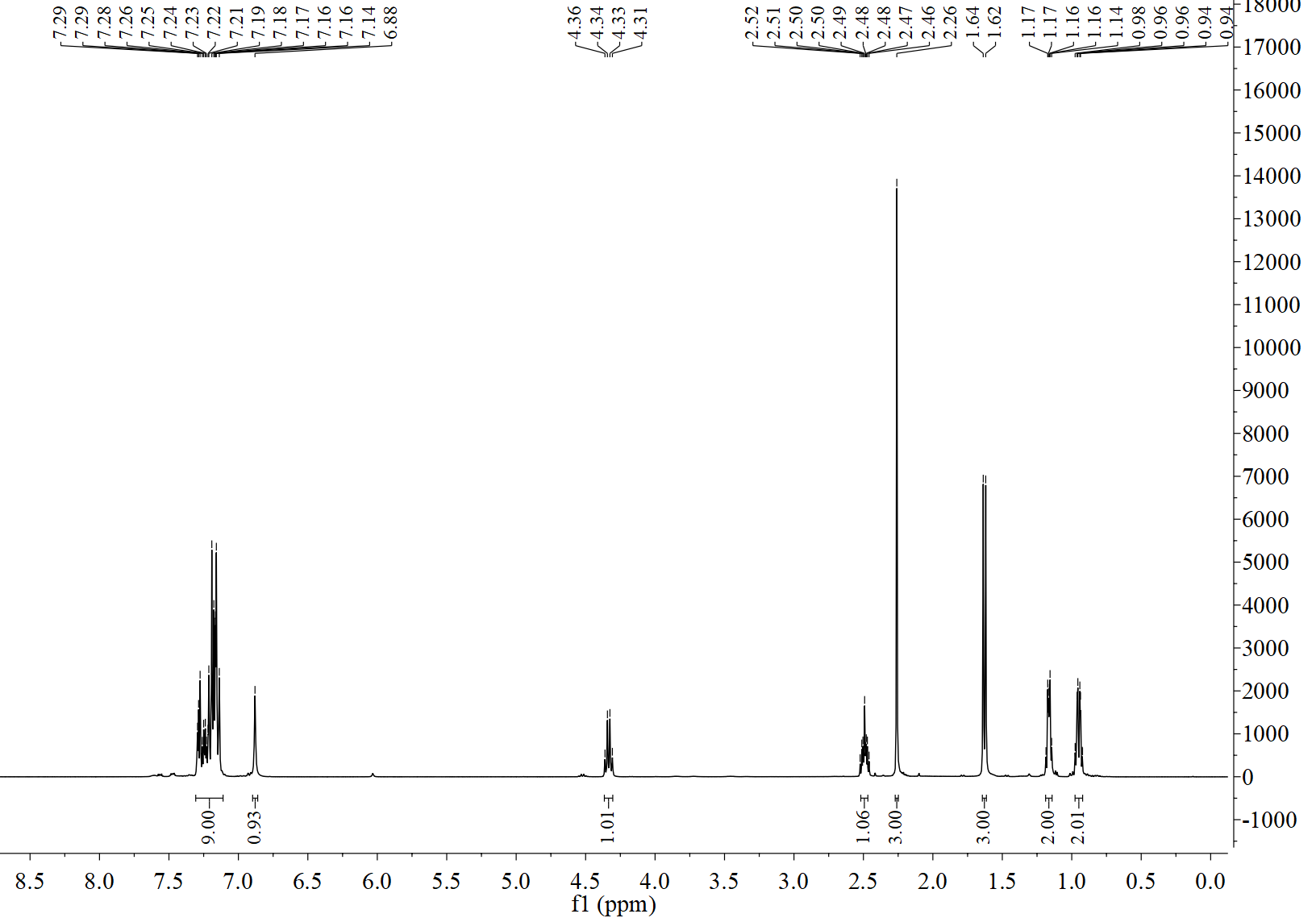


**Figure S80:** ^1^H-NMR (400 MHz, CDCl_3_) of *N*-phenyl-*N*-(1-(*o*-tolyl)ethyl)cyclopropanesulfonamide (**4m**).


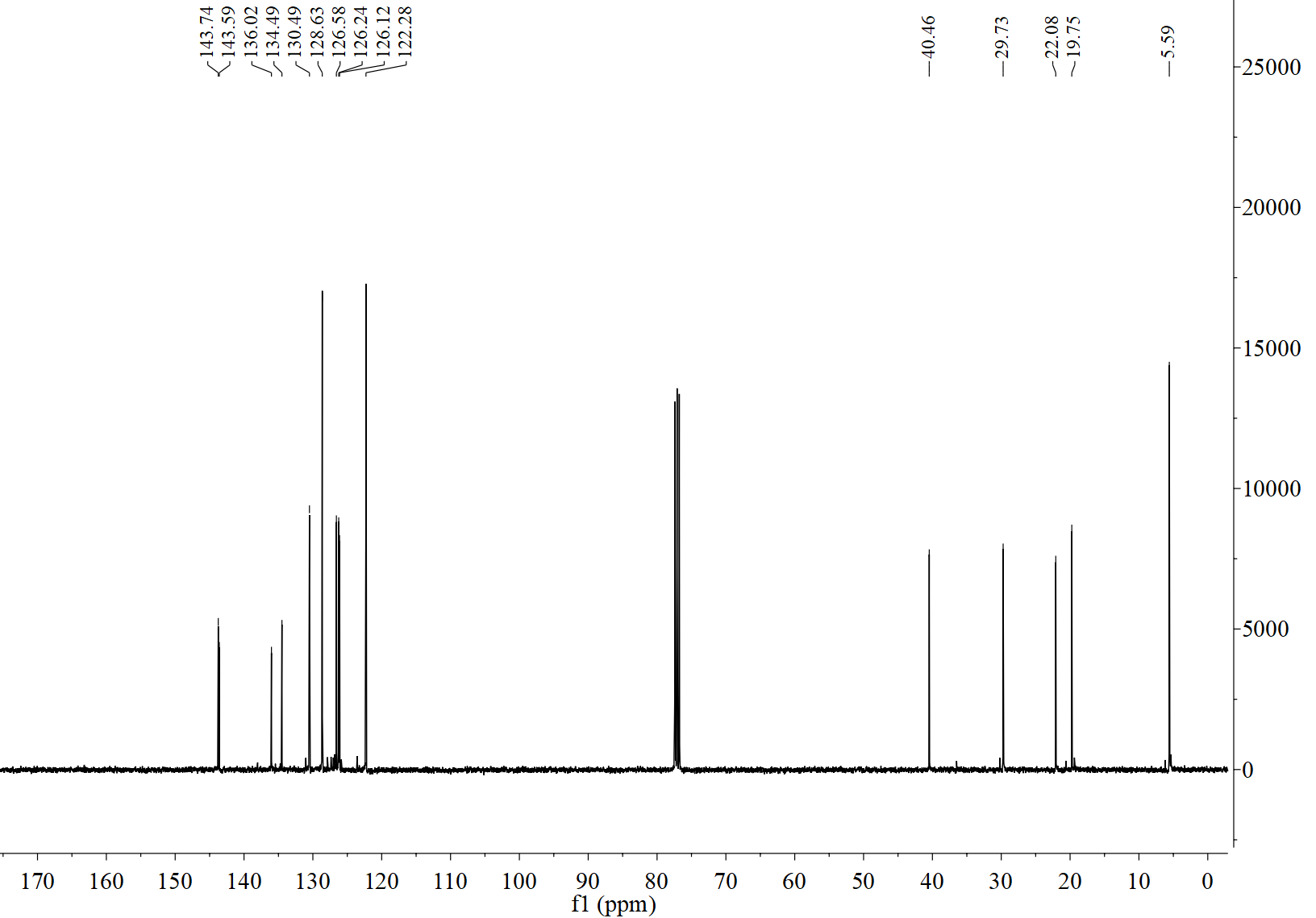


**Figure S81:** ^13^C-NMR (101 MHz, CDCl_3_) of *N*-phenyl-*N*-(1-(*o*-tolyl)ethyl)cyclopropanesulfonamide (**4m**).


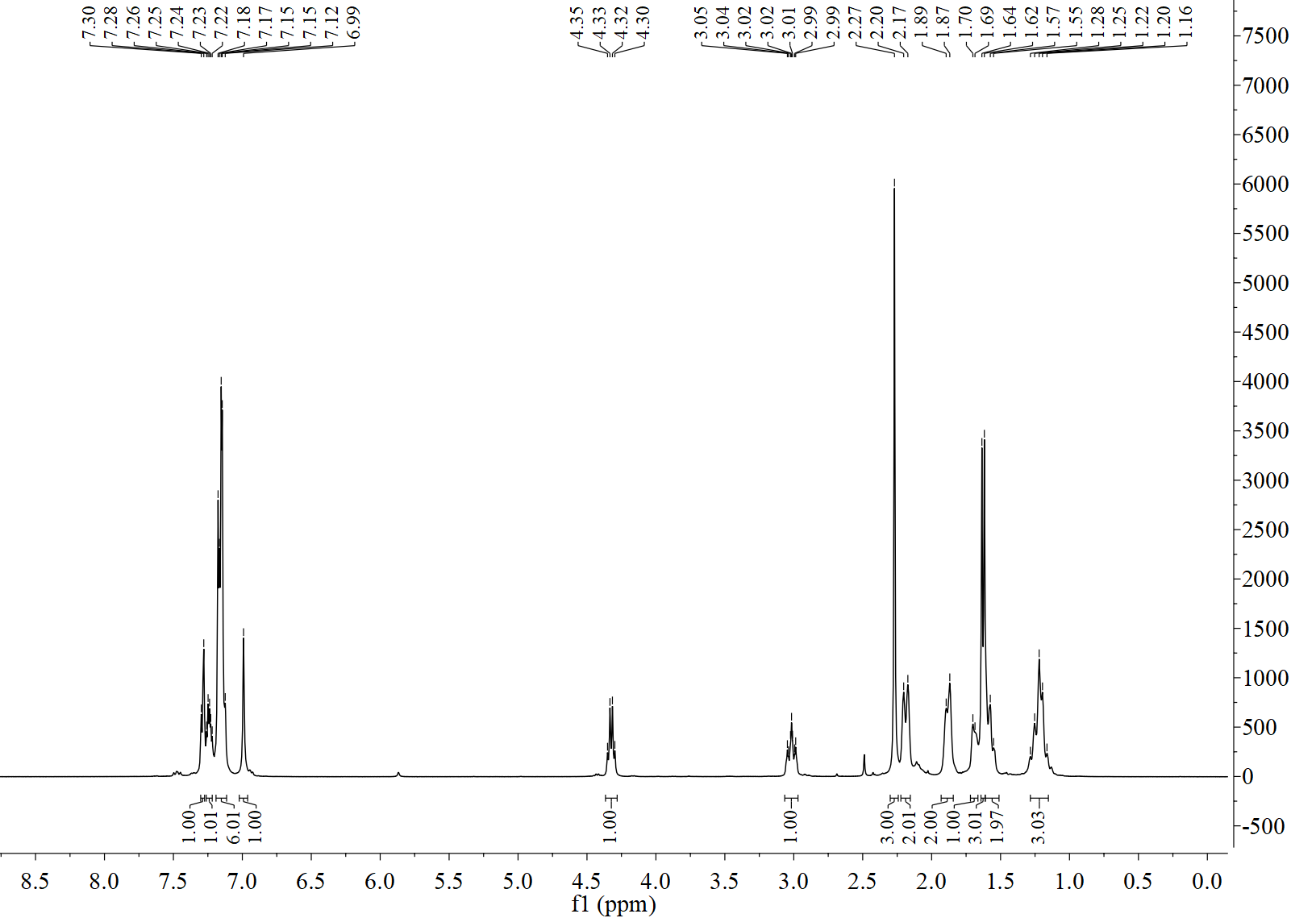


**Figure S82:** ^1^H-NMR (400 MHz, CDCl_3_) of *N*-phenyl-*N*-(1-(*o*-tolyl)ethyl)cyclohexanesulfonamide (**4n**).


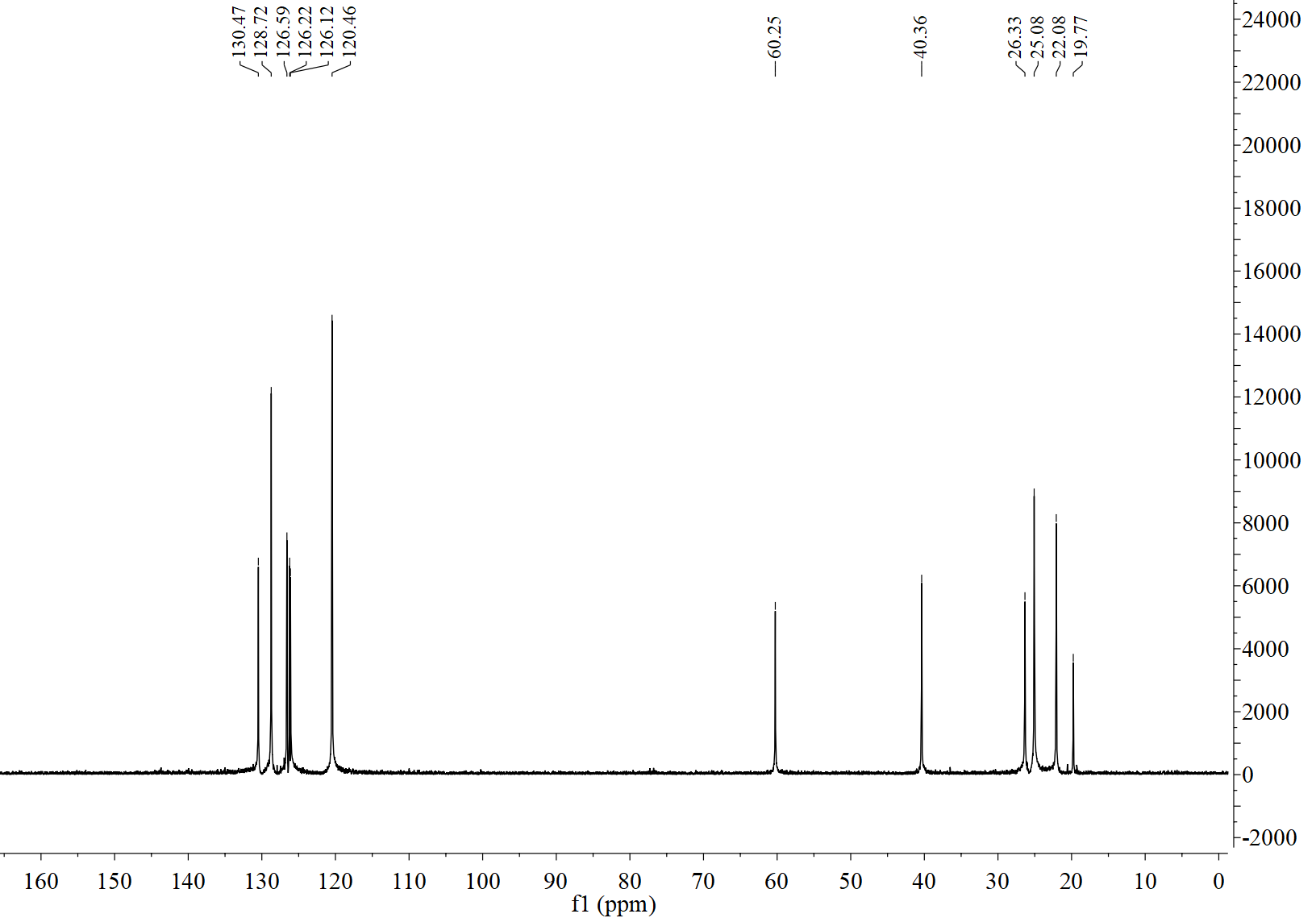


**Figure S83:** ^13^C-NMR (101 MHz, CDCl_3_) of *N*-phenyl-*N*-(1-(*o*-tolyl)ethyl)cyclohexanesulfonamide (**4n**).


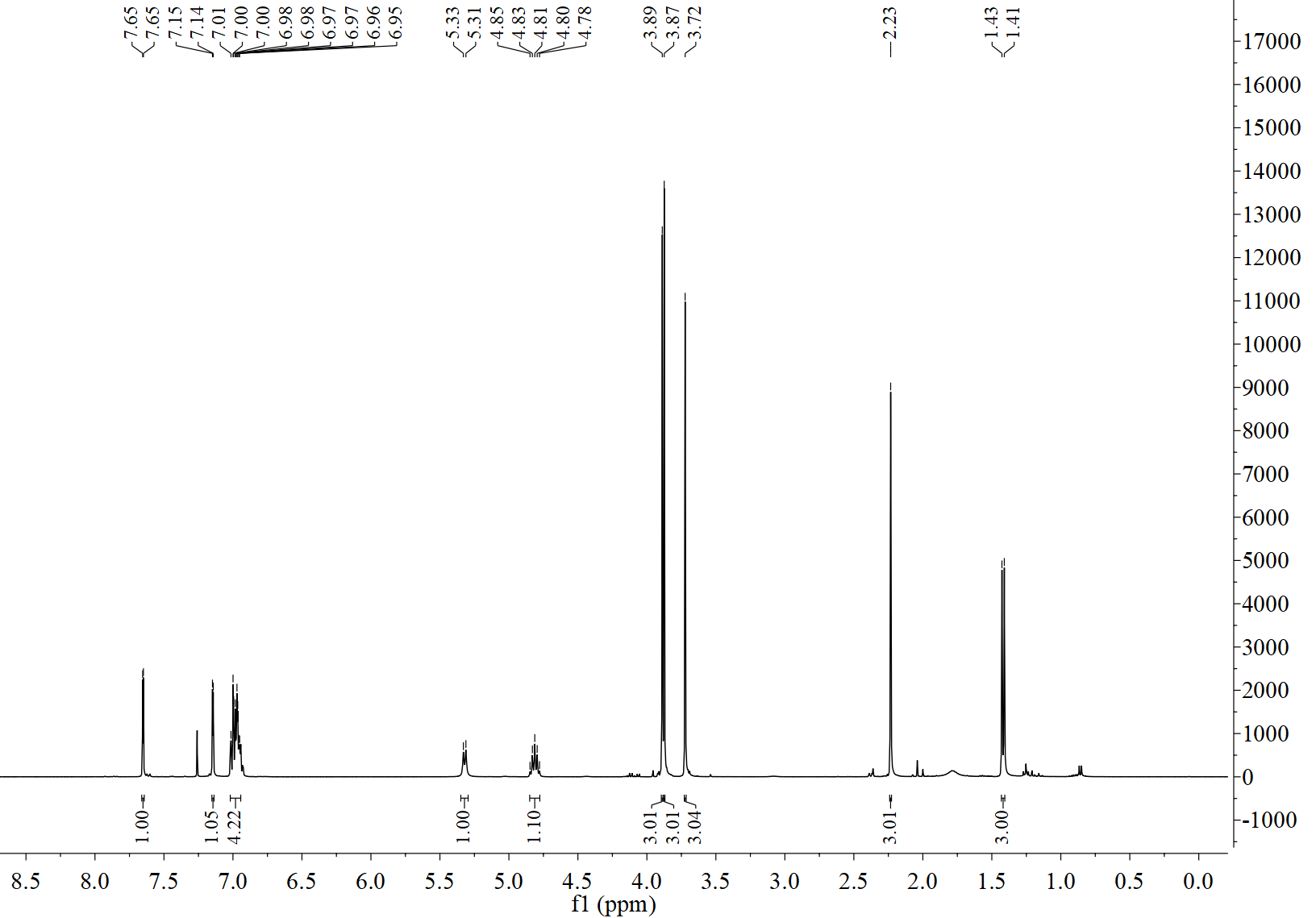


**Figure S84:** ^1^H-NMR (400 MHz, CDCl_3_) of methyl 2,3-dimethoxy-5-(*N*-(1-(*o*-tolyl)ethyl)sulfamoylbenzoate (**4o**).


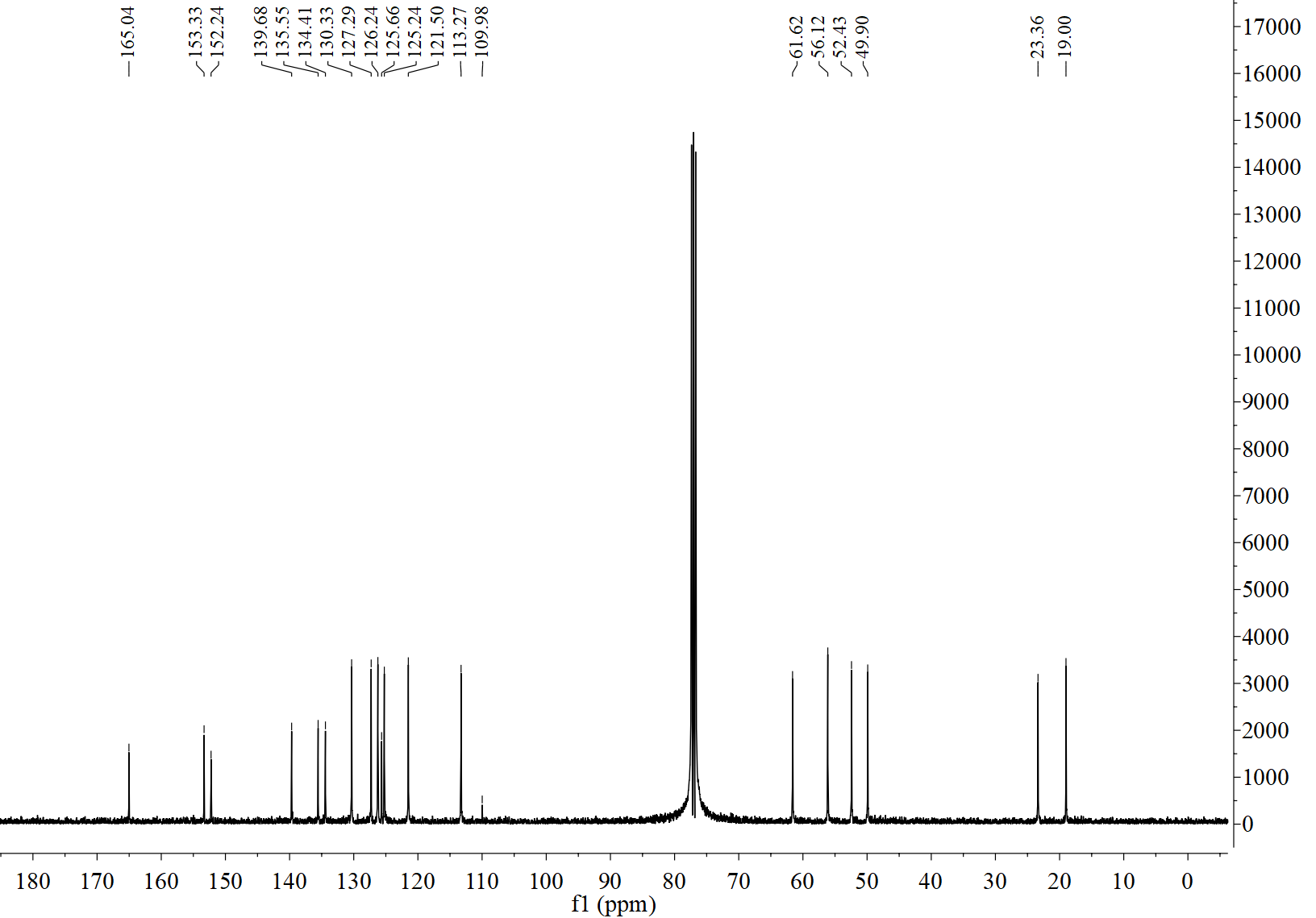


**Figure S85:** ^13^C-NMR (101 MHz, CDCl_3_) of methyl 2,3-dimethoxy-5-(*N*-(1-(*o*-tolyl)ethyl)sulfamoylbenzoate (**4o**).


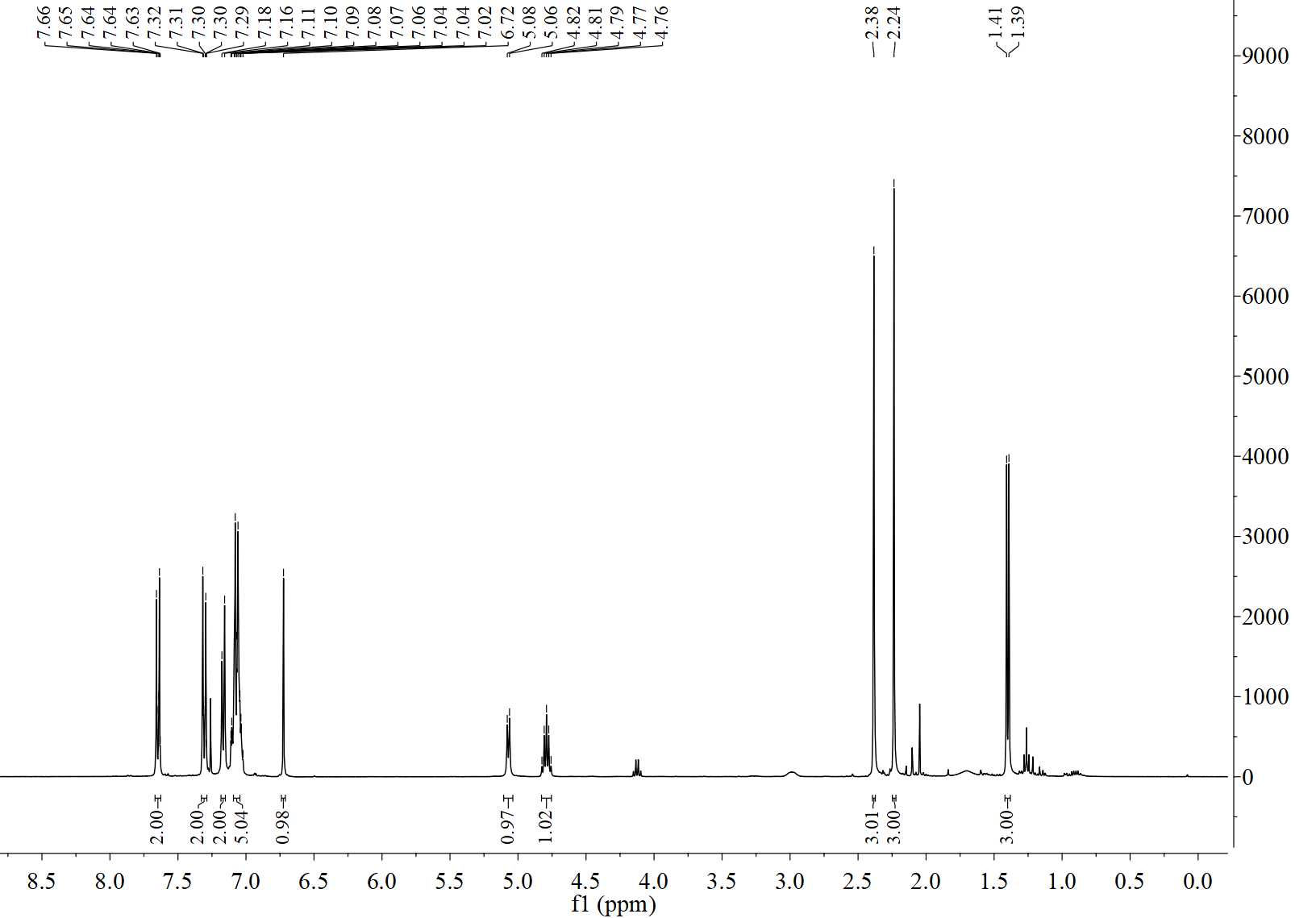


**Figure S86:** ^1^H-NMR (400 MHz, CDCl_3_) of 4-(5-(*p*-tolyl)-3-(trifluoromethyl)-1*H*-pyrazol-1-yl)-*N*-(1-(*o*-tolyl)ethyl)benzenesulfonamide (**4p**).


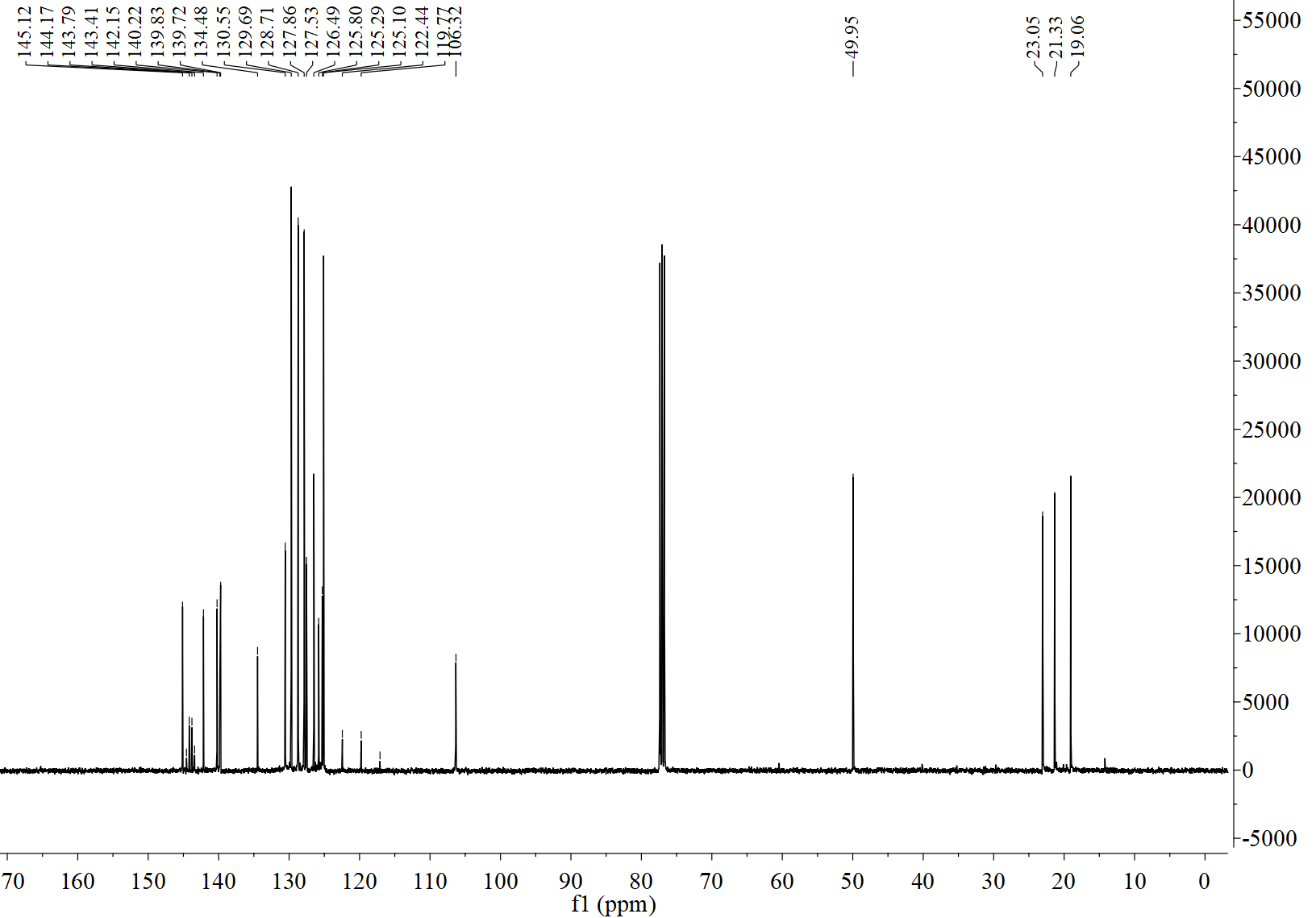


**Figure S87:** ^13^C-NMR (101 MHz, CDCl_3_) of 4-(5-(*p*-tolyl)-3-(trifluoromethyl)-1*H*-pyrazol-1-yl)-*N*-(1-(*o*-tolyl)ethyl)benzenesulfonamide (**4p**).


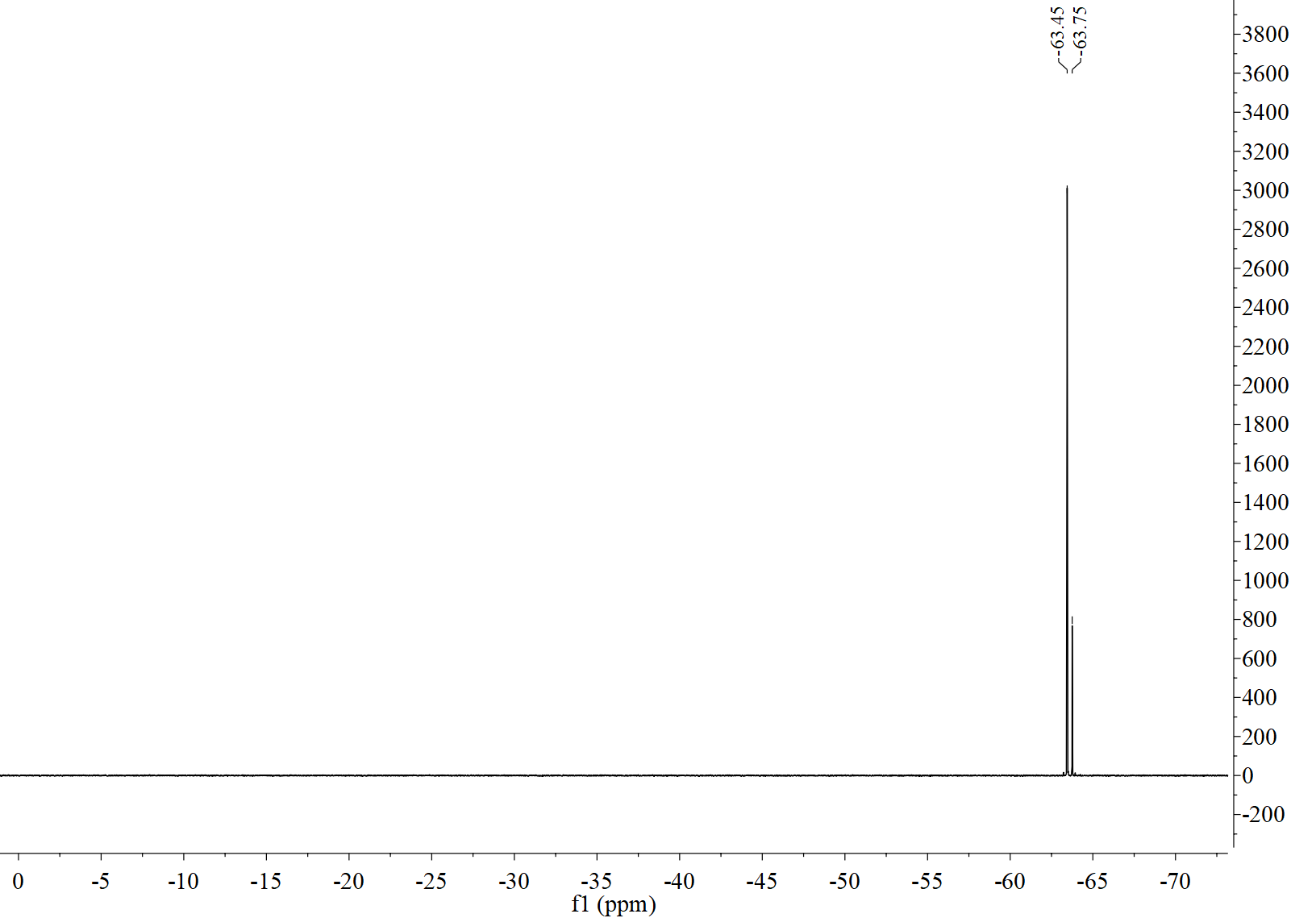


PhCF_3_

**Figure S88:** ^19^F-NMR (376 MHz, CDCl_3_) of 4-(5-(*p*-tolyl)-3-(trifluoromethyl)-1*H*-pyrazol-1-yl)-*N*-(1-(*o*-tolyl)ethyl)benzenesulfonamide (**4p**) with PhCF_3_ as internal standard.

# NMR-Spectra of the synthetized starting materials


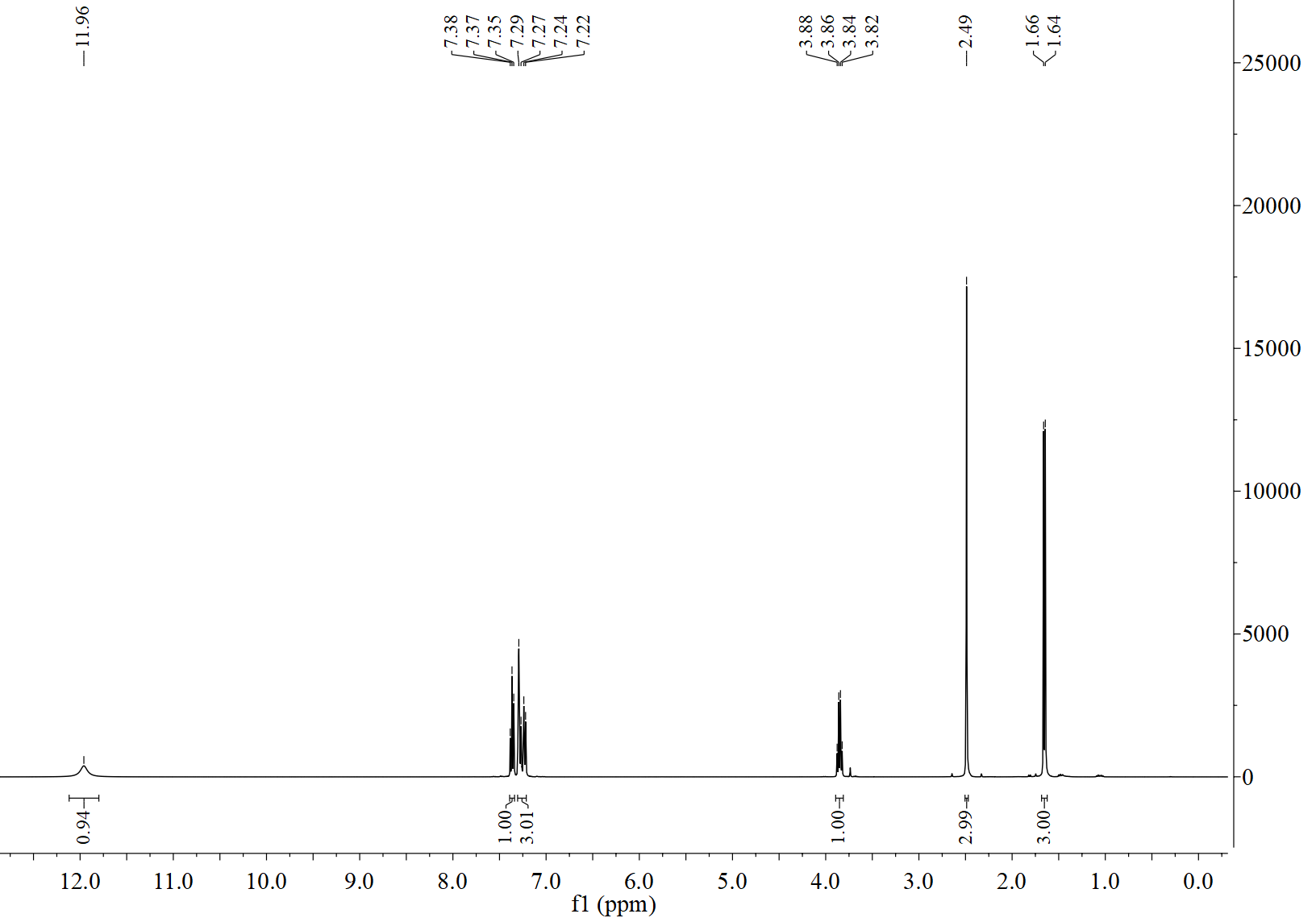


**Figure S89:** ^1^H-NMR (400 MHz, CDCl_3_) of 2-(*m*-tolyl)propanoic acid (**1d**).


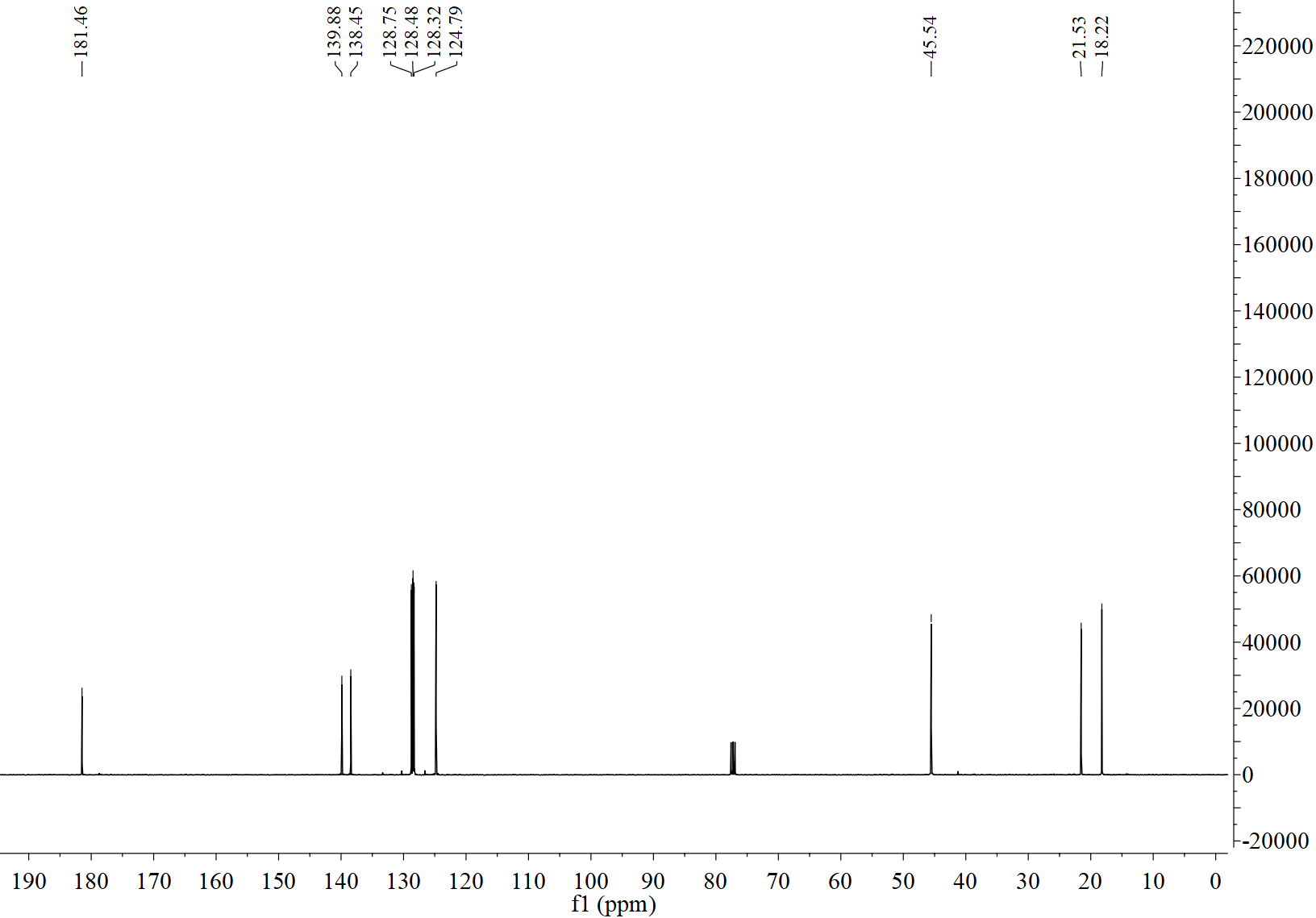


**Figure S90:** ^13^C-NMR (101 MHz, CDCl_3_) of 2-(*m*-tolyl)propanoic acid (**1d**).


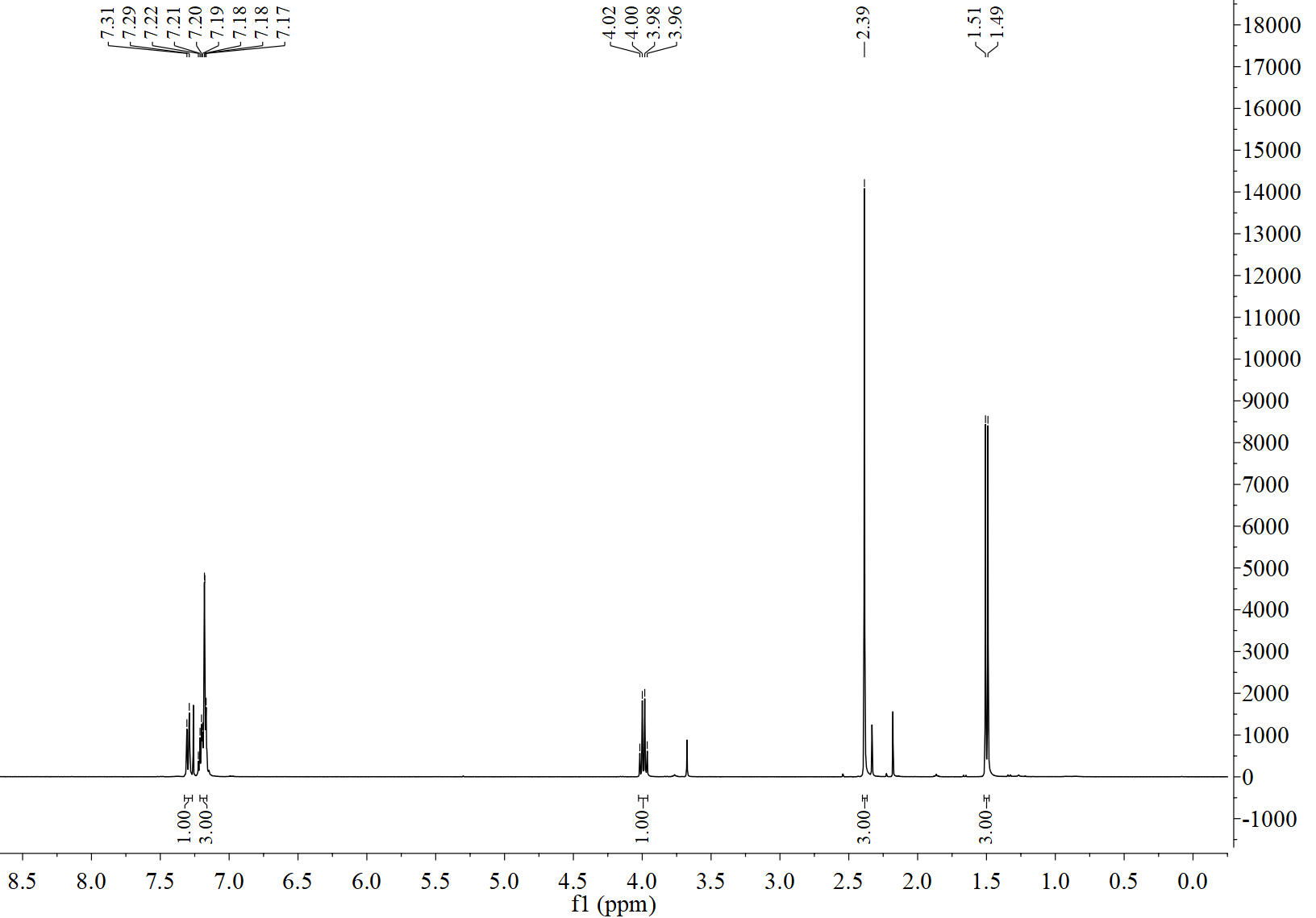


**Figure S91:** ^1^H-NMR (400 MHz, CDCl_3_) of 2-(*o*-tolyl)propanoic acid (**1e**).


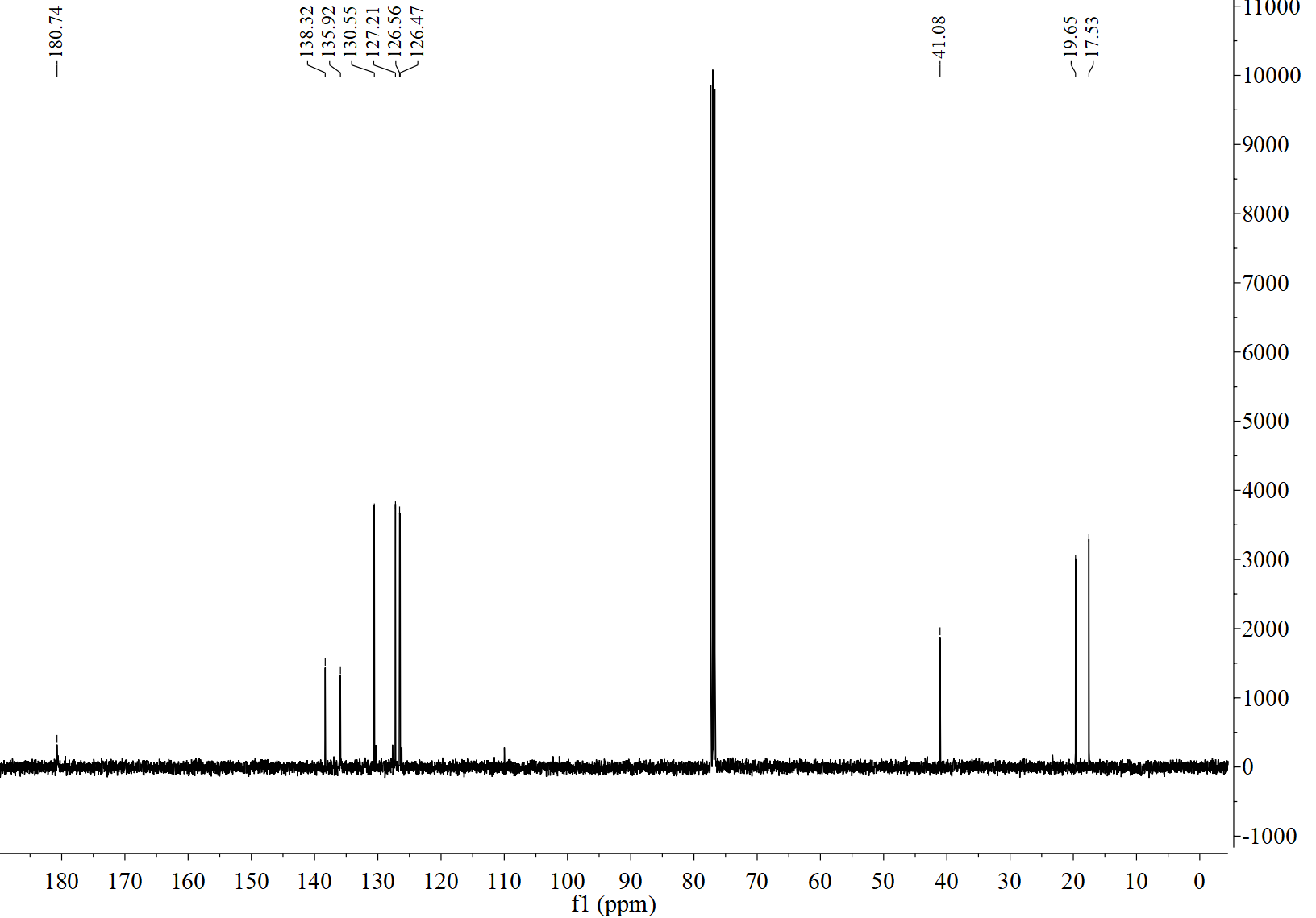


**Figure S92:** ^13^C-NMR (101 MHz, CDCl_3_) of 2-(*o*-tolyl)propanoic acid (**1e**).


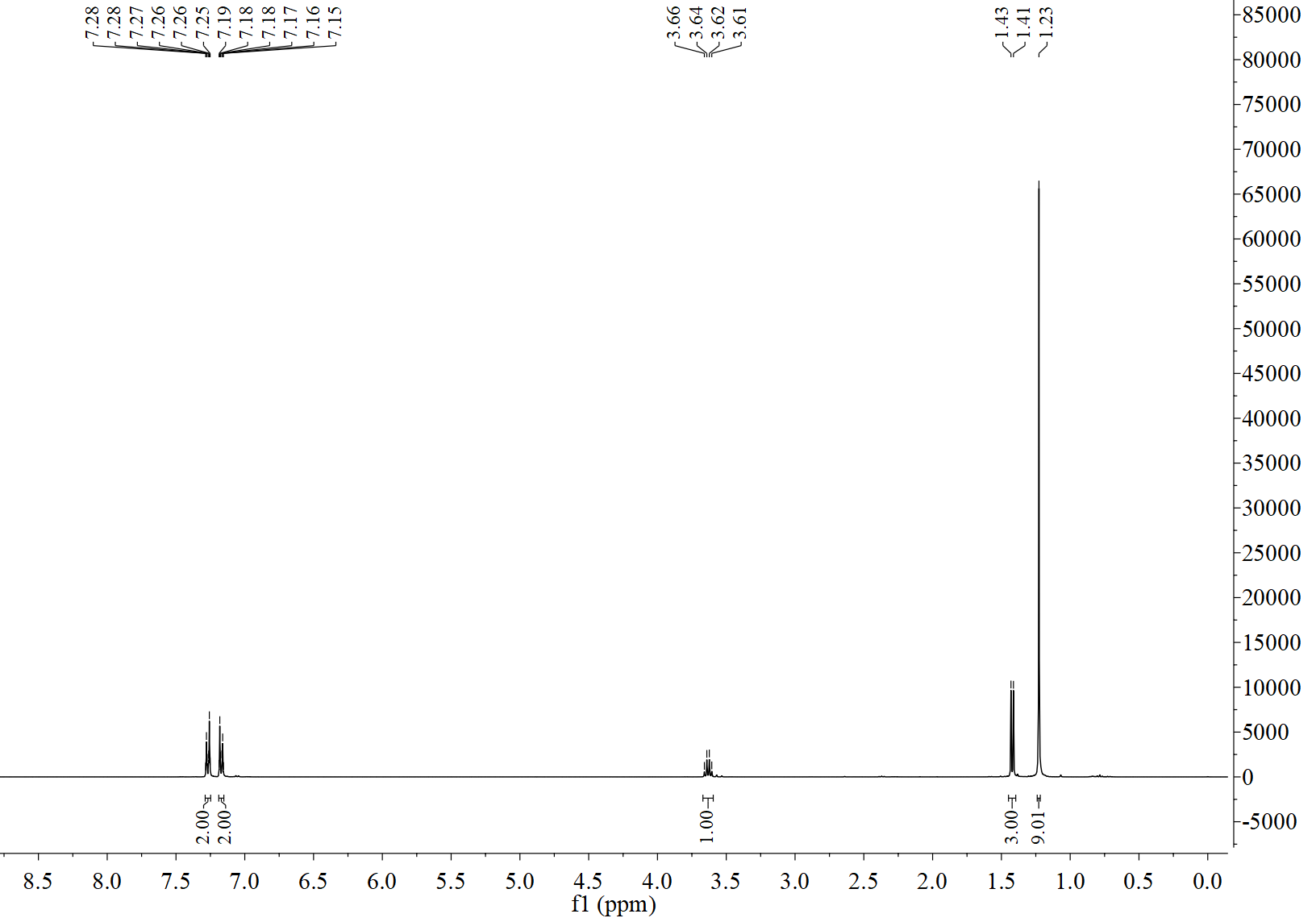


**Figure S93:** ^1^H-NMR (400 MHz, CDCl_3_) of 2-(4-(*tert*-butyl)phenyl)propanoic acid (**1f**).


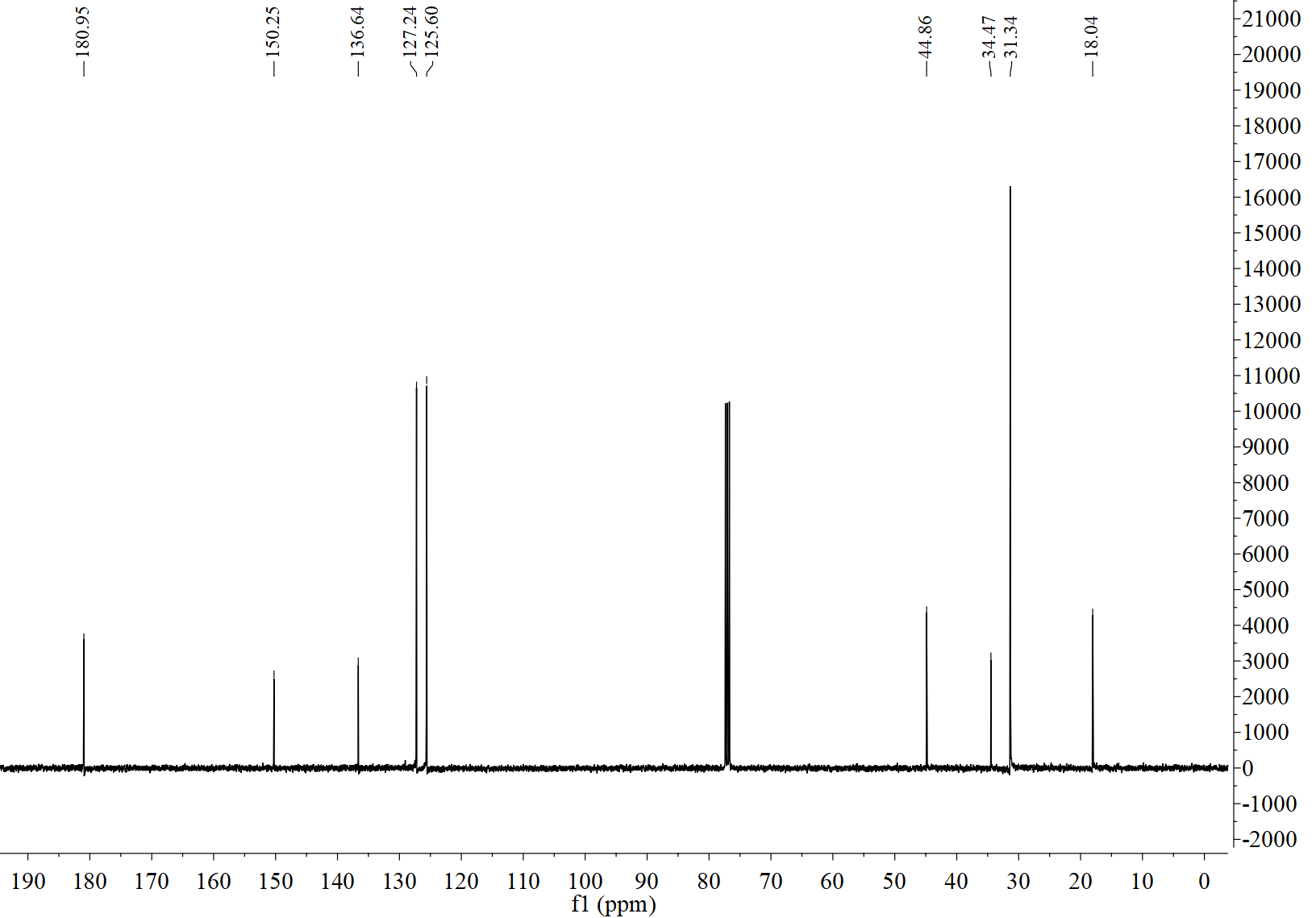


**Figure S94:** ^13^C-NMR (101 MHz, CDCl_3_) of 2-(4-(*tert*-butyl)phenyl)propanoic acid (**1f**).


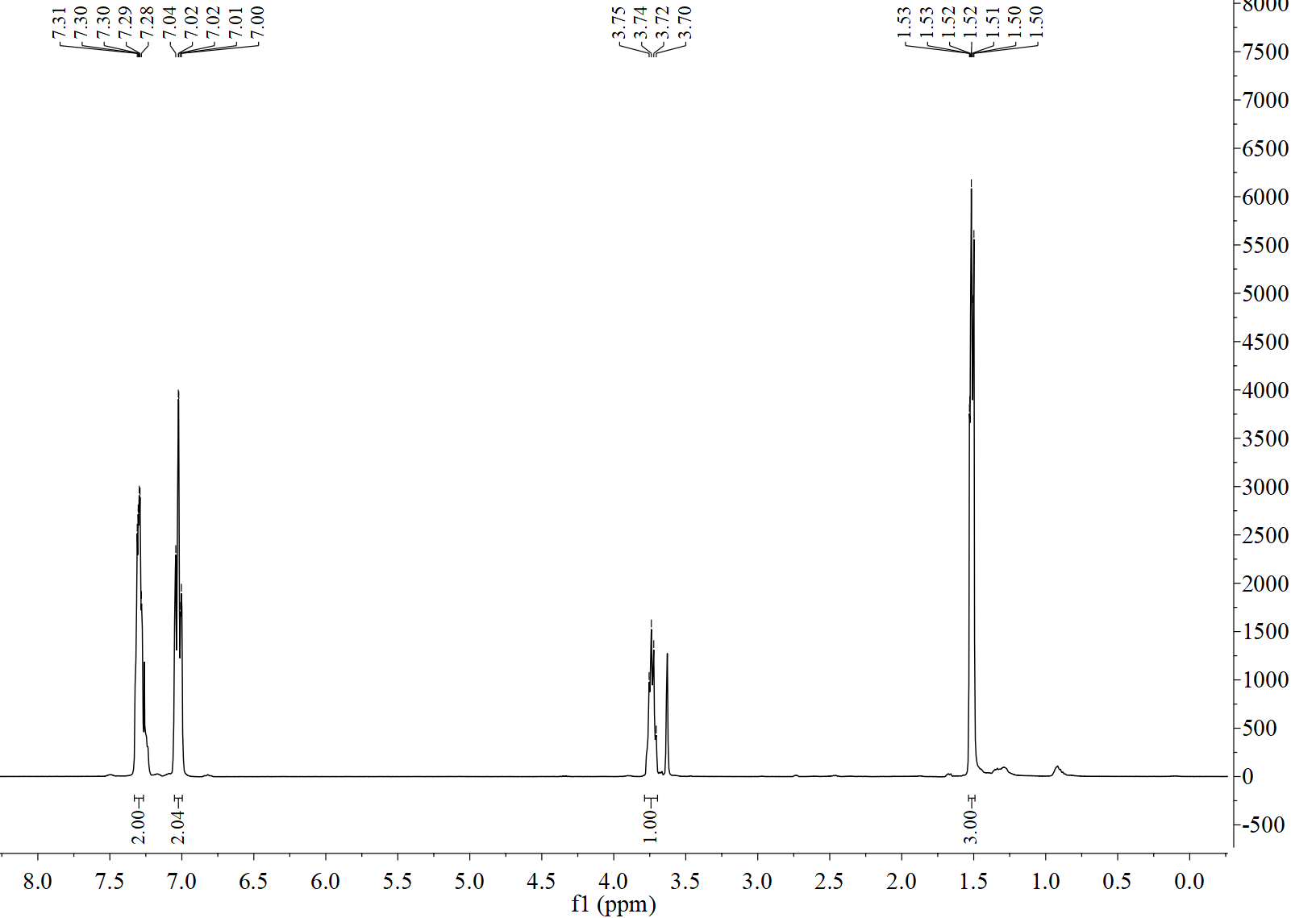


**Figure S95:** ^1^H-NMR (400 MHz, CDCl_3_) of 2-(4-fluorophenyl)propanoic acid (**1g**).


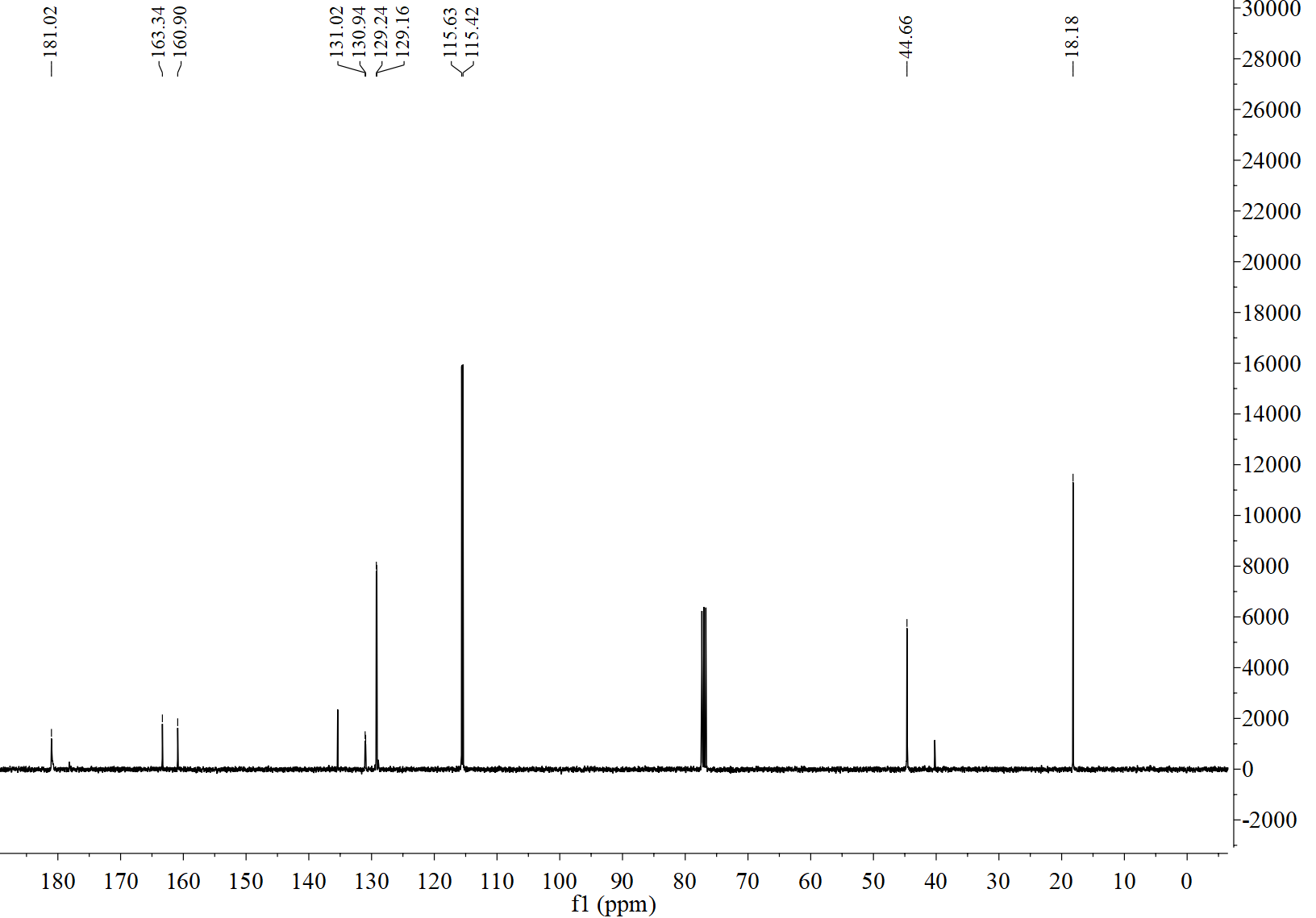


**Figure S96:** ^13^C-NMR (101 MHz, CDCl_3_) of 2-(4-fluorophenyl)propanoic acid (**1g**).


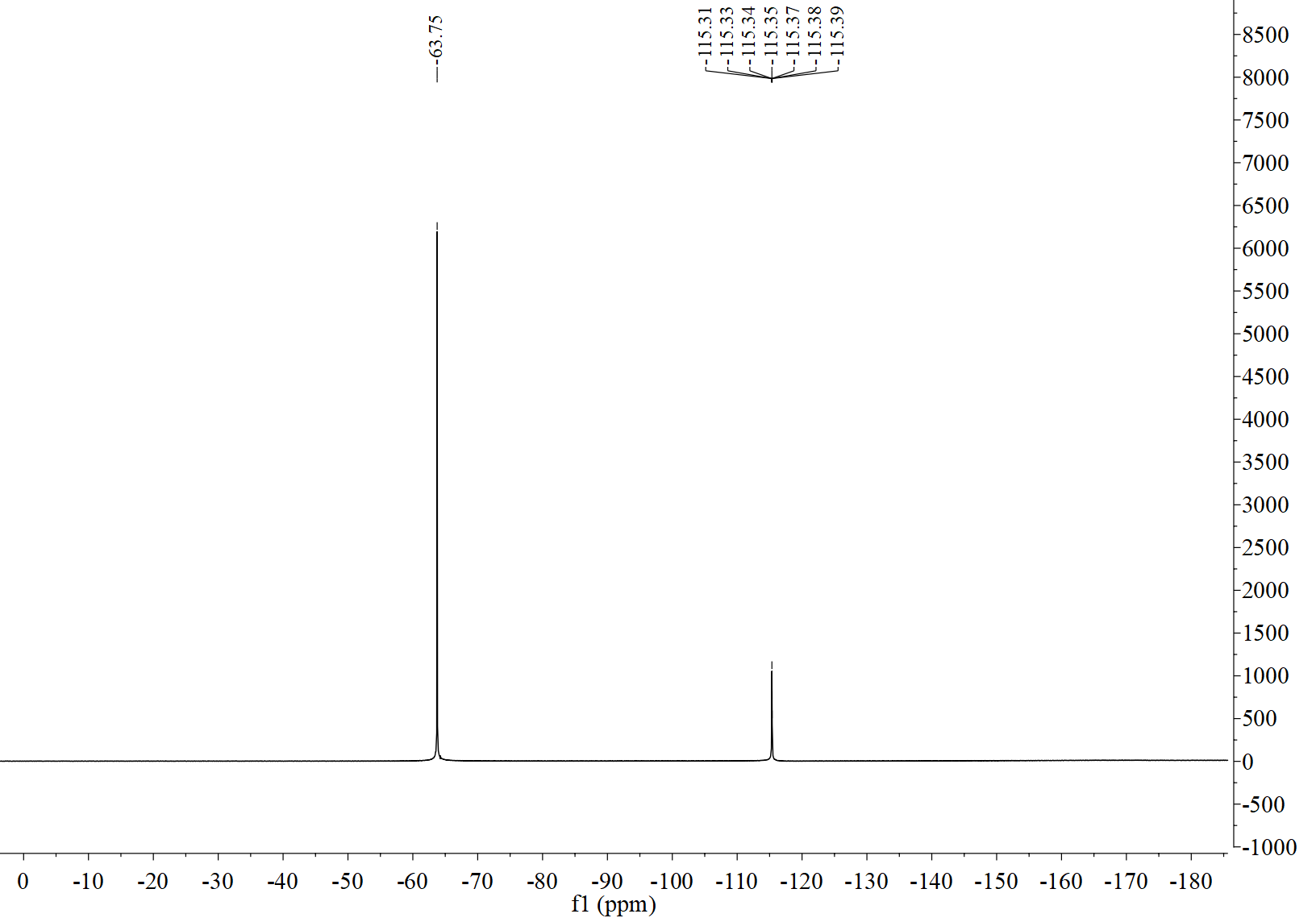


PhCF_3_

**Figure S97:** ^19^F-NMR (376 MHz, CDCl_3_) of 2-(4-fluorophenyl)propanoic acid (**1g**) with PhCF_3_ as internal standard.


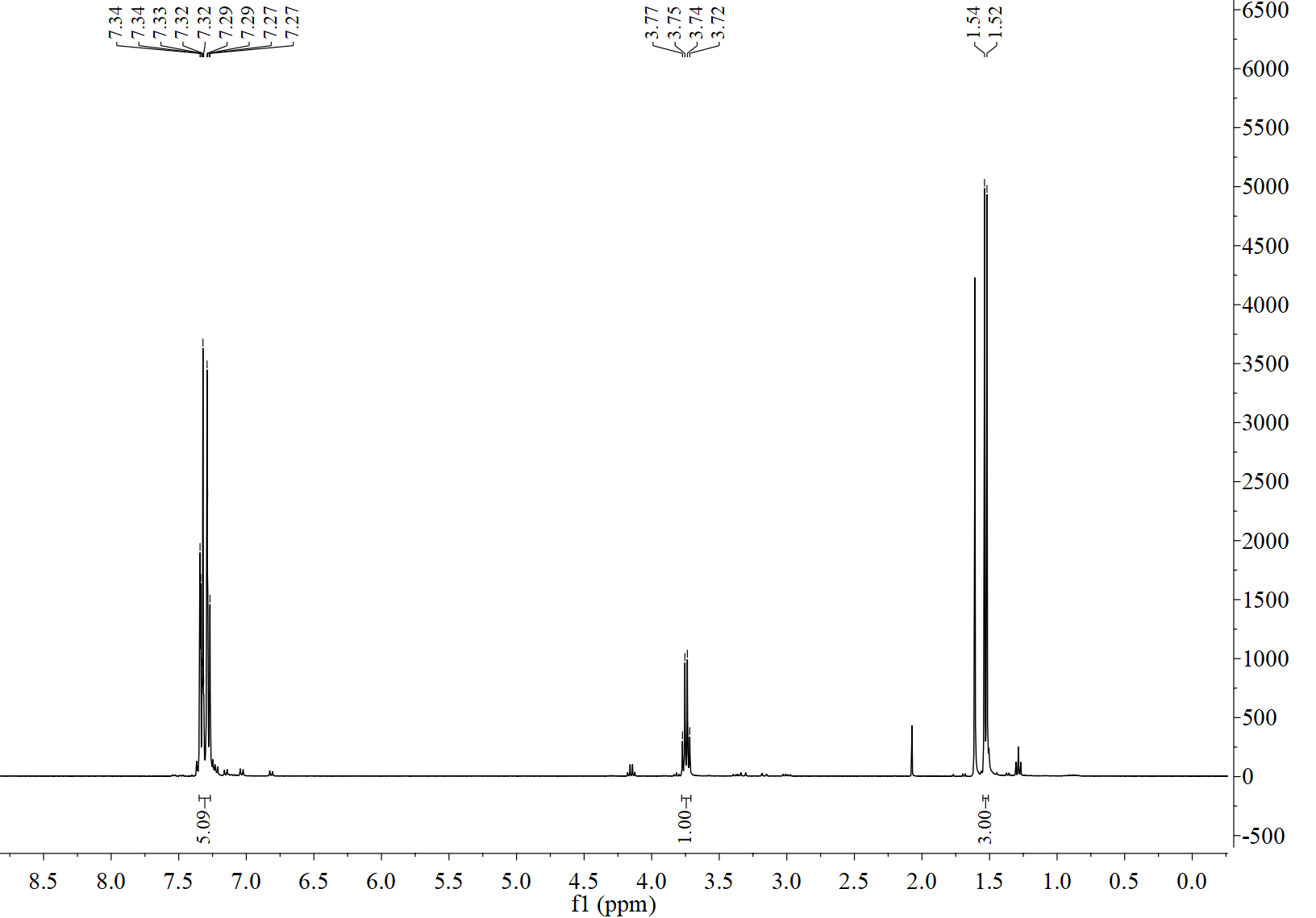


**Figure S98:** ^1^H-NMR (400 MHz, CDCl_3_) of 2-(4-chlorophenyl)propanoic acid (**1h**).


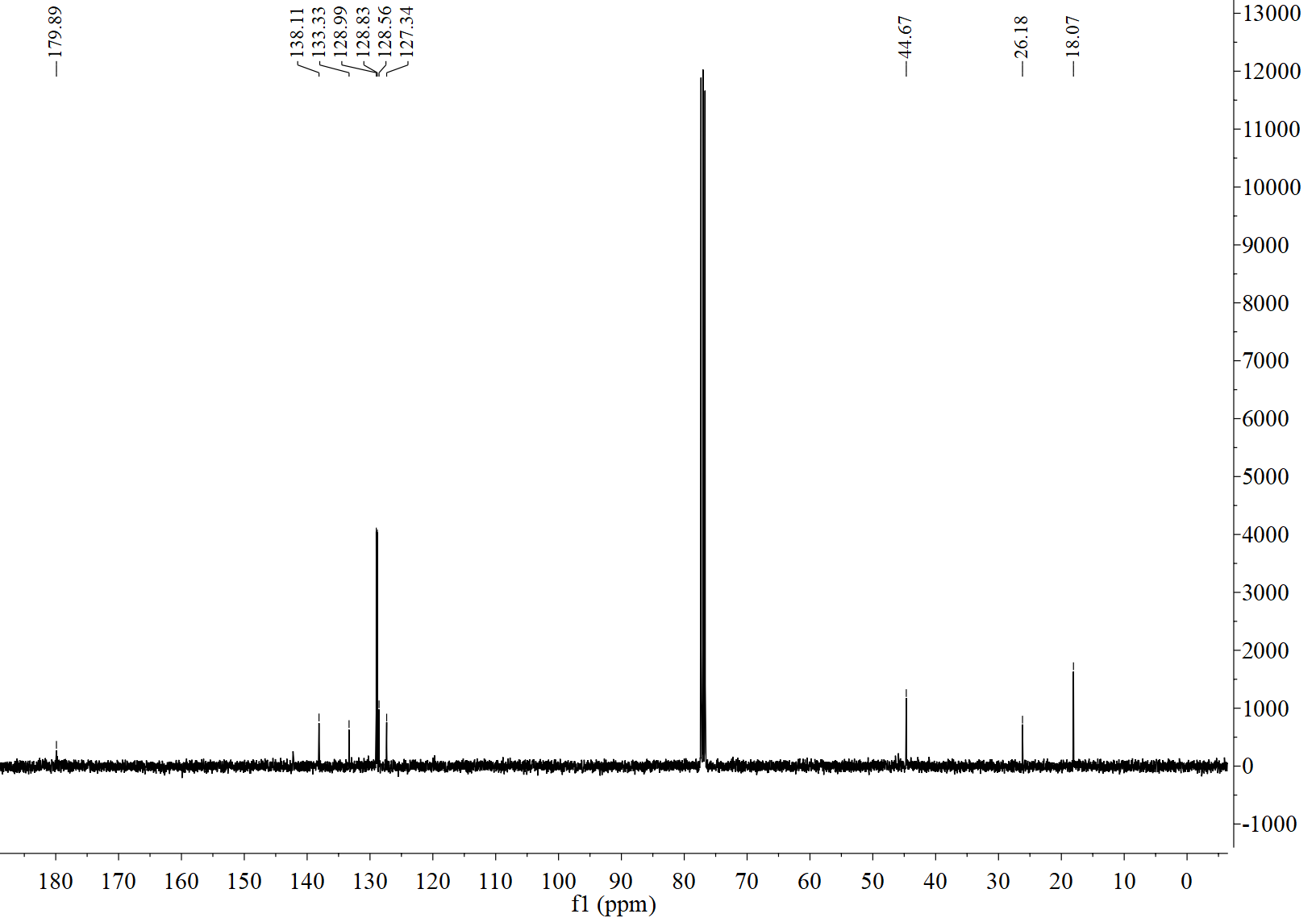


**Figure S99:** ^13^C-NMR (101 MHz, CDCl_3_) of 2-(4-chlorophenyl)propanoic acid (**1h**).


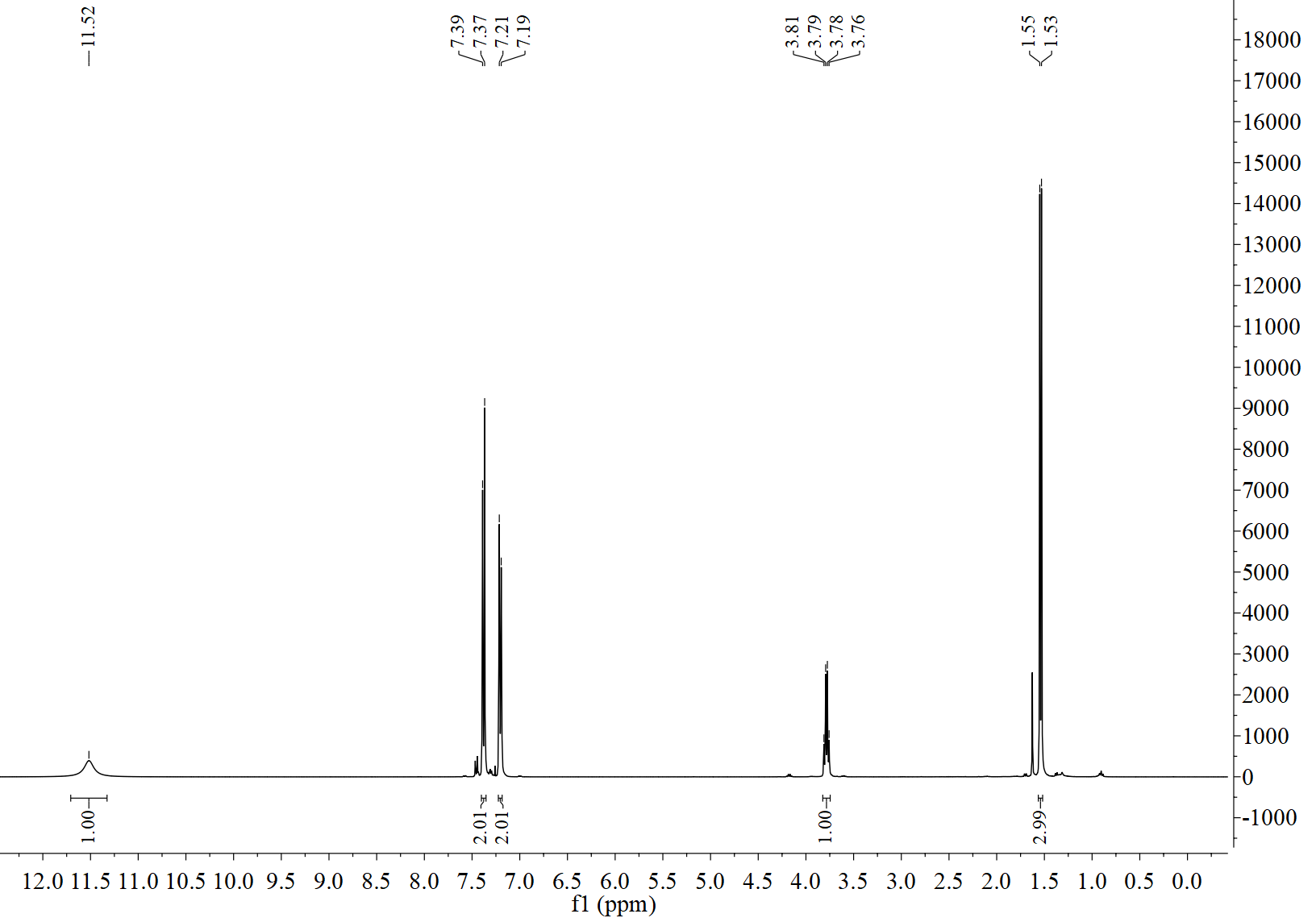


**Figure S100:** ^1^H-NMR (400 MHz, CDCl_3_) of 2-(4-trifluoromethoxy)phenyl)propanoic acid (**1i**).


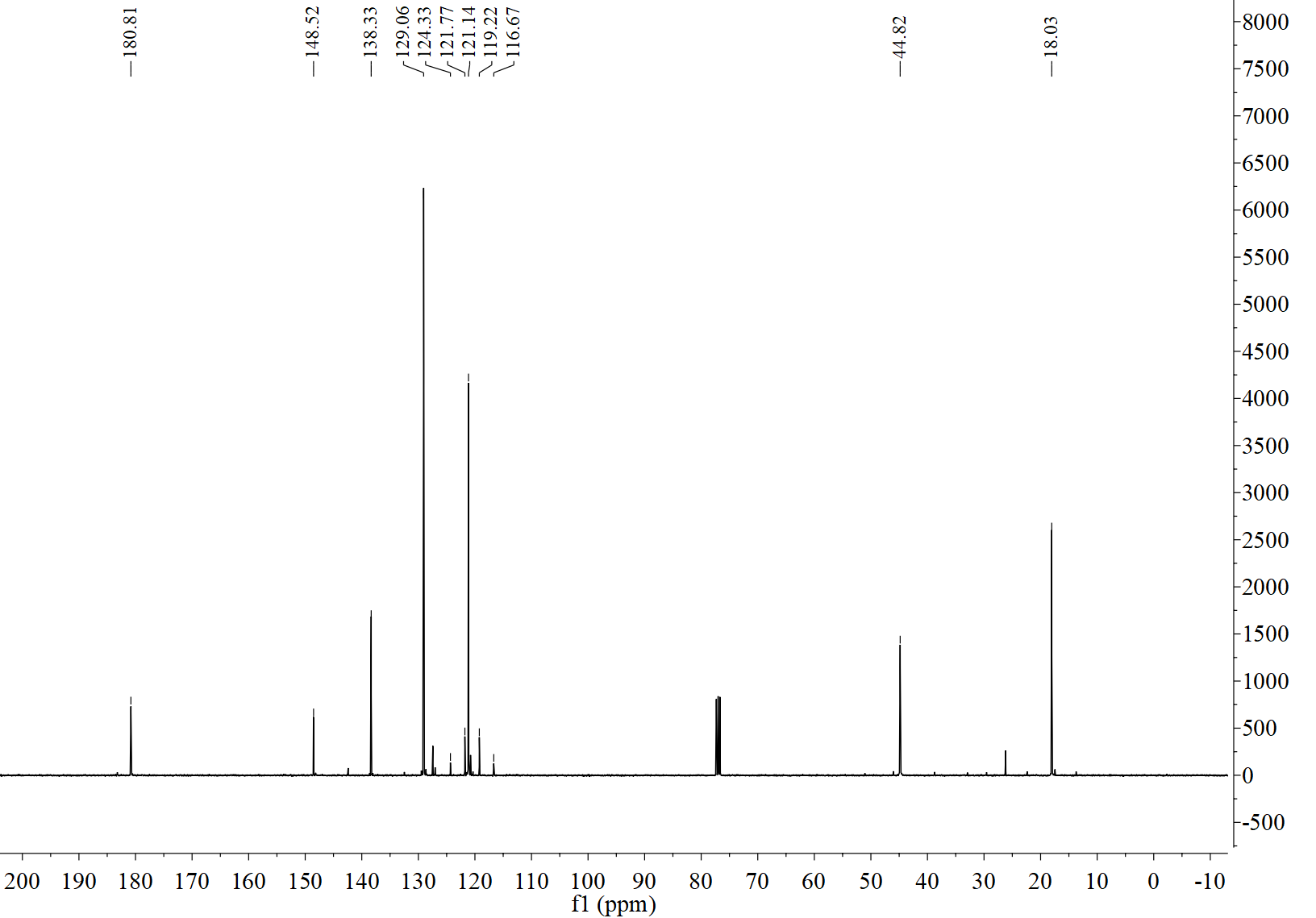


**Figure S101:** ^13^C-NMR (101 MHz, CDCl_3_) of 2-(4-trifluoromethoxy)phenyl)propanoic acid (**1i**).


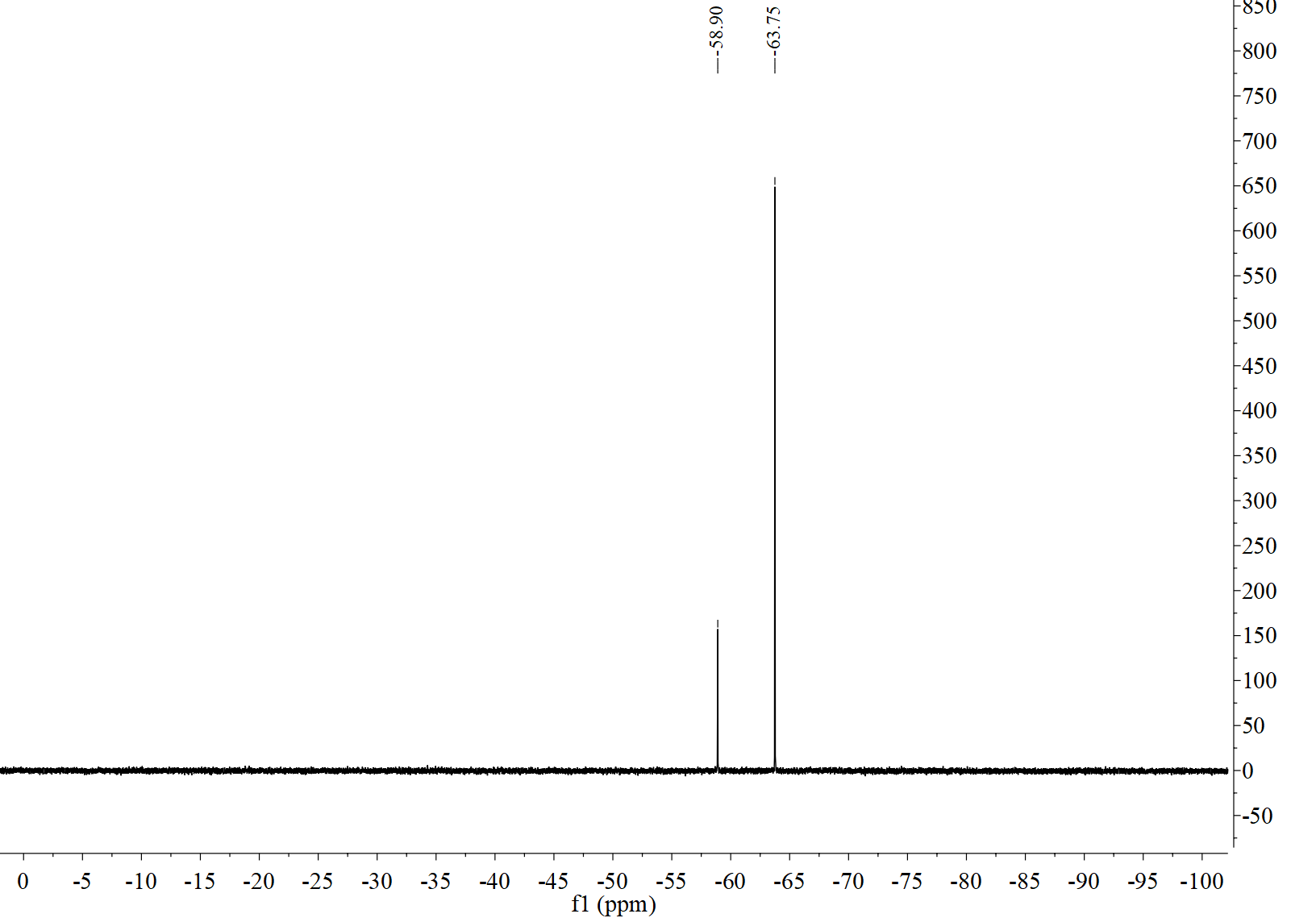


PhCF_3_

**Figure S102:** ^19^F-NMR (376 MHz, CDCl_3_) of 2-(4-trifluoromethoxy)phenyl)propanoic acid (**1i**) with PhCF_3_ as internal standard.

**Figure S103:** ^1^H-NMR (400 MHz, CDCl_3_) of 2-phenylhexanoic acid (**1m**).

**Figure S104:** ^13^C-NMR (101 MHz, CDCl_3_) of 2-phenylhexanoic acid (**1m**).

**Figure S105:** ^1^H-NMR (400 MHz, CDCl_3_) of 2,3-diphenylpropanoic acid (**1n**).

**Figure S106:** ^13^C-NMR (101 MHz, CDCl_3_) of 2,3-diphenylpropanoic acid (**1n**).

**Figure S107:** ^1^H-NMR (400 MHz, CDCl_3_) of 3-cyclohexyl-2-phenylpropanoic acid (**1o**).

**Figure S108:** ^13^C-NMR (101 MHz, CDCl_3_) of 3-cyclohexyl-2-phenylpropanoic acid (**1o**).

**Figure S109:** ^1^H-NMR (400 MHz, CDCl_3_) of 2-cyclohexyl-2-phenylacetic acid (**1p**).

**Figure S110:** ^13^C-NMR (101 MHz, CDCl_3_) of 2-cyclohexyl-2-phenylacetic acid (**1p**).

**Figure S111:** ^1^H-NMR (400 MHz, CDCl_3_) of 2-(naphthalen-1-yl)propanoic acid (**1q**).

**Figure S112:** ^13^C-NMR (101 MHz, CDCl_3_) of 2-(naphthalen-1-yl)propanoic acid (**1q**).

**Figure S113:** ^1^H-NMR (400 MHz, CDCl_3_) of 1-phenylcyclopentane-1-carboxylic acid (**S1**).

**Figure S114:** ^13^C-NMR (101 MHz, CDCl_3_) of 1-phenylcyclopentane-1-carboxylic acid (**S1**).

**Figure S115:** ^1^H-NMR (400 MHz, CDCl_3_) of 2-phenylpent-4-enoic acid (**S2**).

**Figure S116:** ^13^C-NMR (101 MHz, CDCl_3_) of 2-phenylpent-4-enoic acid (**S2**).

**Figure S117:** ^1^H-NMR (400 MHz, CDCl_3_) of 2-(thiophen-2-yl)propanoic acid (**S3**).

**Figure S118:** ^13^C-NMR (101 MHz, CDCl_3_) of 2-(thiophen-2-yl)propanoic acid (**S3**).

**Figure S119:** ^1^H-NMR (400 MHz, DMSO-*d*_6_) of 4-trifluoromethylbenzenesulfonamide (**2c**).

**Figure S120:** ^13^C-NMR (101 MHz, DMSO-*d*_6_) of 4-trifluoromethylbenzenesulfonamide (**2c**).

PhCF_3_

**Figure S121:** ^19^F-NMR (376 MHz, DMSO-*d*_6_) of 4-trifluoromethylbenzenesulfonamide (**2c**) with PhCF_3_ as internal standard.

**Figure S122:** ^1^H-NMR (400 MHz, DMSO-*d*_6_) of 4-cyanobenzenesulfonamide (**2d**).

**Figure S123:** ^13^C-NMR (101 MHz, DMSO-*d*_6_) of 4-cyanobenzenesulfonamide (**2d**).

**Figure S124:** ^1^H-NMR (400 MHz, DMSO-*d*_6_) of 3,5-dichlorobenzenesulfonamide (**2f**).

**Figure S125:** ^13^C-NMR (101 MHz, DMSO-*d*_6_) of 3,5-dichlorobenzenesulfonamide (**2f**).

**Figure S126:** ^1^H-NMR (400 MHz, DMSO-*d*_6_) of 2,4,6-trimethylbenzenesulfonamide (**2g**).

**Figure S127:** ^13^C-NMR (101 MHz, DMSO-*d*_6_) of 2,4,6-trimethylbenzenesulfonamide (**2g**).

**Figure S128:** ^1^H-NMR (400 MHz, DMSO-*d*_6_) of naphthalene-1-sulfonamide (**2h**).

**Figure S129:** ^13^C-NMR (101 MHz, DMSO-*d*_6_) of naphthalene-1-sulfonamide (**2h**).

**Figure S130:** ^1^H-NMR (400 MHz, DMSO-*d*_6_) of methyl 4-sulfamoylbenzoate (**2i**).

**Figure S131:** ^13^C-NMR (101 MHz, DMSO-*d*_6_) of methyl 4-sulfamoylbenzoate (**2i**).

**Figure S132:** ^1^H-NMR (400 MHz, DMSO-*d*_6_) of 4’fluoro-[1,1’-biphenyl]-4-sulfonamide (**2k**).

**Figure S133:** ^13^C-NMR (101 MHz, DMSO-*d*_6_) of 4’fluoro-[1,1’-biphenyl]-4-sulfonamide (**2k**).

PhCF_3_

**Figure S134:** ^19^F-NMR (376 MHz, DMSO-*d*_6_) of 4’fluoro-[1,1’-biphenyl]-4-sulfonamide (**2k**) with PhCF_3_ as internal standard.

**Figure S135:** ^1^H-NMR (400 MHz, CDCl_3_) of *N*-phenylcyclopropanesulfonamide (**2m**).

**Figure S136:** ^13^C-NMR (101 MHz, CDCl_3_) of *N*-phenylcyclopropanesulfonamide (**2m**).

**Figure S137:** ^1^H-NMR (400 MHz, CDCl_3_) of *N*-phenylcyclopentanesulfonamide (**2n**).

**Figure S138:** ^13^C-NMR (101 MHz, CDCl_3_) of *N*-phenylcyclopentanesulfonamide (**2n**).

**Figure S139:** ^1^H-NMR (400 MHz, DMSO-*d*_6_) of methyl 2,3-dimethoxy-5-sulfamoylbenzoate (**2o**).

**Figure S140:** ^13^C-NMR (101 MHz, DMSO-*d*_6_) of methyl 2,3-dimethoxy-5-sulfamoylbenzoate (**2o**).

**Figure S141:** ^1^H-NMR (400 MHz, DMSO-*d*_6_) of 4-nitrobenzenesulfonamide (**2q**).

**Figure S142:** ^1^H-NMR (400 MHz, DMSO-*d*_6_) of 4-nitrobenzenesulfonamide (**2q**).
